# Synthesis and Neurotropic Activity of New 5-Piperazinopyrazolo[3,4-*c*]-2,7-naphthyridines and Isoxazolo[5,4-*c*]-2,7-naphthyridines

**DOI:** 10.3390/ph18040597

**Published:** 2025-04-19

**Authors:** Samvel N. Sirakanyan, Elmira K. Hakobyan, Athina Geronikaki, Domenico Spinelli, Anthi Petrou, Victor G. Kartsev, Hasmik A. Yegoryan, Hasmik V. Jughetsyan, Mariam E. Manukyan, Ruzanna G. Paronikyan, Tatevik A. Araqelyan, Anush A. Hovakimyan

**Affiliations:** 1 Scientific Technological Center of Organic and Pharmaceutical Chemistry of National Academy of Science of Republic of Armenia, Institute of Fine Organic Chemistry of A.L. Mnjoyan, Yerevan 0014, Armenia; shnnr@mail.ru (S.N.S.); hasmik.yegoryan@mail.ru (H.A.Y.); jughetsyan2002@mail.ru (H.V.J.); m.manukyan.2022@internet.ru (M.E.M.); paronikyan.ruzanna@mail.ru (R.G.P.); tatev160396@mail.ru (T.A.A.); aaa.h.87@mail.ru (A.A.H.); 2 Department of Pharmacy, School of Health, Aristotle University of Thessaloniki, 54124 Thessaloniki, Greece; ipetrou@pharm.auth.gr; 3 Dipartimento di Chimica G. Ciamician, Alma Mater Studiorum-Università di Bologna, Via F. Selmi 2, 40126 Bologna, Italy; 4 InterBioScreen, Moscow 119019, Russia; vkartsev@ibscreen.chg.ru

**Keywords:** pyrazolo[3,4-*c*]-2,7-naphthyridines, pyrimido[1′,2′:1,5]pyrazolo[3,4-*c*]-2,7-naphthyridines, isoxazolo[5,4-*c*]-2,7-naphthyridines, neurotropic activity, docking studies

## Abstract

**Background/Objectives**: Approximately 1% of people worldwide suffer from epilepsy. The development of safer and more effective antiepileptic medications (AEDs) is still urgently needed because all AEDs have some unwanted side effects and roughly 30% of epileptic patients cannot stop having seizures when taking current AEDs. It should be noted that the derivatives of pyrazolo[3,4-*b*]pyridine are important core structures in many drug substances. The aim of this study is to synthesize new derivatives of piperazino-substituted pyrazolo[3,4-*c*]-2,7-naphthyridines and 9,11-dimethylpyrimido[1′,2′:1,5]pyrazolo[3,4-*c*]-2,7-naphthyridines for the evaluation of their neurotropic activity. **Methods**: The synthesis of the target compounds was performed starting from 1-amino-3-chloro-2,7-naphthyridines and using well-known methods. The structures of all the synthesized compounds were confirmed by spectroscopic data. Compounds were studied for their potential neurotropic activities (anticonvulsant, sedative, anti-anxiety, and antidepressive), as well as side effects, in 450 white mice of both sexes and 50 male Wistar rats. The anticonvulsant effect of the newly synthesized compounds was investigated by using the following tests: pentylenetetrazole, thiosemicarbazide-induced convulsions, and maximal electroshock. The psychotropic properties of the selected compounds were evaluated by using the following tests: the Open Field test, the Elevated Plus Maze (EPM), the Forced Swimming test, and Rotating Rod Test to study muscle relaxation. For the docking studies, AutoDock 4 (version 4.2.6) was used, as well as the structures of the GABA_A_ receptor (PDB ID: 4COF), the SERT transporter (PDB ID: 3F3A), and the 5-HT_1A_ receptor (PDB ID: 3NYA) obtained from the Protein Data Bank. **Results:** A series of piperazino-substituted pyrazolo[3,4-*c*]-2,7-naphthyridines (**3a**–**j**) and 9,11-dimethylpyrimido[1′,2′:1,5]pyrazolo[3,4-*c*]-2,7-naphthyridines (**4a**–**j**), as well as new heterocyclic systems, i.e., isoxazolo[5,4-*c*]-2,7-naphthyridines **6a**–**d**, were synthesized and evaluated for their neurotropic activity. The investigation showed that some of these compounds (**3a**,**b**,**d**,**f**–**i** and **4a**,**d**,**f**,**i**) display high anticonvulsant activity, especially in the test of antagonism with pentylenetetrazol, surpassing the well-known antiepileptic drug ethosuximide. Thus, the most active compounds in the pentylenpotetrazole test are **3h**, **3i**, and **4i**; the ED50 of compound **4i** is 23.8, and the therapeutic index is more than 33.6, which is the highest among these three active compounds. On the other hand, they simultaneously exhibit psychotropic (anxiolytic, antidepressant, or sedative) or behavioral depressant) effects. The effective compounds do not cause myorelaxation at the tested doses and have high therapeutic indices. Docking on the most active compounds, i.e., **3h**, **3i**, and **4i**, is in agreement with the experimental results. **Conclusions:** The studies reveled that some of these compounds (**3i**, **4a**, and **4i**) display high anticonvulsant and psychotropic activities. The most active compounds contained methyl and diphenylmethyl groups in the piperazine ring. The docking studies identified compounds **3i**, **4i**, and **4a** as the most potent anticonvulsants, showing strong affinity for GABA_A_, 5-HT_1A_ receptors, and the SERT transporter. Notably, compound **4i** formed two hydrogen bonds with Thr176 and Arg180 on GABA_A_ and exhibited a binding energy (−8.81 kcal/mol) comparable to that of diazepam (−8.90 kcal/mol). It also showed the strongest binding to SERT (−7.28 kcal/mol), stabilized by interactions with Gly439, Ile441, and Arg11. Furthermore, 4i displayed the best docking score with 5-HT_1A_ (−9.10 kcal/mol) due to multiple hydrogen bonds and hydrophobic interactions, supporting its potential as a dual-acting agent targeting both SERT and 5-HT_1A_.

## 1. Introduction

There are 4.6 million new cases of epilepsy worldwide each year. Safer, less toxic, and more effective antiepileptic drugs are urgently needed for patients with difficult-to-control seizures. The available drugs are symptomatically effective in only 60–70% of patients [1]. The primary tasks of the modern stage of epilepsy and epileptic syndromes are the nationwide introduction of new antiepileptic drugs (AEDs) with innovative mechanisms of action on the “target” of the pathological epileptic system and the use of new AEDs not only as an additional therapy for drug-resistant epilepsy but perhaps also for a faster transition to new forms of drugs in the earliest stages of ineffective treatment with basic drugs [2,3].

In the search for new neurotropic compounds in experimental psychopharmacology, it seems important and relevant to use animal models of both the pathology itself and its individual manifestations. Recently, in the treatment of antiepileptic drugs, mainly of the second generation, there is a tendency to optimize treatment aimed at the use of anticonvulsants with extended combined properties [4]. One of the leading tasks in this direction is the search for substances suitable not only for the treatment of mental disorders but also for the prevention of stressful situations and functional overloads in healthy people.

Recent literature data confirmed that pyrazolo[3,4-*b*]pyridine derivatives are important core structures in many drug substances [5,6,7,8,9,10,11,12,13,14]. This heterocyclic structure has always been the focus of attention of synthetic organic chemists, who have always been looking for new and effective methods for synthesizing this class of compounds [14,15,16,17]. Up to now, a very large number of pyrazole derivatives have been synthesized and evaluated for their biological activity [18,19]. In particular, pyrazolo[3,4-*b*]pyridine derivatives have been reported as potent antitumor [5,6,7], antimicrobial [7,8,9,10], anti-inflammatory [14], and nervous system agents [14]. A recent review of the biomedical applications of 1*H*-pyrazolo[3,4-*b*]pyridines has highlighted the importance of this class of compounds in medicinal and pharmaceutical chemistry [14].

**Figure 1:**
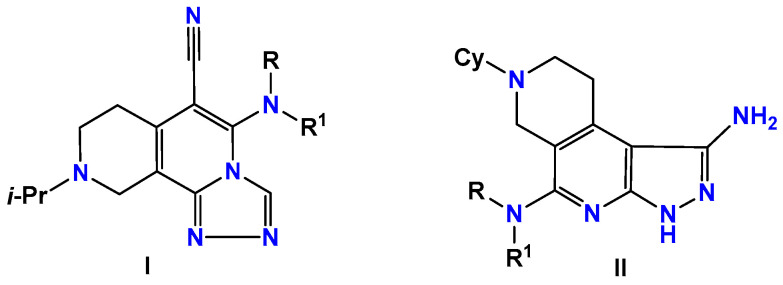
2,7-Naphthyridine derivatives with neurotropic activity. R, R^1^ = H, alkyl; RNR^1^ = cyclic amine.

On the other hand, it should be noted that compounds with a 2,7-naphthyridine scaffold have a wide spectrum of biological activity, especially cytotoxic and antitumor activities, as reported by a recent review [20]. Some of these compounds are also potent and selective inhibitors of various enzymes and kinases [21,22,23]. However, until now, the 2,7-naphthyridine derivatives 1,2,4-triazolo[3,4-*a*]-2,7-naphthyridines **I** and pyrazolo[3,4-*c*]-2,7-naphthyridines **II**, with neurotrophic activity, have been reported only by us (Figure 1) [24,25]. 

Literature data showed that isoxazolo[5,4-*c*]pyridines and their derivatives are compounds with high biological potential [26,27,28,29]. It should be noted that these compounds are very close in their structure to pyrazolo[3,4-*c*]pyridines: isoxazolo[5,4-*c*]pyridines, instead of a nitrogen atom, contain an oxygen atom. Considering this, it was very interesting from both biological and theoretical points of view to synthesize this type of compounds.

Thus, based on the above information and the fact that antiepileptic drugs have many side effects, herein, we report the synthesis of several new pyrazolo[3,4-*b*]pyridines, their fused derivatives (pyrimido[1′,2′:1,5]pyrazolo[3,4-*c*]-2,7-naphthyridines), and new heterocyclic systems (isoxazolo[5,4-*c*]-2,7-naphthyridines), with the aim of identifying more active and less neurotoxic and toxic substances.

## 2. Results and Discussion

### 2.1. Chemistry

**Scheme 1:**
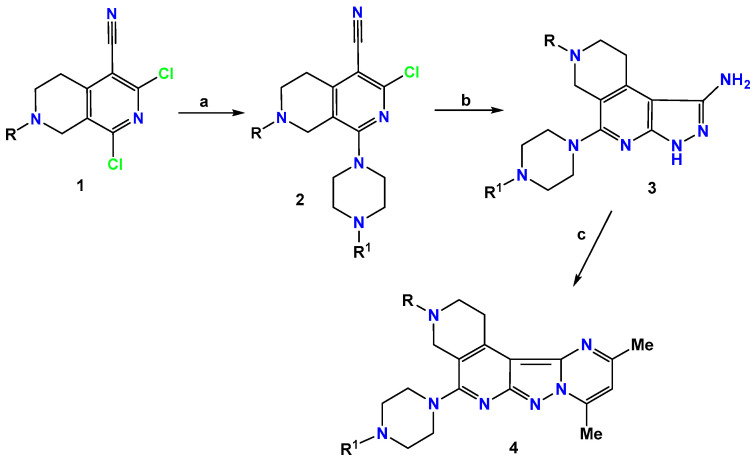
Reagents and conditions: (**a**) amine, EtOH, Et_3_N, refluxed for 5 h; (**b**) N_2_H_4_**^.^**H_2_O, BuOH, refluxed for 8 h; (**c**) MeCOCH_2_COMe, refluxed for 5 h.

The synthetic procedures for the synthesis of compounds such as pyrazolo[3,4-*c*]-2,7-naphthyridines and 9,11-dimethylpyrimido[1′,2′:1,5]pyrazolo[3,4-*c*]-2,7-naphthyridines were well developed and reported by us previously [30,31]. 1,3-Dichloro-2,7-naphthyridines **1** [32] were reacted with substituted piperazines in absolute ethanol, thus leading to the substitution of the chlorine atom at the first position of the 2,7-naphthyridine ring with the formation of 3-chloro-2,7-naphthyridines **2a**–**j** in high yields: Y = 70–8% (Scheme 1). It should be noted that previously, we carried out ESP charge calculations for compounds **1a**–**c** involved in the S_N_Ar reactions, and the studies revealed a negligible difference between the charges of the two carbon atoms bound to the chlorine atoms, so the different reactivity of the two sites in the 2,7-naphthyridine ring can be explained only by the steric hindrance caused by the cyano group [33].

Further, the reaction of compounds **2** and hydrazine hydrate in butanol under reflux was carried out. Thus, by primarily using hydrazine, we made a nucleophilic substitution of the chlorine atom at the third position of the 2,7-naphthyridine ring, followed by intramolecular cyclization, leading to the corresponding 7-*R*-5-(4-*R*^1^piperazin-1-yl)-6,7,8,9-tetrahydro-3*H*-pyrazolo[3,4-*c*]-2,7-naphthyridin-11-amines **3a**–**j** in high yields: Y = 76–88% (Scheme 1 and Table 1).

Based on these newly synthesized pyrazolo[3,4-*c*]-2,7-naphthyridines, a pyrimidine ring was constructed via a condensation/cyclization process known as the Knorr synthesis of pyrazoles [34]. Thus, the treatment of pyrazolo[3,4-*c*]-2,7-naphthyridines **3** with acetylacetone [31,35] afforded tetracyclic 3-*R*-9,11-dimethyl-5-(4-*R*^1^piperazin-1-yl)1,2,3,4-tetrahydropyrimido[1′,2′:1,5]pyrazolo[3,4-*c*]-2,7-naphthyridines **4a**–**j** in moderate yields: Y = 67–72% (Scheme 1 and Table 1).

The structures of the obtained compounds **2**, **3**, and **4** were confirmed by NMR, IR, and MS spectroscopic data and by elemental analysis. The IR spectra of compound **3** did not show the characteristic absorption bands of the cyano group in the region at ν 2215–2221 cm^−1^, thus confirming the cyclization process, and in the ^1^H NMR spectra, the protons of the NH and NH_2_ groups were present at 11.30–11.42 ppm and 4.47–4.54 ppm, respectively.

As expected, in the IR spectra of compounds **4**, the absorption bands of the NH and NH_2_ groups were absent, while in the ^1^H NMR spectra of these compounds, the protons of two singlet methyl groups at 2.61–2.67 ppm and 2.83–2.90 ppm and one CH singlet proton at 6.98–7.27 ppm were observed, proving the cyclization process.

**Scheme 2:**
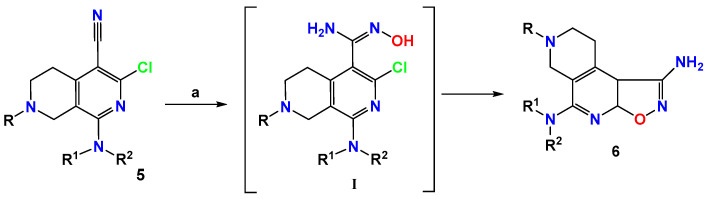
Reagents and conditions: (**a**) NH_2_OH**^.^**HCl, NaOEt, EtOH, refluxed for 15 h.

By continuing our work, we have also succeeded at synthesizing new heterocyclic systems: isoxazolo[5,4-*c*]-2,7-naphthyridines **6a**–**d**. Thus, 7-*R*-1-amino-3-chloro-5,6,7,8-tetrahydro-2,7-naphthyridine-4-carbonitriles **5a**–**d** [33] were reacted with hydroxylamine hydrochloride in absolute ethanol in the presence of sodium ethoxide [26,27]. The coupling reaction between the cyano group and hydroxylamine occurred with the formation of an intermediate product: amidoxims **I**. Then, as a result of the intramolecular cyclization, new heterocyclic systems, i.e., isoxazolo[5,4-*c*]-2,7-naphthyridines **6a**–**d**, were formed in moderate yields (Scheme 2 and Table 2).

As in the previous two cases (compounds **3** and **4**), here too, the IR spectra provide primarily important information about the structure of the obtained compounds. Thus, the infrared spectra of compounds **6a**–**d** demonstrated the absorption bands at 3205–3468 cm^−1^ typical of the amino group, while the strong absorption bands of the cyano substituent were missing, thus proving that the cyclization process had occurred. In the ^1^H NMR spectra, the singlet signals of the NH_2_ group at 5.35–5.54 ppm were observed. The structures of these new systems were also supported by the ^13^C NMR spectra and MS data and are in agreement with the proposed structures.

### 2.2. Biological Evaluation

#### 2.2.1. Evaluation of Anticonvulsant Activity

All twenty synthesized compounds were evaluated for possible anticonvulsant activity [36,37,38,39,40]. The evaluation revealed that they exhibit variable degrees of antagonism against pentylenetetrazole (PTZ) varying between 20% and 80%. Compounds **3a**,**b**,**d**,**f**–**i** and **4a**,**d**,**f**,**i** with the prevention of 60–80% of pentylenetetrazole seizures at a dose of 50 mg/kg, appeared to be the most active. The rest of the compounds (**3c**,**e**,**j**; **4b**,**c**,**e**,**g**,**h**,**j**; and **6a**–**d**) showed up to 20–40% activity and were not further studied.

The data on the anticonvulsant activity of the eleven most active compounds and the reference drugs ethosuximide and diazepam [41,42] in mice are shown in Table 1. The evaluation revealed that the studied compounds exhibit greater anticonvulsant activity against pentylenetetrazole compared with ethosuximide but are less effective than diazepam. However, unlike diazepam, these compounds do not induce muscle relaxation [43] at the studied doses of 12.5, 50, and 200 mg/kg. Their therapeutic indexes (TIs) are significantly higher than that of the reference drug, ethosuximide (see Table 3).

Among the studied compounds, the most active ones—**3a**,**b**,**d**,**f**–**i** and **4a**,**d**,**f**,**i**—were selected for further evaluation using the thiosemicarbazide (TSC) seizure model, which affects GABA exchange. At a dose of 100 mg/kg, these compounds increase the latency of thiosemicarbazide (TSC) seizures by 1.1 to 2.0 times compared with the control. Ethosuximide shows a similar effect, extending the latency of TSC convulsions. In contrast, diazepam is ineffective in this model. 

According to the antagonism data with pentylenetetrazol and the therapeutic indices of the compounds presented in Table 3, it is obvious that the most active compounds are **3h**, **3i**, and **4i**. The ED50 of compound **4i** is 23.8, and the therapeutic index is more than 33.6, which is the highest among these three active compounds.

According to the MES test, the studied compounds, as well as the reference drugs, did not show anticonvulsant effects. They were ineffective in protecting against tonic and clonic seizures induced by MES.

**Figure 2:**
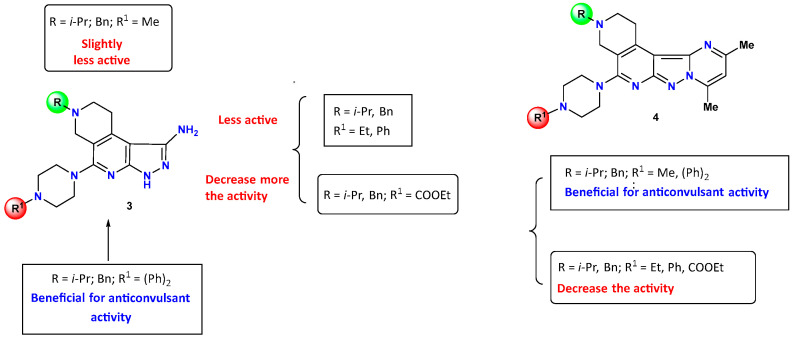
Structure–activity relationship study on anticonvulsant activity for pyrazolo [3,4-*c*]-2,7-naphthyridines **3** and pyrimido[1′,2′:1,5]pyrazolo[3,4-*c*]-2,7-naphthyridines **4**.

The structure–activity relationship study on anticonvulsant activity revealed that the presence of the diphenylmethyl group in the piperazine ring is beneficial for anticonvulsant activity for both series of compounds: pyrazolo[3,4-*c*]-2,7-naphthyridines **3d**,**i** and pyrimido[1′,2′:1,5]pyrazolo[3,4-*c*]-2,7-naphthyridines **4d**,**i**. Moreover, compounds containing the benzyl group at the seventh position of the 2,7-naphthyridine ring, **3i** and **4i**, were more active than compounds with an isopropyl group at the same position, **3d** and **4d** (Figure 2). It can also be argued that the cyclization of the pyrimidine ring in these cases did not essentially affect the activity of the compounds.

The introduction of the methyl group in the piperazine ring (compounds **3a**,**f** and **4a**,**f**) slightly decreased the activity. The replacement of the methyl group with ethyl and phenyl in pyrazolo[3,4-*c*]-2,7-naphthyridines in general led to compounds **3b**,**c**,**g**,**h** with further decreased activity.

Pyrazolo[3,4-*c*]-2,7-naphthyridines **3e**,**j** and pyrimido[1′,2′:1,5]pyrazolo[3,4-*c*]-2,7-naphthyridines **4e**,**j** with the COOEt substituent in the piperazine ring were the least active compounds, and in these cases, the cyclization process also did not affect the activity of the compounds (Figure 2).

#### 2.2.2. Evaluation of Psychotropic Effects

**Figure 3:**
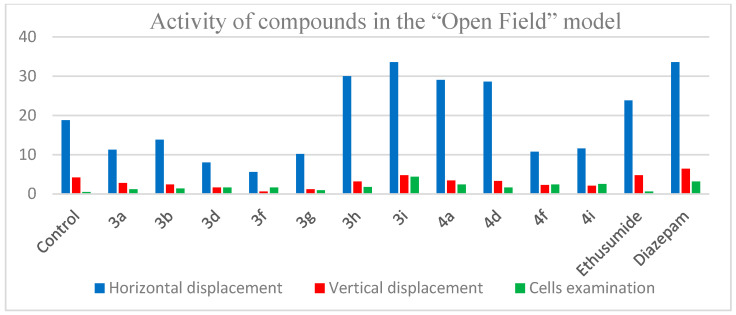
Graphical representation of Table 4.

The 11 most potent compounds, i.e., **3a**,**b**,**d**,**f**–**i** and **4a**,**d**,**f**,**i**, were assessed for their psychotropic properties at a dose of 50 mg/kg in the Open Field [44,45], Elevated Plus Maze (EPM) [46,47], and Forced Swimming [48] tests. This dose was selected because the ED50 of these compounds is within the 50 mg/kg range according to the confidence intervals. Research on motor behavior in rats was conducted by using a modified Open Field model. In this behavioral model, the rat control group showed an average of 18.8 horizontal movements and 4.2 vertical movements and examined 0.5 cells (see Table 4 and Figure 3). The compounds under study induced changes in behavioral indices compared with the control group, resulting in noticeable alterations in both horizontal and vertical movements of the animals after the compound’s administration. Compounds **3h**, **3i**, **4a**, and **4d** caused a statistically significant increase in the horizontal movements of the animals, indicating an activating effect, while the other compounds decreased horizontal movement.

Regarding vertical movements, compound **3i** significantly increased them, indicating an activation effect on behavior. Compounds that reduce the horizontal and vertical movements of animals exhibit a sedative effect. However, all the selected compounds, especially **3f**, **3i**, **4a**, and **4i**, significantly increased the number of cell examinations compared with the control, which may indicate anti-anxiety effects (see Table 4 and Figure 3).

The most active of these was compound **3i**, which increased both horizontal and vertical displacements, as well as the number of cell examinations. Ethosuximide, at an effective dose of 200 mg/kg, had no impact on any of the research activity indicators. In contrast, diazepam (2 mg/kg) significantly increased the number of cells examined compared with the control group, demonstrating a pronounced anti-anxiety effect. Similar to the studied compounds **3h**, **3i**, **4a**, and **4d**, diazepam also increased horizontal movements, exhibiting an activating effect on behavior. Compound **4i**, a product of the cyclization of **3i**, did not alter the horizontal and vertical displacements but led to the suppression of search activities compared with **3i**.

**Figure 4:**
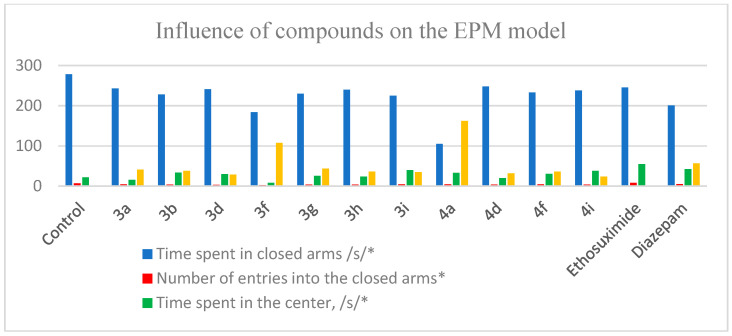
Graphical representation of Table 5. * *p* ≤ 0.05 probability level.

In the Elevated Plus Maze (EPM) model, control animals predominantly remained in the closed arms—for a total of 278 s (see Table 5, Figure 4). Some of investigated compounds (**3b**, **3i**, and **4i**), along with ethosuximide and diazepam, significantly increased the time spent by the experimental animals in the center of the maze. Furthermore, the tested compounds significantly reduced the time spent in the closed arms and the number of entries into the closed arms. Following administration of these compounds (**4i**, **3f**, and **4a**) and diazepam, the experimental animals spent 24, 108, and 162 s, respectively, in the open arms, in contrast to the control animals and those receiving ethosuximide at a dose of 200 mg/kg (see Table 5, Figure 4), suggesting an anxiolytic effect for these compounds. Compounds **3f** and **4a** showed a more pronounced (up to two–three times) anxiolytic effect compared with diazepam.

One of the most widely utilized tests is the Forced Swimming test (FST), sometimes referred to as Porsolt’s test. Based on the idea that immobility is a gauge of behavioral despair, the FST is used to track depressive-like behavior.

**Figure 5:**
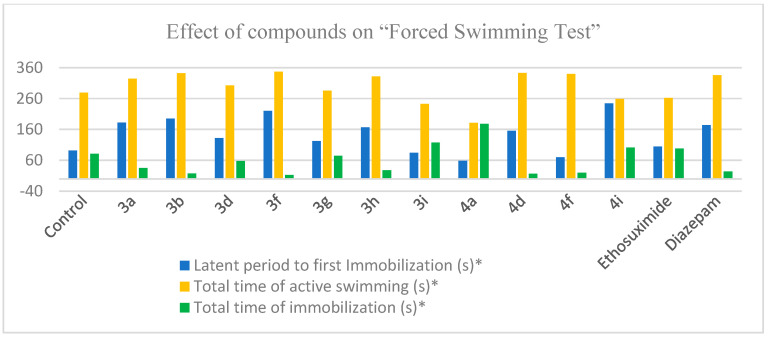
Graphical representation of Table 6. * *p* ≤ 0.05 probability level.

The first immobilization in control mice in the Forced Swimming model (Table 6 and Figure 5) happened at 92 s. At a dose of 50 mg/kg, several of the investigated derivatives (**3a**, **3b**, **3d**, **3f**, **3g**, **3h**, **4d**, and **4i**) statistically significantly extended the latent period of the first immobility and shortened its duration. For many compounds, the overall immobilization duration reduced; compound **3f** had the lowest value, 12.5 s. These compounds and diazepam increased the total time of active swimming, suggesting that they exert an antidepressant effect in the same manner as diazepam. Particularly noteworthy are compounds **3b**, **3f**, **3h**, **4d**, and **4f**. The results obtained when using 200 mg/kg ethosuximide are consistent with the control findings.

According to structure–activity relationship study, the presence of a benzyl substituent at seventh position of 6,7,8,9-tetrahydro-3*H*-pyrazolo[3,4-*c*][3,8]naphthyridin-1-amine (**3f**), as well as 4-methylpiperazine, is beneficial for the antidepressant activity in the Forced Swimming model. Pyrazolo[3,4-*c*]-2,7-naphthyridines **3** showed higher activity in this test than pyrimido[1′,2′:1,5]pyrazolo[3,4-*c*]-2,7-naphthyridines **4**.

### 2.3. Molecular Docking

#### 2.3.1. Docking to GABA_A_ Receptor—Prediction of Mechanism of Anticonvulsant and Anxiolytic Activity

Antiepileptic drugs often target GABA_A_ receptors by blocking sodium channels or enhancing γ-aminobutyric acid (GABA) activity [49,50]. To gain deeper insights into the molecular mechanisms underlying GABA_A_ receptor inhibition and explore interactions within its active site, docking studies were performed on all tested compounds.

The GABA_A_ receptor’s crystal structure was obtained from the Protein Data Bank (PDB) under ID 4COF for docking experiments [51]. The receptor’s X-ray diffraction structure had a resolution of 2.97 Å, with R and R-free values of 0.206 and 0.226, respectively. As part of the docking study validation, the original co-crystal ligand, benzamidine, was removed and re-docked to the catalytic site by using the same preparation parameters applied to the tested compounds. The root-mean-square deviation (RMSD) between the co-crystal and re-docked pose was determined to be 0.34.

**Figure 6:**
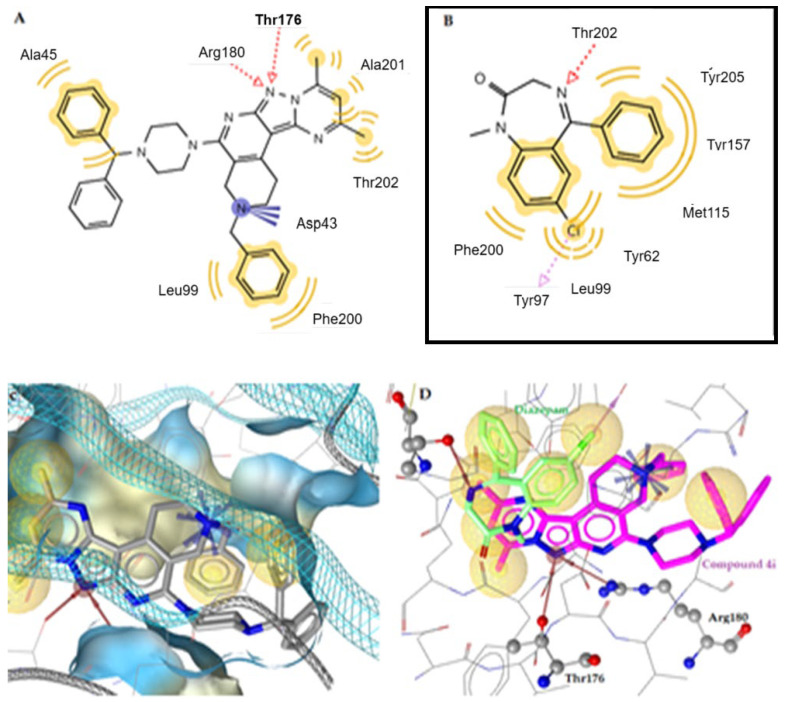
(**A**) Two-dimensional interaction diagram of compound **4i** docking pose, (**B**) two-dimensional interaction diagram of Diazepam docking pose, and (**C**) docked pose of compound **4i** in GABA_A_ receptor. (**D**) Superposition of compound **4i** (magenta) and diazepam (green) in GABA_A_ receptor. Hydrogen bonds are shown as red dotted arrows and hydrophobic interactions as yellow spheres.

The results are summarized in Table 7. It was found that compound **4i** formed two hydrogen bonds with the residues Thr176 (N···H, 3.78 Å) and Arg180 (N···H, 3.26 Å), whereas diazepam only formed one hydrogen bond with Thr202 (N···H, 2.67), according to Figure 6. The complex ligand–enzyme was further stabilized by interacting hydrophobically with the residues Ala45, Leu99, Val198, Phe200, Ala201, and Thr202.

#### 2.3.2. Docking to SERT Transporter and 5-HT_1A_ Receptor

The two primary kinds of antidepressant drugs are selective serotonin reuptake inhibitors (SSRIs) and tricyclic antidepressants [52]. These medications work by blocking the serotonin (5-HT) transporter (SERT), which stops serotonin from entering pre-synaptic neurons again. To stop serotonergic neurotransmission, serotonin must be removed from the synaptic cleft by SERT, a transmembrane protein found in the pre-synaptic neuron membrane. Increased serotonin levels activate 5-HT1A receptors, leading to reduced serotonergic neurotransmission and delaying the onset of antidepressant effects [53,54]. This delay persists until the 5-HT1A receptors become desensitized, allowing serotonin release to return to normal.

Considering these factors, docking studies were conducted on both the SERT transporter and the 5-HT1A receptor to evaluate whether the tested compounds function as dual inhibitors of SERT and antagonists of pre-synaptic auto-inhibitory 5-HT1A receptors.

Since no crystal structure of the human SERT transporter is available in the Protein Data Bank (PDB), the X-ray crystal structure of LeuT bound to L-Tryptophan (PDB ID: 3F3A) [55], a prokaryotic homolog of SERT, was used for the docking studies. The results of these studies are presented in Table 8. As a validation step, the co-crystal ligand L-Tryptophan was removed and re-docked to the protein’s binding site by using the same parameters as those applied to the tested compounds (RMSD = 0.86).

**Figure 7:**
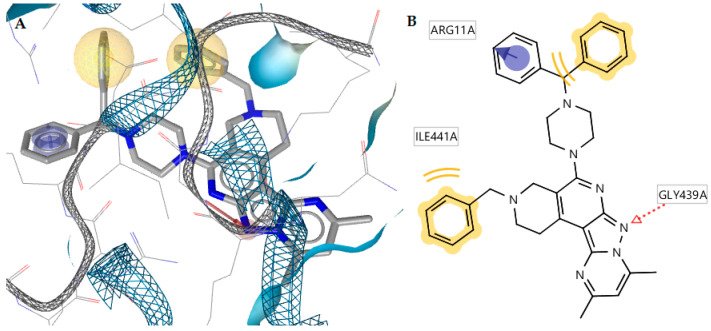
(**A**) Docked pose of compound **4i** and SERT transporter complex; (**B**) 2D ligand interaction diagram of compound **4i**. Hydrogen bonds are shown as red dotted arrows and hydrophobic interactions as yellow spheres.

Among the tested compounds, compound **4i** emerged as one of the most promising. It formed a hydrogen bond with Gly439 and hydrophobic interactions with Ile441, and there was an aromatic interaction between its benzene moiety and Arg11 in the SERT transporter (Figure 7). These interactions suggest good binding affinity, potentially contributing to its antagonistic activity at the transporter’s site.

The crystal structure of the human β2-adrenergic receptor bound to the antagonist alprenolol (PDB ID: 3NYA) was utilized for the docking studies on the 5-HT1A receptor [56,57]. To ensure the reliability of the docking parameters, alprenolol, the original co-crystallized ligand, was re-docked to the protein’s catalytic site, yielding a root-mean-square deviation (RMSD) of 0.98 between the experimental and re-docked conformations.

**Figure 8:**
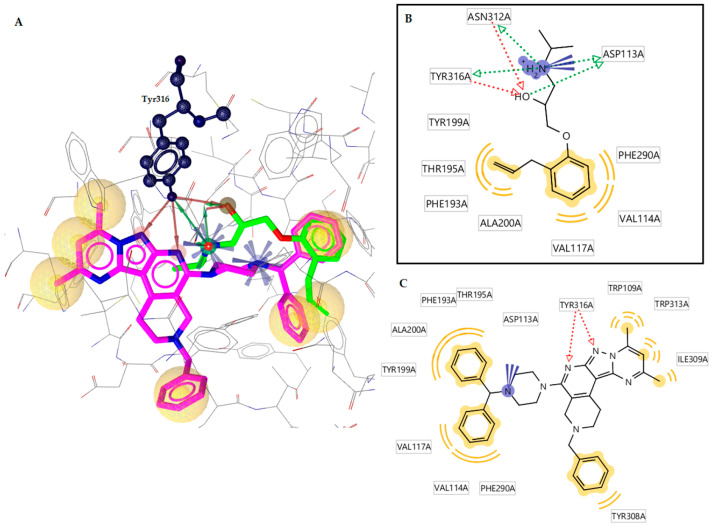
(**A**) Superposition of compound **4i** (magenta) and alprenolol (green) into 5-HT_1A_ receptor. (**B**) Two-dimensional ligand interaction diagram alprenolol into 5-HT_1A_ receptor. (**C**) Two-dimensional ligand interaction diagram for docked compound **4i**. Hydrogen bonds are shown as red dotted arrows and hydrophobic interactions as yellow spheres.

All tested compounds (Table 9) were docked to the orthosteric binding site of the 5-HT_1A_ receptor, with compound **4i** achieving the best docking score (−9.10 kcal/mol). This was attributed to the formation of two hydrogen bonds with the Tyr316 residue. Additionally, the compound interacted hydrophobically with several residues, including Trp109, Trp313, Ile309, Thr195, Phe193, Val114, The290, Tyr308, Val117, Tyr199, and Ala200 (Figure 8), similar to alprenolol (Figure 8A).

Finally, docking studies suggest that compound **4i** could serve as a dual-target agent, as it appears to effectively inhibit the SERT transporter while also strongly binding to the 5-HT_1A_ receptor.

## 3. Materials and Methods

### 3.1. Chemistry: Experimental Part

General information: All chemicals and solvents were of commercially high purity grade and purchased from Sigma Aldrich (Saint Louis, MO, USA). The ^1^H and ^13^C NMR spectra for compounds **2a**–**d**, **f**–**j**, **3a**–**j**, and **4a**–**f**, **h**–**j** were recorded in DMSO-*d*_6_/CCl_4_ (1/3) solution (300 MHz for ^1^H and 75 MHz for ^13^C) on a Mercury 300VX spectrometer (Varian Inc., Palo Alto, CA, USA). The ^1^H and ^13^C NMR spectra for compounds **6a**–**d** and **4g** were recorded on a BRUKER AVANCE NEO spectrometer (Bruker BioSpin GmbH, Rheinstetten, Germany) with operating frequencies of 400 and 100 MHz, respectively, in DMSO-*d*_6_/CCl_4_(1/3) and CDCl_3_ at 303 K. Chemical shifts were expressed as *δ* (parts per million) in relation to TMS as internal standard. The IR spectra were recorded on a Nicolet Avatar 330-FT-IR spectrophotometer (Thermo Nicolet, Foster, CA, USA) in vaseline, with *ν_max_* in cm^−1^. The MS spectra were recorded on a Waters XEVO G3 Q-Tof spectrometer (Waters Corporation Company, Milford, MA, USA). Elemental analyses were performed on an Elemental Analyzer Euro EA 3000 (EuroVector, Pavia, Italy). Melting points were determined on a MP450 melting point apparatus. Compounds **1a**,**b** [32] and **5a**–**d** [33] were already described. Physicochemical data for compound **2e** are not given; it did not crystallize and was isolated as an oil (yield of 73%).

#### 3.1.1. General Procedure for Synthesis of Compounds **2a**–**d**,**f**–**j**

A mixture of 1,3-dichloro-2,7-naphthyridine **1** (10 mmol), appropriate amine (11 mmol), and triethylamine (1.53 mL, 11 mmol) in absolute ethanol (60 mL) was heated under reflux for 5 h followed by the addition of water (50 mL) after completion of the reaction. The precipitate was filtered off, washed with water, dried, and recrystallized from ethanol.

7-Isopropyl-3-chloro-1-(4-methylpiperazine-1-yl)-5,6,7,8-tetrahydro-2,7-naphthyridine-4-carbonitrile (**2a**). Colorless solid, yield 76%, m.p. 139–140 °C. IR *ν*/cm^−1^: 2221 (C≡N). ^1^H NMR (300 MHz, DMSO-*d*_6_/CCl_4_, 1/3) δ 1.09 (d, *J* = 6.6 Hz, 6H, CH(CH_3_)_2_), 2.27 (s, 3H, NCH_3_), 2.43–2.48 (m, 4H, CH_3_N(CH_2_)_2_), 2.76 (t, *J* = 5.9 Hz, 2H, NCH_2_CH_2_), 2.88 (sep, *J* = 6.5 Hz, 1H, CH(CH_3_)_2_), 2.91 (t, *J* = 6.0 Hz, 2H, NCH_2_CH_2_), 3.31–3.36 (m, 4H, N(CH_2_)_2_), 3.38 (s, 2H, NCH_2_). ^13^C NMR (75 MHz, DMSO-*d*_6_/CCl_4_, 1/3) δ 18.02, 29.03, 44.13, 45.38, 48.18, 48.51, 53.14, 54.17, 100.43, 113.95, 119.85, 147.79, 150.69, 159.35. Anal. calcd. for C_17_H_24_ClN_5_: C 61.16; H 7.25; N 20.98%. Found: C 60.81; H 7.41; N 21.19%.

7-Isopropyl-3-chloro-1-(4-ethylpiperazine-1-yl)-5,6,7,8-tetrahydro-2,7-naphthyridine-4-carbonitrile (**2b**). Cream solid, yield 72%, m.p. 123–124 °C. IR *ν*/cm^−1^: 2217 (C≡N). ^1^H NMR (300 MHz, DMSO-*d*_6_/CCl_4_, 1/3) δ 1.08 (t, *J* = 7.2 Hz, 3H, NCH_2_CH_3_), 1.09 (d, *J* = 6.5 Hz, 6H, CH(CH_3_)_2_), 2.41 (q, *J* = 7.2 Hz, 2H, NCH_2_CH_3_), 2.47–2.53 (m, 4H, C_2_H_5_N(CH_2_)_2_), 2.76 (t, *J* = 5.9 Hz, 2H, NCH_2_CH_2_), 2.89 (sep, *J* = 6.5 Hz, 1H, CH(CH_3_)_2_), 2.90 (t, *J* = 5.8 Hz, 2H, NCH_2_CH_2_), 3.32–3.37 (m, 4H, N(CH_2_)_2_), 3.38 (s, 2H, NCH_2_). ^13^C NMR (75 MHz, DMSO-*d*_6_/CCl_4_, 1/3) δ 11.49, 18.01, 29.03, 44.09, 48.29, 48.56, 51.43, 51.96, 53.14, 100.36, 113.96, 119.76, 147.81, 150.64, 159.31. Anal. calcd. for C_18_H_26_ClN_5_: C 62.14; H 7.53; N 20.13%. Found: C 62.44; H 7.36; N 20.36%.

7-Isopropyl-3-chloro-1-(4-phenylpiperazine-1-yl)-5,6,7,8-tetrahydro-2,7-naphthyridine-4-carbonitrile (**2c**)**.** Cream solid, yield 78%, m.p. 198–199 °C. IR *ν*/cm^−1^: 2221 (C≡N). ^1^H NMR (300 MHz, DMSO-*d*_6_/CCl_4_, 1/3) δ 1.10 (d, *J* = 6.5 Hz, 6H, CH(CH_3_)_2_), 2.79 (t, *J* = 5.8 Hz, 2H, NCH_2_CH_2_), 2.87 (sep, *J* = 6.4 Hz, 1H, CH(CH_3_)_2_), 2.93 (t, *J* = 6.1 Hz, 2H, NCH_2_CH_2_), 3.26–3.32 (m, 4H, PhN(CH_2_)_2_), 3.46 (s, 2H, NCH_2_), 3.48–3.55 (m, 4H, N(CH_2_)_2_), 6.79 (t,t, *J* = 7.3, 1.2 Hz, 1H, Ph), 6.88–6.94 (m, 2H, Ph), 7.16–7.24 (m, 2H, Ph). ^13^C NMR (75 MHz, DMSO-*d*_6_/CCl_4_, 1/3) δ 18.02, 29.07, 44.07, 48.32, 48.36, 48.41, 53.15, 100.80, 113.90, 115.51, 119.17, 120.17, 128.37, 147.82, 150.36, 150.87, 159.33. Anal. calcd. for C_22_H_26_ClN_5_: C 66.74; H 6.62; N 17.69%. Found: C 67.08; H 6.79; N 17.47%.

7-Isopropyl-3-chloro-1-[4-(diphenylmethyl)piperazine-1-yl]-5,6,7,8-tetrahydro-2,7-naphthyridine-4-carbonitrile (**2d**). Cream solid, yield 75%, m.p. 184–185 °C. IR *ν*/cm^−1^: 2221 (C≡N). ^1^H NMR (300 MHz, DMSO-*d*_6_/CCl_4_, 1/3) δ 1.06 (d, *J* = 6.5 Hz, 6H, CH(CH_3_)_2_), 2.47–2.53 (m, 4H, Ph_2_N(CH_2_)_2_), 2.73 (t, *J* = 5.7 Hz, 2H, NCH_2_CH_2_), 2.87 (sep, *J* = 6.3 Hz, 1H, CH(CH_3_)_2_), 2.90 (t, *J* = 5.8 Hz, 2H, NCH_2_CH_2_), 3.34 (s, 2H, NCH_2_), 3.36–3.42 (m, 4H, N(CH_2_)_2_), 4.28 (s, 1H, CH(Ph)_2_), 7.15 (t,t, *J* = 7.3, 1.2 Hz, 2H, Ph), 7.22–7.29 (m, 4H, Ph), 7.39–7.44 (m, 4H, Ph). ^13^C NMR (75 MHz, DMSO-*d*_6_/CCl_4_, 1/3) δ 17.89, 29.06, 43.97, 48.52, 51.18, 53.15, 75.26, 100.41, 113.96, 119.84, 126.44, 127.17, 127.97, 141.93, 147.78, 150.64, 159.25. Anal. calcd. for C_29_H_32_ClN_5_: C 71.66; H 6.64; N 14.41%. Found: C 71.97; H 6.79; N 14.61%.

7-Benzyl-3-chloro-1-(4-methylpiperazin-1-yl)-5,6,7,8-tetrahydro-2,7-naphthyridine-4-carbonitrile (**2f**). Cream solid, yield 70%, m.p. 138–140 °C. IR *ν*/cm^−1^: 2215 (C≡N). ^1^H NMR (300 MHz, DMSO-*d*_6_/CCl_4_, 1/3) δ 2.23 (s, 3H, NCH_3_), 2.34–2.41 (m, 4H, CH_3_N(CH_2_)_2_), 2.74 (t, *J* = 6.0 Hz, 2H, NCH_2_CH_2_), 2.93 (t, *J* = 6.0 Hz, 2H, NCH_2_CH_2_), 3.27–3.33 (m, 4H, N(CH_2_)_2_), 3.34 (s, 2H, NCH_2_), 3.67 (s, 2H, NCH_2_Ph), 7.19–7.33 (m, 5H, Ph). ^13^C NMR (75 MHz, DMSO-*d*_6_/CCl_4_, 1/3) δ 28.35, 45.32, 48.06, 48.02, 52.59, 54.05, 61.26, 100.23, 113.92, 118.88, 126.66, 127.68, 128.27, 137.10, 147.98, 150.28, 159.18. Anal. calcd. for C_21_H_24_ClN_5_: C 66.04; H 6.33; N 18.34%. Found: C 65.71; H 6.51; N 18.12%.

7-Benzyl-3-chloro-1-(4-ethylpiperazin-1-yl)-5,6,7,8-tetrahydro-2,7-naphthyridine-4-carbonitrile (**2g**). Colorless solid, yield 83%, m.p. 123–125 °C. IR *ν*/cm^−1^: 2216 (C≡N). ^1^H NMR (300 MHz, DMSO-*d*_6_/CCl_4_, 1/3) δ 1.06 (t, *J* = 7.1 Hz, 3H, NCH_2_CH_3_), 2.32–2.46 (m, 6H, NCH_2_CH_3_, CH_3_N(CH_2_)_2_), 2.74 (t, *J* = 6.0 Hz, 2H, NCH_2_CH_2_), 2.93 (t, *J* = 5.9 Hz, 2H, NCH_2_CH_2_), 3.27–3.35 (m, 4H, N(CH_2_)_2_), 3.34 (s, 2H, NCH_2_), 3.67 (s, 2H, NCH_2_Ph), 7.19–7.32 (m, 5H, Ph). ^13^C NMR (75 MHz, DMSO-*d*_6_/CCl_4_, 1/3) δ 11.41, 28.36, 48.07, 48.13, 51.37, 51.81, 52.64, 61.27, 100.12, 113.97, 118.80, 126.67, 127.68, 128.29, 137.11, 148.00, 150.24, 159.13. Anal. calcd. for C_22_H_26_ClN_5_: C 66.74; H 6.62; N 17.69%. Found: C 67.09; H 6.78; N 17.92%.

7-Benzyl-3-chloro-1-(4-phenylpiperazin-1-yl)-5,6,7,8-tetrahydro-2,7-naphthyridine-4-carbonitrile (**2h**). Cream solid, yield 79%, m.p. 160–162 °C. IR *ν*/cm^−1^: 2219 (C≡N). ^1^H NMR (300 MHz, DMSO-*d*_6_/CCl_4_, 1/3) δ 2.76 (t, *J* = 5.8 Hz, 2H, NCH_2_CH_2_), 2.95 (t, *J* = 5.8 Hz, 2H, NCH_2_CH_2_), 3.17–3.24 (m, 4H, PhN(CH_2_)_2_), 3.43 (s, 2H, NCH_2_), 3.45–3.51 (m, 4H, N(CH_2_)_2_), 3.70 (s, 2H, NCH_2_Ph), 6.79 (t,t, *J* = 7.3, 1.0 Hz, 1H, Ph), 6.86–6.91 (m, 2H, Ph), 7.16–7.33 (m, 7H, Ph). ^13^C NMR (75 MHz, DMSO-*d*_6_/CCl_4_, 1/3) δ 28.38, 47.97, 48.16, 48.26, 52.50, 61.23, 100.56, 113.90, 115.49, 119.15, 126.71, 127.72, 128.31, 128.38, 137.11, 148.00, 150.33, 150.45, 159.14. Anal. calcd. for C_26_H_26_ClN_5_: C 70.34; H 5.90; N 15.77%. Found: C 69.96; H 6.04; N 15.97%.

7-Benzyl-3-chloro-1-[4-(diphenylmethyl)piperazin-1-yl]-5,6,7,8-tetrahydro-2,7-naphthyridine-4-carbonitrile (**2i**). Colorless solid, yield 74%, m.p. 170–172 °C. IR *ν*/cm^−1^: 2221 (C≡N). ^1^H NMR (300 MHz, DMSO-*d*_6_/CCl_4_, 1/3) δ 3.17–3.24 (m, 4H, Ph_2_N(CH_2_)_2_), 2.70 (t, *J* = 5.7 Hz, 2H, NCH_2_CH_2_), 2.91 (t, *J* = 5.8 Hz, 2H, NCH_2_CH_2_), 3.30 (s, 2H, NCH_2_), 3.32–3.38 (m, 4H, N(CH_2_)_2_), 3.62 (s, 2H, NCH_2_Ph), 4.23 (s, 1H, CH(Ph)_2_), 7.16 (t,t, *J* = 7.3, 1.1 Hz, 2H, Ph), 7.21–7.30 (m, 8H, Ph), 7.37–7.43 (m, 5H, Ph). Anal. calcd. for C_33_H_32_ClN_5_: C 74.21; H 6.04; N 13.11%. Found: C 74.55; H 6.21; N 13.32%.

Ethyl 4-(7-benzyl-3-chloro-4-cyano-5,6,7,8-tetrahydro-2,7-naphthyridin-1-yl)-piperazine-1-carboxylate (**2j**). Cream solid, yield 81%, m.p. 132–134 °C. IR *ν*/cm^−1^: 2221 (C≡N). ^1^H NMR (300 MHz, DMSO-*d*_6_/CCl_4_, 1/3) δ 1.26 (t, *J* = 7.1 Hz, 3H, CH_2_CH_3_), 2.76 (t, *J* = 5.8 Hz, 2H, NCH_2_CH_2_), 2.95 (t, *J* = 5.8 Hz, 2H, NCH_2_CH_2_), 3.26–3.31 (m, 4H, N(CH_2_)_2_), 3.38 (s, 2H, NCH_2_), 3.42–3.50 (m, 4H, N(CH_2_)_2_), 3.68 (s, 2H, NCH_2_Ph), 4.09 (q, *J* = 7.1 Hz, 2H, CH_2_CH_3_), 7.20–7.34 (m, 5H, Ph). ^13^C NMR (75 MHz, DMSO-*d*_6_/CCl_4_, 1/3) δ 14.21, 28.39, 42.82, 48.02, 48.12, 52.27, 60.41, 61.22, 101.05, 113.78, 119.51, 126.71, 127.71, 128.30, 137.11, 147.93, 150.69, 153.96, 159.23. Anal. calcd. for C_23_H_26_ClN_5_O_2_: C 62.79; H 5.96; N 15.92%. Found: C 62.42; H 6.11; N 15.69%.

#### 3.1.2. General Procedure for Synthesis of Compounds **3a**–**j**

A mixture of 1-amino-3-chloro-2,7-naphthyridine **1** (1 mmol) and hydrazine hydrate (501 mg, 10 mmol) in butanol (20 mL) was refluxed for 8 h. The butanol was distilled off to dryness, and water (50 mL) was added to the residue. The separated crystals were filtered off, washed with water, dried, and recrystallized from ethanol.

7-Isopropyl-5-(4-methylpiperazin-1-yl)-6,7,8,9-tetrahydro-3*H*-pyrazolo[3,4-*c*]-2,7-naphthyridin-1-amine (**3a**). Cream solid, yield 78%, m.p. 242–244 °C. IR *ν*/cm^−1^: 3050, 3178, 3213, 3440 (NH, NH_2_). ^1^H NMR (300 MHz, DMSO-*d*_6_/CCl_4_, 1/3) δ 1.11 (d, *J* = 6.4 Hz, 6H, CH(CH_3_)_2_), 2.28 (ս, 3H, NCH_3_), 2.46–2.53 (m, 4H, CH_3_N(CH_2_)_2_), 2.77 (t, *J* = 5.9 Hz, 2H, NCH_2_CH_2_), 2.85 (sep, *J* = 6.4 Hz, 1H, CH(CH_3_)_2_), 3.05–3.11 (m, 4H, N(CH_2_)_2_), 3.16 (t, *J* = 5.8 Hz, 2H, NCH_2_CH_2_), 3.49 (s, 2H, NCH_2_), 4.48 (s, 2H, NH_2_), 11.34 (s, 1H, NH). ^13^C NMR (75 MHz, DMSO-*d*_6_/CCl_4_, 1/3) δ 18.25, 26.84, 44.97, 45.55, 48.32, 49.43, 53.32, 54.70, 100.93, 114.51, 139.62, 147.15, 150.14, 159.59. Anal. calcd. for C_17_H_27_N_7_: C 61.98; H 8.26; N 29.76%. Found: C 61.66; H 8.44; N 30.01%. ESI HRMS [C_17_H_27_N_7_+H^+^] Calculated: 330.2406. Found: 330.2413.

5-(4-Ethylpiperazin-1-yl)-7-isopropyl-6,7,8,9-tetrahydro-3*H*-pyrazolo[3,4-*c*]-2,7-naphthyridin-1-amine (**3b**). Cream solid, yield 76%, m.p. 229–231 °C. IR *ν*/cm^−1^: 3050, 3174, 3213, 3440 (NH, NH_2_). ^1^H NMR (300 MHz, DMSO-*d*_6_/CCl_4_, 1/3) δ 1.10 (t, *J* = 7.3 Hz, 3H, NCH_2_CH_3_), 1.11 (d, *J* = 6.5 Hz, 6H, CH(CH_3_)_2_), 2.42 (q, *J* = 7.2 Hz, 2H, NCH_2_CH_3_), 2.50–2.57 (m, 4H, C_2_H_5_N(CH_2_)_2_), 2.76 (t, *J* = 5.9 Hz, 2H, NCH_2_CH_2_), 2.85 (sep, *J* = 6.5 Hz, 1H, CH(CH_3_)_2_), 3.05–3.11 (m, 4H, N(CH_2_)_2_), 3.16 (t, *J* = 5.9 Hz, 2H, NCH_2_CH_2_), 3.50 (s, 2H, NCH_2_), 4.48 (s, 2H, NH_2_), 11.37 (s, 1H, NH). ^13^C NMR (75 MHz, DMSO-*d*_6_/CCl_4_, 1/3) δ 11.65, 18.25, 26.84, 44.95, 48.36, 49.55, 51.63, 52.45, 53.32, 100.89, 114.51, 139.60, 147.17, 150.15, 159.60. Anal. calcd. for C_18_H_29_N_7_: C 62.94; H 8.51; N 28.55%. Found: C 63.29; H 8.70; N 28.33%. ESI HRMS [C_18_H_29_N_7_+H^+^] Calculated: 344.2563. Found: 344.2569.

7-Isopropyl-5-(4-phenylpiperazin-1-yl)-6,7,8,9-tetrahydro-3*H*-pyrazolo[3,4-*c*]-2,7-naphthyridin-1-amine (**3c**). Cream solid, yield 81%, m.p. 248–250 °C. IR *ν*/cm^−1^: 3023, 3090, 3165, 3210, 3438 (NH, NH_2_). ^1^H NMR (300 MHz, DMSO-*d*_6_/CCl_4_, 1/3) δ 1.12 (d, *J* = 6.5 Hz, 6H, CH(CH_3_)_2_), 2.79 (t, *J* = 5.9 Hz, 2H, NCH_2_CH_2_), 2.87 (sep, *J* = 6.5 Hz, 1H, CH(CH_3_)_2_), 3.19 (t, *J* = 5.8 Hz, 2H, NCH_2_CH_2_), 3.23–3.34 (m, 8H, C_4_H_8_N_2_), 3.57 (s, 2H, NCH_2_), 4.50 (s, 2H, NH_2_), 6.76 (t,t, *J* = 7.3, 1.0 Hz, 1H, Ph), 6.90–6.95 (m, 2H, Ph), 7.15–7.23 (m, 2H, Ph), 11.38 (s, 1H, NH). ^13^C NMR (75 MHz, DMSO-*d*_6_/CCl_4_, 1/3) δ 18.23, 26.84, 44.94, 48.24, 48.54, 49.69, 53.36, 101.14, 114.52, 115.28, 118.72, 128.30, 139.84, 147.19, 150.10, 150.74, 159.40. Anal. calcd. for C_22_H_29_N_7_: C 67.49; H 7.47; N 25.04%. Found: C 67.79; H 7.65; N 25.25%. ESI HRMS [C_22_H_29_N_7_ + H^+^] Calculated: 392.2563. Found: 392.2567.

5-[4-(Diphenylmethyl)piperazin-1-yl]-7-isopropyl-6,7,8,9-tetrahydro-3*H*-pyrazolo[3,4-*c*]-2,7-naphthyridin-1-amine (**3d**). Light yellow solid, yield 88%, m.p. 236–238 °C. IR *ν*/cm^−1^: 3208, 3342, 3410, 3486 (NH, NH_2_). ^1^H NMR (300 MHz, DMSO-*d*_6_/CCl_4_, 1/3) δ 1.09 (d, *J* = 6.5 Hz, 6H, CH(CH_3_)_2_), 2.48–2.56 (m, 4H, CHN(CH_2_)_2_), 2.75 (t, *J* = 5.8 Hz, 2H, NCH_2_CH_2_), 2.83 (sep, *J* = 6.5 Hz, 1H, CH(CH_3_)_2_), 3.09–3.18 (m, 6H, NCH_2_CH_2_, N(CH_2_)_2_), 3.46 (s, 2H, NCH_2_), 4.28 (s, 1H, CH(Ph)_2_), 4.47 (s, 2H, NH_2_), 7.14 (t,t, *J* = 7.3, 1.2 Hz, 2H, Ph), 7.22–7.29 (m, 4H, Ph), 7.40–7.45 (m, 4H, Ph), 11.30 (s, 1H, NH). ^13^C NMR (75 MHz, DMSO-*d*_6_/CCl_4_, 1/3) δ 18.20, 26.82, 44.87, 48.42, 49.66, 51.67, 53.33, 75.70, 100.87, 114.45, 126.30, 127.22, 127.93, 139.56, 142.40, 147.15, 150.11, 159.46. Anal. calcd. for C_29_H_35_N_7_: C 72.32; H 7.32; N 20.36%. Found: C 72.66; H 7.49; N 20.59%. ESI HRMS [C_29_H_35_N_7_ + H^+^] Calculated: 482.3032. Found: 482.3034.

Ethyl 4-(1-amino-7-isopropyl-6,7,8,9-tetrahydro-3*H*-pyrazolo-[3,4-*c*]-2,7-naphthyridin-5-yl)piperazine-1-carboxylate (**3e**). Cream solid, yield 85%, m.p. 226–228 °C. IR ν/cm^−1^: 1703 (C=O), 3087, 3145, 3309, 3378 (NH, NH_2_). ^1^H NMR (300 MHz, DMSO-*d*_6_/CCl_4_, 1/3) δ 1.11 (d, *J* = 6.5 Hz, 6H, CH(CH_3_)_2_), 1.27 (t, *J* = 7.1 Hz, 3H, CH_2_CH_3_), 2.77 (t, *J* = 5.6 Hz, 2H, NCH_2_CH_2_), 2.86 (sep, *J* = 6.5 Hz, 1H, CH(CH_3_)_2_), 3.03–3.12 (m, 4H, N(CH_2_)_2_), 3.17 (br t, *J* = 5.6 Hz, 2H, NCH_2_CH_2_), 3.53 (s, 2H, NCH_2_), 3.53–3.60 (m, 4H, N(CH_2_)_2_), 4.09 (q, *J* = 7.1 Hz, 2H, CH_2_CH_3_), 4.53 (s, 2H, NH_2_), 11.41 (s, 1H, NH). ^13^C NMR (75 MHz, DMSO-*d*_6_/CCl_4_, 1/3) δ 14.28, 18.21, 26.85, 43.34, 44.89, 48.16, 49.55, 53.36, 60.25, 101.29, 114.47, 140.06, 147.23, 150.01, 154.21, 159.25. Anal. calcd. for C_19_H_29_N_7_O_2_: C 58.89; H 7.54; N 25.30 %. Found: C 58.56; H 7.69; N 25.54%.

7-Benzyl-5-(4-methylpiperazin-1-yl)-6,7,8,9-tetrahydro-3*H*-pyrazolo[3,4-*c*]-2,7-naphthyridin-1-amine (**3f**). Cream solid, yield 83%, m.p. 244–246 °C. IR *ν*/cm^−1^: 3220, 3309, 3371 (NH, NH_2_). ^1^H NMR (300 MHz, DMSO-*d*_6_/CCl_4_, 1/3) δ 2.25 (s, 3H, NCH_3_), 2.38–2.45 (m, 4H, N(CH_2_)_2_), 2.72 (t, *J* = 6.0 Hz, 2H, NCH_2_CH_2_), 3.01–3.08 (m, 4H, N(CH_2_)_2_), 3.19 (t, *J* = 5.9 Hz, 2H, NCH_2_CH_2_), 3.45 (s, 2H, NCH_2_), 3.66 (s, 2H, NCH_2_Ph), 4.50 (s, 2H, NH_2_), 7.18–7.35 (m, 5H, Ph), 11.39 (s, 1H, NH). ^13^C NMR (75 MHz, DMSO-*d*_6_/CCl_4_, 1/3) δ 26.22, 45.48, 48.93, 49.25, 52.93, 54.54, 61.92, 100.88, 113.79, 126.44, 127.61, 128.42, 137.95, 139.4, 147.28, 150.22, 159.50. Anal. calcd. for C_21_H_27_N_7_: C 66.82; H 7.21; N 25.97%. Found: C 67.17; H 7.37; N 26.22%. ESI HRMS [C_21_H_27_N_7_ + H^+^] Calculated: 378.2406. Found: 378.2406.

7-Benzyl-5-(4-ethylpiperazin-1-yl)-6,7,8,9-tetrahydro-3*H*-pyrazolo[3,4-*c*]-2,7-naphthyridin-1-amine (**3g**). Colorless solid, yield 86%, m.p. 221–223 °C. IR ν/cm^−1^: 3031, 3203, 3353, 3444 (NH, NH_2_). ^1^H NMR (300 MHz, DMSO-*d*_6_/CCl_4_, 1/3) δ 1.07 (t, *J* = 7.2 Hz, 3H, NCH_2_CH_3_), 2.38 (q, *J* = 7.2 Hz, 2H, NCH_2_CH_3_), 2.41–2.48 (m, 4H, N(CH_2_)_2_), 2.72 (t, *J* = 5.9 Hz, 2H, NCH_2_CH_2_), 3.01–3.08 (m, 4H, N(CH_2_)_2_), 3.18 (t, *J* = 5.8 Hz, 2H, NCH_2_CH_2_), 3.45 (s, 2H, NCH_2_), 3.66 (s, 2H, NCH_2_Ph), 4.50 (s, 2H, NH_2_), 7.17–7.34 (m, 5H, Ph), 11.39 (s, 1H, NH). ^13^C NMR (75 MHz, DMSO-*d*_6_/CCl_4_, 1/3) δ 11.59, 26.22, 48.96, 49.42, 51.62, 52.30, 53.00, 61.95, 100.82, 113.79, 126.42, 127.59, 128.41, 137.96, 139.36, 147.26, 150.23, 159.52. Anal. calcd. for C_22_H_29_N_7_: C 67.49; H 7.47; N 25.04%. Found: C 67.12; H 7.65; N 25.27%. ESI HRMS [C_22_H_29_N_7_ + H^+^] Calculated: 392.2563. Found: 392.2567.

7-Benzyl-5-(4-phenylpiperazin-1-yl)-6,7,8,9-tetrahydro-3*H*-pyrazolo[3,4-*c*]-2,7-naphthyridin-1-amine (**3h**). Colorless solid, yield 82%, m.p. 227–229 °C. IR *ν*/cm^−1^: 3021, 3095, 3209, 3312, 3411 (NH, NH_2_). ^1^H NMR (300 MHz, DMSO-*d*_6_/CCl_4_, 1/3) δ 2.75 (t, *J* = 5.8 Hz, 2H, NCH_2_CH_2_), 3.18–3.26 (m, 10H, C_4_H_8_N_2_, NCH_2_CH_2_), 3.53 (s, 2H, NCH_2_), 3.68 (s, 2H, NCH_2_Ph), 4.52 (s, 2H, NH_2_), 6.76 (t,t, *J* = 7.3, 1.0 Hz, 1H, Ph), 6.87–6.92 (m, 2H, Ph), 7.15–7.36 (m, 7H, Ph), 11.41 (s, 1H, NH). ^13^C NMR (75 MHz, DMSO-*d*_6_/CCl_4_, 1/3) δ 26.19, 48.42, 48.86, 49.56, 52.83, 61.86, 101.05, 113.76, 115.26, 118.71, 126.45, 127.60, 128.34, 128.38, 137.671, 139.58, 147.26, 150.15, 150.73, 159.30. Anal. calcd. for C_26_H_29_N_7_: C 71.04; H 6.65; N 22.31%. Found: C 70.71; H 6.80; N 22.10%. ESI HRMS [C_26_H_29_N_7_ + H^+^] Calculated: 440.2563. Found: 440.2566.

7-Benzyl-5-[4-(diphenylmethyl)piperazin-1-yl]-6,7,8,9-tetrahydro-3*H*-pyrazolo[3,4-*c*]-2,7-naphthyridin-1-amine (**3i**). Cream solid, yield 87%, m.p. 217–219 °C. IR *ν*/cm^−1^: 3026, 3148, 3301, 3376 (NH, NH_2_). ^1^H NMR (300 MHz, DMSO-*d*_6_/CCl_4_, 1/3) δ 2.39–2.48 (m, 4H, N(CH_2_)_2_), 2.69 (t, *J* = 5.6 Hz, 2H, NCH_2_CH_2_), 3.06–3.13 (m, 4H, N(CH_2_)_2_), 3.18 (t, *J* = 5.6 Hz, 2H, NCH_2_CH_2_), 3.42 (s, 2H, NCH_2_), 3.62 (s, 2H, NCH_2_Ph), 4.25 (s, 1H, CH(Ph)_2_), 4.47 (s, 2H, NH_2_), 7.12–7.32 (m, 10H, Ph), 7.38–7.45 (m, 5H, Ph), 11.34 (s, 1H, NH). ^13^C NMR (75 MHz, DMSO-*d*_6_/CCl_4_, 1/3) δ 26.17, 48.87, 49.54, 51.50, 53.14, 61.91, 75.61, 100.80, 113.67, 126.29, 126.40, 127.29, 127.56, 127.89, 128.32, 137.92, 139.27, 142.23, 147.18, 150.18, 159.34. Anal. calcd. for C_33_H_35_N_7_: C 74.83; H 6.66; N 18.51%. Found: C 74.48; H 6.83; N 18.75%. ESI HRMS [C_33_H_35_N_7_ + H^+^] Calculated: 530.3032. Found: 530.3033.

Ethyl 4-(1-amino-7-benzyl-6,7,8,9-tetrahydro-3*H*-pyrazolo[3,4-*c*]-2,7-naphthyridin-5-yl)piperazine-1-carboxylate (**3j**). Cream solid, yield 79%, m.p. 220–222 °C. IR *ν*/cm^−1^: 1701 (C=O), 3091, 3147, 3314, 3383 (NH, NH_2_). ^1^H NMR (300 MHz, DMSO-*d*_6_/CCl_4_, 1/3) δ 1.27 (t, *J* = 7.1 Hz, 3H, CH_2_CH_3_), 2.75 (t, *J* = 5.8 Hz, 2H, NCH_2_CH_2_), 2.98–3.06 (m, 4H, N(CH_2_)_2_), 3.21 (t, *J* = 5.6 Hz, 2H, NCH_2_CH_2_), 3.42–3.50 (m, 6H, NCH_2_, N(CH_2_)_2_), 3.67 (s, 2H, NCH_2_Ph), 4.08 (q, *J* = 7.1 Hz, 2H, CH_2_CH_3_), 4.54 (s, 2H, NH_2_), 7.19–7.35 (m, 5H, Ph), 11.42 (s, 1H, NH). ^13^C NMR (75 MHz, DMSO-*d*_6_/CCl_4_, 1/3) δ 14.29, 26.17, 43.20, 48.96, 49.46, 52.62, 60.25, 61.87, 101.20, 113.66, 126.49, 127.62, 128.39, 1337.86, 19.74, 147.27, 150.05, 154.12, 159.13. Anal. calcd. for C_23_H_29_N_7_O_2_: C 63.43; H 6.71; N 22.51%. Found: C 63.07; H 6.90; N 22.73%.

#### 3.1.3. General Procedure for Synthesis of Compounds **4a**–**j**

A mixture of pyrazolo[3,4-*c*]-2,7-naphthyridine **2** (1 mmol) and acetylacetone (15 mL) was refluxed for 8 h. Then, the excess of acetylacetone was distilled off, and diethyl ether (50 mL) was added. The formed crystals were filtered off and recrystallized from ethanol.

3-Isopropyl-9,11-dimethyl-5-(4-methylpiperazin-1-yl)-1,2,3,4-tetrahydropyrimido[1′,2′:1,5]pyrazolo[3,4-*c*]-2,7-naphthyridine (**4a**). Light yellow solid, yield 71%, m.p. 221–223 °C. ^1^H NMR (300 MHz, DMSO-*d*_6_/CCl_4_, 1/3) δ 1.14 (d, *J* = 6.4 Hz, 6H, CH(CH_3_)_2_), 2.30 (s, 3H, NCH_3_), 2.52–2.58 (m, 4H, CH_3_N(CH_2_)_2_), 2.64 (s, 3H, CH_3_), 2.84 (s, 3H, CH_3_), 2.89 (t, *J* = 5.5 Hz, 2H, NCH_2_CH_2_), 2.88 (sep, *J* = 6.3 Hz, 1H, CH(CH_3_)_2_), 3.25–3.31 (m, 4H, N(CH_2_)_2_), 3.42 (t, *J* = 5.6 Hz, 2H, NCH_2_CH_2_), 3.59 (s, 2H, NCH_2_), 7.00 (s, 1H, 10-CH). ^13^C NMR (75 MHz, DMSO-*d*_6_/CCl_4_, 1/3) δ 17.01, 18.24, 23.89, 27.73, 44.74, 45.55, 48.73, 49.12, 53.37, 54.54, 99.69, 110.27, 117.10, 140.80, 142.23, 143.69, 154.61, 157.97, 162.38. Anal. calcd. for C_22_H_31_N_7_: C 67.15; H 7.94; N 24.91%. Found: C 67.49; H 8.12; N 25.13%. ESI HRMS [C_22_H_31_N_7_ + H^+^] Calculated: 394.2719. Found: 394.2721.

5-(4-Ethylpiperazin-1-yl)-3-isopropyl-9,11-dimethyl-1,2,3,4-tetrahydropyrimido-[1′,2′:1,5]pyrazolo[3,4-*c*]-2,7-naphthyridine (**4b**). Light yellow solid, yield 68%, m.p. 170–172 °C. ^1^H NMR (300 MHz, DMSO-*d*_6_/CCl_4_, 1/3) δ 1.12 (t, *J* = 7.2 Hz, 3H, NCH_2_CH_3_), 1.14 (d, *J* = 6.4, 6H, CH(CH_3_)_2_), 2.44 (q, *J* = 7.2 Hz, 2H, NCH_2_CH_3_), 2.56–2.61 (m, 4H, C_2_H_5_N(CH_2_)_2_), 2.64 (s, 3H, CH_3_), 2.85 (s, 3H, CH_3_), 2.87 (t, *J* = 5.7 Hz, 2H, NCH_2_CH_2_), 2.88 (sep, *J* = 6.2 Hz, 1H, CH(CH_3_)_2_), 3.25–3.30 (m, 4H, N(CH_2_)_2_), 3.42 (t, *J* = 5.6 Hz, 2H, NCH_2_CH_2_), 3.59 (s, 2H, NCH_2_), 6.99 (s, 1H, 10-CH). ^13^C NMR (75 MHz, DMSO-*d*_6_/CCl_4_, 1/3) δ 11.63, 17.00, 18.23, 23.87, 27.73, 44.72, 48.77, 49.23, 51.62, 52.32, 53.36, 99.66, 110.24, 117.09, 140.75, 142.22, 143.67, 154.58, 157.98, 162.35. Anal. calcd. for C_23_H_33_N_7_: C 67.78; H 8.16; N 24.06%. Found: C 67.42; H 8.32; N 23.82%.

3-Isopropyl-9,11-dimethyl-5-(4-phenylpiperazin-1-yl)-1,2,3,4-tetrahydropyrimido-[1′,2′:1,5]pyrazolo[3,4-*c*]-2,7-naphthyridine (**4c**). Light yellow solid, yield 73%, m.p. 189–191 °C. ^1^H NMR (300 MHz, DMSO-*d*_6_/CCl_4_, 1/3) δ 1.14 (d, *J* = 6.4 Hz, 6H, CH(CH_3_)_2_), 2.65 (s, 3H, CH_3_), 2.85 (s, 3H, CH_3_), 2.82–2.94 (m, 3H, NCH_2_CH_2_, CH(CH_3_)_2_), 3.33–3.49 (m, 10H, NCH_2_CH_2_, C_4_H_8_N_2_), 3.66 (s, 2H, NCH_2_), 6.78 (t,t, *J* = 7.3, 1.1 Hz, 1H, Ph), 6.91–6.99 (m, 2H, Ph), 7.03 (s, 1H, 10-CH), 7.15–7.24 (m, 2H, Ph). ^13^C NMR (75 MHz, DMSO-*d*_6_/CCl_4_, 1/3) δ 17.00, 18.15, 23.89, 27.65, 44.65, 48.45, 49.36, 53.48, 99.82, 104.56, 110.41, 115.33, 118.76, 128.28, 140.95, 142.24, 143.77, 150.73, 154.73, 157.91, 162.21. Anal. calcd. for C_27_H_33_N_7_: C 71.18; H 7.30; N 21.52%. Found: C 71.56; H 7.49; N 21.27%.

5-[4-(Diphenylmethyl)piperazin-1-yl]-3-isopropyl-9,11-dimethyl-1,2,3,4-tetrahydropyrimido[1′,2′:1,5]pyrazolo[3,4-*c*]-2,7-naphthyridine (**4d**). Cream solid, yield 67%, m.p. 143–144 °C. ^1^H NMR (300 MHz, DMSO-*d*_6_/CCl_4_, 1/3) δ 1.12 (d, *J* = 6.5 Hz, 6H, CH(CH_3_)_2_), 2.55–2.61 (m, 4H, N(CH_2_)_2_), 2.63 (s, 3H, CH_3_), 2.80–2.94 (m, 3H, NCH_2_CH_2_, CH(CH_3_)_2_), 2.84 (s, 3H, CH_3_), 3.30–3.36 (m, 4H, N(CH_2_)_2_), 3.41 (t, *J* = 5.5 Hz, 2H, NCH_2_CH_2_), 3.55 (s, 2H, NCH_2_), 4.30 (s, 1H, CH(Ph)_2_), 6.98 (s, 1H, 10-CH), 7.15 (t,t, *J* = 7.3, 1.2 Hz, 2H, Ph), 7.23–7.30 (m, 4H, Ph), 7.43–7.48 (m, 4H, Ph). ^13^C NMR (75 MHz, DMSO-*d*_6_/CCl_4_, 1/3) δ 17.02, 18.18, 23.89, 27.75, 44.64, 48.84, 49.41, 51.52, 53.40, 75.68, 99.65, 110.26, 126.32, 127.23, 127.93, 140.75, 142.23, 142.35, 143.70, 154.60, 157.97, 162.22. Anal. calcd. for C_34_H_39_N_7_: C 74.83; H 7.20; N 17.97%. Found: C 74.48; H 7.37; N 18.17%.

Ethyl 4-(3-isopropyl-9,11-dimethyl-1,2,3,4-tetrahydropyrimido[1′,2′:1,5]pyrazolo-[3,4-*c*]-2,7-naphthyridin-5-yl)piperazine-1-carboxylate (**4e**). Cream solid, yield 69%, m.p. 249–251 °C. ^1^H NMR (300 MHz, DMSO-*d*_6_/CCl_4_, 1/3) δ 1.29 (t, *J* = 7.1 Hz, 3H, CH_2_CH_3_), 1.51 (d, *J* = 6.3 Hz, 6H, CH(CH_3_)_2_), 2.67 (s, 3H, CH_3_), 2.83 (s, 3H, CH_3_), 3.17–3.49 (m, 5H, CH(CH_3_)_2_, N(CH_2_)_2_), 3.56–4.01 (m, 8H, NCH_2_CH_2_, N(CH_2_)_2_), 4.12 (q, *J* = 7.1 Hz, 2H, CH_2_CH_3_), 4.33 (s, 2H, NCH_2_), 7.15 (s, 1H, 10-CH). ^13^C NMR (75 MHz, DMSO-*d*_6_/CCl_4_, 1/3) δ 14.51, 46.48, 17.06, 24.16, 24.48, 43.17, 43.20, 43.26, 43.83, 46.16, 49.31, 56.79, 60.79, 110.83, 112.03, 139.55, 142.51, 145.23, 154.78, 157.30, 157.80, 161.96. Anal. calcd. for C_24_H_33_N_7_O_2_: C 63.84; H 7.37; N 21.71%. Found: C 64.17; H 7.22; 21.50%. ESI HRMS [C_24_H_33_N_7_O_2_ + H^+^] Calculated: 452.2774. Found: 452.2774.

3-Benzyl-9,11-dimethyl-5-(4-methylpiperazin-1-yl)-1,2,3,4-tetrahydropyrimido-[1′,2′:1,5]pyrazolo[3,4-*c*]-2,7-naphthyridine (**4f**). Yellow solid, yield 72%, m.p. 183–185 °C. ^1^H NMR (300 MHz, DMSO-*d*_6_/CCl_4_, 1/3) δ 2.27 (s, 3H, NCH_3_), 2.43–2.50 (m, 4H, N(CH_2_)_2_), 2.62 (s, 3H, CH_3_), 2.85 (s, 3H, CH_3_), 2.82 (t, *J* = 6.0 Hz, 2H, NCH_2_CH_2_), 3.21–3.28 (m, 4H, N(CH_2_)_2_), 3.45 (t, *J* = 5.9 Hz, 2H, NCH_2_CH_2_), 3.56 (s, 2H, NCH_2_), 3.71 (s, 2H, NCH_2_Ph), 7.02 (s, 1H, 10-CH), 7.19–7.36 (m, 5H, Ph). ^13^C NMR (75 MHz, DMSO-*d*_6_/CCl_4_, 1/3) δ 17.00, 23.86, 27.09, 45.38, 48.66, 48.85, 53.26, 54.30, 61.87, 99.55, 110.36, 116.31, 126.41, 127.57, 128.35, 137.80, 140.55, 142.21, 143.76, 154.76, 157.95, 162.14. Anal. calcd. for C_26_H_31_N_7_: C 70.72; H 7.08; N 22.20%. Found: C 70.35; H 6.89; N 21.97%. ESI HRMS [C_26_H_31_N_7_ + H^+^] Calculated: 442.2719. Found: 442.2722.

3-Benzyl-5-(4-ethylpiperazin-1-yl)-9,11-dimethyl-1,2,3,4-tetrahydropyrimido-[1′,2′:1,5]pyrazolo[3,4-*c*]-2,7-naphthyridine (**4g**). Yellow solid, yield 67%, m.p. 205–207 °C. ^1^H NMR (400 MHz, CDCl_3_) δ 1.15 (t, *J* = 7.2 Hz, 3H, NCH_2_CH_3_), 2.49 (q, *J* = 7.2 Hz, 2H, NCH_2_CH_3_), 2.56–2.60 (m, 4H, N(CH_2_)_2_), 2.66 (s, 3H, CH_3_), 2.90 (s, 3H, CH_3_), 2.91 (t, *J* = 6.1 Hz, 2H, NCH_2_CH_2_), 3.38–3.43 (m, 4H, N(CH_2_)_2_), 3.55 (t, *J* = 6.2 Hz, 2H, NCH_2_CH_2_), 3.66 (s, 2H, NCH_2_), 3.77 (s, 2H, NCH_2_Ph), 7.27 (s, 1H, 10-CH), 7.28–7.42 (m, 5H, Ph). ^13^C NMR (100 MHz, CDCl_3_) δ 12.07, 18.01, 24.73, 27.68, 49.50, 49.77, 52.55, 53.06, 54.23, 62.74, 100.35, 110.81, 117.17, 127.29, 128.41, 129.32, 138.26, 141.62, 143.50, 144.81, 156.04, 159.30, 163.53. Anal. calcd. for C_27_H_33_N_7_: C 71.18; H 7.30; N 21.52%. Found: C 70.83; H 7.47; N 21.28%.

3-Benzyl-9,11-dimethyl-5-(4-phenylpiperazin-1-yl)-1,2,3,4-tetrahydropyrimido-[1′,2′:1,5]pyrazolo[3,4-*c*]-2,7-naphthyridine (**4h**). Brown solid, yield 70%, m.p. 193–195 °C. ^1^H NMR (300 MHz, DMSO-*d*_6_/CCl_4_, 1/3) δ 2.63 (s, 3H, CH_3_), 2.85 (t, *J* = 5.9 Hz, 2H, NCH_2_CH_2_), 2.87 (s, 3H, CH_3_), 3.26–3.31 (m, 4H, PhN(CH_2_)_2_), 3.39–3.45 (m, 4H, N(CH_2_)_2_), 3.48 (t, *J* = 5.8 Hz, 2H, NCH_2_CH_2_), 3.64 (s, 2H, NCH_2_), 3.73 (s, 2H, NCH_2_Ph), 6.77 (t,t, *J* = 7.2, 1.0 Hz, 1H, Ph), 6.90–6.95 (m, 2H, Ph), 7.03 (s, 1H, 10-CH), 7.16–7.37 (m, 7H, Ph). ^13^C NMR (75 MHz, DMSO-*d*_6_/CCl_4_, 1/3) δ 17.00, 23.86, 27.09, 48.35, 48.63, 49.22, 53.20, 61.89, 99.74, 110.43, 115.31, 116.35, 118.75, 126.45, 127.58, 128.30, 128.34, 137.79, 140.75, 142.24, 143.80, 150.71, 154.80, 157.94, 162.07. Anal. calcd. for C_31_H_33_N_7_: C 73.93; H 6.60; N 19.47%. Found: C 73.57; H 6.78; N 19.70%. ESI HRMS [C_31_H_33_N_7_ + H^+^] Calculated: 504.2876. Found: 504.2877.

3-Benzyl-5-[4-(diphenylmethyl)piperazin-1-yl]-9,11-dimethyl-1,2,3,4-tetrahydropyrimido[1′,2′:1,5]pyrazolo[3,4-*c*]-2,7-naphthyridine (**4i**). Yellow solid, yield 68%, m.p. 288–290 °C. ^1^H NMR (300 MHz, DMSO-*d*_6_/CCl_4_, 1/3) δ 2.46–2.53 (m, 4H, N(CH_2_)_2_), 2.61 (s, 3H, CH_3_), 2.79 (t, *J* = 5.6 Hz, 2H, NCH_2_CH_2_), 2.85 (s, 3H, CH_3_), 3.25–3.33 (m, 4H, N(CH_2_)_2_), 3.43 (t, *J* = 5.6 Hz, 2H, NCH_2_CH_2_), 3.51 (s, 2H, NCH_2_), 3.66 (s, 2H, NCH_2_Ph), 4.27 (s, 1H, CH(Ph)_2_), 6.99 (s, 1H, 10-CH), 7.12–7.34 (m, 11H, Ph), 7.40–7.46 (m, 4H, Ph). ^13^C NMR (75 MHz, DMSO-*d*_6_/CCl_4_, 1/3) δ 17.01, 23.85, 27.10, 48.66, 49.28, 51.38, 53.50, 61.93, 75.59, 99.53, 110.26, 116.27, 126.32, 126.41, 127.27, 127.56, 127.89, 128.31, 137.82, 140.49, 142.20, 142.24, 143.75, 154.66, 158.02, 162.05. Anal. calcd. for C_38_H_39_N_7_: C 76.87; H 6.62; N 16.51%. Found: C 76.53; H 6.47; N 16.29%.

Ethyl 4-(3-benzyl-9,11-dimethyl-1,2,3,4-tetrahydropyrimido[1′,2′:1,5]pyrazolo-[3,4-*c*]-2,7-naphthyridin-5-yl)piperazine-1-carboxylate (**4j**). Colorless solid, yield 71%, m.p. 215–217 °C. ^1^H NMR (300 MHz, DMSO-*d*_6_/CCl_4_, 1/3) δ 1.28 (t, *J* = 7.1 Hz, 3H, CH_2_CH_3_), 2.63 (s, 3H, CH_3_), 2.86 (t, *J* = 5.8 Hz, 2H, NCH_2_CH_2_), 2.85 (s, 3H, CH_3_), 3.20–3.26 (m, 4H, N(CH_2_)_2_), 3.47 (t, *J* = 5.9 Hz, 2H, NCH_2_CH_2_), 3.50–3.56 (m, 4H, N(CH_2_)_2_), 3.57 (s, 2H, NCH_2_), 3.72 (s, 2H, NCH_2_Ph), 4.08 (q, *J* = 7.1 Hz, 2H, CH_2_CH_3_), 7.03 (s, 1H, 10-CH), 7.19–7.37 (m, 5H, Ph). ^13^C NMR (75 MHz, DMSO-*d*_6_/CCl_4_, 1/3) δ 14.26, 16.99, 23.86, 27.04, 43.07, 48.70, 49.13, 53.00, 60.22, 61.90, 99.85, 110.51, 116.22, 126.47, 127.59, 128.35, 137.77, 140.95, 142.23, 143.86, 154.11, 154.89, 157.81, 161.96. Anal. calcd. for C_28_H_33_N_7_O_2_: C 67.31; H 6.66; N 19.62%. Found: C 66.93; H 6.47; N 19.38%. ESI HRMS [C_28_H_33_N_7_O_2_ + H^+^] Calculated: 500.2774. Found: 500.2776.

#### 3.1.4. General Procedure for Synthesis of Compounds **6a**–**d**

To a stirred solution of sodium ethoxide (408 mg, 6 mmol) and 50 mL of hydroxylamine hydrochloride (347.4 mg, 5 mmol) in absolute ethanol, 1-amino-3-chloro-2,7-naphthyridine **5** (1 mmol) was added, and the reaction mixture was refluxed for 15 h, followed by the addition of water (50 mL) after completion of the reaction. The precipitate was filtered off, washed with water, dried, and recrystallized from ethanol.

7-Isopropyl-5-pyrrolidin-1-yl-6,7,8,9-tetrahydroisoxazolo[5,4-*c*]-2,7-naphthyridin-1-amine (**6a**). Cream solid, yield 63%, m.p. 202–204 °C. IR *ν*/cm^−1^: 3458, 3321, 3211 (NH_2_). ^1^H NMR (400 MHz, DMSO-*d*_6_/CCl_4_, 1/3) δ 1.09 (d, *J* = 6.5, 6H, CH(CH_3_)_2_), 1.92–1.98 (m, 4H, C_4_H_8_N), 2.71 (t, *J* = 5.8 Hz, 2H, NCH_2_CH_2_), 2.87 (sep, *J* = 6.5 Hz, 1H, CH(CH_3_)_2_), 3.08 (t, *J* = 5.9 Hz, 2H, NCH_2_CH_2_), 3.51–3.58 (m, 4H, N(CH_2_)_2_), 3.53 (s, 2H, NCH_2_), 5.35 (s, 2H, NH_2_). ^13^C NMR (100 MHz, DMSO-*d*_6_/CCl_4_, 1/3) δ 18.15, 25.06, 26.75, 44.23, 49.40, 49.68, 53.45, 96.95, 113.48, 141.37, 158.28, 158.60, 167.09. Anal. calcd. for C_16_H_23_N_5_O: C 63.76; H 7.69; N 23.24%. Found: C 63.4; H 7.55; N 23.04%. ESI HRMS [C_16_H_23_N_5_O + H^+^] Calculated: 302.1981. Found: 302.1997.

7-Isopropyl-5-piperidin-1-yl-6,7,8,9-tetrahydroisoxazolo[5,4-*c*]-2,7-naphthyridin-1-amine (**6b**). Colorless solid, yield 67%, m.p. 248–250 °C. IR *ν*/cm^−1^: 3443, 3311, 3205 (NH_2_). ^1^H NMR (400 MHz, DMSO-*d*_6_/CCl_4_, 1/3) δ 1.07 (d, *J* = 6.6, 6H, CH(CH_3_)_2_), 1.59–1.75 (m, 6H, C_5_H_10_N), 2.76 (t, *J* = 5.9 Hz, 2H, NCH_2_CH_2_), 2.84 (sep, *J* = 6.6 Hz, 1H, CH(CH_3_)_2_), 3.07–3.18 (m, 6H, NCH_2_CH_2_, N(CH_2_)_2_), 3.46 (s, 2H, NCH_2_), 5.50 (s, 2H, NH_2_). ^13^C NMR (100 MHz, DMSO-*d*_6_/CCl_4_, 1/3) δ 18.20, 24.02, 25.50, 26.54, 44.73, 48.48, 50.47, 53.30, 99.84, 117.48, 142.05, 158.29, 161.27, 166.98. Anal. calcd. for C_17_H_25_N_5_O: C 64.73; H 7.99; N 22.20%. Found: C 64.37; H 8.16; N 22.43%. ESI HRMS [C_17_H_25_N_5_O + H^+^] Calculated: 316.2137. Found: 316.2143.

7-Isopropyl-5-morpholin-4-yl-6,7,8,9-tetrahydroisoxazolo[5,4-*c*]-2,7-naphthyridin-1-amine (**6c**). Colorless solid, yield 62%, m.p. 256–258 °C. IR *ν*/cm^−1^: 3448, 3331, 3221 (NH_2_). ^1^H NMR (400 MHz, DMSO-*d*_6_/CCl_4_, 1/3) δ 1.08 (d, *J* = 6.7, 6H, CH(CH_3_)_2_), 2.76 (t, *J* = 5.9 Hz, 2H, NCH_2_CH_2_), 2.87 (sep, *J* = 6.6 Hz, 1H, CH(CH_3_)_2_), 3.09–3.19 (m, 6H, NCH_2_CH_2_, N(CH_2_)_2_), 3.48 (s, 2H, NCH_2_), 3.73–3.79 (m, 4H, O(CH_2_)_2_), 5.54 (s, 2H, NH_2_). ^13^C NMR (100 MHz, DMSO-*d*_6_/CCl_4_, 1/3) δ 18.13, 26.55, 44.44, 48.51, 49.76, 53.29, 65.93, 100.45, 117.41, 142.61, 158.30, 160.11, 166.82. Anal. calcd. for C_16_H_23_N_5_O_2_: C 60.55; H 7.30; N 22.07%. Found: C 60.92; H 7.48; N 22.32%.

7-Isobutyl-5-pyrrolidin-1-yl-6,7,8,9-tetrahydroisoxazolo[5,4-*c*]-2,7-naphthyridin-1-amine (**6d**). Colorless solid, yield 65%, m.p. 204–206 °C. IR *ν*/cm^−1^: 3468, 3293, 3188 (NH_2_). ^1^H NMR (400 MHz, DMSO-*d*_6_/CCl_4_, 1/3) δ 0.91 (d, *J =* 6.5 Hz, 6H, CH(CH_3_)_2_), 1.79–1.91 (m, 1H, CH(CH_3_)_2_), 1.89–1.99 (m, 4H, C_4_H_8_N), 2.24 (d, *J =* 7.4 Hz, 2H, CHCH_2_), 2.67 (t, *J =* 5.9 Hz, 2H, NCH_2_CH_2_), 3.11 (t, *J =* 5.8 Hz, 2H, NCH_2_CH_2_), 3.45 (s, 2H, NCH_2_), 3.48–3.54 (m, 4H, N(CH_2_)_2_), 5.37 (s, 2H, NH_2_). ^13^C NMR (100 MHz, DMSO-*d*_6_/CCl_4_, 1/3) δ 20.48, 25.04, 25.19, 26.17, 49.04, 49.66, 54.45, 66.02, 96.94, 112.85, 141.15, 158.28, 158.54, 167.14. Anal. calcd. for C_17_H_25_N_5_O: C 64.73; H 7.99; N 22.20%. Found: C 65.08; H 7.83; N 22.41%. ESI HRMS [C_17_H_25_N_5_O + H^+^] Calculated: 316.2137. Found: 316.2141.

### 3.2. Biological Evaluation: Experimental Part

Compounds’ possible neurotropic effects (anticonvulsant, sedative, anti-anxiety, and antidepressive) and adverse effects were examined in 50 male Wistar rats (weighing 120–140 g) and 450 white mice of both sexes (weighing 18–24 g). Every animal group was kept in the same room at 25 ± 2 °C and fed the same food. Reference substances were the sedative diazepam and the well-known antiepileptic medication ethosuximide [41,42]. The Litchfield and Wilcoxon statistical approach was applied to ascertain ED50, neurotoxic TD50, and LD50 [58]. The number of fatalities following 24 h exposure to dosages ranging from 100 to 1020 mg/kg was used to determine acute toxicity (LD50). Every biological experiment was carried out in strict adherence to the European Convention for the Protection of Vertebrate Animals. All animal procedures were performed in accordance with the Guidelines for the Care and Use of Laboratory Animals (ETS No123, Strasbourg, 18 March 1986).

#### 3.2.1. Evaluation of Anticonvulsant Potency

The following tests were used to examine the anticonvulsant impact of the newly synthesized compounds: pentylenetetrazole (PTZ)-induced convulsions, thiosemicarbazide-induced convulsions, and maximum electroshock (MES) [36,37,38,39,40]. For the investigation, outbred mice weighing 18–22 g were employed.

#### 3.2.2. PTZ-Induced Convulsions

PTZ was injected subcutaneously at a dose of 90 mg/kg, inducing convulsions in 95% of the animals (CD95%). Test substances were administered intraperitoneally (i.p.) at doses ranging from 10 to 200 mg/kg in a suspension of carboxymethylcellulose with Tween-80, 45 min before PTZ injection. Control animals received an emulsifier. Each dose of the test compounds was tested in six animals. Each animal was placed in an individual plastic cage for 1 h of observation. Seizures and clonic convulsions were recorded. 

#### 3.2.3. Thiosemicarbazide-Induced Convulsions

Thiosemicarbazide, an antimetabolite that inhibits GABA synthesis by targeting glutamic acid decarboxylase in the brain, was administered subcutaneously to mice at a dose of 18 mg/kg as a 0.5% solution, inducing clonic convulsions. The test compounds were administered intraperitoneally at a dose of 100 mg/kg in a suspension of carboxymethylcellulose with Tween-80, 45 min before thiosemicarbazide administration. The comparative drugs were administered as follows: ethosuximide at a dose of 200 mg/kg and diazepam at a dose of 2 mg/kg. The anticonvulsant activity of the test compounds was evaluated based on the latency time to the onset of seizures.

#### 3.2.4. MES Test

The following parameters were employed in the MES test, which is an animal model for generalized tonic seizures in epilepsy: 50 mA, 0.2 s, and an oscillation frequency of 50 impulses per second. By preventing the tonic-extensor phase of convulsions, the investigated drugs’ anticonvulsant qualities were evaluated.

#### 3.2.5. Examination of Psychotropic Effects of Synthesized Compounds

The psychotropic properties of the selected compounds were evaluated by using the following tests: the Open Field test [44,45], the Elevated Plus Maze (EPM) [46,47], the Forced Swimming test [48], and Rotating Rod Test to study muscle relaxation [43].

#### 3.2.6. Open Field Test

Research on motor behavior in rats was conducted by using a modified Open Field model. The setup featured an instrument with the floor divided into squares, each with perforated cells. Experiments were carried out during the daytime under natural light conditions. Each test group included 8 rats per compound, as well as control and reference drugs (diazepam and ethosuximide). The compounds were administered intraperitoneally at an effective dose of 50 mg/kg in a suspension of carboxymethylcellulose and Tween-80. Within 5 min of administration, indicators of both sedative and activating behavior were recorded, including the number of horizontal movements, instances of standing on the hind legs (vertical movements), and the sniffing of the cells.

#### 3.2.7. EPM Test

The Elevated Plus Maze test was used to evaluate the anti-anxiety and sedative effects of the tested compounds in mice. The maze consists of a cruciform apparatus elevated above the floor, with a pair of open arms and a pair of closed arms positioned opposite each other. The test compounds and the reference drug were administered intraperitoneally at doses of 50 mg/kg and 2 mg/kg, respectively, before the experiments. Control animals were given an emulsifier. The anxiolytic effect of the compounds was assessed by measuring the increase in the number of entries into the open (light) arms and the time spent in them, while ensuring that total motor activity did not increase. The sedative effect was evaluated based on the time spent in the closed (dark) arms and the number of attempts to enter the center of the maze.

#### 3.2.8. Forced Swimming Test

The Forced Swimming test was used to evaluate the “despair” and “depression” effects of the tested compounds. The test compounds and the reference drug were administered intraperitoneally at doses of 50 mg/kg and 2 mg/kg, respectively, prior to the experiments. Following injection, the experimental animals were placed in a glass container (22 cm in height and 14 cm in diameter) filled to a depth of one-third with water. The latency to immobility, the total duration of active swimming, and the time spent immobile were recorded during the 6 min period in which the animals were in the container. The experiments were conducted under natural light conditions.

#### 3.2.9. Evaluation of Coordination of Movement (Muscle Relaxant)

To evaluate the coordination of movement (muscle relaxant) in experimental animals, the Rotating Rod Test in mice was used. To this end, mice were placed on a metal rod with a corrugated rubber coating, which rotated at 5 revolutions per minute. The number of experimental mice (administered with the newly synthesized compounds at a dose of 50 mg/kg to 500 mg/kg) that were unable to stay on the rod for 2 min was recorded. The test determines the neurotoxic effects of compounds.

### 3.3. Docking Studies

For docking studies, AutoDock 4 (version 4.2.6) was used. The structures of the GABA_A_ receptor (PDB ID: 4COF), the SERT transporter (PDB ID: 3F3A), and the 5-HT_1A_ receptor (PDB ID: 3NYA) were obtained from the Protein Data Bank. The docking protocol followed was consistent with previously published studies [51,52,53,59].

All molecules were sketched in the chemdraw12.0 program. The geometry of the built compounds was optimized by using molecular mechanical force fields 94 (MMFF94) energy via the program LigandScout (ver. 4.4.5), and partial charges were also calculated; the conformers of each ligand were generated, and the one with the best conformation was maintained and saved as mol2 files, which were passed to ADT for file preparation. There, polar hydrogen was added to each structure, followed by computing Gasteiger and Kollman charges and the torsions. The region of interest, used by Autodock4 for docking runs and by Autogrid4 for affinity grid map preparation, was defined in such a way to comprise the whole catalytic binding site by using a grid size of 110 × 110 × 110 xyz points with a grid spacing of 0.375 Å. The grid centers were calculated to be at x = −20.558, y = −19.574, and z = 127.994 for the GABA_A_ receptor; at x = −19.7478, y = 22.417, and z = −14.3006 for the SERT transporter; and at x = −8.207, y = 9.305, and z = −48.61 for the 5-HT_1A_ receptor. For the simulation, default values of quaternation, translation, and torsion steps were applied. The Lamarckian Genetic Algorithm with default parameters was applied for minimization. The number of docking runs was 100. After docking, the 100 solutions were clustered into groups with RMS lower than 1.0 Ε. The clusters were ranked by the lowest energy representative of each cluster. Upon the completion of docking, the best poses were screened by examination of binding energy (Δ*G*_binding_, kcal/mol) and number in cluster. In order to describe the ligand–binding pocket interactions, the top-ranked binding mode found by AutoDock in complex with the binding pocket of the enzyme was selected. Accelrys Discovery Studio 2020 Client [60,61] and LigandScout (ver. 4.4.5) were used for the graphical representations of all ligand–protein complexes.

## 4. Conclusions

Starting from 1,3-dichloro-2,7-naphthyridines, a series of piperazino-substituted pyrazolo[3,4-*c*]-2,7-naphthyridines and pyrimido[1′,2′:1,5]pyrazolo[3,4-*c*]-2,7-naphthy ridines and new heterocyclic systems (isoxazolo[5,4-*c*]-2,7-naphthyridines) were synthesized. The investigation of their neurotropic activity showed that some of these compounds (**3a**,**b**,**d**,**f**–**i** and **4a**,**d**,**f**,**i**) display high anticonvulsant activity, especially in the test of antagonism with pentylenetetrazol, surpassing the well-known antiepileptic drug ethosuximide. Thus, the most active compounds in the pentylenpotetrazole test are **3h**, **3i**, and **4i,** with the ED50 of compound **4i** being 23.8 and the therapeutic index being more than 33.6, which is the highest among these three active compounds. On the other hand, they simultaneously exhibit psychotropic (anxiolytic, antidepressant, or sedative) or behavioral depressant effects. 

The effective compounds do not cause myorelaxation at the tested doses and have high therapeutic indices. Their therapeutic indexes are significantly higher than that of the reference drug, ethosuximide. At a dose of 100 mg/kg, these compounds increased the latency of thiosemicarbazide seizures by 1.1 to 2.0 times compared with the control. Compounds **3f** and **4a** showed a more pronounced (up to two–three times) anxiolytic effect compared with diazepam. Compared with the controls, the compounds increased the active swimming time, indicating antidepressant activity.

The most active compounds contained methyl and diphenylmethyl groups in the piperazine ring. Finally, the docking studies revealed that the most potent anticonvulsant compounds (**3i**, **4i**, and **4a**) showed high affinity for the GABAA and 5-HT1A receptors, as well as the SERT transporter. Thus, compound **4i** forms two hydrogen bonds with the residues Thr176 (N···H, 3.78 Å) and Arg180 (N···H, 3.26 Å), while diazepam only forms one hydrogen bond with Thr202 (N···H, 2.67) docked on the GABA_A_ receptor with the highest free binding energy (−8.81kcal/mol), comparable to that of diazepam (−8.90 kcal/mol). At the same time, compound 4i docked to the SERT transporter appeared to be the most promising with a free binding energy of −7.28, the highest among all compounds. The complex ligand compound–enzyme was stabilized by hydrogen bond formation with Gly439 and a hydrophobic interaction with Ile441, and there was an aromatic interaction between the benzene moiety and Arg11 in the SERT transporter. Furthermore, compound 4i docked to the orthosteric binding site of the 5-HT_1A_ receptor achieved the best docking score (−9.10 kcal/mol). This high docking score is due to two hydrogen bonds and plenty of hydrophobic interactions, which stabilized more the complex compound–enzyme. Thus, compound **4i** can be considered a dual-acting agent targeting the SERT transporter and the 5-HT_1A_ receptor.

## Figures and Tables

**Table 1:** Starting compounds: **1**, **2**, pyrazolo[3,4-*c*]-2,7-naphthyridines **3**, and pyrimido[1′,2′:1,5]pyrazolo[3,4-*c*]-2,7-naphthyridines **4**.

Compound	R	R^1^	Compound	R	R^1^
**1a**	*i*-Pr	–	**2e/3e/4e**	*i*-Pr	COOEt
**1b**	Bn	–	**2f/3f/4f**	Bn	Me
**1c**	*i*-Bu	–	**2g/3g/4g**	Bn	Et
**2a**/**3a**/**4a**	*i*-Pr	Me	**2h/3h/4h**	Bn	Ph
**2b**/**3b**/**4b**	*i*-Pr	Et	**2i/3i/4i**	Bn	CH(Ph)_2_
**2c**/**3c**/**4c**	*i*-Pr	Ph	**2j/3j/4j**	Bn	COOEt
**2d**/**3d**/**4d**	*i*-Pr	CH(Ph)_2_			

**Table 2:** Isoxazolo[5,4-*c*]-2,7-naphthyridines **6**.

Compound	R	NR^1^R^2^	Yield *, % (For Compounds 6)
**5a**/**6a**	*i*-Pr	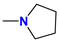	63
**5b**/**6b**	*i*-Pr	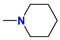	67
**5c**/**6c**	*i*-Pr	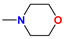	62
**5d**/**6d**	*i*-Bu	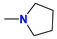	65

* Yields after recrystallization.

**Table 3:** Anticonvulsant activity and toxicity of the examined compounds of **3a**,**b**,**d**,**f**–**i** and **4a**,**d**,**f**,**i** and the reference drugs.

Compound	ED50 * mg/kg (by PTZ Antagonism)	TD50 * mg/kg	LD50 * mg/kg	TI	Latency of Convulsions Induced by TSC, min	
					M ± m	I**
Control	–	–	–	–	54.0 ± 2.5	1.0
**3a**	34.0 (29.6 ÷ 39.1)	>200	>500	>14.7	66.9 ± 7.1	1.24
**3b**	32.5 (27.1 ÷ 39.0)	>200	>500	>15.4	61.0 ± 3.5	1.13
**3d**	41.2 (35.2 ÷ 48.2)	>200	>700	>17.0	94.6 ± 9.2	1.75
**3f**	37.5 (28.8 ÷ 43.1)	>200	>600	>16.0	64.4 ± 6.9	1.20
**3g**	38.5 (32.1 ÷ 46.2)	>200	>700	>18.2	73.4 ± 7.2	1.40
**3h**	28.5 (23.9 ÷ 33.9)	>200	>600	>21.0	67.6 ± 8.7	1.25
**3i**	25.8 (21.5 ÷ 31.0)	>200	>600	>23.3	66.2 ± 7.9	1.23
**4a**	33.0 (27.5 ÷ 39.6)	>200	>600	>18.2	58.8 ± 5.8	1.10
**4d**	40.0 (32.0 ÷ 50.0)	>200	>600	>15.0	110.0 ± 11.5	2.00
**4f**	44.2 (36.8 ÷ 53.0)	>200	>700	>15.8	60.2 ± 5.2	1.15
**4i**	23.8 (19.8 ÷ 28.6)	>200	>800	>33.6	68.0 ± 6.1	1.26
**Ethosuximide** **(200 mg/kg)**	155 (117.5 ÷ 204.5)	520 (413 ÷ 655)	1325 (1200 ÷ 1462)	8.50	101.0 ± 14.0	1.87
**Diazepam** **(2 mg/kg)**	0.5 (0.4 ÷ 0.7)	2.7 (1.4 ÷ 5.5)	180 (128.5 ÷ 252.0)	360	55.0 ± 3.5	1.0

* *p* = 0.05 probability level; I**—an increase in the threshold.

**Table 4:** Effects of compounds **3a**,**b**,**d**,**f**–**i** and **4a**,**d**,**f**,**i** and reference drugs in rats in Open Field test.

Compound	Dose mg/kg	Amount (Absolute Data During 5 min) *		
		Horizontal Displacement	Vertical Displacement	Cells
Control	–	18.8 ± 3.2	4.2 ± 0.7	0.5 ± 0.2
**3a**	50	11.3 ± 2.8 **	2.8 ± 0.3 **	1.2 ± 0.2 **
**3b**	50	12.8 ± 1.6 **	2.4 ± 0.7 **	1.4 ± 0.3 **
**3d**	50	8.0 ± 1.1 **	1.6 ± 0.4 **	1.6 ± 0.5 **
**3f**	50	5.6 ± 1.4 **	0.6 ± 0.4 **	2.6 ± 0.2 **
**3g**	50	10.2 ± 2.2 **	1.2 ± 0.3 **	0.9 ± 0.1 **
**3h**	50	30.0 ± 4.1 **	3.2 ± 0.8	1.8 ± 0.1 **
**3i**	50	33.5 ± 4.2 **	6.4 ± 0.7 **	4.4 ± 1.1 **
**4a**	50	29.0 ± 3.1 **	3.4 ± 0.6	2.5 ± 0.5 **
**4d**	50	28.6 ± 2.8 **	3.1 ± 0.1 **	1.6 ± 0.3 **
**4f**	50	10.8 ± 3.3 **	2.3 ± 0.8 **	2.4 ± 0.6 **
**4i**	50	11.6 ± 1.3 **	2.1 ± 0.9 **	2.7 ± 0.5 **
**Ethosuximide**	200	23.8 ± 3.8	4.8 ± 0.9	0.6 ± 0.08
**Diazepam**	2	33.6 ± 4.2 **	6.4 ± 1.0 **	3.2 ± 0.9 **

* *p* ≤ 0.05 probability level; ** the differences are statistically significant compared with the control.

**Table 5:** Effects of compounds **3a**,**b**,**d**,**f**–**i** and **4a**,**d**,**f**,**i** on the state of “fear and despair” of mice in the EPM model (observation time 5 min).

Compound	Dose mg/kg	Time Spent in Closed Arms; /s/ *	Number of Entries into Closed Arms *	Time Spent in the Center; /s/ *	Time Spent in Open Arms; /s/ *
Control	−	278.2 ± 20.0	7.0 ± 1.2	21.8 ± 4.4	–
**3a**	50	243.0 ± 16.9 **	4.8 ± 1.3 **	15.7 ± 4.2	41.3 ± 13.0 **
**3b**	50	228.0 ± 15.9 **	3.8 ± 1.7 **	34.0 ± 7.2 **	38.0 ± 8.7 **
**3d**	50	241.0 ± 16.9 **	3.2 ± 0.7 **	30.0 ± 3.1	29.0 ± 12.0 **
**3f**	50	184.0 ± 21.6 **	2.6 ± 0.7 **	8.0 ± 1.2 **	108.0 ± 22.7 **
**3g**	50	230.0 ± 17.0 **	3.8 ± 0.9 **	26.0 ± 5.6	44.0 ± 8.1 **
**3h**	50	240.0 ± 11.9 **	3.9 ± 0.9 **	24.0 ± 5.1	36.0 ± 10.2 **
**3i**	50	225.0 ± 23.7 **	4.6 ± 0.8 **	40.0 ± 5.2 **	35.0 ± 6.6 **
**4a**	50	105.0 ± 15.0 **	4.8 ± 0.9 **	33.0 ± 5.7 **	162.0 ± 12.6 **
**4d**	50	248.0 ± 16.1 **	4.2 ± 1.0 **	20.0 ± 3.5 **	32.0 ± 7.7 **
**4f**	50	233.0 ± 21.0 **	4.7 ± 1.0 **	31.0 ± 5.7 **	36.0 ± 5.4 **
**4i**	50	238.2 ± 17.8 **	3.9 ± 1.1 **	38.0 ± 5.5 **	24.0 ± 5.5 **
**Ethosuximide**	200	245.2 ± 15.0	8.1 ± 2.5	54.8 ± 4.7 **	–
**Diazepam**	2	200.5 ± 15.2 **	5.5 ± 1.2	42.5 ± 3.9 **	57.0 ± 4.2 **

* *p* ≤ 0.05 probability level; ** the differences are statistically significant compared with the control.

**Table 6:** Effects of compounds **3a**,**b**,**d**,**f**–**i** and **4a**,**d**,**f**,**i** on the Forced Swimming test (observation time 6 min).

Compound	Dose mg/kg	Latent Period to I Immobilization (s) *	Total Time of Immobilization (s) *	Total Time of Active Swimming (s) *
**Control**	–	92.0 ± 7.8	81.0 ± 8.8	279.0 ± 23.3
**3a**	50	182.0 ± 15.3 **	35.5 ± 8.1 **	324.5 ± 20.1 **
**3b**	50	195.0 ± 15.2 **	17.5 ± 2.7	342.0 ± 25.3 **
**3d**	50	132.5± 9.7 **	57.5 ± 9.7 **	302.5 ± 20.1 **
**3f**	50	220.0 ± 18.6 **	12.5 ± 0.6 **	347.5 ± 21.0 **
**3g**	50	122.0 ± 15.3 **	74.5 ± 21.0 **	285.5 ± 23.0 **
**3h**	50	167.0 ± 11.8 **	28.3 ± 7.8 **	331.7 ± 21.0 **
**3i**	50	85.0 ± 12.0	117.5 ± 4.8 **	242.5 ± 18.8 **
**4a**	50	59.0 ± 10.6 **	178.0 ± 15.5 **	181.8 ± 19.9 **
**4d**	50	156.0 ± 15.8 **	16.7 ± 2.7 **	343.3 ± 29.7 **
**4f**	50	70.0 ± 8.6	20.0 ± 5.7	340.0 ± 27.7 **
**4i**	50	245.0 ± 26.4 **	101.0 ± 11.1 **	259.0 ± 28.0 **
**Ethosuximide**	200	105.0 ± 9.6	98.0 ± 9.9	262.0 ± 14.4
**Diazepam**	2	174.0 ± 18.1 **	24.0 ± 6.6 **	336.0 ± 18.9 **

* *p* ≤ 0.05 probability level; ** the differences are statistically significant compared with the control.

**Table 7:** Molecular docking to GABA_A_ receptor.

No.	Est. Binding Energy (kcal/mol)	I-H	Residues Involved in Hydrogen Bond Formation	Hydrophobic Interactions	Positive Ionizable Interactions
**3a**	−7.20	1	Thr202 (N···H, 3.18 Å),	Tyr62, Leu99, Phe200	Asp43
**3b**	−6.96	1	Tyr97 (N···H, 3.23 Å)	Thr176, Phe200, Tyr205	–
**3d**	−6.32	1	Thr202 (N···H, 3.75 Å)	Thr176, Phe200, Ala201	–
**3f**	−6.40	–	–	Thr176, Phe200	–
**3g**	−6.72	1	Tyr205 (N···H, 2.76 Å)	Tyr62, Thr176, Phe200	-
**3h**	−7.72	1	Thr202 (N···H, 3.54 Å)	Tyr62, Thr176, Ala201, Phe200	-
**3i**	−8.12	1	Thr176 (N···H, 3.20 Å)	Ala45, Tyr62, Ala201, Thr202	-
**4a**	−6.93	–	–	Tyr62, Thr176, Ala201, Tyr205	-
**4d**	−6.22	–	–	Ty157, Phe200, Tyr205	-
**4f**	−6.56	–	–	Ala201, Tyr205	-
**4i**	−8.81	2	Thr176 (N···H, 3.78 Å), Arg180 (N···H, 3.26 Å)	Ala45, Leu99, Val198, Phe200, Ala201, Thr202	Asp43
Diazepam	−8.90	1	Thr202 (N···H, 2.67 Å)	Tyr62, Leu99, Met115, Tyr157, Phe200, Tyr205	-

**Table 8:** Molecular docking in SERT transporter (PDB ID: 3F3A).

No.	Est. Binding Energy (kcal/mol)	I-H	Residues Involved in Hydrogen Bond Formation	Residues Involved in Hydrophobic Interactions	Residues Involved in Aromatic Interactions
**3a**	−4.86	-	-	-	-
**3b**	−7.16	1	Arg11 (N···H, 2.57 Å)	Asp267, Gly433	-
**3d**	−6.40	-	-	His7, Arg431	-
**3f**	−5.67	-	-	His7, Ile441	-
**3g**	−6.92	-	-	Asp267, Gly433	-
**3h**	−6.45	-	-	His7, Arg431	-
**3i**	−7.48	1	His7 (N···H, 3.25 Å)	Asp267, Gly433	Lys264
**4a**	−5.58	-	-	Asp267, Arg431	-
**4d**	−5.29	-	-	Asp267, Gly432	-
**4f**	−6.26	-	-	Gly433	-
**4i**	−7.28	1	Gly439 (N···H, 2.54 Å)	Ile441	Arg11

**Table 9:** Molecular docking free binding energy (kcal/mol) for 5-HT_1A_ receptor (PDB ID: 3NYA).

No.	Est. Binding Energy (kcal/mol)	Residues Involved in Hydrogen Bond Formation	Residues Involved in Hydrophobic Interactions	Residues Involved in Positive Ionizable Interactions
**3a**	−5.80	-	Thr195, Phe193, Val114, The290	-
**3b**	−8.82	Tyr316 (N···H, 3.76 Å)	Val114, Phe193, Ala200, Phe289, Tyr316, Phe290	N-Asp113
**3d**	−6.92	-	Ala200, Ile309, Asn312	-
**3f**	−5.08	-	Trp286, Asn312	-
**3g**	−5.27	-	Phe193, Trp313, Phe290	-
**3h**	−6.17	-	Trp109, Val117, Phe193, Thr195, Trp286	-
**3i**	−7.08	Tyr316 (N···H, 3.28 Å)	Trp109, Tyr308, Thr195, Ala200, Asn312	-
**4a**	−8.25	Tyr118 (N···H, 2.74 Å), Tyr316 (N···H, 3.59 Å)	Trp109, Tyr308, Phe193, Val114, Tyr110, Ile201, Ala200	-
**4d**	−4.30	-	Ile309, Thr195, Phe193, Trp313	-
**4f**	−7.12	Tyr316 (N···H, 2.58 Å)	Tyr316, Trp286, Asn312, Ala200	-
**4i**	−9.10	Tyr316 (N···H, 3.04 Å), Tyr316 (N···H, 2.57 Å)	Trp109, Trp313, Ile309, Thr195, Phe193, Val114, The290, Tyr308, Val117, Tyr199, Ala200	N-Asp113
Alprenolol	−13.19	Asp113, Asn312, Tyr316	Val117, Val114, Thr195, Phe193, Tyr199, Phe290	N-Asp113

## Data Availability

Data are contained within the article and Supplementary Materials.

## Supplementary Materials

The following supporting information can be downloaded at: https://www.mdpi.com/article/10.3390/ph18040597/s1.

## References

[1] WHO (2019). Epilepsy: A Public Health Imperative. Summary.

[2] Riney K., Bogacz A., Somerville E., Hirsch E., Nabbout R., Scheffer I.E., Zuberi S.M., Alsaadi T., Jain S., French J. (2022). International League Against Epilepsy classification and definition of epilepsy syndromes with onset at a variable age: Position statement by the ILAE Task Force on Nosology and Definitions. Epilepsia.

[3] Wirrell E.C., Tinuper P., Perucca E., Moshe S.L. (2022). Introduction to the epilepsy syndrome papers. Epilepsia.

[4] Saadi A., Patenaude B., Mateen F.J. (2016). Quality of life in epilepsy—31 inventory (QOLIE-31) scores: A global comparison. Epilepsy Behav..

[5] Basir N.H., Ramle A.Q., Ng M.P., Tan C.H., Tiekink E.R.T., Sim K.S., Basirun W.J., Khairuddean M. (2024). Discovery of indoleninylpyrazolo[3,4-*b*]pyridines as potent chemotherapeutic agents against colorectal cancer cells. Bioorg. Chem..

[6] Salem M.S., Ali M.A.M. (2016). Novel Pyrazolo[3,4-*b*]pyridine Derivatives: Synthesis, Characterization, Antimicrobial and Antiproliferative Profile. Biol. Pharm. Bull..

[7] El-Borai M.A., Rizk H.F., Abd-Aal M.F., El-Deeb I.Y. (2012). Synthesis of pyrazolo[3,4-*b*]pyridines under microwave irradiation in multi-component reactions and their antitumor and antimicrobial activities—Part 1. Eur. J. Med. Chem..

[8] Variya H.H., Panchal V., Patel G.R. (2019). Synthesis and characterization of 4-((5-bromo-1*H*-pyrazolo[3,4-*b*]pyridin-3-yl)amino)-*N*-(substituted)benzenesulfonamide as antibacterial, and antioxidant candidates. Curr. Chem. Lett..

[9] Elsherif M.A. (2021). Antibacterial evaluation and molecular properties of pyrazolo[3,4-*b*]pyridines and thieno[2,3-*b*]pyridines. J. Appl. Pharm. Sci..

[10] Abeed A.A., El-Emary T.I., Youssef M.S., Hefzy I., Ibrahim A.M. (2023). Synthesis and reactions of fused pyrazolo[3,4-*b*]pyridine derivatives: Insecticidal activity and digestive dysfunction against Mosquito Larvae. Curr. Org. Chem..

[11] Sun Y., Tang H., Wang X., Feng F., Fan T., Zhao D., Xiong B., Xie H., Liu T. (2022). Identification of 1*H*-pyrazolo[3,4-*b*]pyridine derivatives as novel and potent TBK1 inhibitors: Design, synthesis, biological evaluation, and molecular docking study. J. Enzyme Inhib. Med. Chem..

[12] Bharate S.B., Mahajan T.R., Gole Y.R., Nambiar M., Matan T.T., Kulkarni-Almeida A., Balachandran S., Junjappa H., Balakrishnan A., Vishwakarma R.A. (2008). Synthesis and evaluation of pyrazolo[3,4-*b*]pyridines and its structural analogues as TNF-α and IL-6 inhibitors. Bioorg. Med. Chem..

[13] Liu N., Wang X., Fu Q., Qin Q., Wu T., Lv R., Zhao D., Cheng M. (2023). Design, synthesis and biological evaluation of pyrazolo[3,4-*b*]pyridine derivatives as TRK inhibitors. RSC Med. Chem..

[14] Donaire-Arias A., Montagut A.M., Bellacasa R.P., Estrada-Tejedor R., Teixidó J., Borrell J.I. (2022). 1*H*-Pyrazolo[3,4-*b*]pyridines: Synthesis and Biomedical Applications. Molecules.

[15] Dodiya D.K., Trivedi A.R., Kataria V.B., Shah V.H. (2012). Advances in the synthesis of pyrazolo[3,4-*b*]pyridines. Curr. Org. Chem..

[16] Smolobochkin A.V., Gazizov A.S., Garifzyanov A.R., Burilov A.R., Pudovik M.A. (2022). Methods for the synthesis of 1*H*-pyrazolo[3,4-*b*]pyridine derivatives. Russ. Chem. Bull..

[17] Sirakanyan S.N., Spinelli D., Geronikaki A., Hovakimyan A.A. (2017). New methods for the synthesis of 3-amino-6,7-dihydro-5H-cyclopenta[c]pyridine-4-carbonitriles and cyclopenta[d]pyrazolo[3,4-*b*]pyridines via a Smiles-type rearrangement. J. Heterocycl. Chem..

[18] Becerra D., Abonia R., Juan-Carlos C. (2022). Recent Applications of the Multicomponent Synthesis for Bioactive Pyrazole Derivatives. Molecules.

[19] Becerra D., Juan-Carlos C. (2025). Recent advances in the synthesis of anticancer pyrazole derivatives using microwave, ultrasound, and mechanochemical techniques. RSC Adv..

[20] Wójcicka A. (2021). Synthesis and Biological Activity of 2,7-Naphthyridine Derivatives: An Overview. Curr. Org. Chem..

[21] Guo C., Guzzo P.R., Hadden M., Sargent B.J., Yet L., Kan Y., Palyha O., Kelly T.M., Guan X., Rosko K. (2010). Synthesis of 7-benzyl-5-(piperidin-1-yl)-6,7,8,9-tetrahydro-3*H*-pyrazolo[3,4-*c*][3,8]-naphthyridin-1-ylamine and its analogs as Bombesin receptor subtype-3 agonists. Bioorg. Med. Chem. Lett..

[22] Gabos B., Lundkvist M., Af Rosenschold M.M., Shamovsky I., Zlatoidsky P. (2010). Novel Hydantoin Derivatives as Metalloproteinase Inhibitors. U.S. Patent.

[23] Ukita T., Nakamura Y., Kubo A., Yamamoto Y., Moritani Y., Saruta K., Higashijima T., Kotera J., Fujishige K., Takagi M. (2003). 1,7- and 2,7-naphthyridine derivatives as potent and highly specific PDE5 inhibitors. Bioorganic Med. Chem. Lett..

[24] Sirakanyan S.N., Tonoyants N.A., Noravyan A.S., Dzhagatspanyan I.A., Nazaryan I.M., Akopyan A.G., Paronikyan R.G., Minasyan N.S. (2014). Synthesis and neurotropic activity of triazolo[3,4-*a*]- and triazolo[5,1-*a*]-2,7-naphthyridines. Pharm. Chem. J..

[25] Sirakanyan S.N., Hakobyan E.K., Nikoghosyan A.G., Paronikyan R.G., Dzhagatspanyan I.A., Nazaryan I.M., Akopyan A.G., Hovakimyan A.A. (2018). Synthesis and neurotropic activity of new 7-cyclohexyl-6,7,8,9-tetrahydro-3*H*-pyrazolo[3,4-*c*]-2,7-naphthyridine-1,5-diamines. Pharm. Chem. J..

[26] Dyadyuchenko L.V., Dotsenko V.V. (2021). Synthesis of isoxazolo[5,4-*b*]pyridine derivatives (microreview). Chem. Heterocycl. Compd..

[27] Poreba K., Wietrzyk J., Opolski A. (2003). Synthesis and antiproliferative activity in vitro of new 3-substituted aminoisoxazolo[5,4-*b*]pyridines. Acta Pol. Pharm. Drug Res..

[28] Astolfi A., Kudolo M., Brea J., Manni G., Manfroni G., Palazzotti D., Sabatini S., Cecchetti F., Felicetti T., Cannalire R. (2019). Discovery of potent p38a MAPK inhibitors through a funnel like workflow combining in silico screening and in vitro validation. Eur. J. Med. Chem..

[29] Chikkula K.V., Raja S. (2017). Isoxazole—A Potent Pharmacophore. Int. J. Pharm. Pharm. Sci..

[30] Sirakanyan S.N., Spinelli D., Geronikaki A., Hakobyan E.K., Kartsev V.G., Yegoryan H.A., Paronikyan E.G., Ghazaryan S.G., Hovakimyan A.A. (2022). Synthesis of new pentaheterocyclic systems based on the triazolo[3,4-*a*]-2,7-naphthyridine core. Arkivoc.

[31] Sirakanyan S.N., Hakobyan E.K., Spinelli D., Geronikaki A., Kartsev V.G., Yegoryan H.A., Hovakimyan A.A. (2022). Synthesis of new heterocyclic systems fused at pyrazolo[3,4-*c*]-2,7-naphthyridine core. Mendeleev Commun..

[32] Sirakanyan S.N., Spinelli D., Geronikaki A., Hovakimyan A.A., Noravyan A.S. (2014). New heterocyclic systems derived from pyridine: New substrates for the investigation of the azide/tetrazole equilibrium. Tetrahedron.

[33] Sirakanyan S.N., Spinelli D., Mattioli E.J., Calvaresi M., Geronikaki A., Kartsev V.G., Hakobyan E.K., Yegoryan H.A., Jughetsyan H.V., Manukyan M.E. (2024). Synthesis and Rearrangement of New 1,3-Diamino-2,7-naphthyridines and 1-Amino-3-oxo-2,7-naphthyridines. Int. J. Mol. Sci..

[34] Yang P., Yang Y., Zhang C., Yang X.-J., Wu B. (2009). Isolation of the half-condensed pyrazoline intermediates en route to N-(1,10-phenanthroline)-pyrazole derivatives from the Knorr reaction. Synth. Commun..

[35] El-Enany M.M., El-Meligie S.E.M., Abdou N.A., El-Nassan H.B. (2010). Synthesis of pyrazolo[3,4-*b*]pyridine and pyrido[2′,3′:3,4]pyrazolo[1,5-*a*]pyrimidine derivatives. J. Chem. Res..

[36] Vogel H.G., Vogel V.H., Vogel H.E. (2008). Psychotropic and Neurotropic Activity. Drug Discovery and Evaluation: Pharmacological Assays.

[37] Swinyard E.A., Purpura D.P., Penry J.K., Tower D., Woodbury D.M., Walter R. (1992). Experimental Models of Epilepsy.

[38] Katzung B.G. (2003). Drugs Used in Generalized Seizures. Basic and Clinical Pharmacology.

[39] Yuen E.S.M., Troconiz I.F. (2015). Can pentylenetetrazole and maximal electroshock rodent seizure models quantitatively predict antiepileptic efficacy in humans?. Seizure.

[40] Mironov A.H. (2012). The 1th Part. Manual for Preclinical Studies of Drugs.

[41] Löscher W., Klein P. (2021). The Pharmacology and Clinical Efficacy of Antiseizure Medications: From Bromide Salts to Cenobamate and Beyond. CNS Drugs.

[42] Zhang M., Kou L., Qin Y., Chen J., Bai D., Zhao L., Lin H., Jiang G. (2022). A bibliometric analysis of the recent advances in diazepam from 2012 to 2021. Front. Pharmacol..

[43] Lubrich C., Giesler P., Kipp M. (2022). Motor behavioral deficits in the cuprizone model: Validity of the rotarod test paradigm. Int. J. Mol. Sci..

[44] File S.E. (2001). Factors controlling measures of anxiety and responses to novelty in the mouse. Behav. Brain Res..

[45] Prut L., Belzung C. (2003). The open field as a paradigm to measure the effects of drugs on anxiety-like behaviors: A review. Eur. J. Pharmacol..

[46] Pellow S., Chopin P., File S.E., Briley M. (1985). Validation of open: Closed arm entries in an elevated plus-maze as a measure of anxiety in the rat. J. Neurosci. Methods.

[47] Graeff F.G., Netto C.F., Zangrossi H. (1998). The elevated T-maze as an experimental model of anxiety. Neurosci. Biobehav. Rev..

[48] Petit-Demouliere B., Chenu F., Bourin M. (2005). Forced swimming test in mice: A review of antidepressant activity. Psychopharmacology.

[49] Rogawski M.A., Löscher W. (2004). The neurobiology of antiepileptic drugs. Nat. Rev. Neurosci..

[50] Kammerer M., Rassner M.P., Freiman T.M., Feuerstein T.J. (2011). Effects of antiepileptic drugs on GABA release from rat and human neocortical synaptosomes. Naunyn-Schmiedeberg’s Arch. Pharmacol..

[51] Miller P.S., Aricescu A.R. (2014). Crystal structure of a human GABAA receptor. Nature.

[52] Saier M.H. (2000). A functional-phylogenetic classification system for transmembrane solute transporters. Microbiol. Mol. Biol. Rev..

[53] Perrone R., Berardi F., Colabufo N.A., Lacivita E., Larizza C., Leopoldo M., Tortorella V. (2005). Design and synthesis of long-chain arylpiperazines with mixed affinity for serotonin transporter (SERT) and 5-HT(1A) receptor. J. Pharm. Pharmacol..

[54] Blier P. (2001). Pharmacology of rapid-onset antidepressant treatment strategies. J. Clin. Psychiatry.

[55] Singh S.K., Piscitelli C.L., Yamashita A., Gouaux E. (2008). A competitive inhibitor traps LeuT in an opento-out conformation. Science.

[56] Kuipers W., Link R., Standaar P.J., Stoit A.R., Van Wijngaarden I., Leurs R., Ijzerman A.P. (1997). Study of the interaction between aryloxypropanolamines and Asn386 in helix VII of the human 5hydroxytryptamine1A receptor. Mol. Pharmacol..

[57] Wacker D., Fenalti G., Brown M.A., Katritch V., Abagyan R., Cherezov V., Stevens R.C. (2010). Conserved binding mode of human beta2 adrenergic receptor inverse agonists and antagonist revealed by X-ray crystallography. J. Am. Chem. Soc..

[58] Litchfield J.T., Wilcoxon F. (1949). A simplified method of evaluating dose-effect experiments. J. Pharmacol. Exp. Ther..

[59] Sirakanyan S.N., Spinelli D., Geronikaki A., Hakobyan E.K., Petrou A., Kartsev V., Yegoryan H.A., Paronikyan E.G., Zuppiroli L., Jughetsyan H.V. (2024). New triazole-based hybrids as neurotropic agents. RSC Adv..

[60] Bandaru S., Tiwari G., Akka J., Marri V.K., Alvala M., Gutlapalli V.R., Nayarisseri A., Mundluru H.P. (2015). Identification of high affinity bioactive Salbutamol conformer directed against mutated (Thr164Ile) beta 2 adrenergic receptor. Curr. Top. Med. Chem..

[61] (2005). Accelrys Discovery Studio® Visualizer.

