# Supplementary material for: Synthesis and Neurotropic Activity of New 5-Piperazinopyrazolo[3,4-c]-2,7-naphthyridines and Isoxazolo[5,4-c]-2,7-naphthyridines
Source: Pharmaceuticals (Basel). 2025 Apr 19;18(4):597. doi: 10.3390/ph18040597 (PMC12030261; doi:10.3390/ph18040597)
Supplement: Supplementary file 1 [file pharmaceuticals-18-00597-s001.zip › pharmaceuticals-3577244-supplementary.pdf]

Supplementary Data

Synthesis and Neurotropic Activity of New 5-Piperazinopyrazolo[3,4-*c*]-2,7-naphthyridines and Isoxazolo[5,4-*c*]-2,7-naphthyridines

Samvel N. Sirakanyan[1], Elmira K. Hakobyan[1,*], Athina Geronikaki[2,*], Domenico Spinelli[3,†], Anthi Petrou[2], Victor G. Kartsev [4], Hasmik A. Yegoryan[1], Hasmik V. Jughetsyan[1], Mariam E. Manukyan[1], Ruzanna G. Paronikyan[1], Tatevik A. Araqelyan[1], and Anush A. Hovakimyan[1]

1 Scientific Technological Center of Organic and Pharmaceutical Chemistry of National Academy of Science of Republic of Armenia, Institute of Fine Organic Chemistry of A.L.Mnjoyan, Armenia 0014, Yerevan; shnnr@mail.ru

2 Department of Pharmacy, School of Health, Aristotle University of Thessaloniki, 54124 Thessaloniki, Greece; geronik@pharm.auth.gr

3 Dipartimento di Chimica G. Ciamician, Alma Mater Studiorum-Università di Bologna, Via F. Selmi 2, Bologna 40126, Italy

4 InterBioScreen, Moscow 119019, Russia; vkartsev@ibscreen.chg.ru

† dead. We dedicate this paper to our good friend and collaborator Prof. Spinelli, who passed away on March 21, 2025.

* Correspondence: hakobyan.elmira@mail.ru (H.E.K.); geronik@pharm.auth.gr (A.G.)

^{1}H, ^{13}C NMR and MS spectra for all new synthesized compounds

2a

Molecular Structure Research Centre, Yerevan, Armenia, Varian Mercury-300VX

H1 300.088 MHz, nt = 16, np = 32000, temp = 30.0 C, lb = -0.2, solvent = DMSO/CCl4 1/3

Jan 25 2022

T21-078

ANUSH_TEMA t21-078

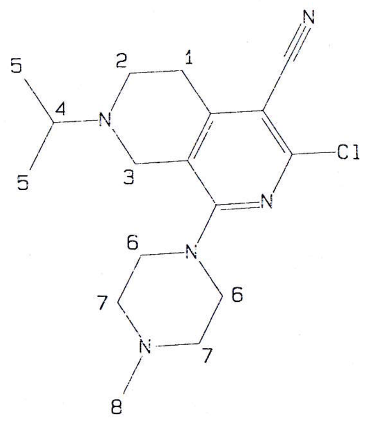

$C_{17}H_{24}ClN_5$

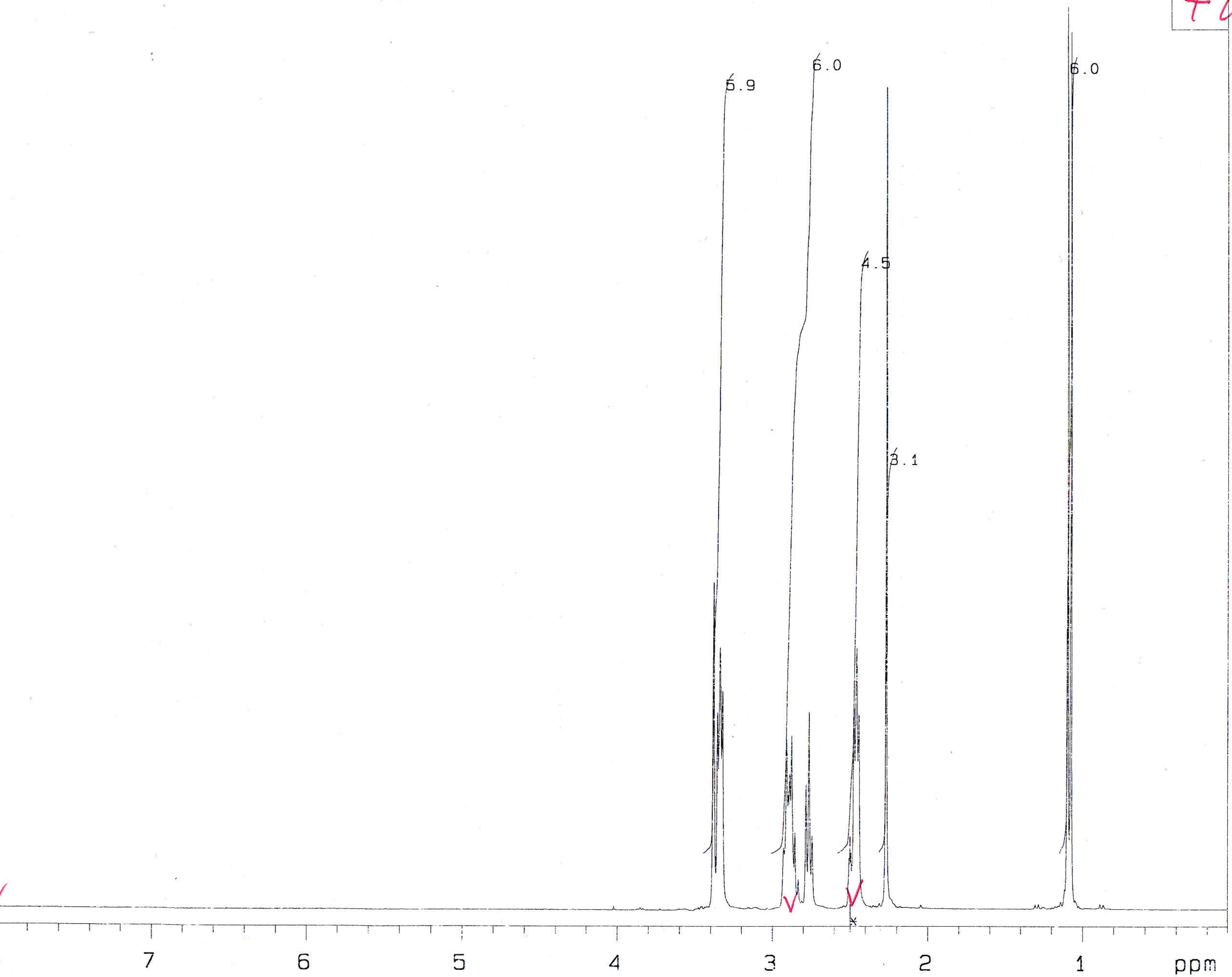

2a

Molecular Structure Research Centre, Yerevan, Armenia, Varian Mercury-300VX

C13 75.465 MHz, nt = 528, np = 19998, temp = 30.0 C, lb = 1.0, solvent = DMSO/CCl4 1/3

Jan 25 2022

ANUSH_TEMA t21-078

T21-078

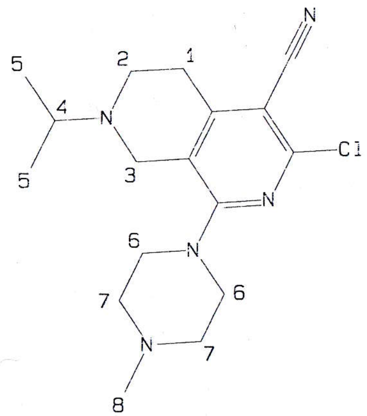

$C_{17}H_{24}ClN_5$

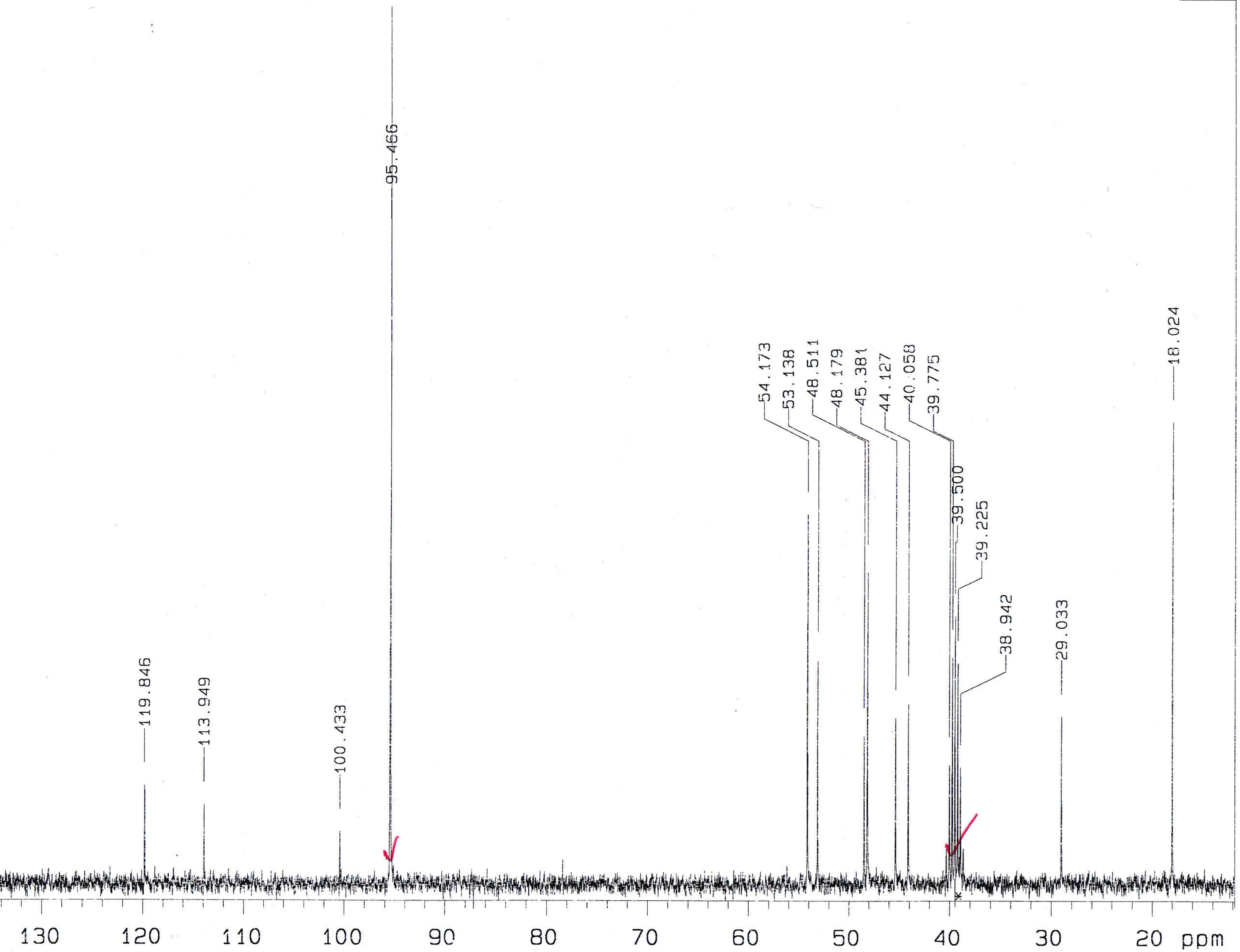

+ [illegible]

2b

Molecular Structure Research Centre, Yerevan, Armenia, Varian Mercury-300VX

H1 300.088 MHz, nt = 16, np = 32000, temp = 30.0 C, lb = -0.2, solvent = DMSO/CCl4 1/3

Mar 15 2022

T21-130

ANUSH_TEMA t21-130

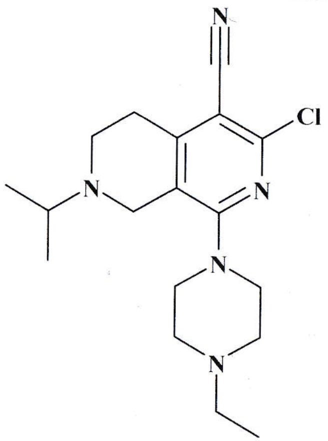

$C_{18}H_{26}ClN_5$

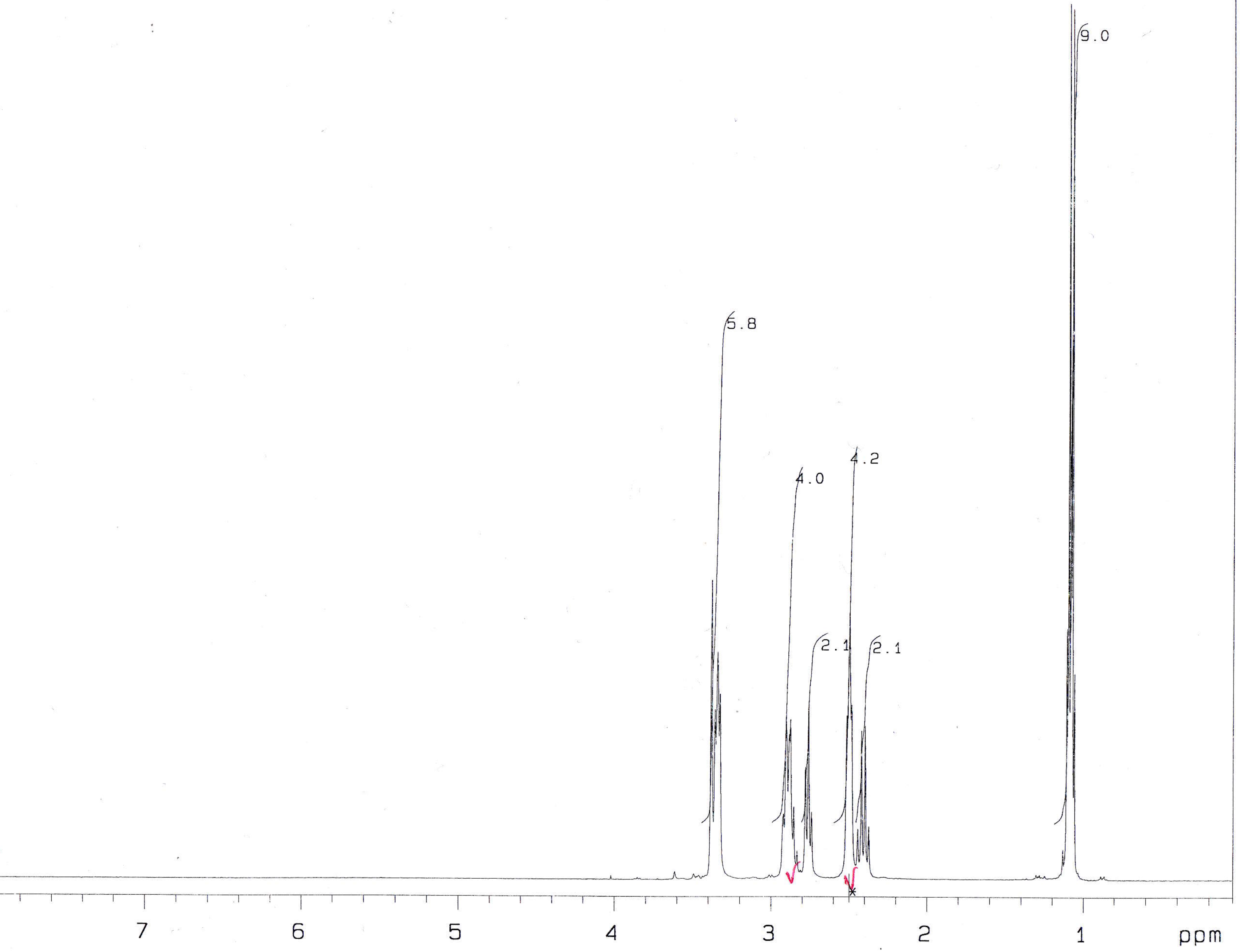

2b

Molecular Structure Research Centre, Yerevan, Armenia, Varian Mercury-300VX

C13 75.465 MHz, nt = 224, np = 19998, temp = 30.0 C, lb = 1.0, solvent = DMSO/CCl4 1/3

Mar 15 2022

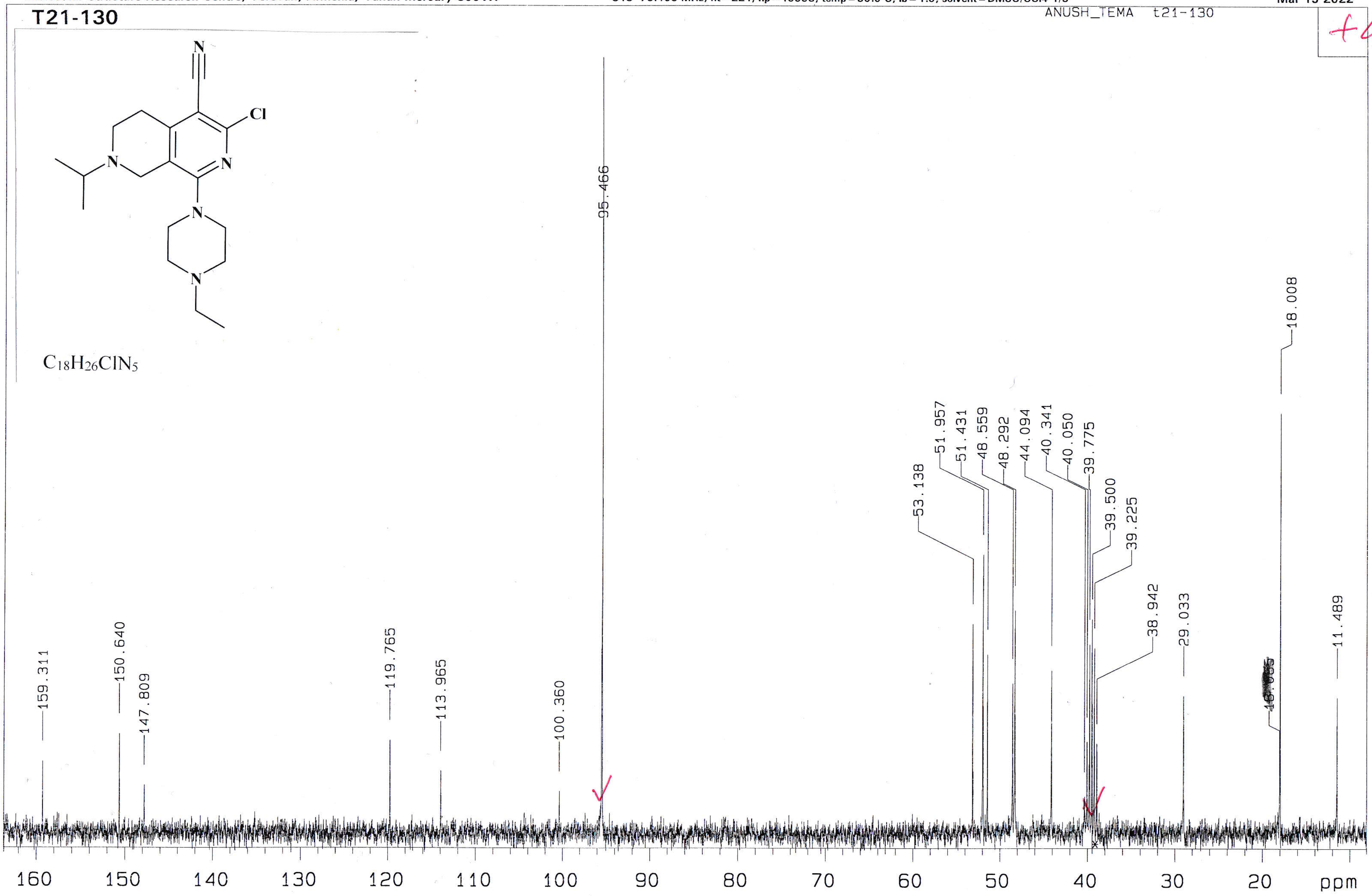

2c

Molecular Structure Research Centre, Yerevan, Armenia, Varian Mercury-300VX

H1 300.088 MHz, nt = 16, np = 32000, temp = 30.0 C, lb = -0.2, solvent = DMSO

Mar 9 2022

T21-118

NOCI_22 t21-118

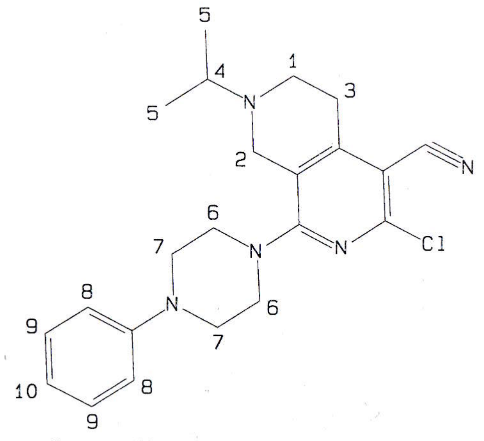

$C_{22}H_{26}ClN_5$

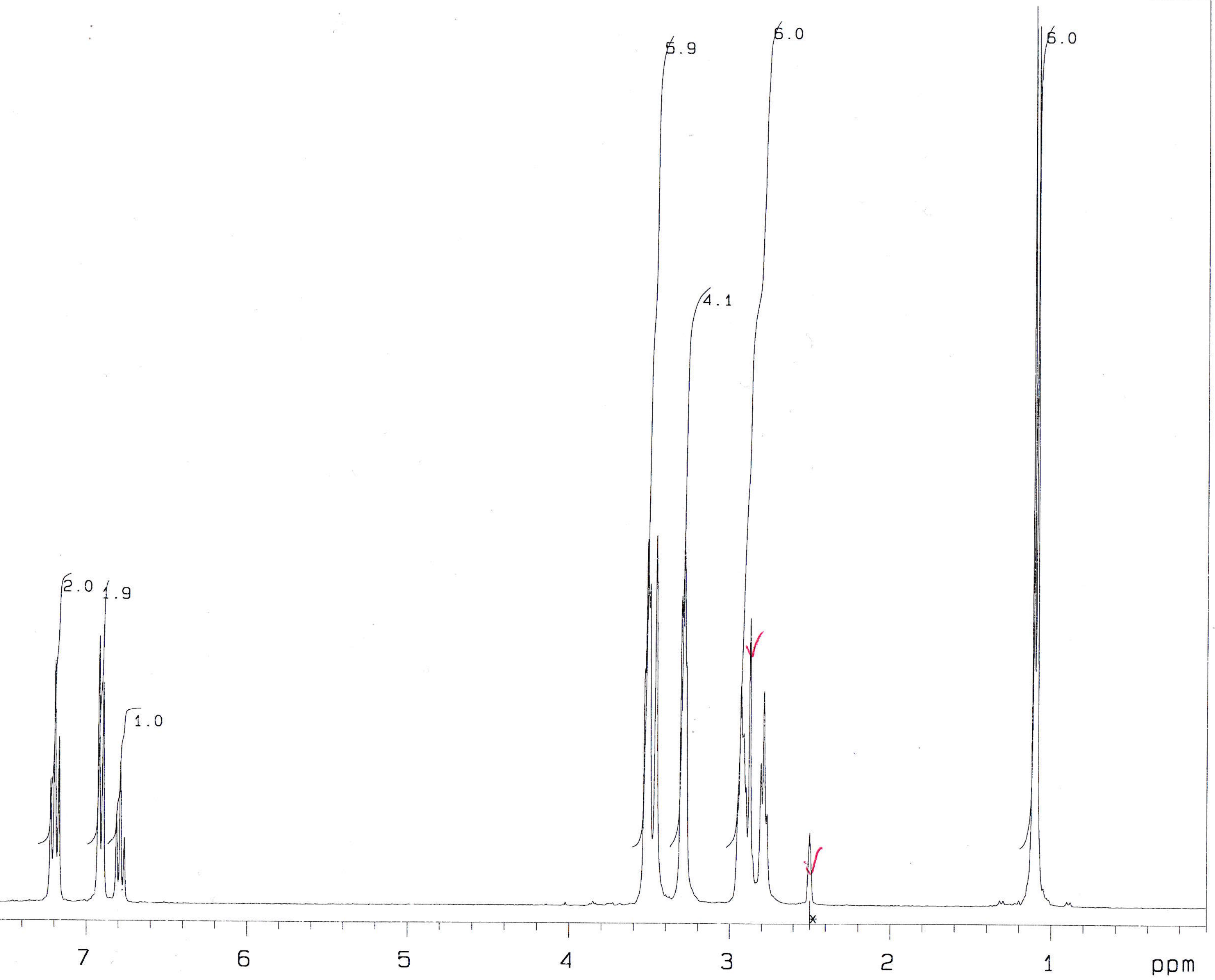

2c

Molecular Structure Research Centre, Yerevan, Armenia, Varian Mercury-300VX

C13 75.465 MHz, nt = 240, np = 19998, temp = 30.0 C, lb = 1.0, solvent = DMSO/CCl4 1/3

Mar 9 2022

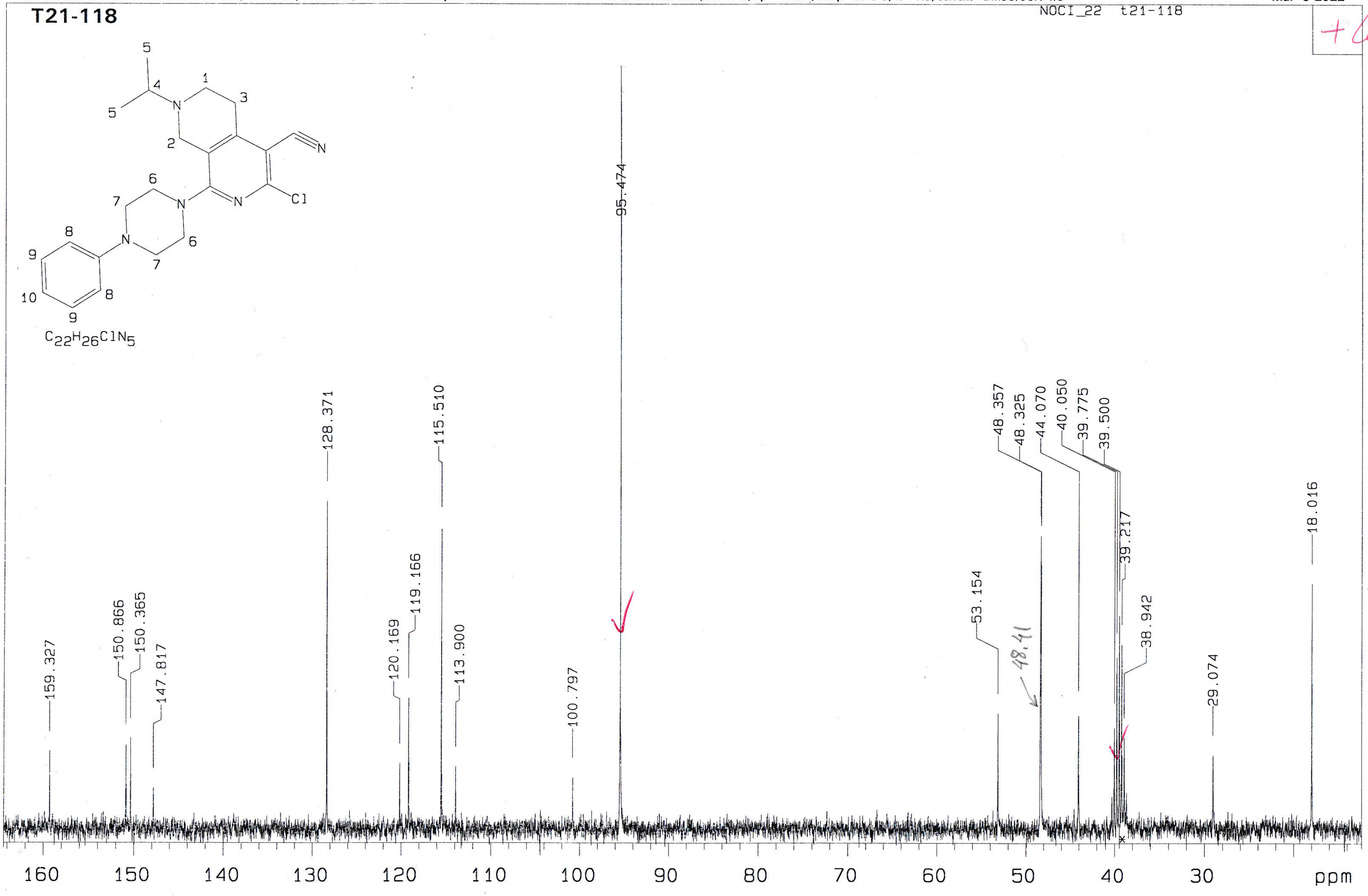

2d

Molecular Structure Research Centre, Yerevan, Armenia, Varian Mercury-300VX

H1 300.088 MHz, nt = 16, np = 32000, temp = 30.0 C, lb = -0.2, solvent = DMSO

Mar 25 2022

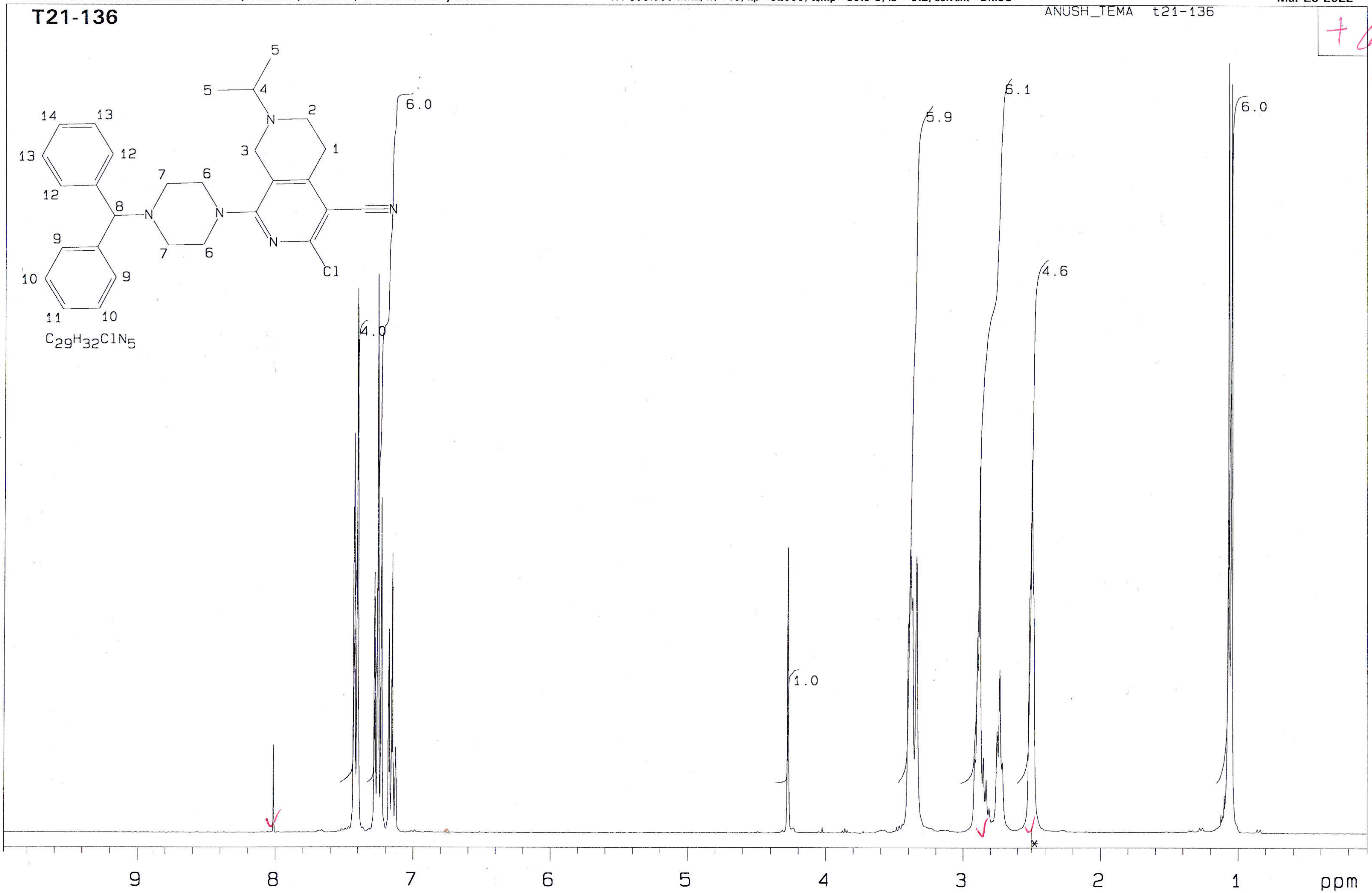

2d

Molecular Structure Research Centre, Yerevan, Armenia, Varian Mercury-300VX

C13 75.465 MHz, nt = 304, np = 19998, temp = 30.0 C, lb = 1.0, solvent = DMSO/CCl4 1/3

Mar 25 2022

ANUSH_TEMA t21-136

T21-136

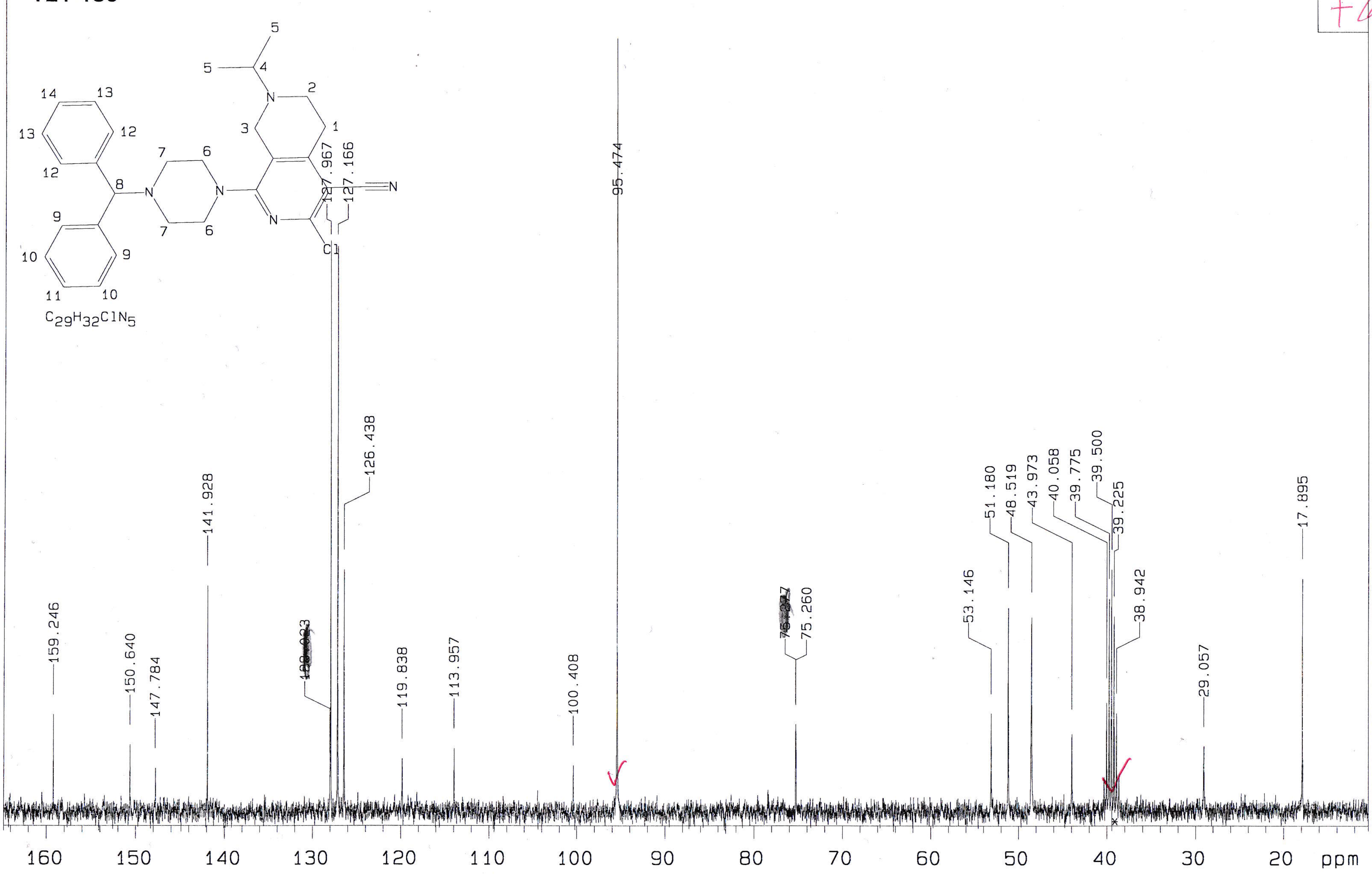

2f

Molecular Structure Research Centre, Yerevan, Armenia, Varian Mercury-300VX

H1 300.088 MHz, nt = 16, np = 32000, temp = 30.0 C, lb = -0.2, solvent = DMSO

Jul 8 2022

T21-183

SM-016

ANUSH_TEMA t21-183

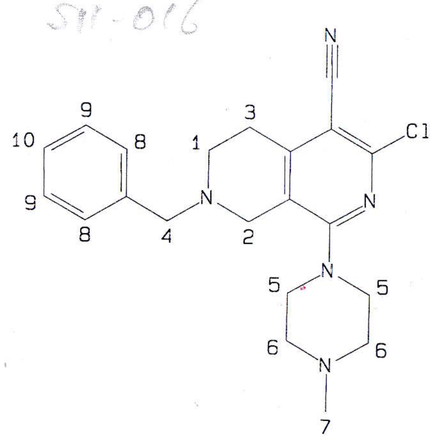

$C_{21}H_{24}ClN_5$

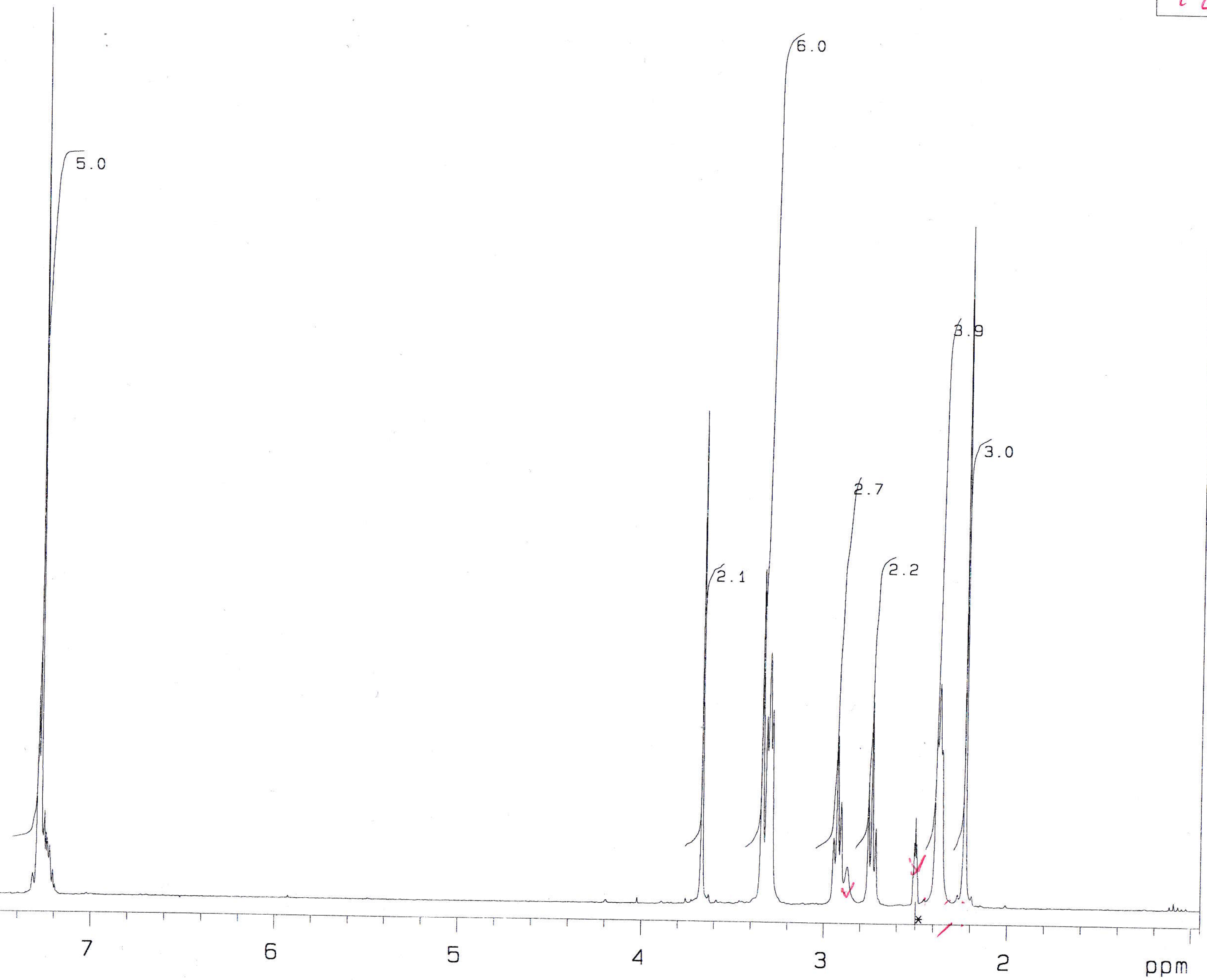

2f

Molecular Structure Research Centre, Yerevan, Armenia, Varian Mercury-300VX

C13 75.465 MHz, nt = 400, np = 19998, temp = 30.0 C, lb = 1.0, solvent = DMSO/CCl4 1/3

Jul 8 2022

T21-183

ANUSH_TEMA t21-183

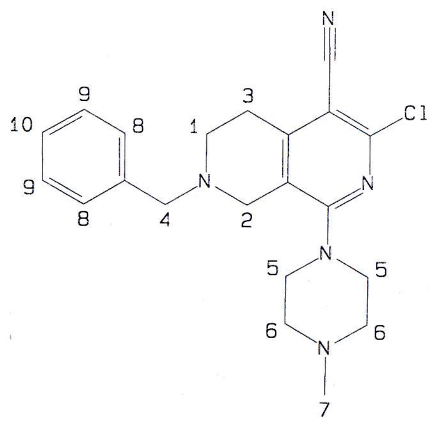

$C_{21}H_{24}ClN_5$

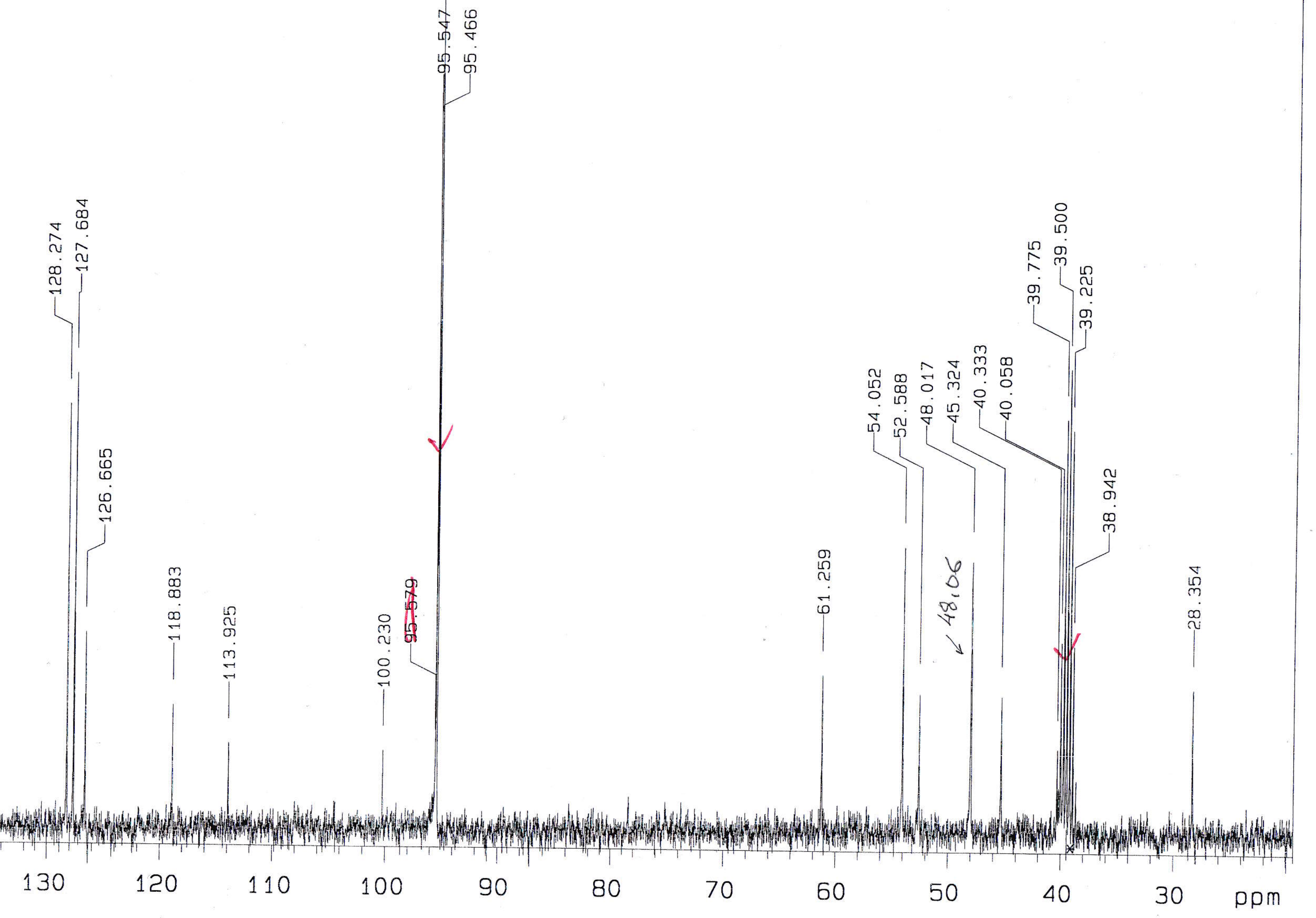

2g

Molecular Structure Research Centre, Yerevan, Armenia, Varian Mercury-300VX

H1 300.088 MHz, nt=16, np=32000, temp=30.0 C, lb=-0.2, solvent=DMSO/CCl4 1/3

Jan 12 2023

ANUSH_TEMA t21-215

T21-215

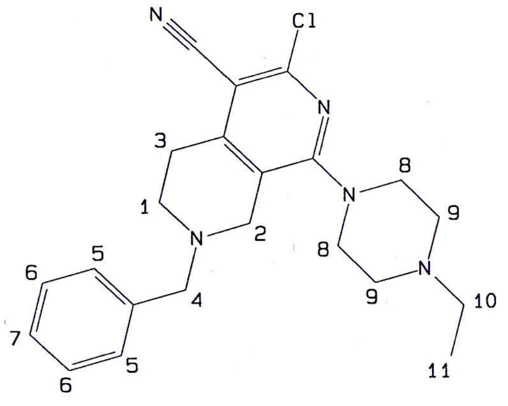

$C_{22}H_{26}ClN_5$

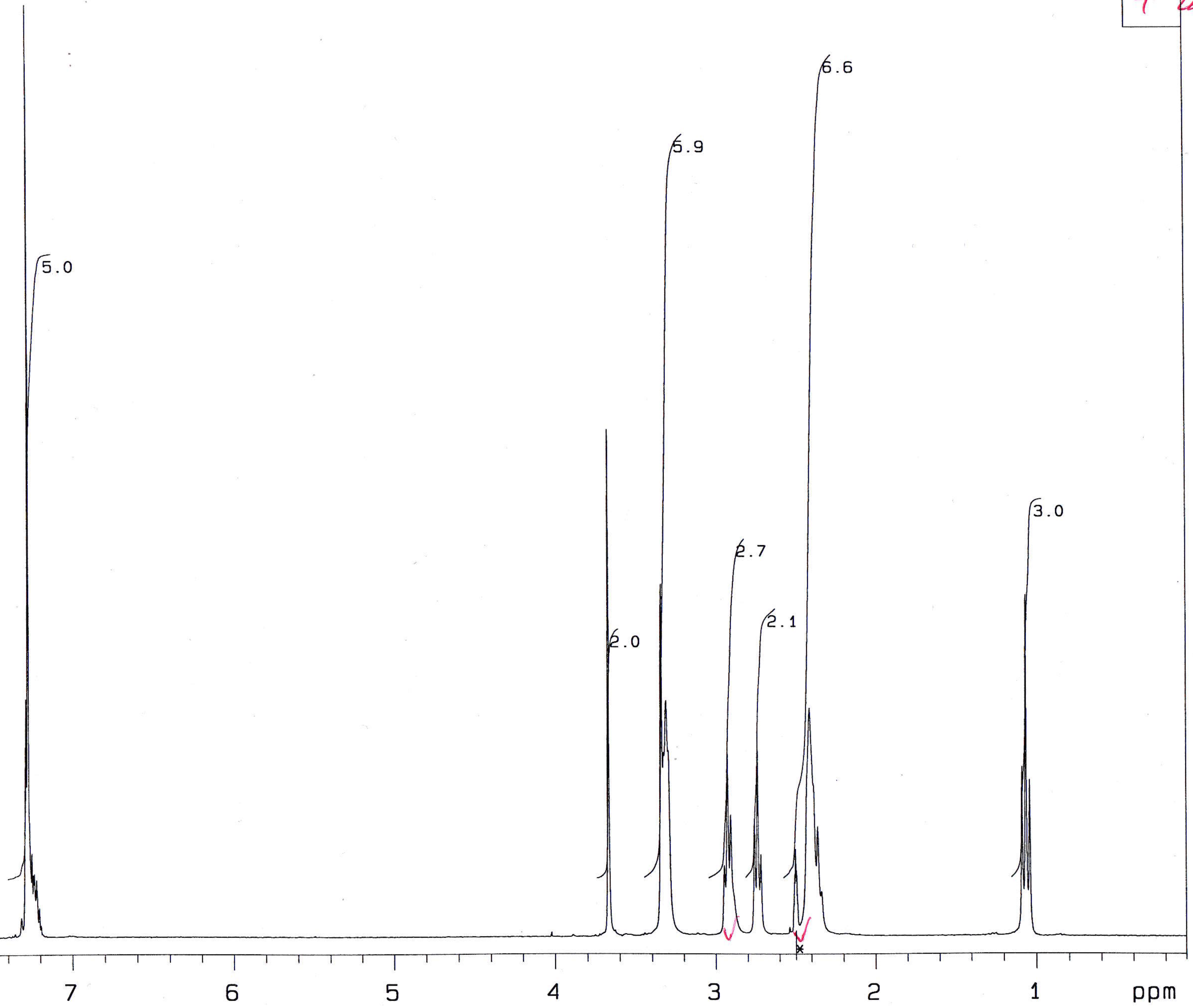

+ [signature]

Molecular Structure Research Centre, Yerevan, Armenia, Varian Mercury-300VX

C13 75.465 MHz, nt=512, np=19998, temp=30.0 C, lb=1.0, solvent=DMSO/CCl4 1/3

Jan 12 2023

ANUSH_TEMA t21-215

T21-215

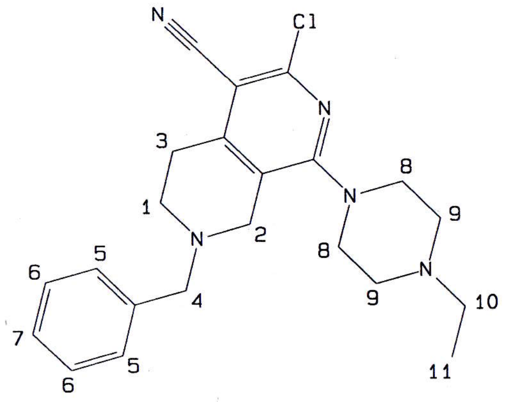

$C_{22}H_{26}ClN_5$

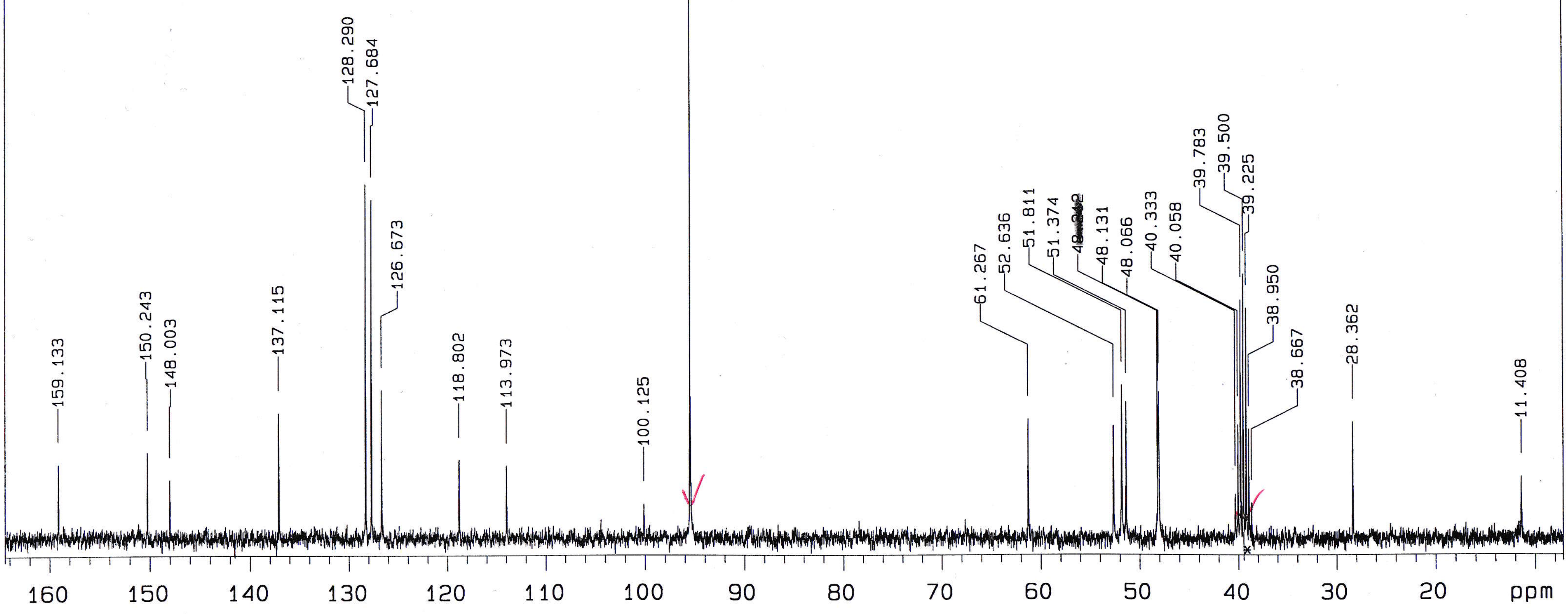

2h

Molecular Structure Research Centre, Yerevan, Armenia, Varian Mercury-300VX

H1 300.088 MHz, nt = 16, np = 32000, temp = 30.0 C, lb = -0.2, solvent = DMSO/CCl4 1/3

Jan 12 2023

ANUSH_TEMA t21-216

T21-216

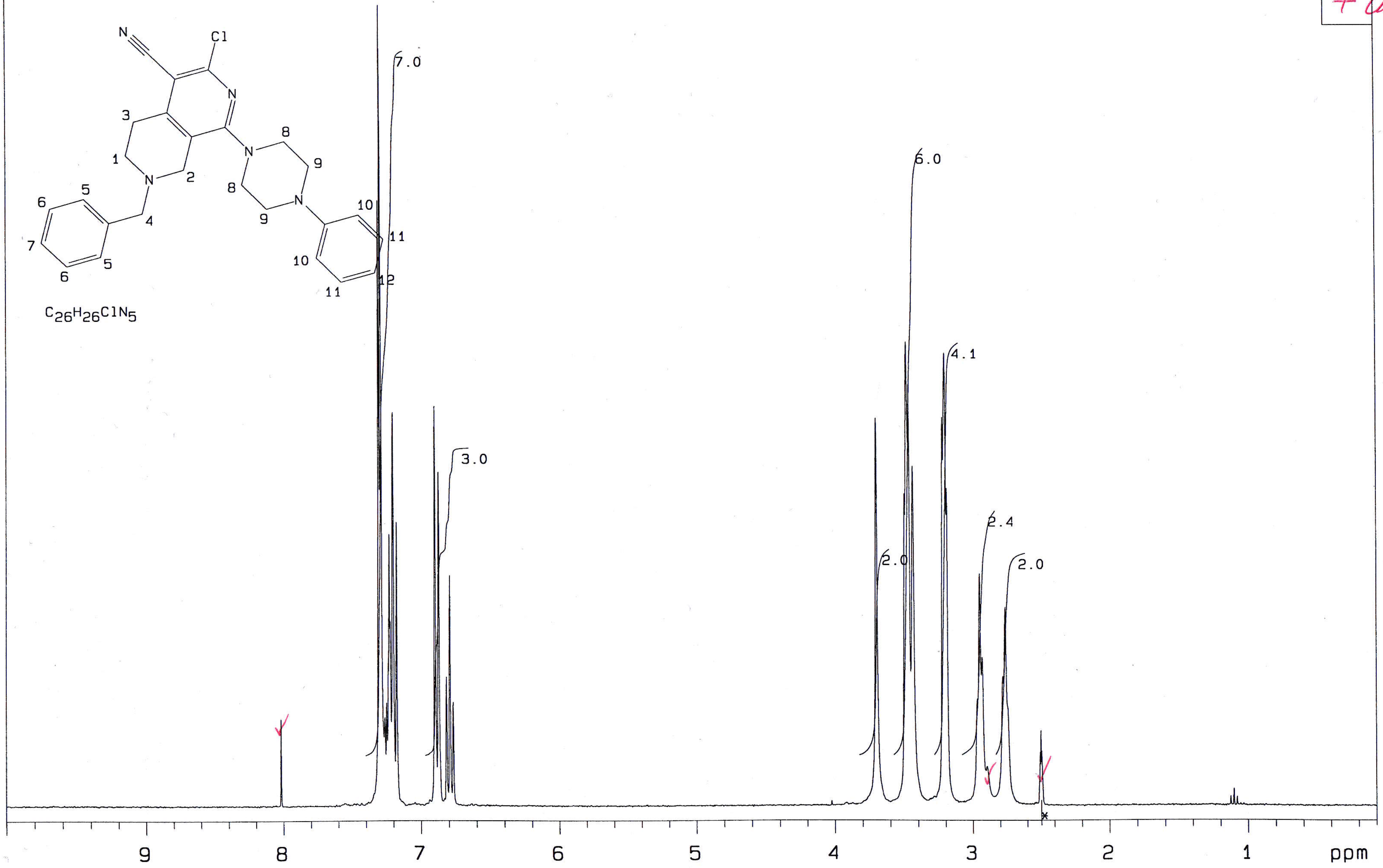

+ [illegible]

2h

Molecular Structure Research Centre, Yerevan, Armenia, Varian Mercury-300VX

C13 75.465 MHz, nt = 256, np = 19998, temp = 30.0 C, lb = 1.0, solvent = DMSO/CCl4 1/3

Jan 12 2023

ANUSH_TEMA t21-216

T21-216

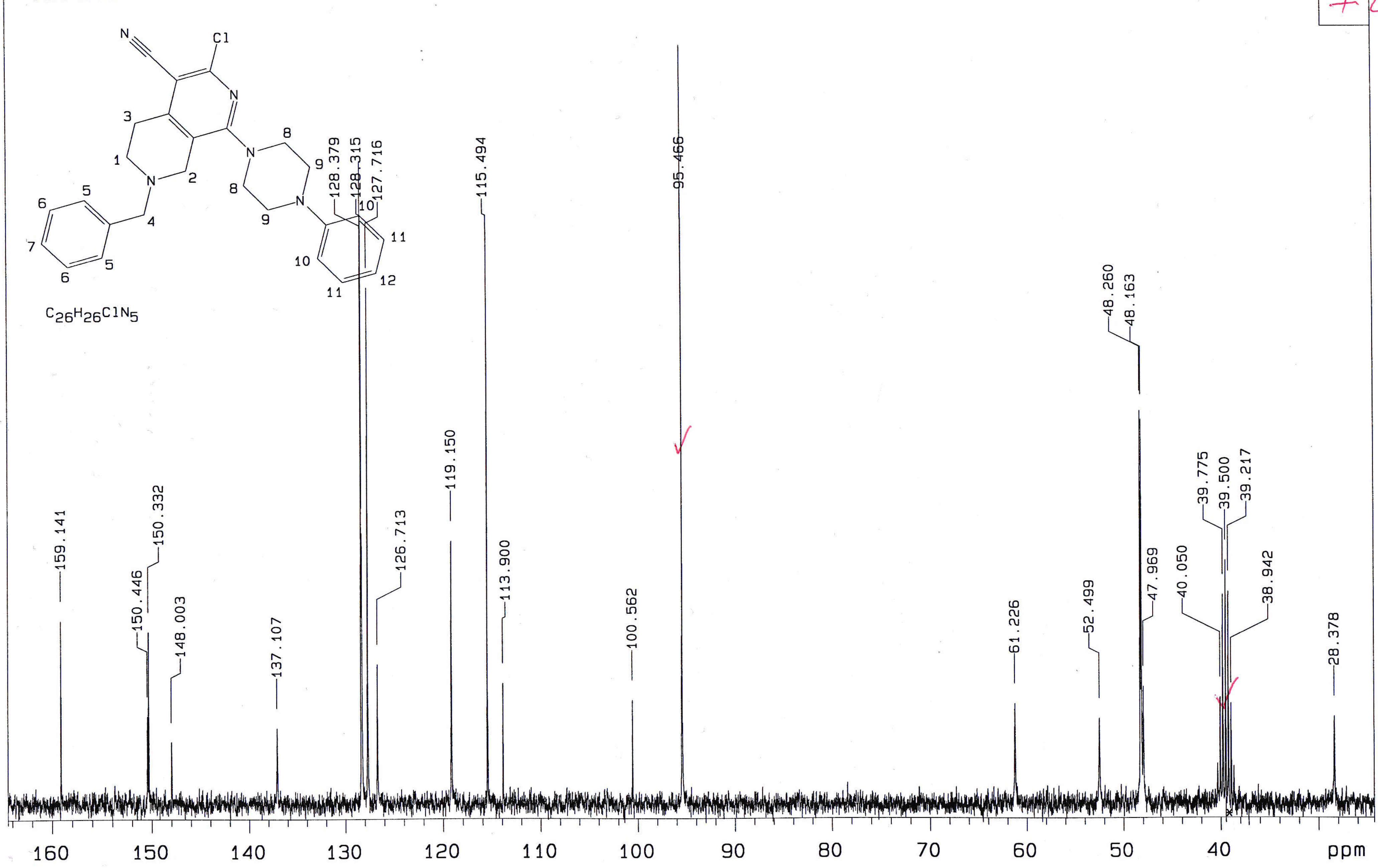

2i

Molecular Structure Research Centre, Yerevan, Armenia, Varian Mercury-300VX

H1 300.088 MHz, nt = 16, np = 32000, temp = 30.0 C, lb = -0.2, solvent = DMSO/CCl4 1/3

Jan 23 2023

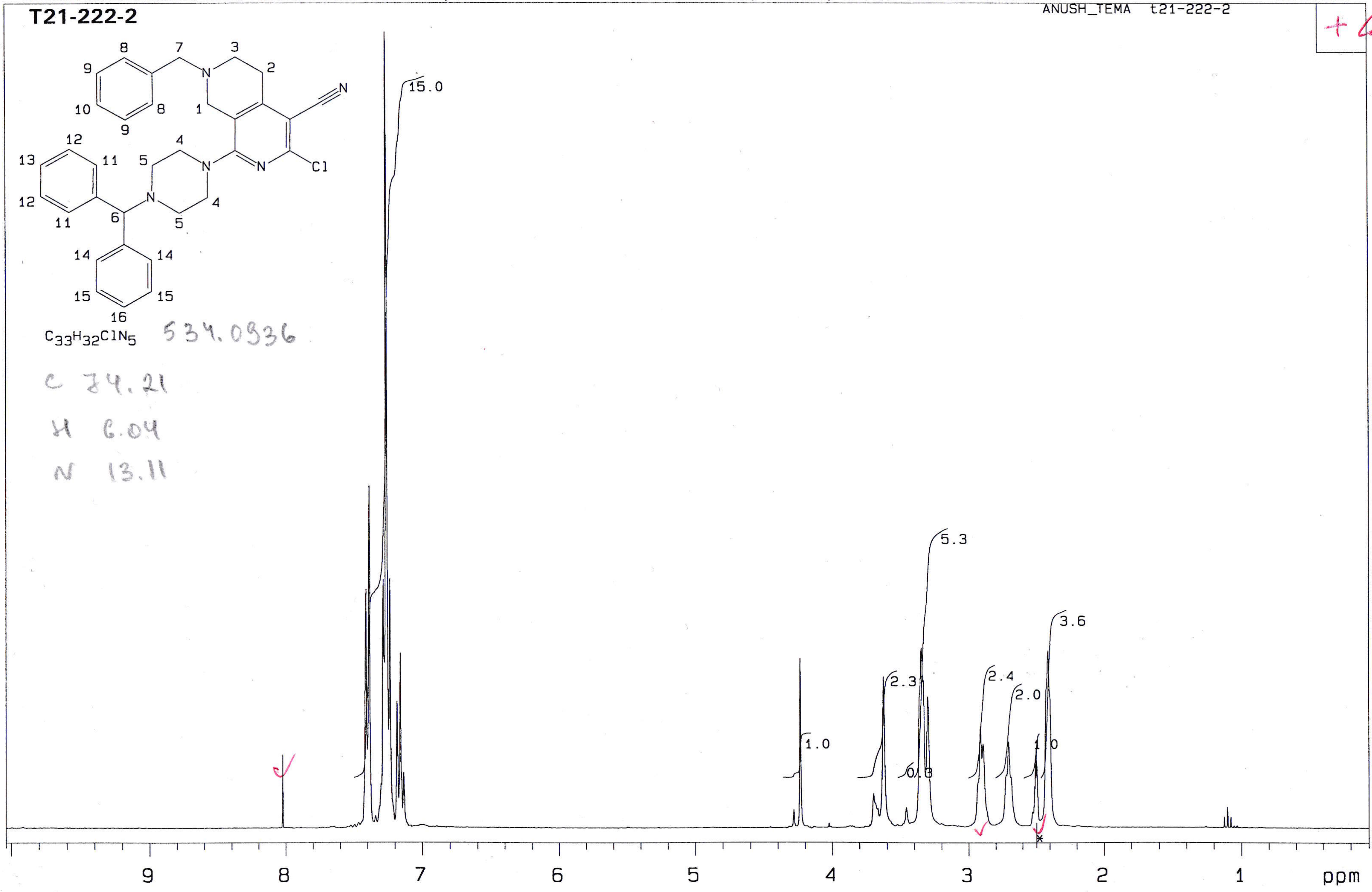

2j

Molecular Structure Research Centre, Yerevan, Armenia, Varian Mercury-300VX

H1 300.088 MHz, nt = 16, np = 32000, temp = 30.0 C, lb = -0.2, solvent = DMSO/CCl4 1/3

Jan 10 2023

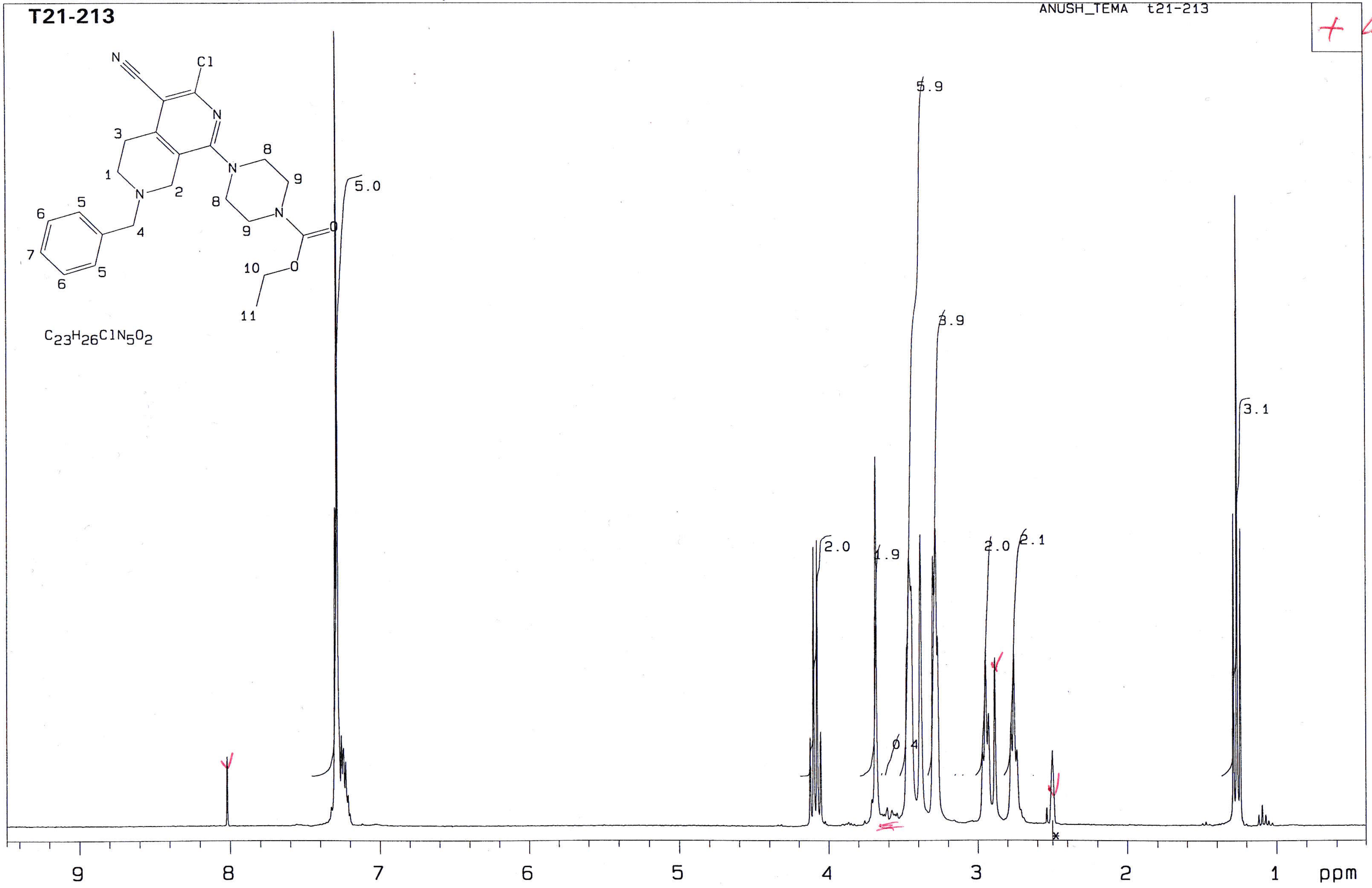

2j

Molecular Structure Research Centre, Yerevan, Armenia, Varian Mercury-300VX

C13 75.465 MHz, nt=400, np=19998, temp=30.0 C, lb=1.0, solvent=DMSO/CCl4 1/3

Jan 10 2023

ANUSH_TEMA t21-213

T21-213

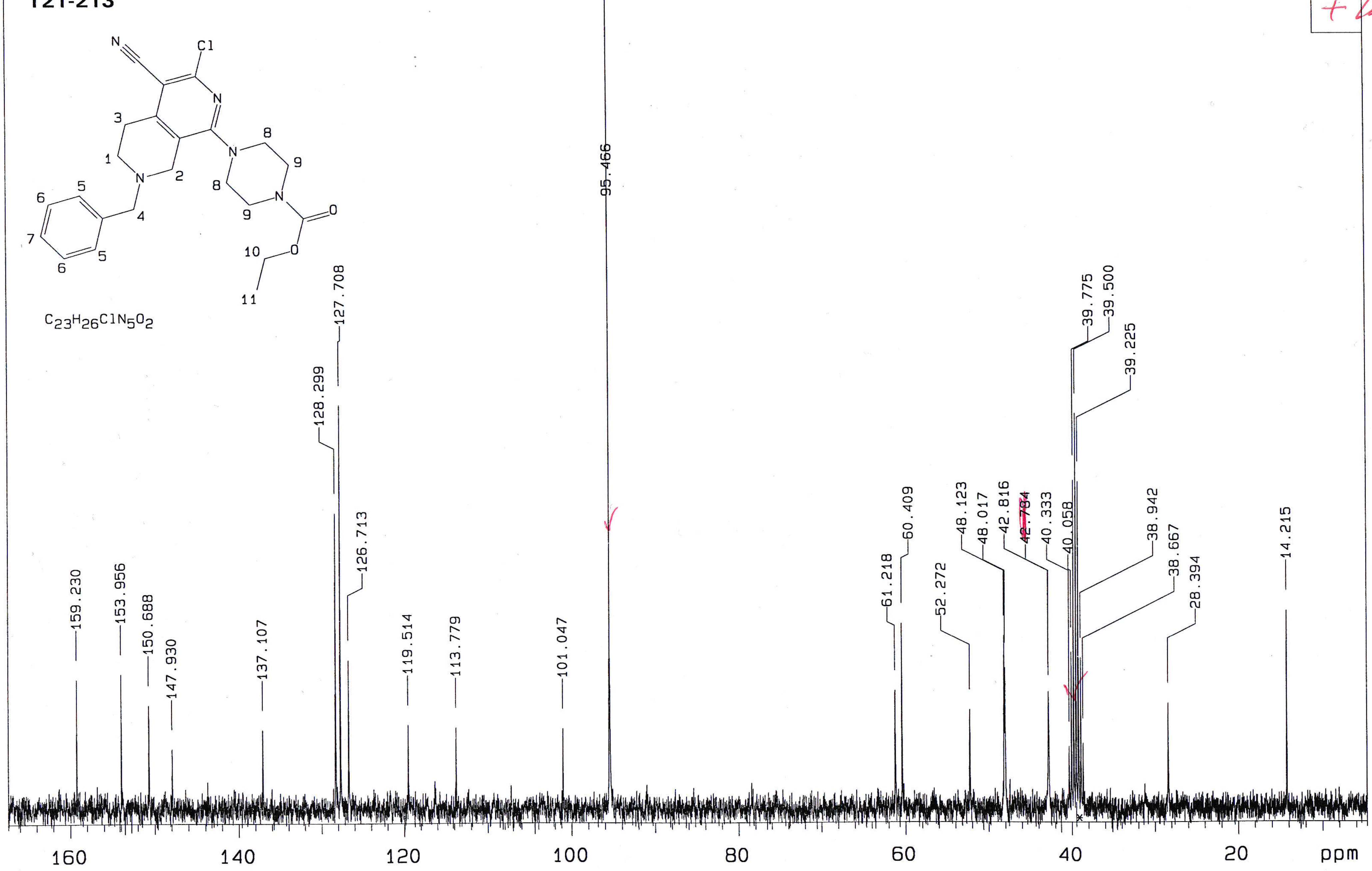

+

3a

Molecular Structure Research Centre, Yerevan, Armenia, Varian Mercury-300VX

H1 300.088 MHz, nt = 16, np = 32000, temp = 30.0 C, lb = -0.2, solvent = DMSO/CCl4 1/3

Mar 22 2022

T21-133

ANUSH_TEMA t21-133

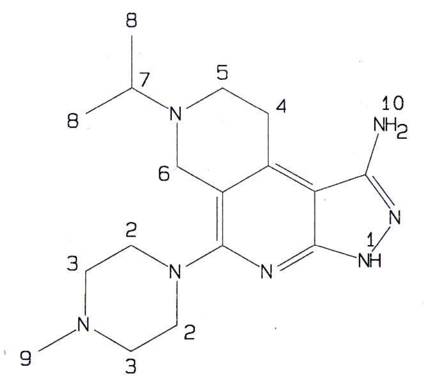

$C_{17}H_{27}N_7$

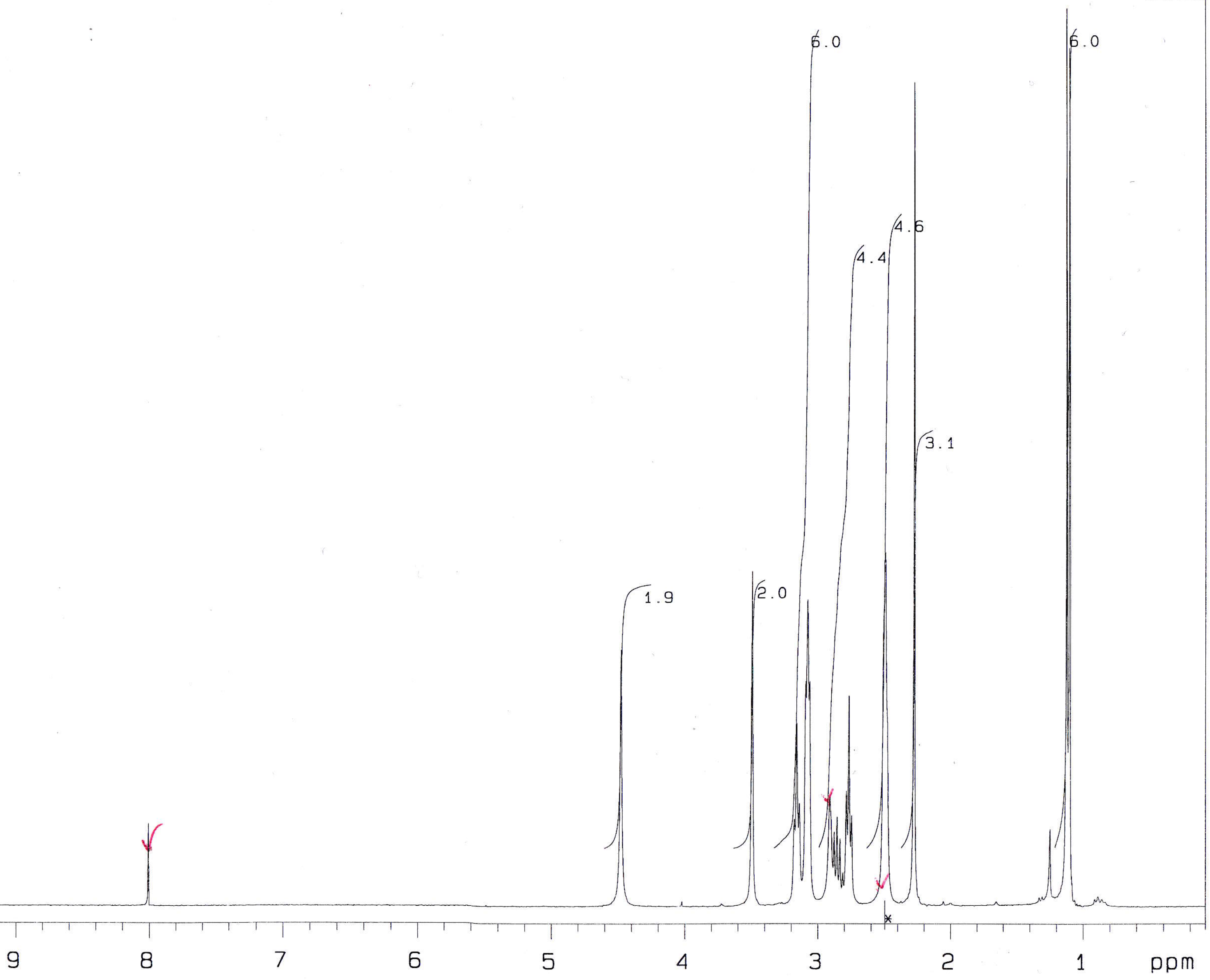

3a

Molecular Structure Research Centre, Yerevan, Armenia, Varian Mercury-300VX

C13 75.465 MHz, nt=400, np=19998, temp=30.0 C, lb=1.0, solvent=DMSO/CCl4 1/3

Mar 22 2022

ANUSH_TEMA t21-133

T21-133

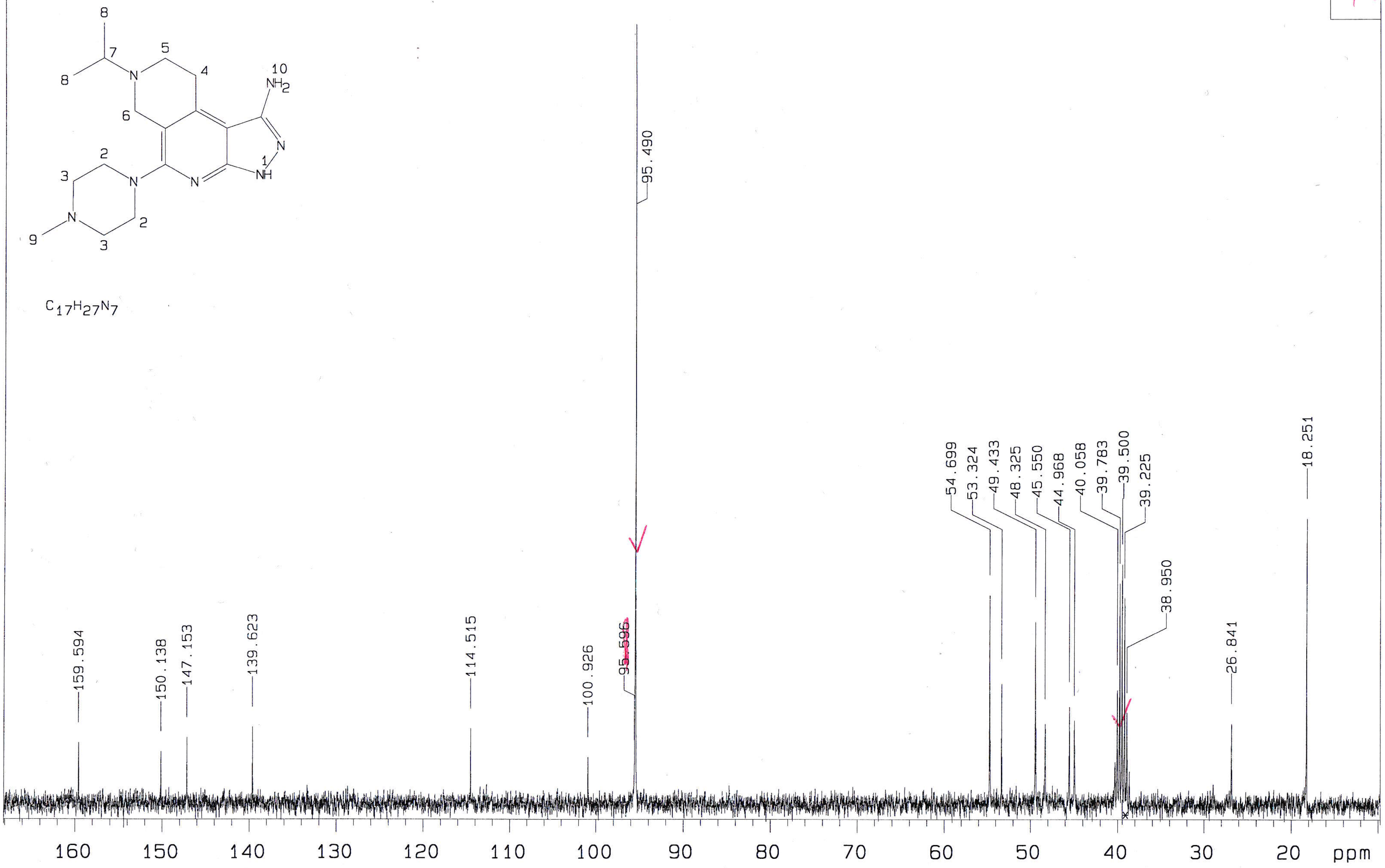

3a

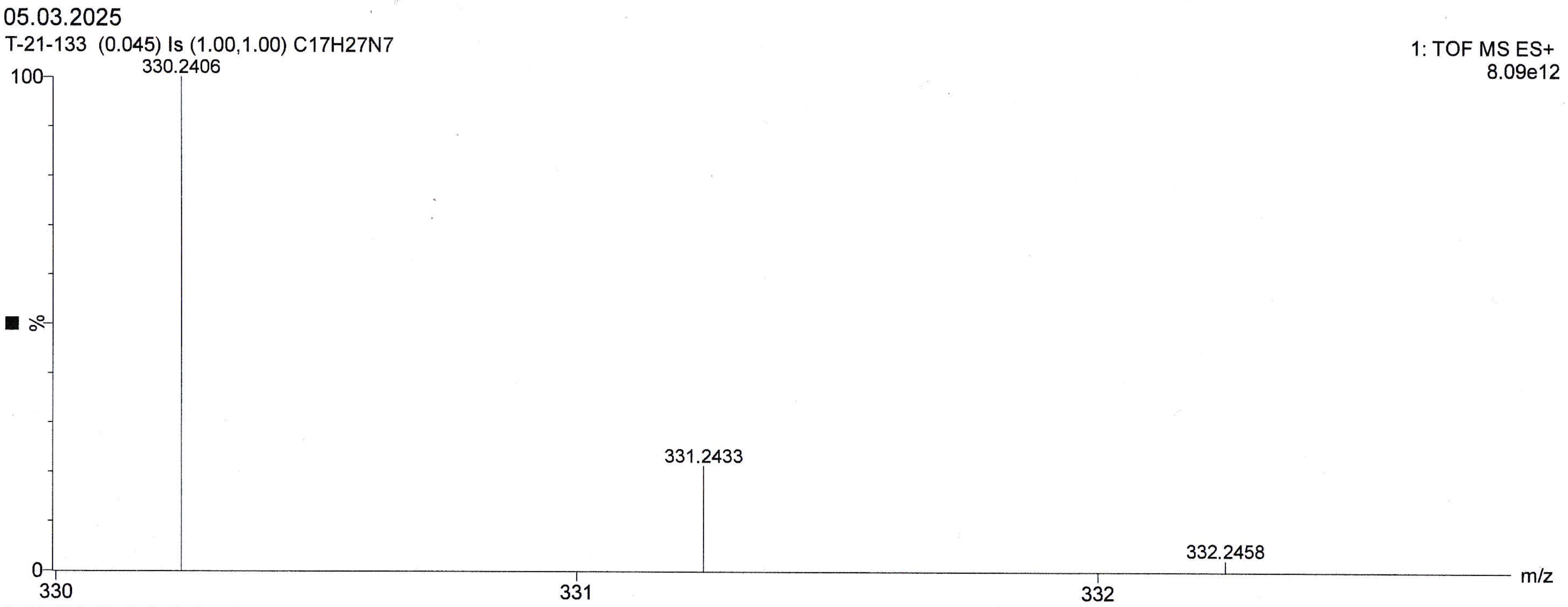

05.03.2025
T-21-133 (0.045) Is (1.00,1.00) C17H27N7
1: TOF MS ES+
8.09e12
100
%
0
330.2406
331.2433
332.2458
330
331
332
m/z

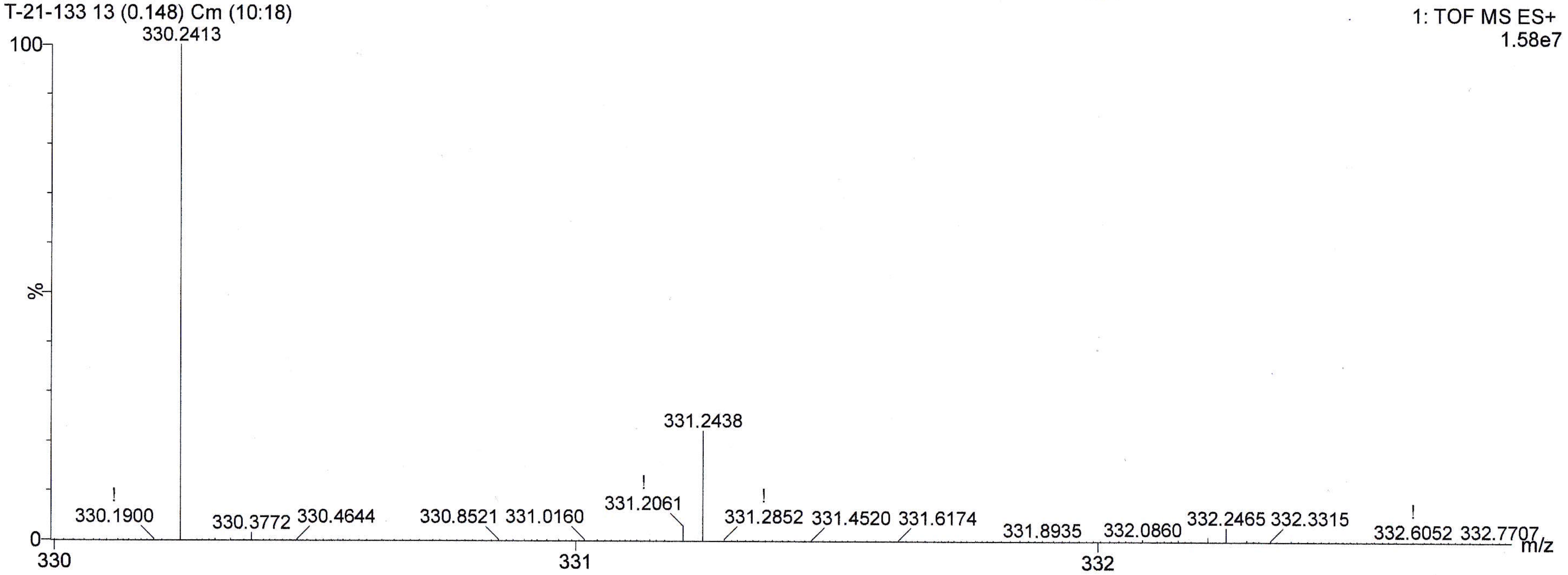

T-21-133 13 (0.148) Cm (10:18)
1: TOF MS ES+
1.58e7
100
%
0
330.2413
330.1900
330.3772
330.4644
330.8521
331.0160
331.2061
331.2438
331.2852
331.4520
331.6174
331.8935
332.0860
332.2465
332.3315
332.6052
332.7707
330
331
332
m/z

36

Molecular Structure Research Centre, Yerevan, Armenia, Varian Mercury-300VX

H1 300.088 MHz, nt = 16, np = 32000, temp = 30.0 C, lb = -0.2, solvent = DMSO/CCl4 1/3

Mar 23 2022

ANUSH_TEMA t21-132

T21-132

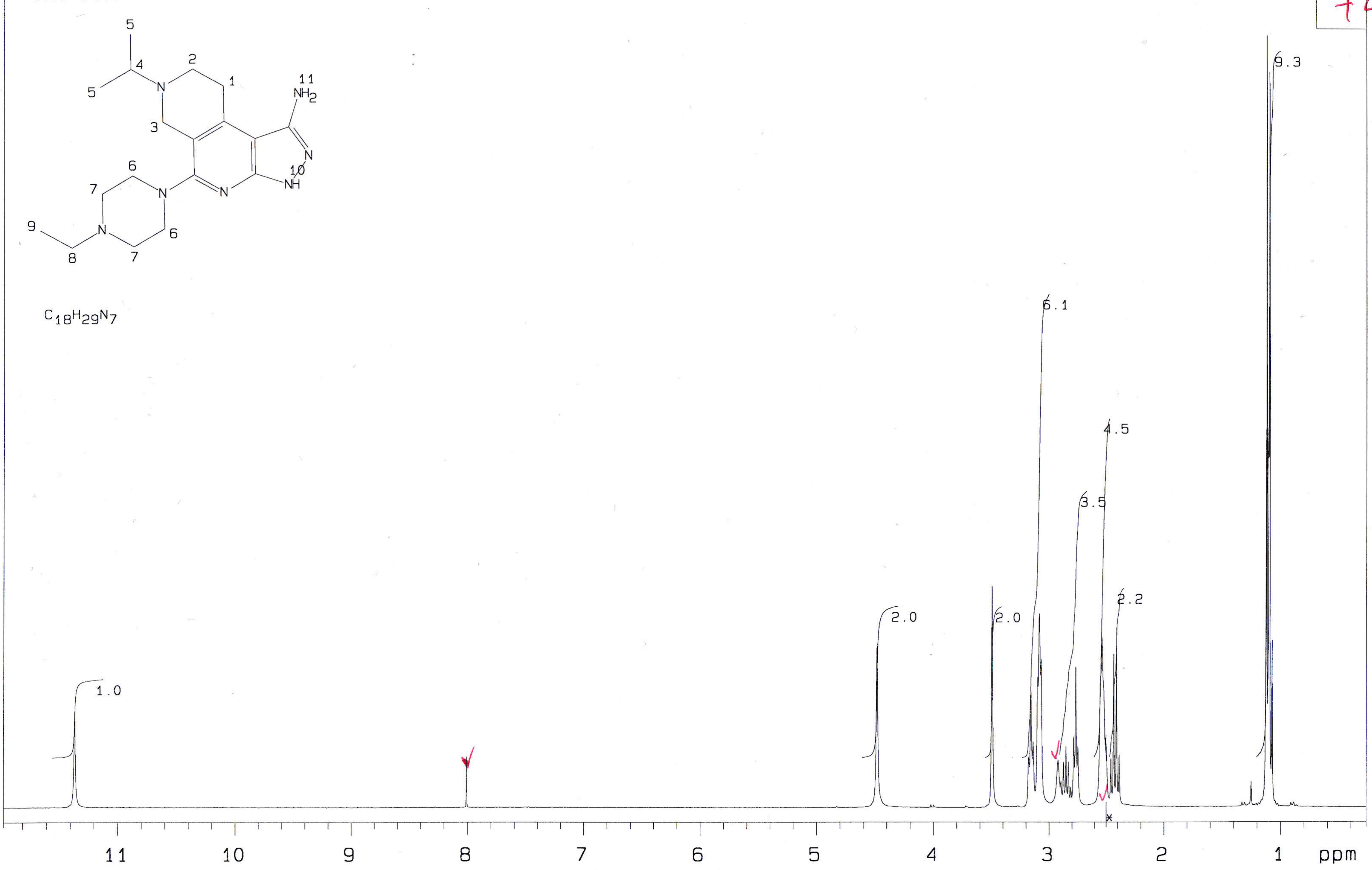

3b

Molecular Structure Research Centre, Yerevan, Armenia, Varian Mercury-300VX

C13 75.465 MHz, nt = 176, np = 19998, temp = 30.0 C, lb = 1.0, solvent = DMSO/CCl4 1/3

Mar 23 2022

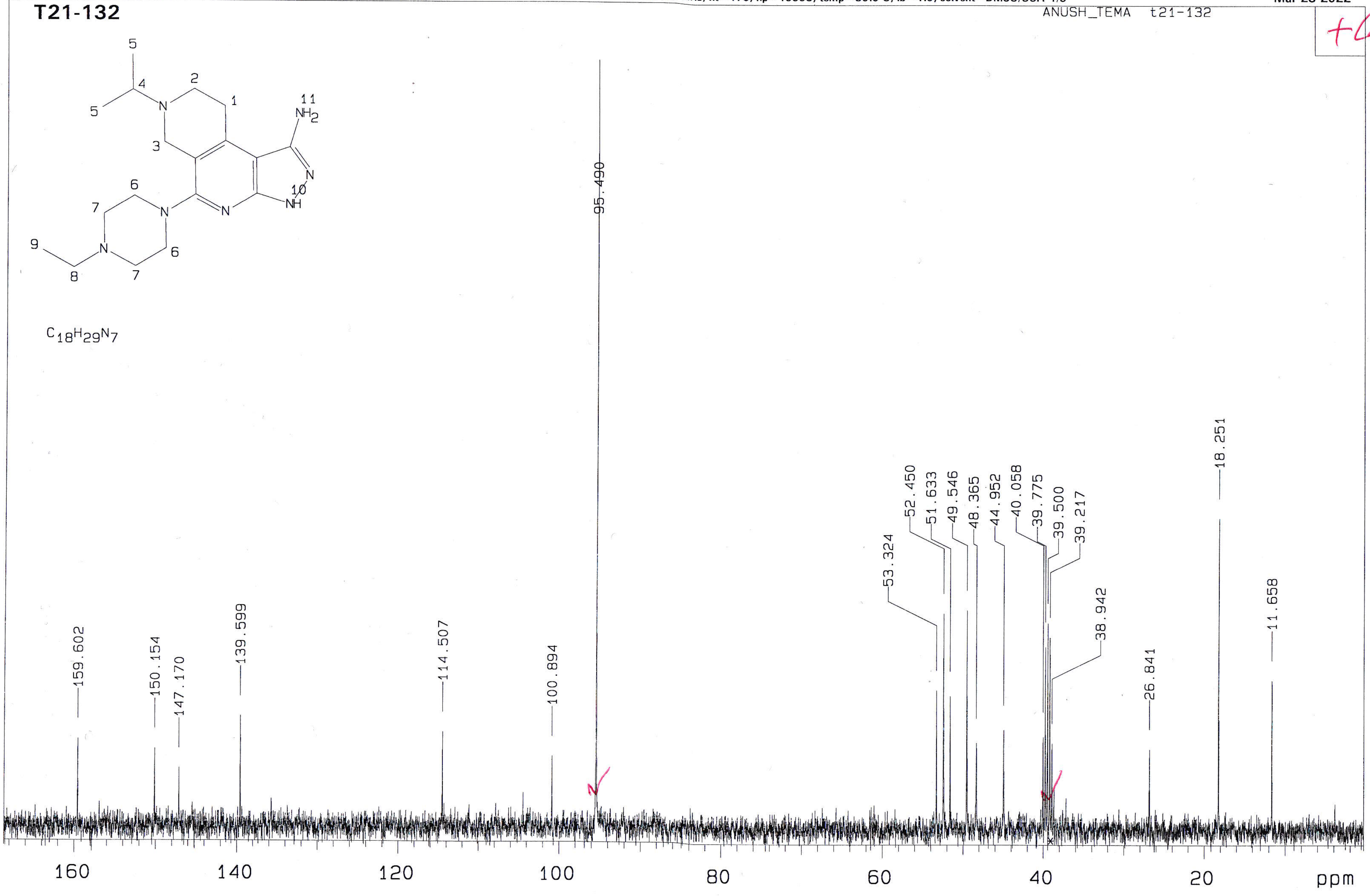

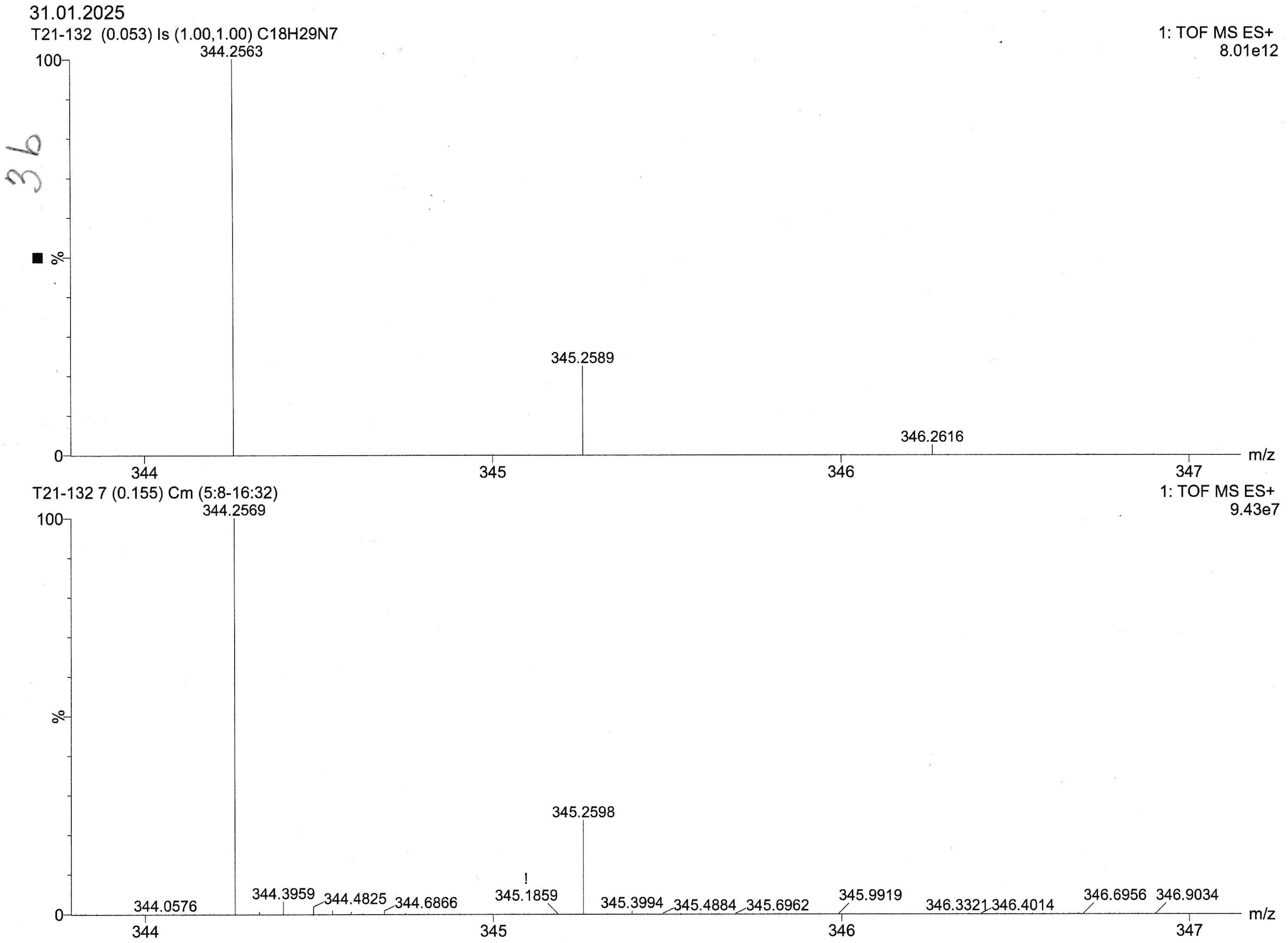
3b
31.01.2025
T21-132 (0.053) Is (1.00,1.00) C18H29N7
1: TOF MS ES+
8.01e12
344.2563
345.2589
346.2616
100
%
0
344
345
346
347
m/z
T21-132 7 (0.155) Cm (5:8-16:32)
1: TOF MS ES+
9.43e7
344.2569
344.0576
344.3959
344.4825
344.6866
345.1859
345.2598
345.3994
345.4884
345.6962
345.9919
346.3321
346.4014
346.6956
346.9034
100
%
0
344
345
346
347
m/z

3c

Molecular Structure Research Centre, Yerevan, Armenia, Varian Mercury-300VX

H1 300.088 MHz, nt = 16, np = 32000, temp = 30.0 C, lb = -0.2, solvent = DMSO/CCl4 1/3

Mar 17 2022

T21-131

ANUSH_TEMA t21-131

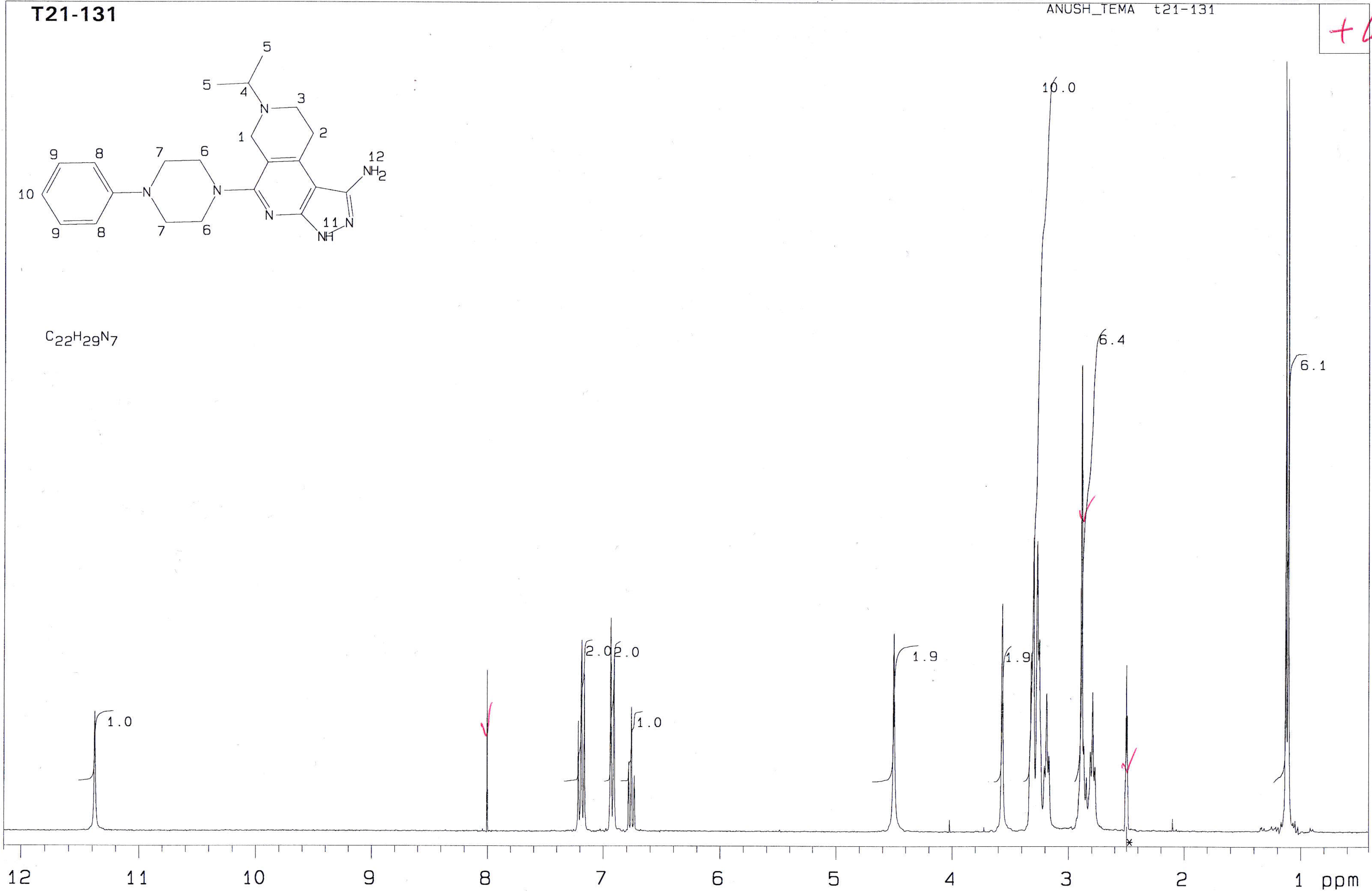

3c

Molecular Structure Research Centre, Yerevan, Armenia, Varian Mercury-300VX

C13 75.465 MHz, nt = 864, np = 19998, temp = 30.0 C, lb = 2.0, solvent = DMSO/CCl4 1/3

Mar 17 2022

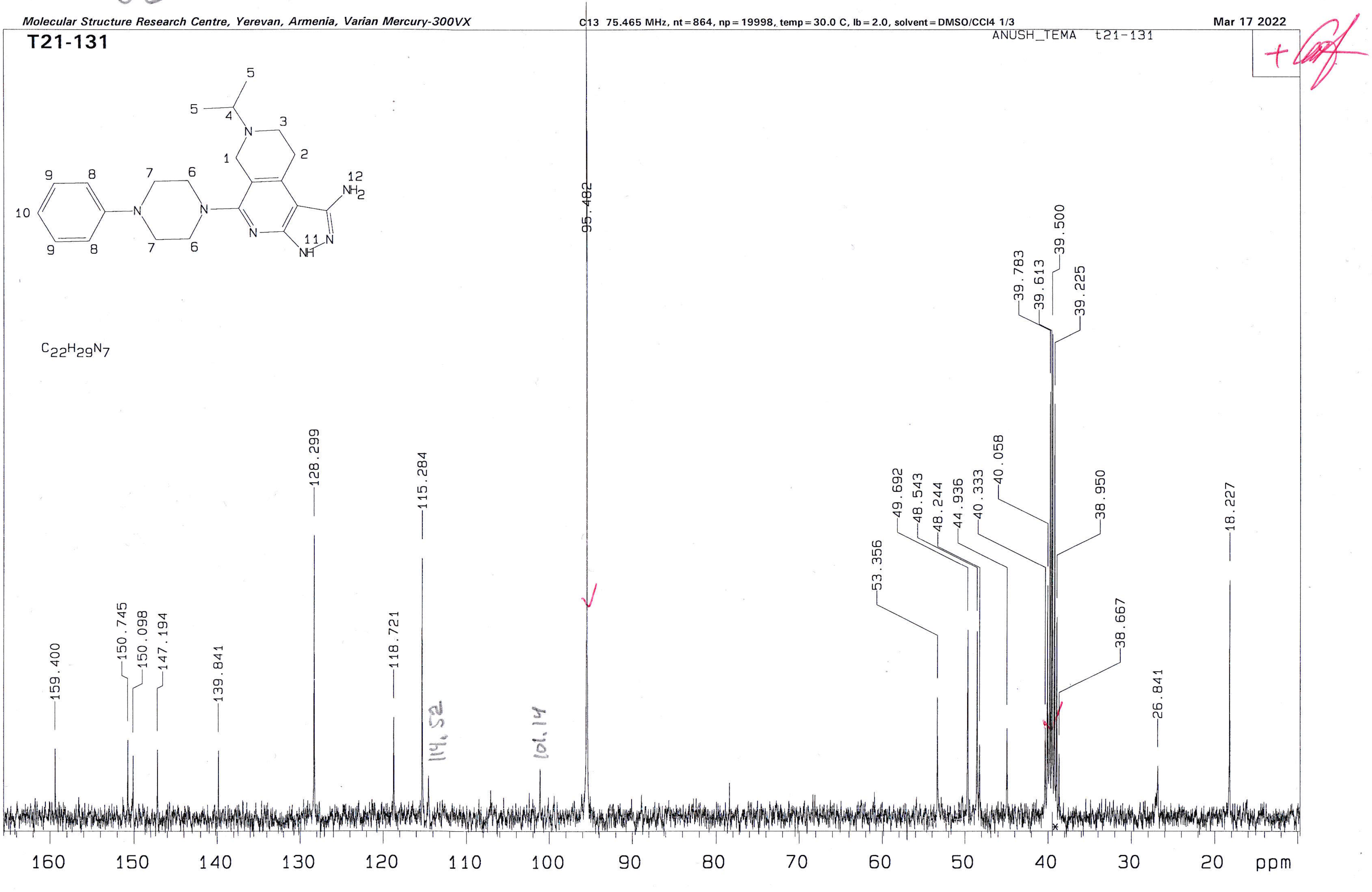

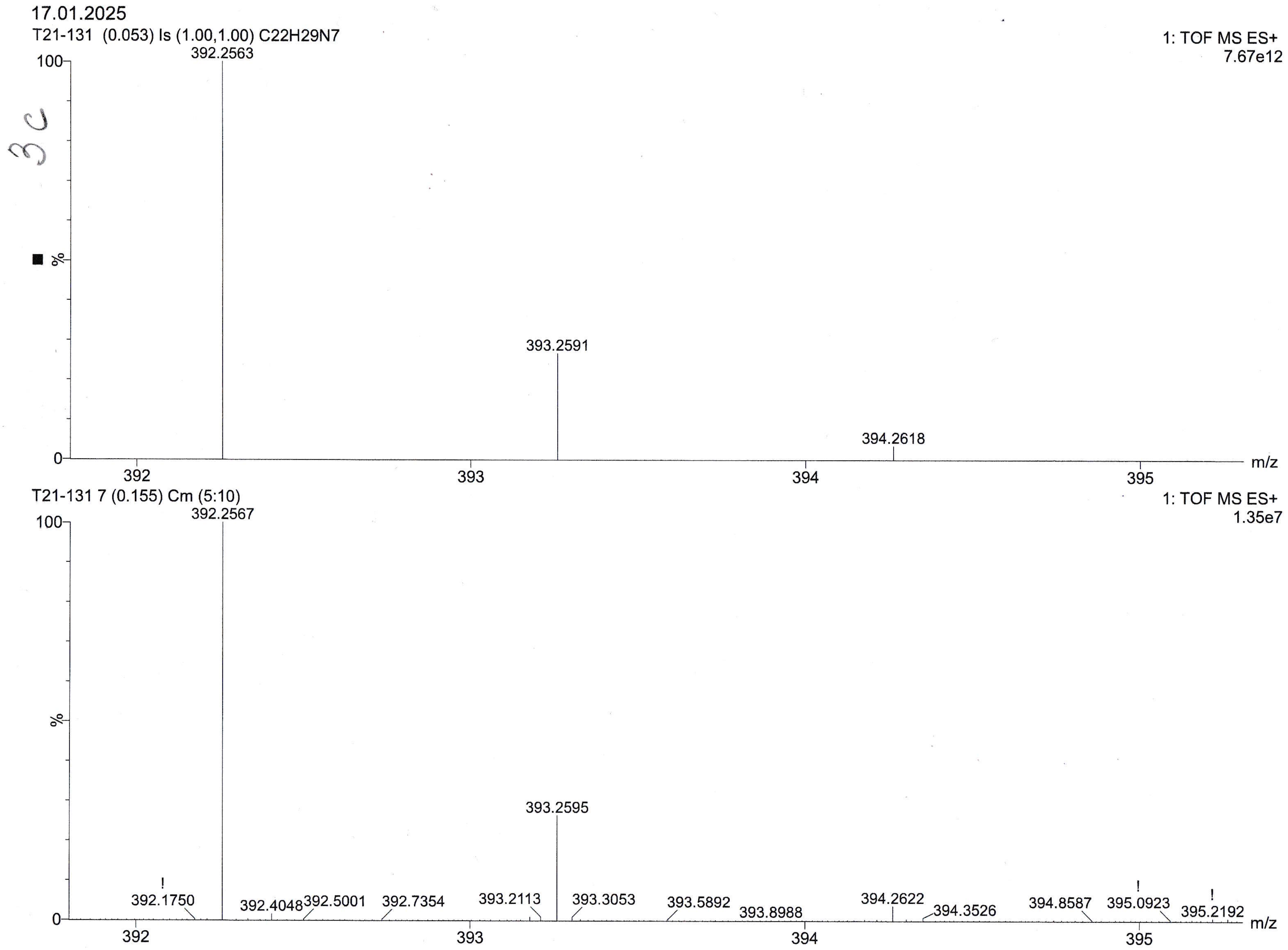
3c
17.01.2025
T21-131 (0.053) Is (1.00,1.00) C22H29N7
1: TOF MS ES+
7.67e12
100
%
0
392.2563
393.2591
394.2618
392
393
394
395
m/z
T21-131 7 (0.155) Cm (5:10)
1: TOF MS ES+
1.35e7
100
%
0
392.2567
392.1750
392.4048
392.5001
392.7354
393.2113
393.2595
393.3053
393.5892
393.8988
394.2622
394.3526
394.8587
395.0923
395.2192
392
393
394
395
m/z

3d

Molecular Structure Research Centre, Yerevan, Armenia, Varian Mercury-300VX

H1 300.088 MHz, nt=16, np=32000, temp=30.0 C, lb=-0.2, solvent=DMSO/CCl4 1/3

Mar 30 2022

T21-142

ANUSH_TEMA t21-142

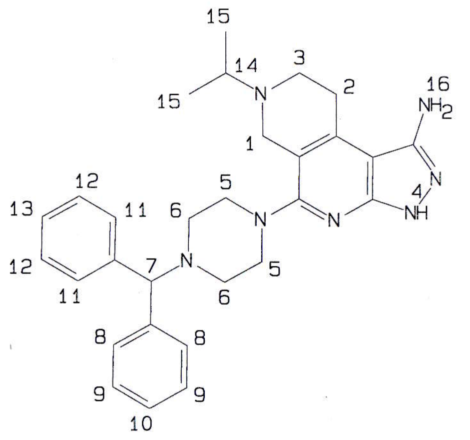

$C_{29}H_{35}N_7$

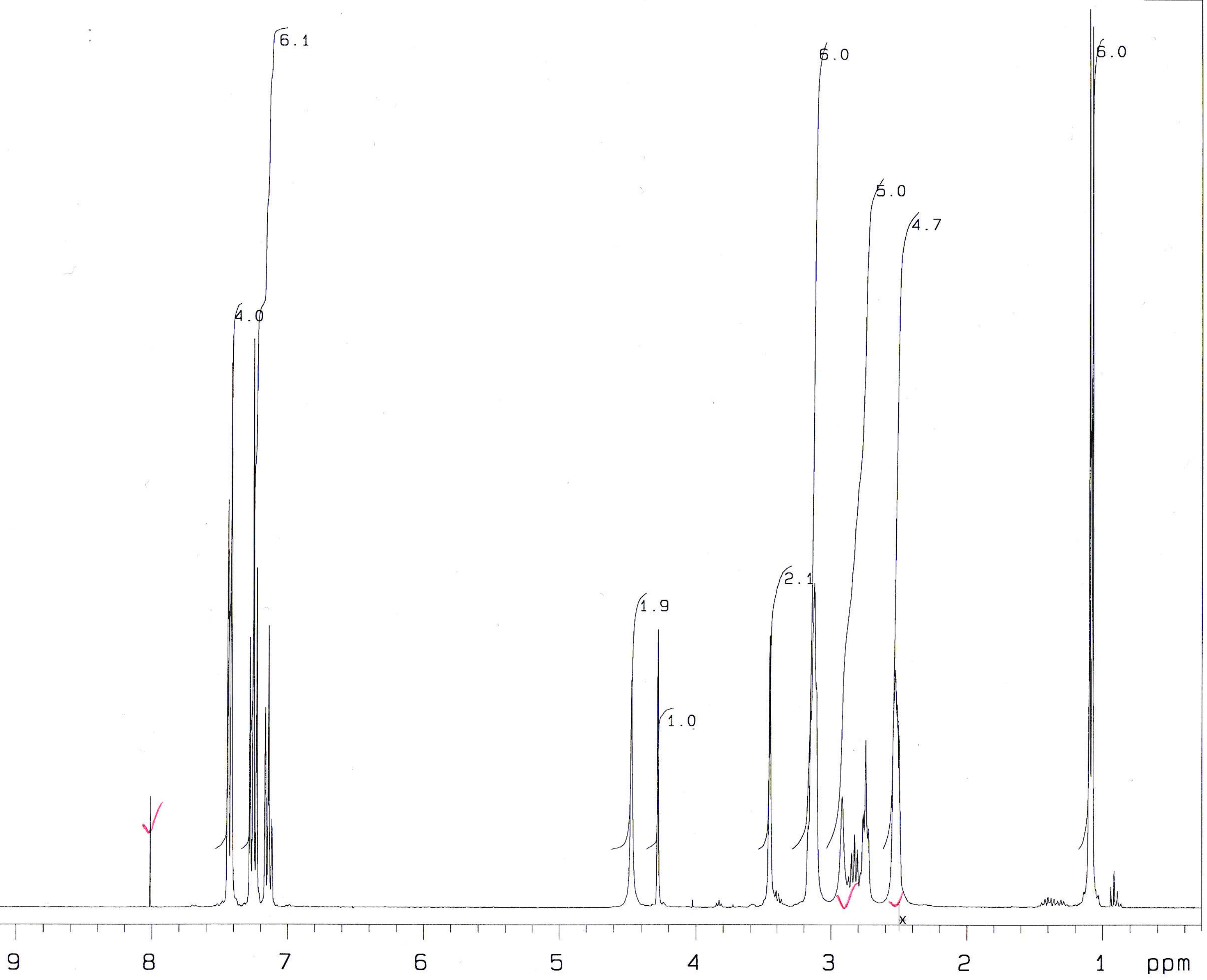

3d

Molecular Structure Research Centre, Yerevan, Armenia, Varian Mercury-300VX

C13 75.465 MHz, nt = 368, np = 19998, temp = 30.0 C, lb = 1.0, solvent = DMSO/CCl4 1/3

Mar 30 2022

ANUSH_TEMA t21-142

T21-142

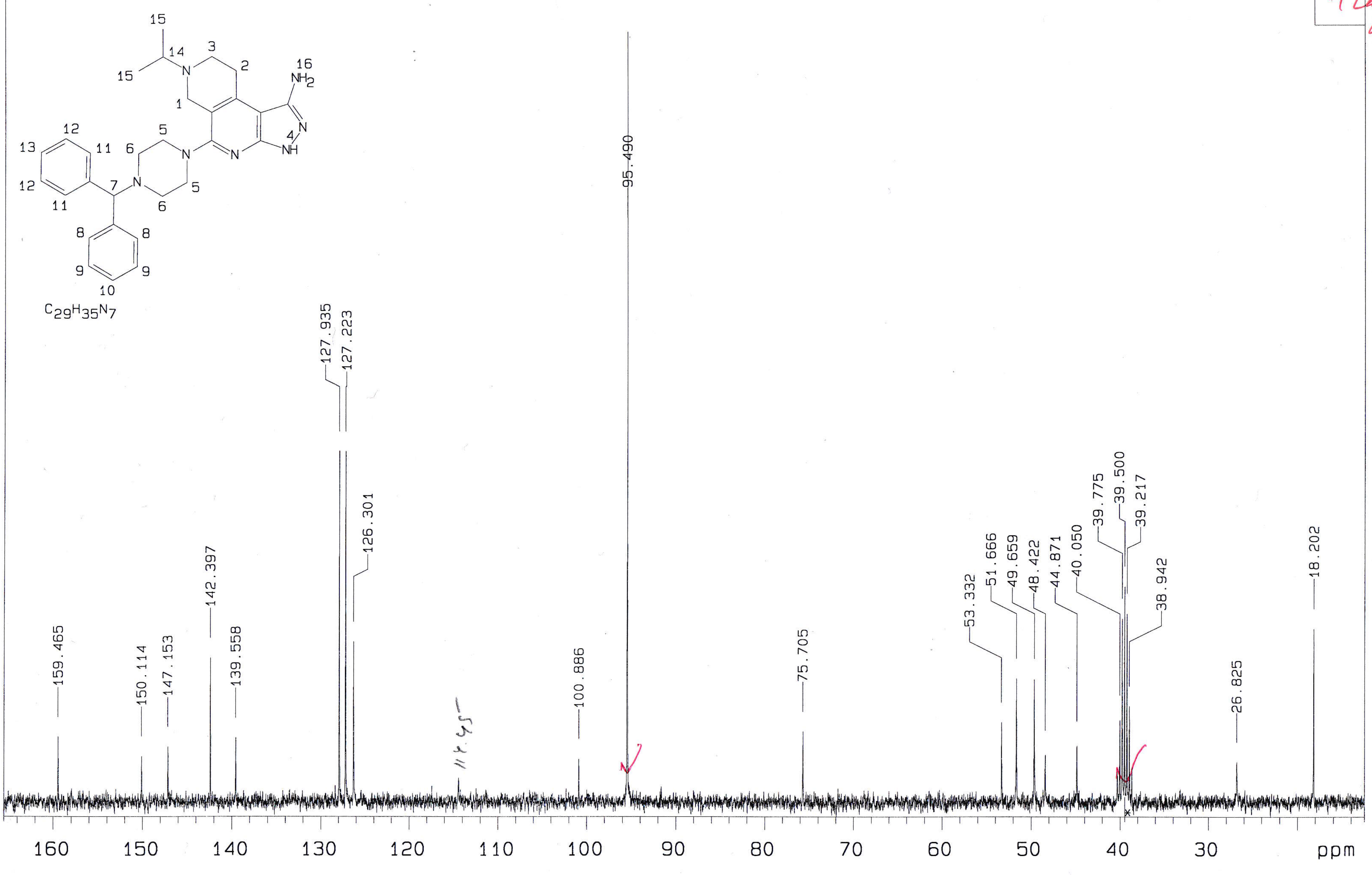

05.03.2025

3d

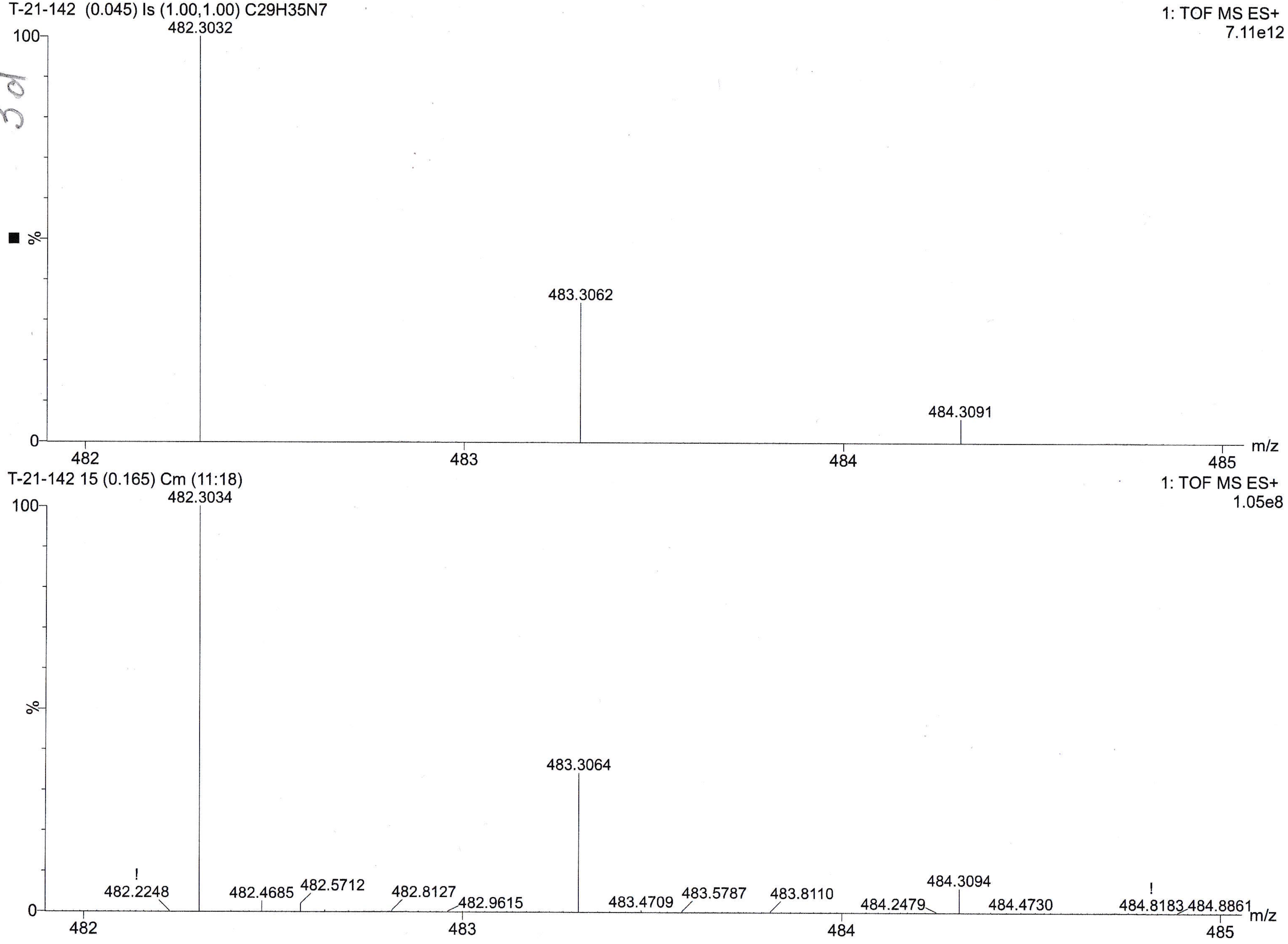

T-21-142 (0.045) Is (1.00,1.00) C29H35N7
1: TOF MS ES+
7.11e12
482.3032
483.3062
484.3091
100
%
0
482
483
484
485
m/z
T-21-142 15 (0.165) Cm (11:18)
1: TOF MS ES+
1.05e8
482.3034
483.3064
484.3094
482.2248
482.4685
482.5712
482.8127
482.9615
483.4709
483.5787
483.8110
484.2479
484.4730
484.8183
484.8861
100
%
0
482
483
484
485
m/z

3e

Molecular Structure Research Centre, Yerevan, Armenia, Varian Mercury-300VX

H1 300.088 MHz, nt=16, np=32000, temp=30.0 C, lb=-0.2, solvent=DMSO/CCl4 1/3

Feb 16 2023

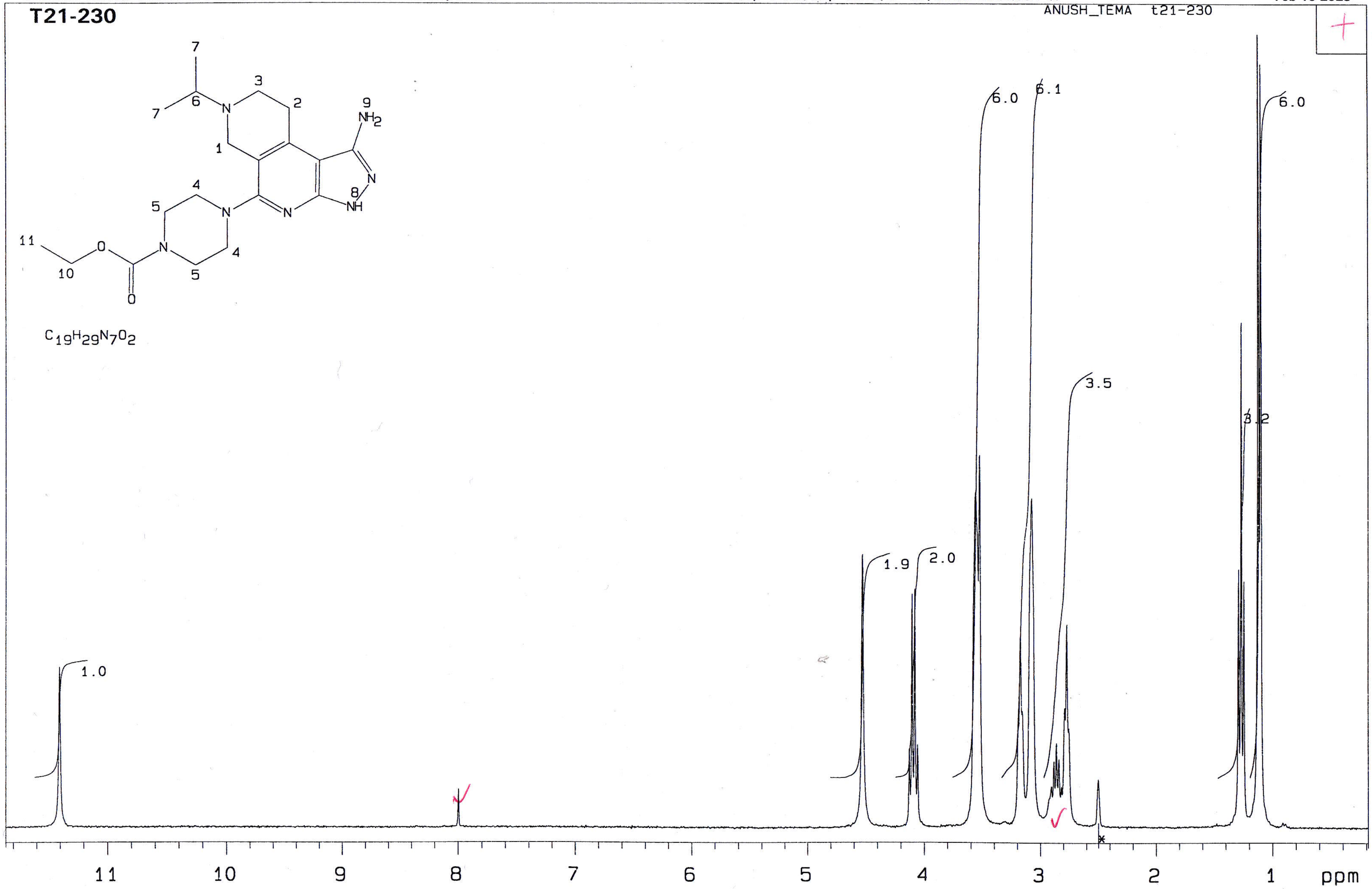

3e

Molecular Structure Research Centre, Yerevan, Armenia, Varian Mercury-300VX

C13 75.465 MHz, nt=304, np=19998, temp=30.0 C, lb=1.0, solvent=DMSO/CCl4 1/3

Feb 16 2023

T21-230

ANUSH_TEMA t21-230

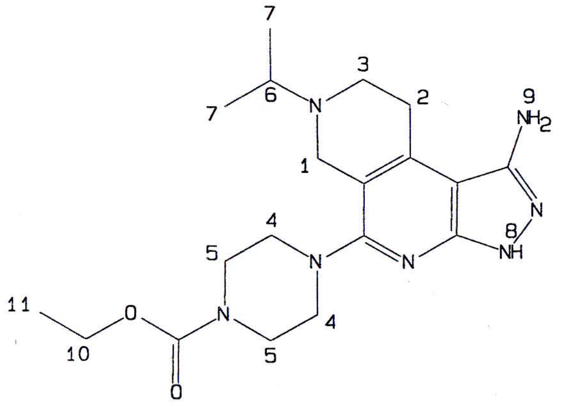

$C_{19}H_{29}N_7O_2$

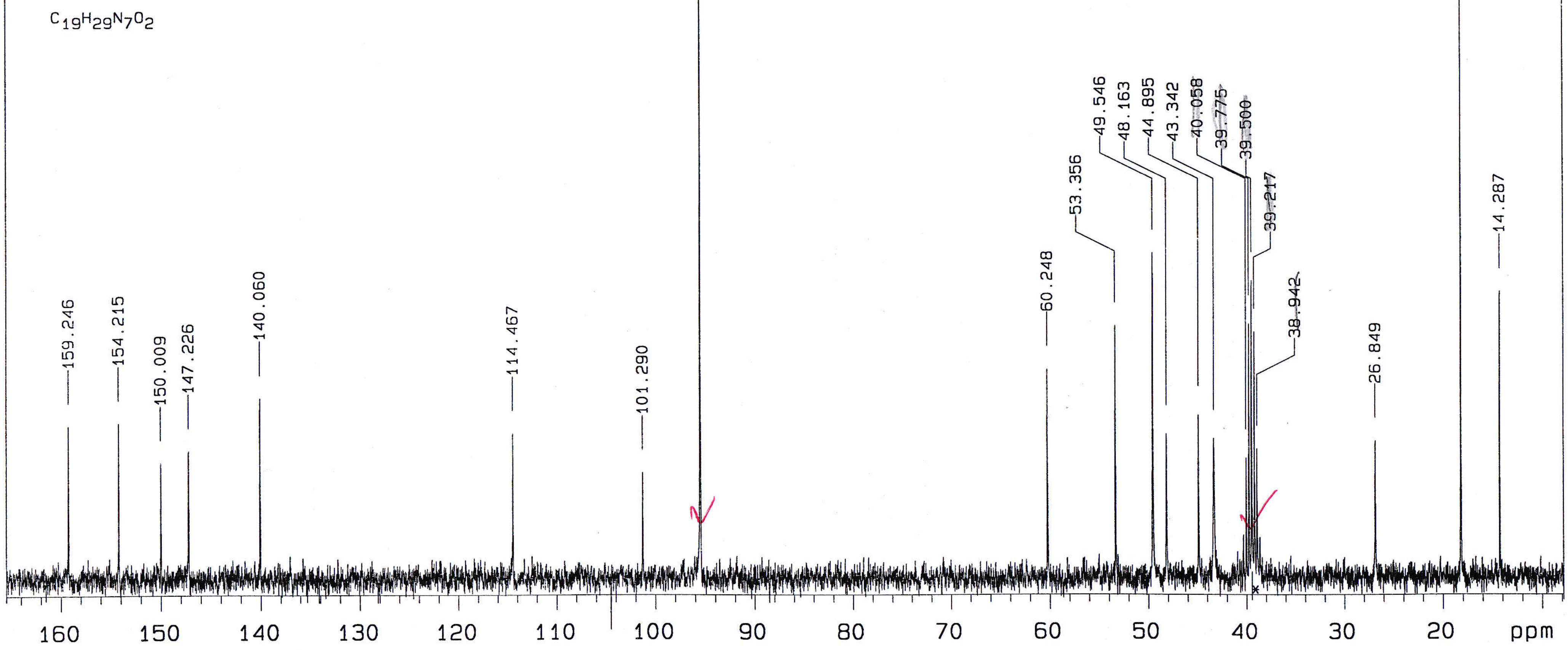

3f

Molecular Structure Research Centre, Yerevan, Armenia, Varian Mercury-300VX

H1 300.088 MHz, nt=16, np=32000, temp=30.0 C, lb=-0.2, solvent=DMSO/CCl4 1/3

Jan 13 2023

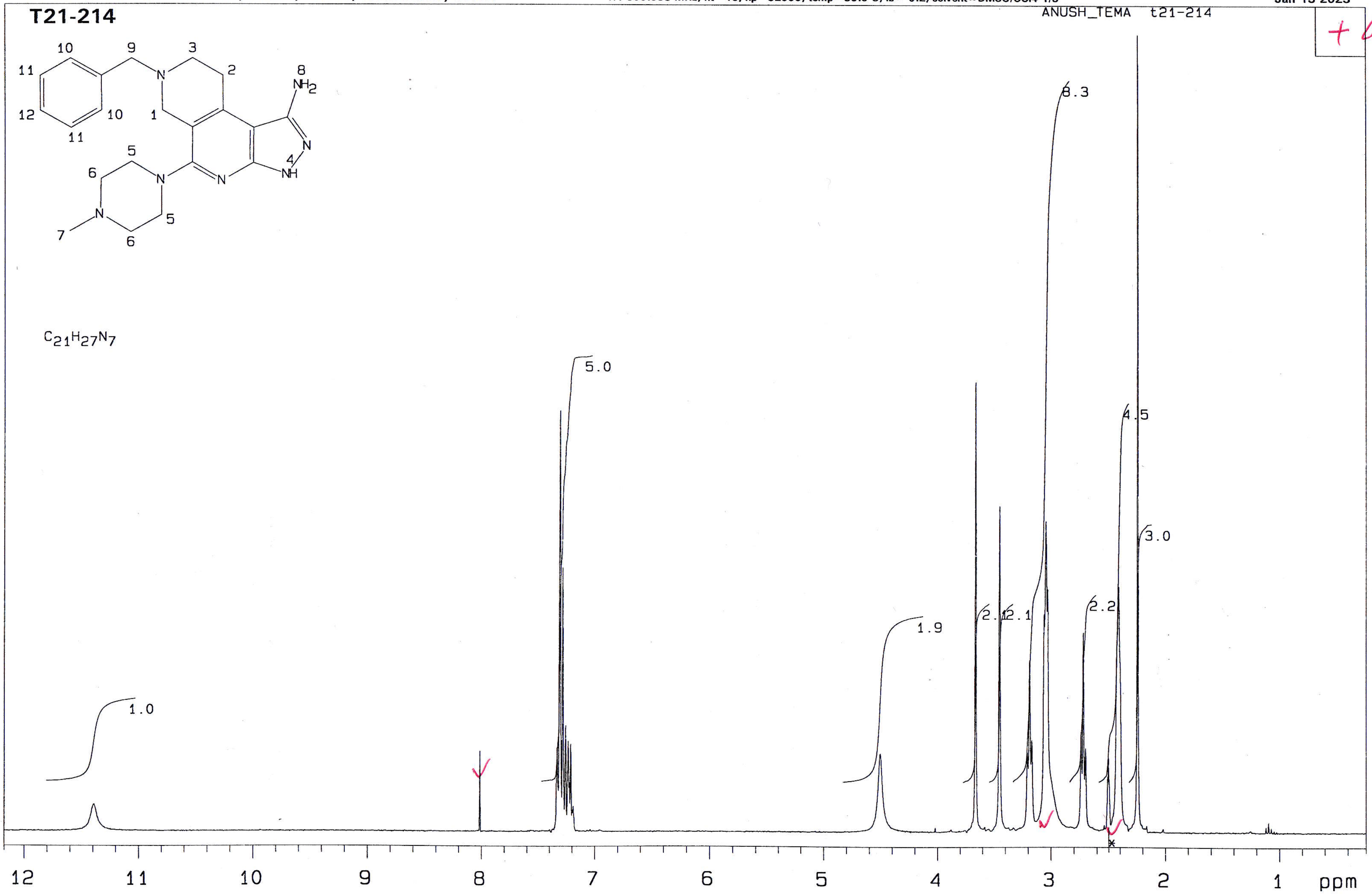

3f

Molecular Structure Research Centre, Yerevan, Armenia, Varian Mercury-300VX

C13 75.465 MHz, nt = 128, np = 19998, temp = 30.0 C, lb = 1.0, solvent = DMSO/CCl4 1/3

Jan 13 2023

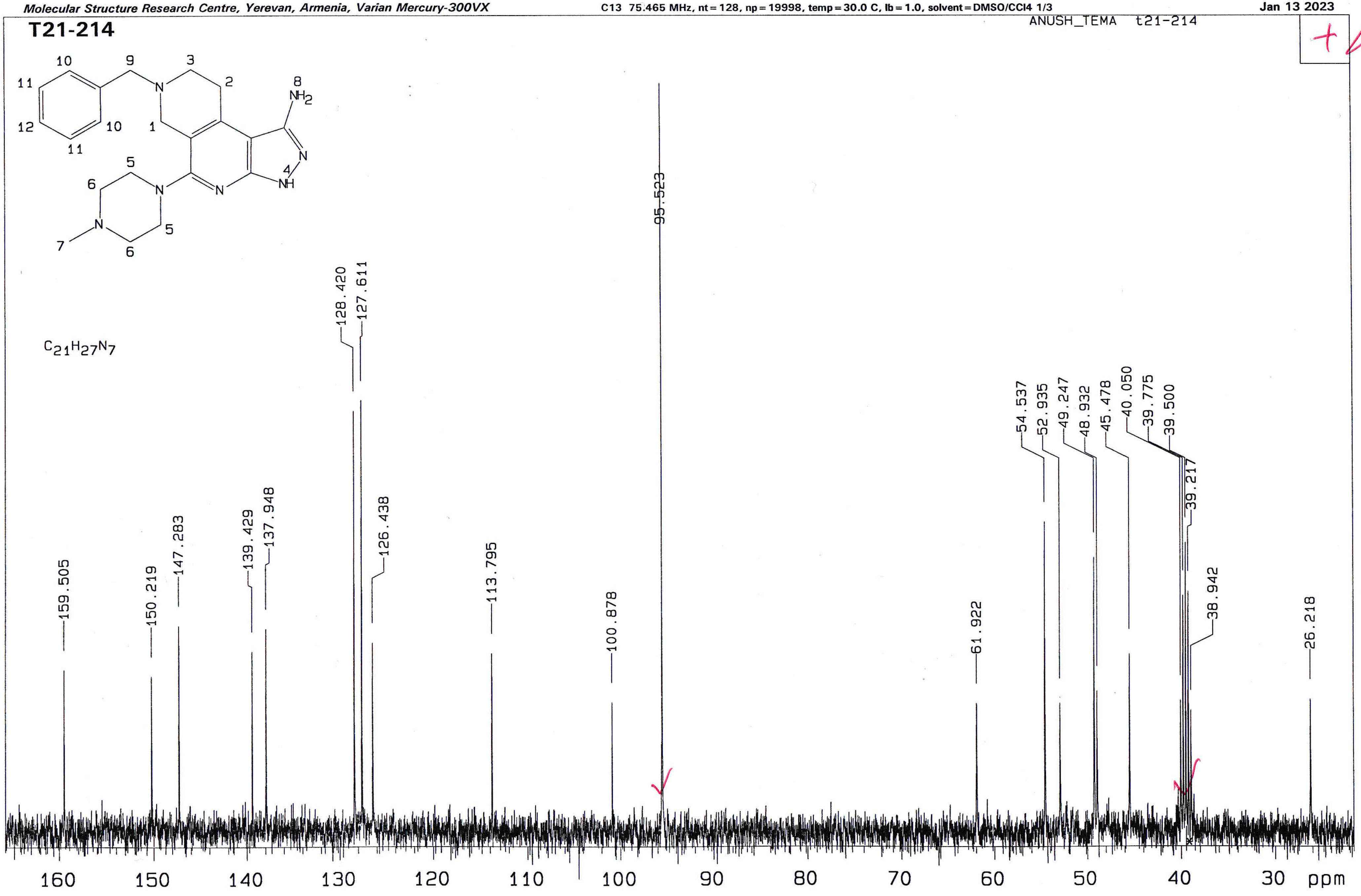

31.01.2025

T21-214 (0.172) Is (1.00,1.00) C21H27N7

3f

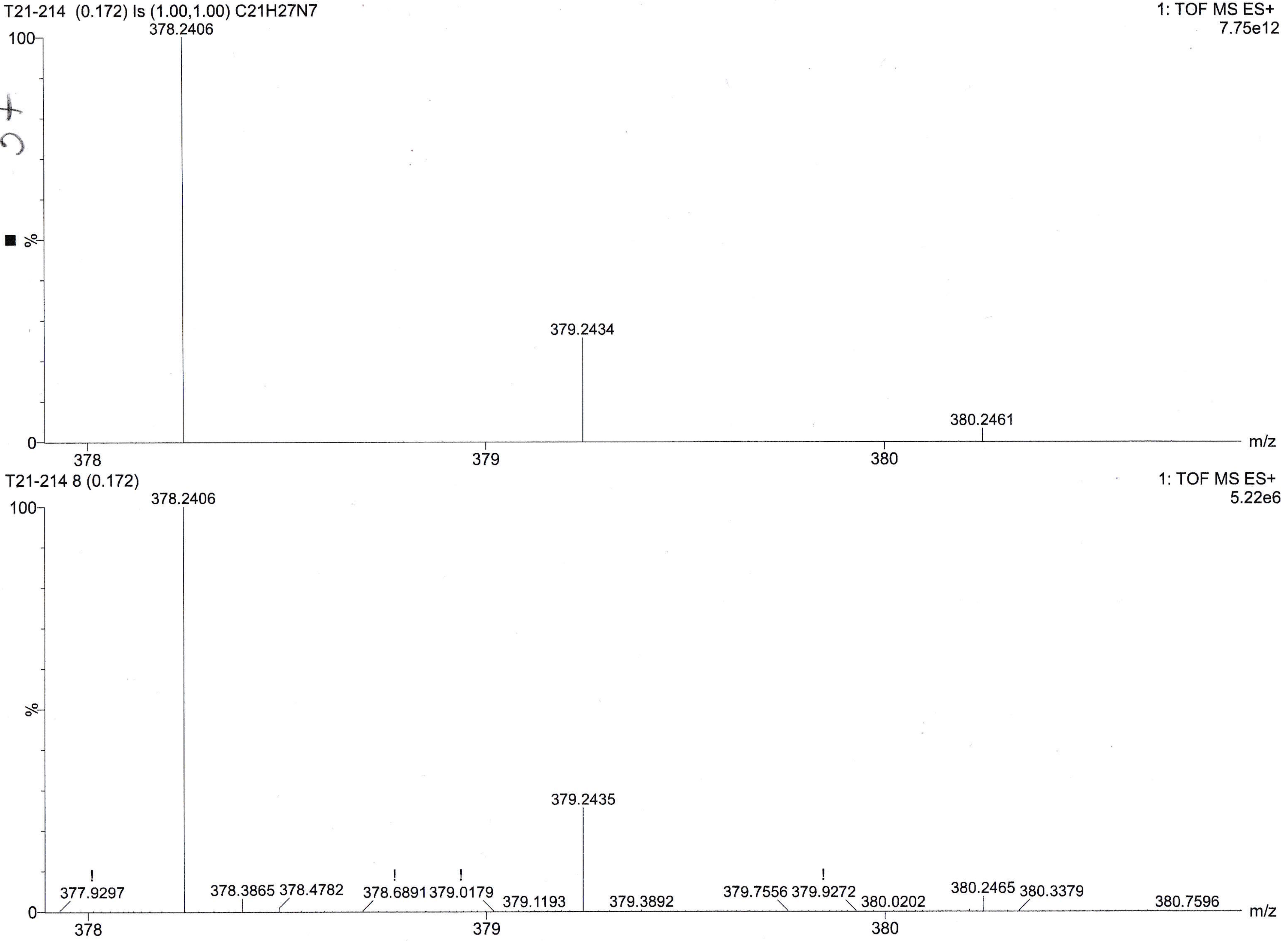

3g

Molecular Structure Research Centre, Yerevan, Armenia, Varian Mercury-300VX

H1 300.088 MHz, nt=16, np=32000, temp=30.0 C, lb=-0.2, solvent=DMSO/CCl4 1/3

Jan 18 2023

ANUSH_TEMA t21-219

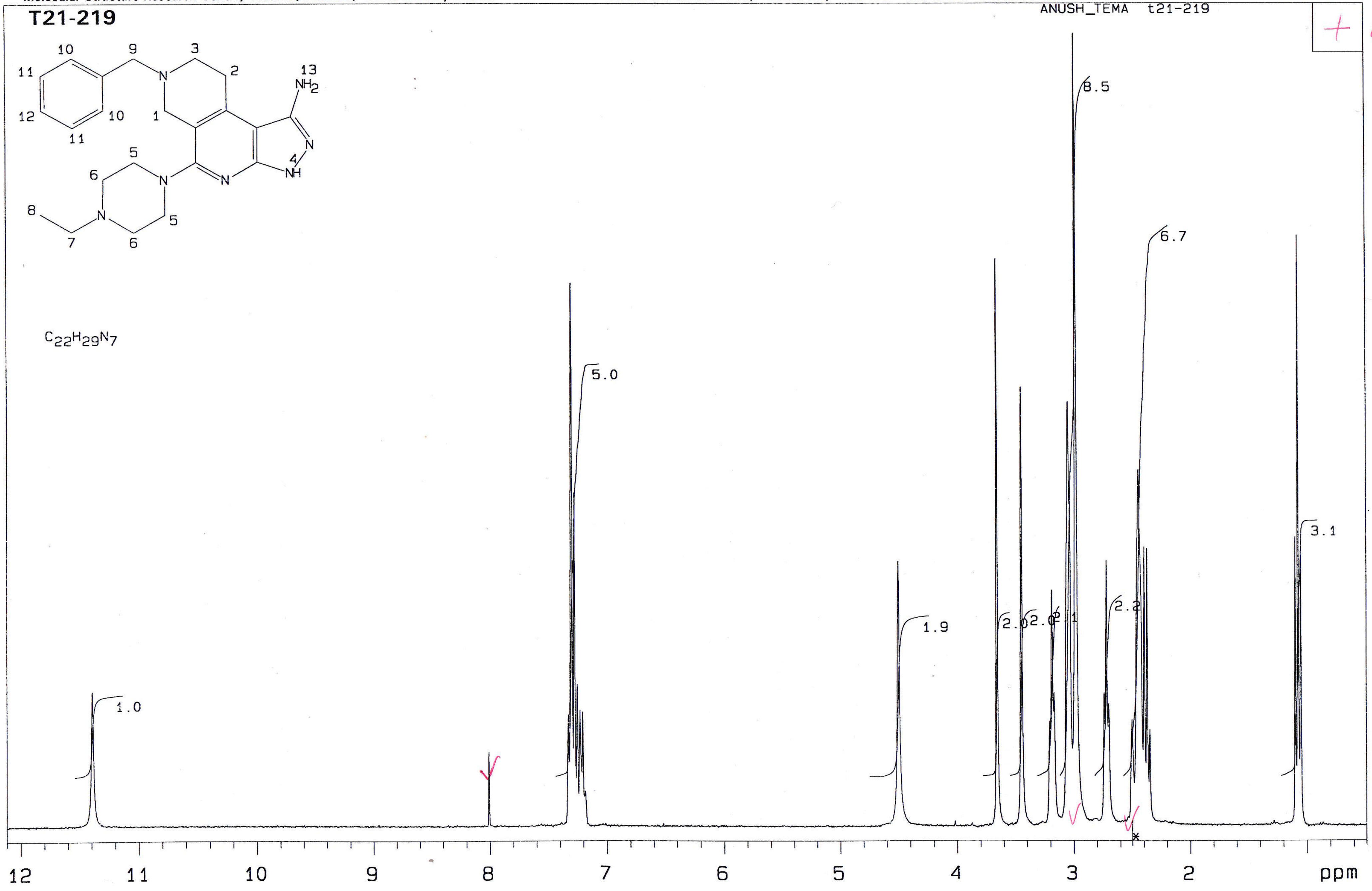

Molecular Structure Research Centre, Yerevan, Armenia, Varian Mercury-300VX

C13 75.465 MHz, nt=352, np=19998, temp=30.0 C, lb=1.0, solvent=DMSO/CCl4 1/3

Jan 18 2023

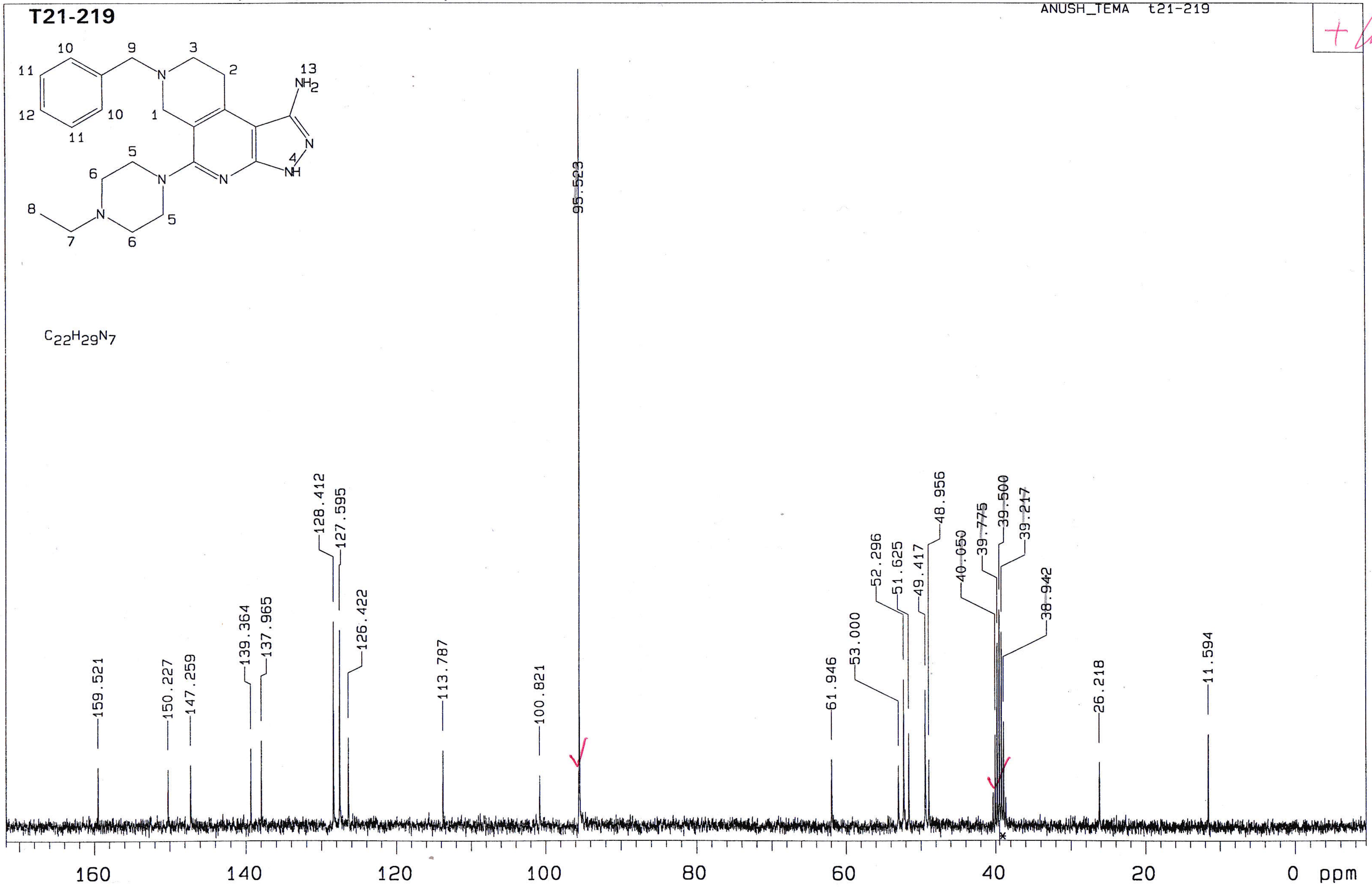

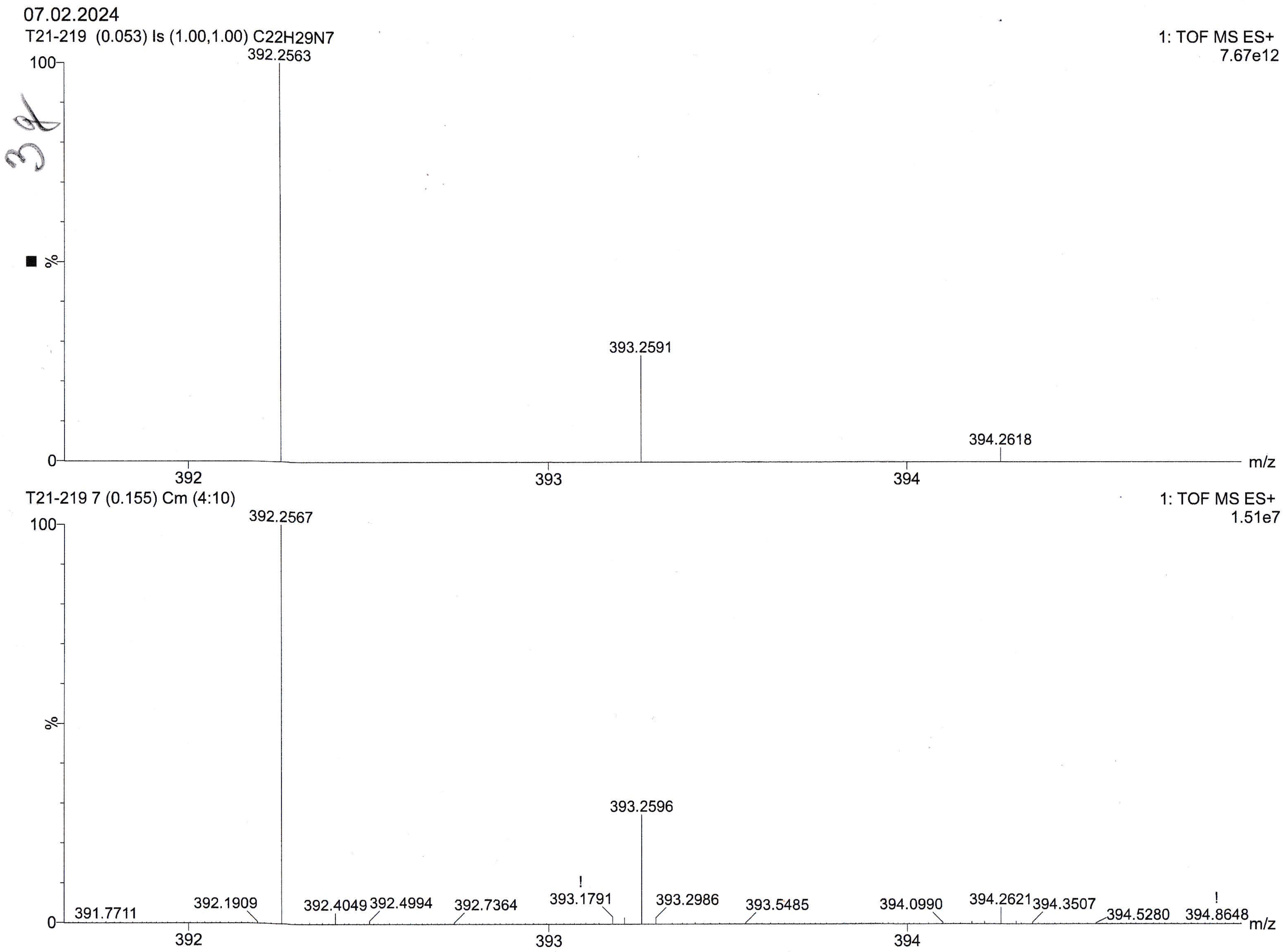

3g
07.02.2024
T21-219 (0.053) Is (1.00,1.00) C22H29N7
1: TOF MS ES+
7.67e12
100
%
0
392.2563
393.2591
394.2618
392
393
394
m/z
T21-219 7 (0.155) Cm (4:10)
1: TOF MS ES+
1.51e7
100
%
0
392.2567
391.7711
392.1909
392.4049
392.4994
392.7364
393.1791
393.2596
393.2986
393.5485
394.0990
394.2621
394.3507
394.5280
394.8648
392
393
394
m/z

3h

Molecular Structure Research Centre, Yerevan, Armenia, Varian Mercury-300VX

H1 300.088 MHz, nt=16, np=32000, temp=30.0 C, lb=-0.2, solvent=DMSO/CCl4 1/3

Jan 17 2023

ANUSH_TEMA t21-220

T21-220

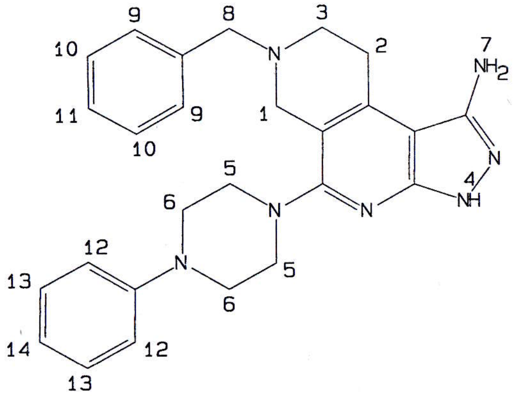

$C_{26}H_{29}N_7$

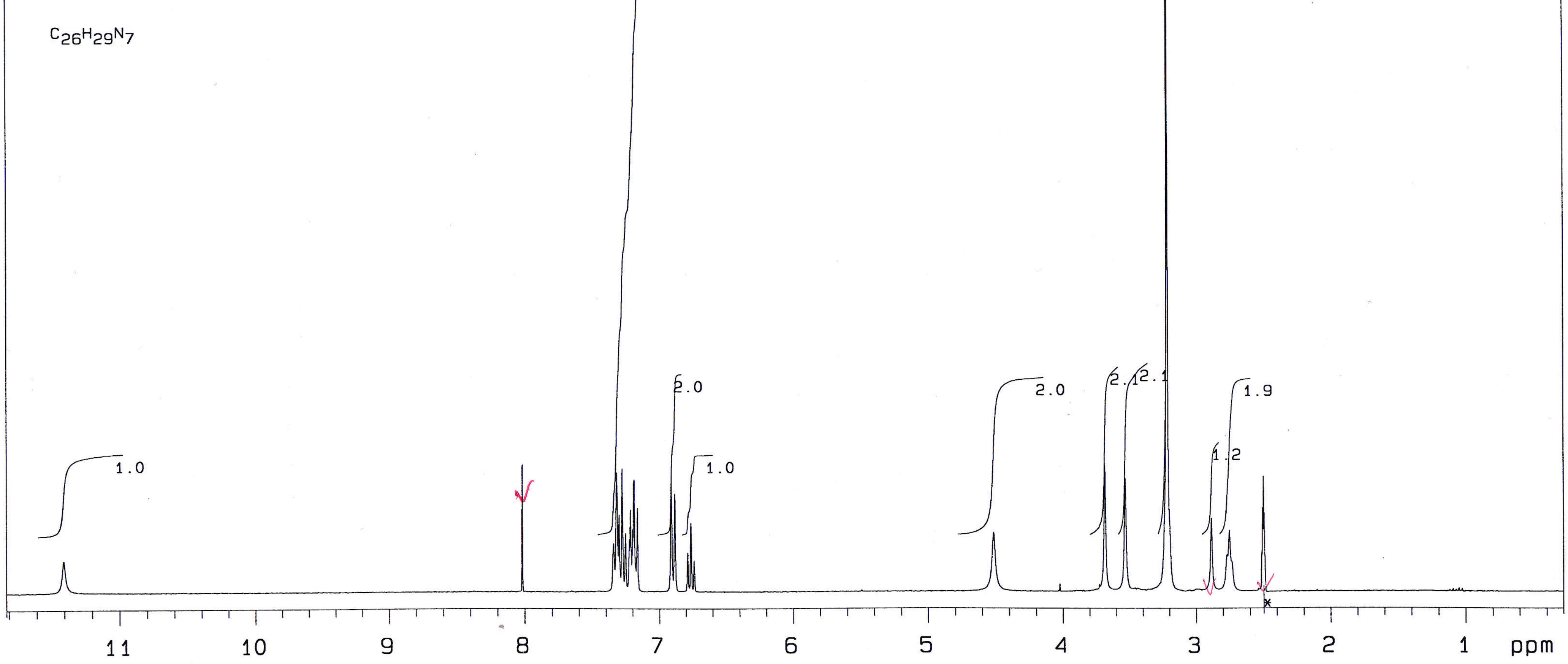

3h

Molecular Structure Research Centre, Yerevan, Armenia, Varian Mercury-300VX

C13 75.465 MHz, nt=256, np=19998, temp=30.0 C, lb=1.0, solvent=DMSO/CCl4 1/3

Jan 17 2023

ANUSH_TEMA t21-220

T21-220

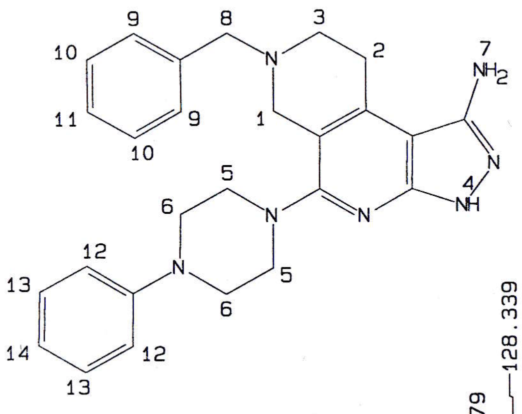

$C_{26}H_{29}N_7$

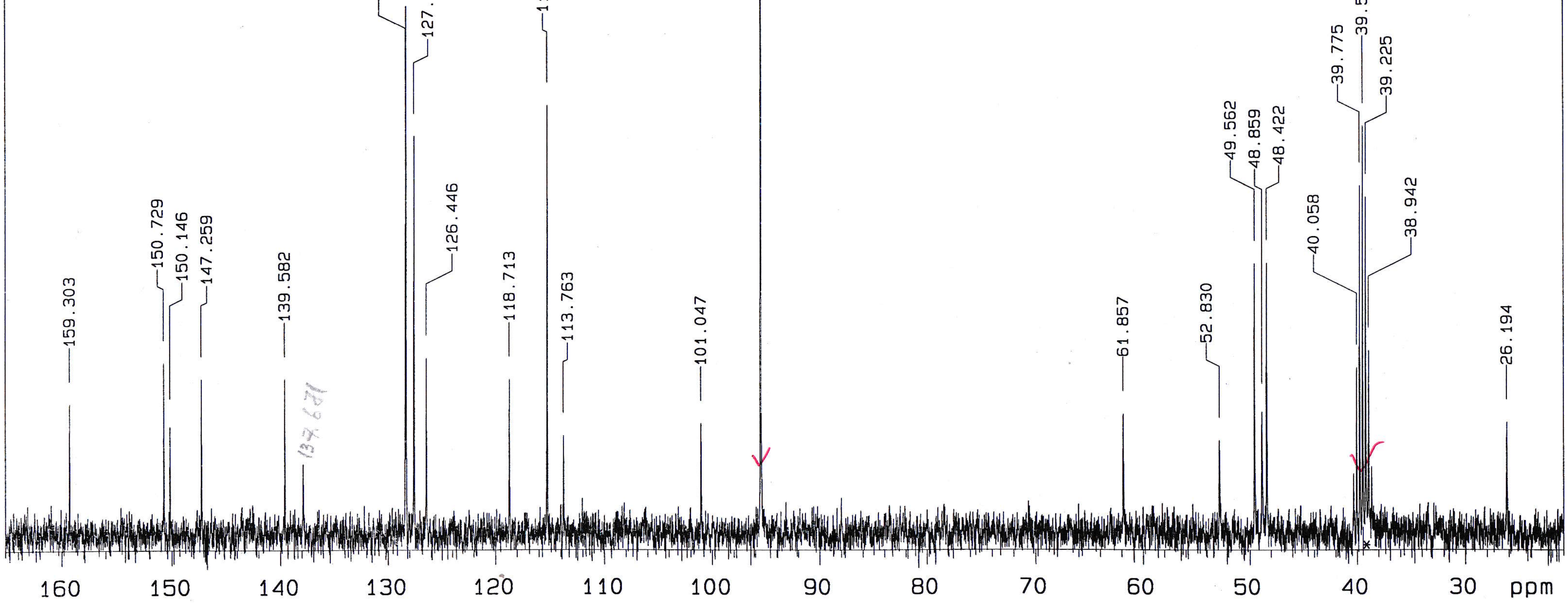

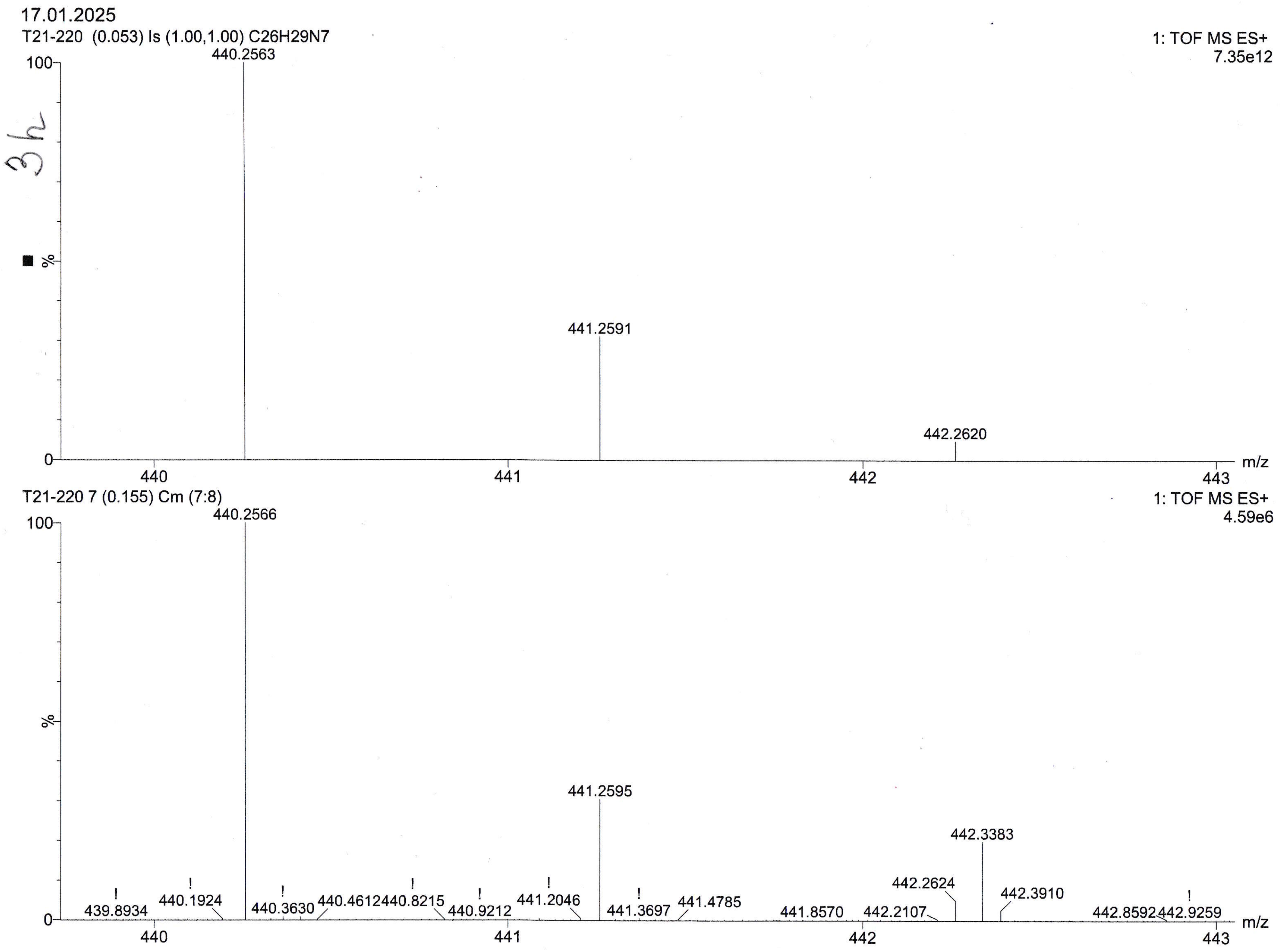
17.01.2025
3h
T21-220 (0.053) Is (1.00,1.00) C26H29N7
1: TOF MS ES+
7.35e12
440.2563
441.2591
442.2620
100
%
0
440
441
442
443
m/z
T21-220 7 (0.155) Cm (7:8)
1: TOF MS ES+
4.59e6
440.2566
441.2595
442.3383
442.2624
442.3910
439.8934
440.1924
440.3630
440.4612
440.8215
440.9212
441.2046
441.3697
441.4785
441.8570
442.2107
442.8592
442.9259
100
%
0
440
441
442
443
m/z

3i

Molecular Structure Research Centre, Yerevan, Armenia, Varian Mercury-300VX

H1 300.088 MHz, nt = 16, np = 32000, temp = 30.0 C, lb = -0.2, solvent = DMSO/CCl4 1/3

Mar 2 2023

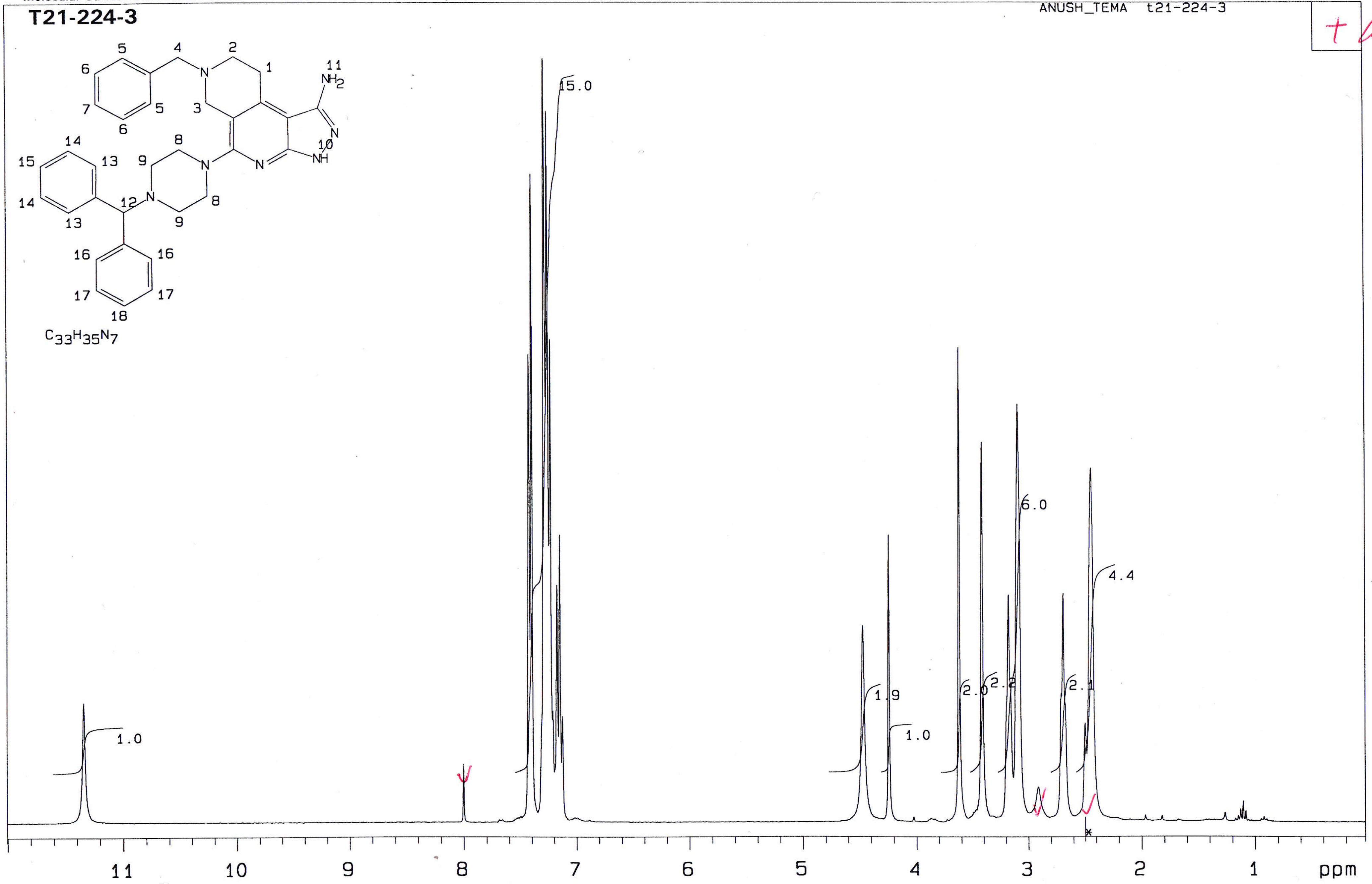

3i

Molecular Structure Research Centre, Yerevan, Armenia, Varian Mercury-300VX

C13 75.465 MHz, nt=512, np=19998, temp=30.0 C, lb=2.0, solvent=DMSO/CCl4 1/3

Mar 2 2023

ANUSH_TEMA t21-224-3

T21-224-3

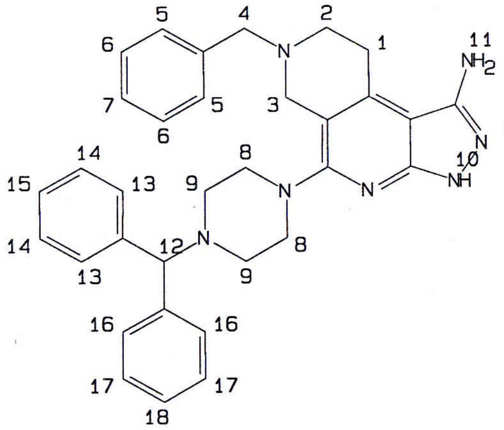

$C_{33}H_{35}N_7$

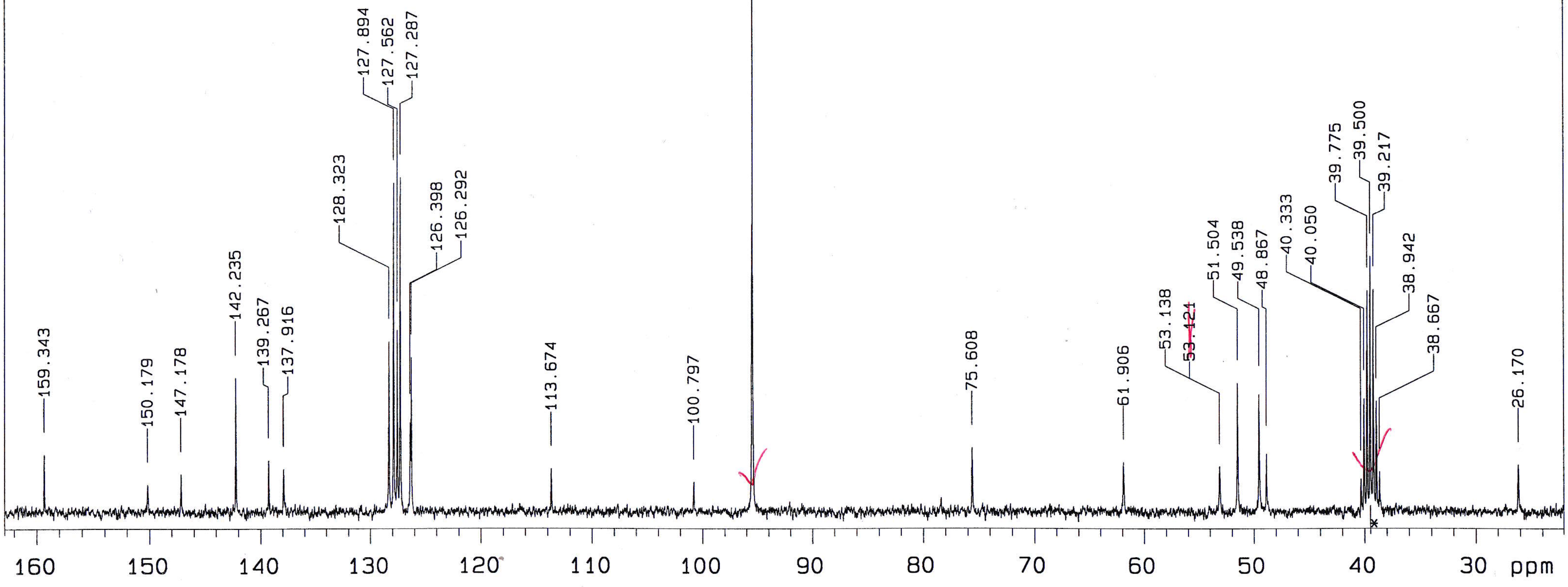

3i

05.03.2025

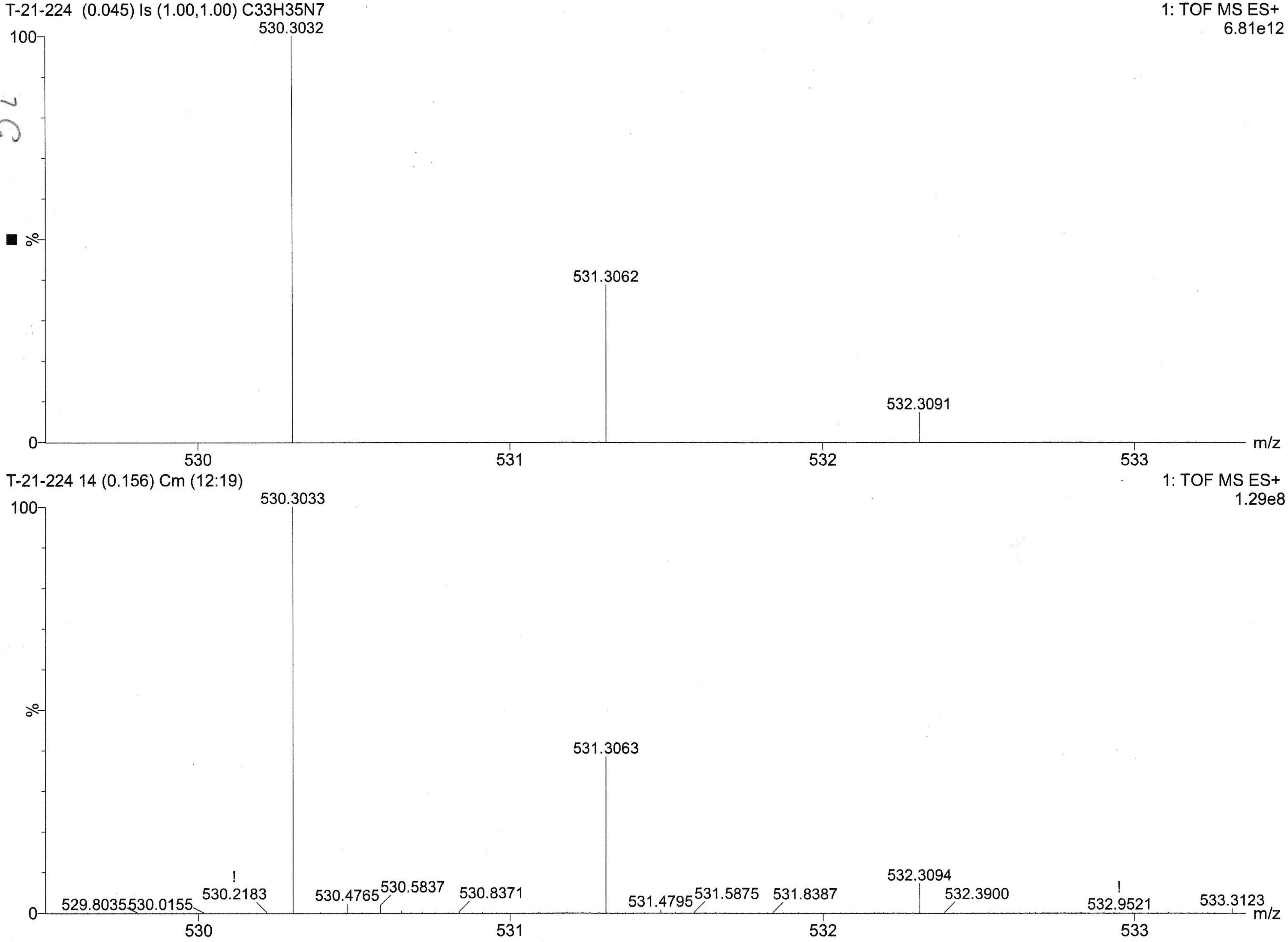

T-21-224 (0.045) Is (1.00,1.00) C33H35N7
1: TOF MS ES+
6.81e12
100
%
0
530.3032
531.3062
532.3091
530
531
532
533
m/z
T-21-224 14 (0.156) Cm (12:19)
1: TOF MS ES+
1.29e8
530.3033
531.3063
529.8035
530.0155
530.2183
530.4765
530.5837
530.8371
531.4795
531.5875
531.8387
532.3094
532.3900
532.9521
533.3123

3j

Molecular Structure Research Centre, Yerevan, Armenia, Varian Mercury-300VX

H1 300.088 MHz, nt=16, np=32000, temp=30.0 C, lb=-0.2, solvent=DMSO/CCl4 1/3

Jan 25 2023

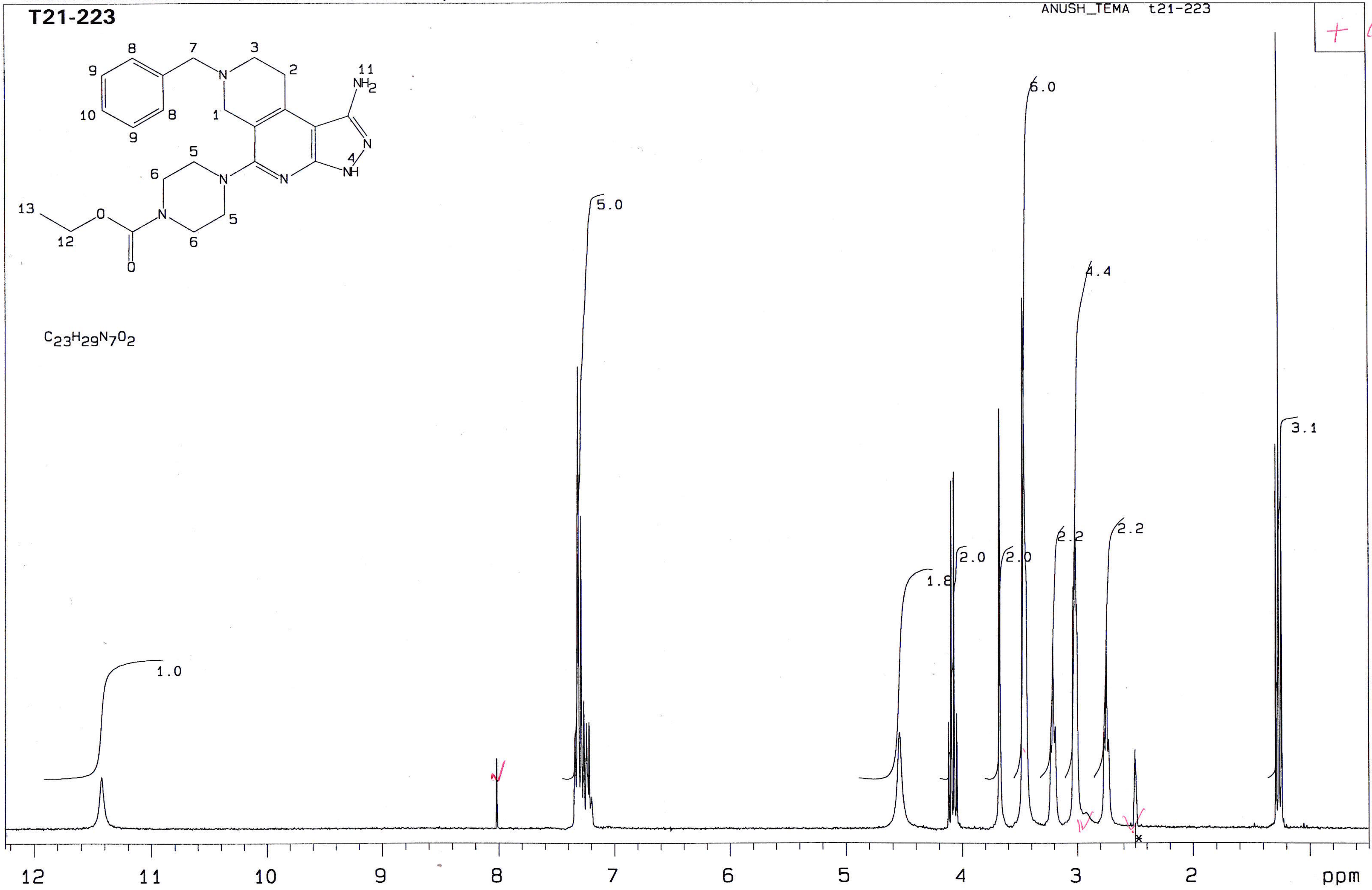

3j

Molecular Structure Research Centre, Yerevan, Armenia, Varian Mercury-300VX

C13 75.465 MHz, nt=352, np=19998, temp=30.0 C, lb=1.0, solvent=DMSO/CCl4 1/3

Jan 25 2023

ANUSH_TEMA t21-223

T21-223

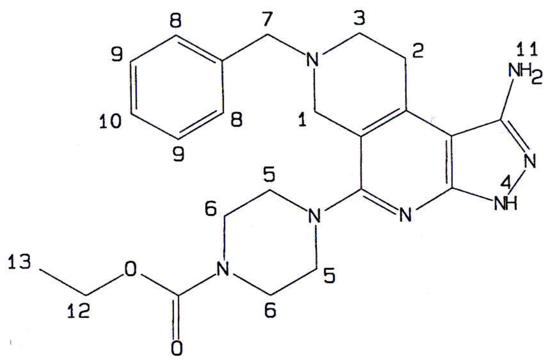

$C_{23}H_{29}N_7O_2$

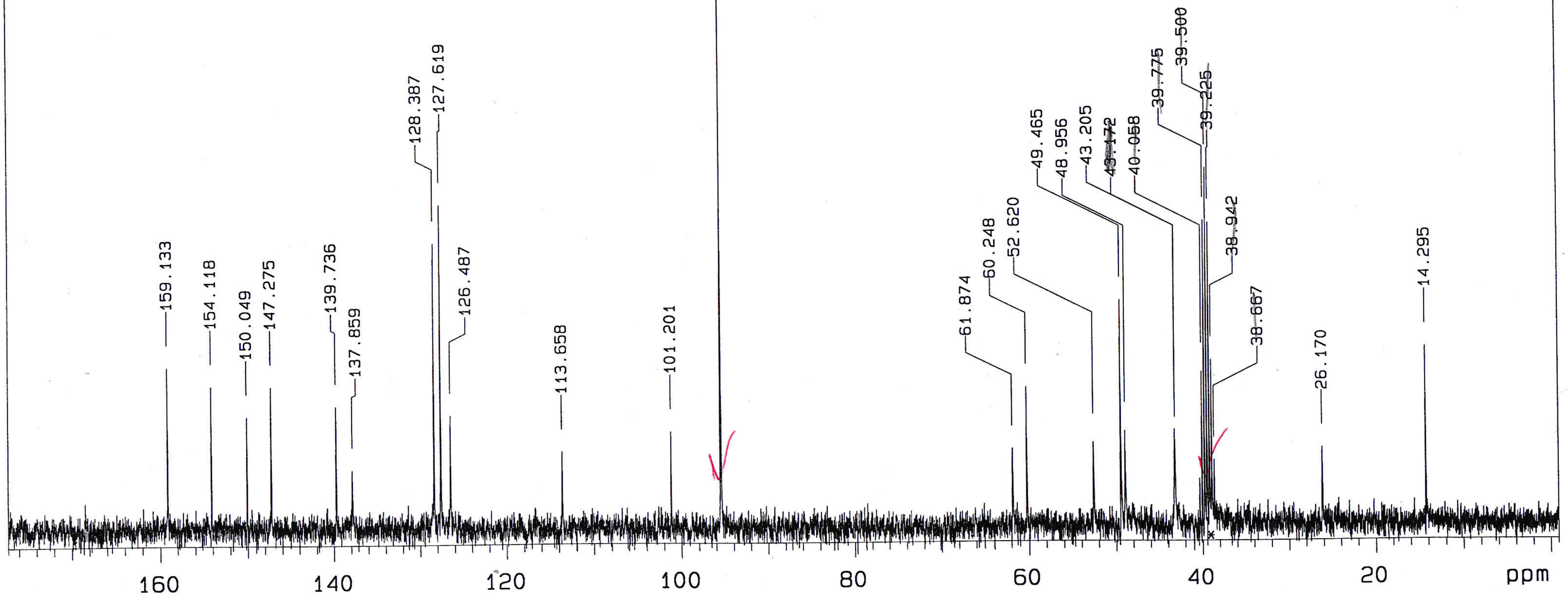

4a

Molecular Structure Research Centre, Yerevan, Armenia, Varian Mercury-300VX

H1 300.088 MHz, nt = 16, np = 32000, temp = 30.0 C, lb = 1.0, solvent = DMSO/CCl4 1/3

Apr 12 2022

ANUSH_TEMA t21-139

T21-139

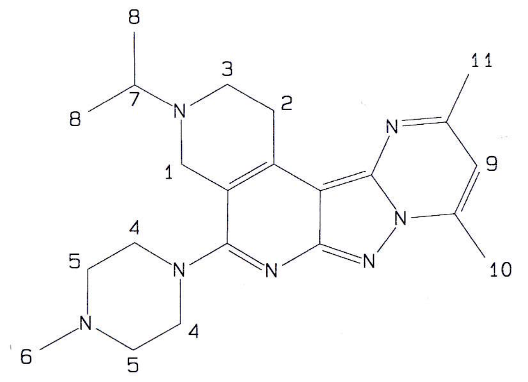

$C_{22}H_{31}N_7$

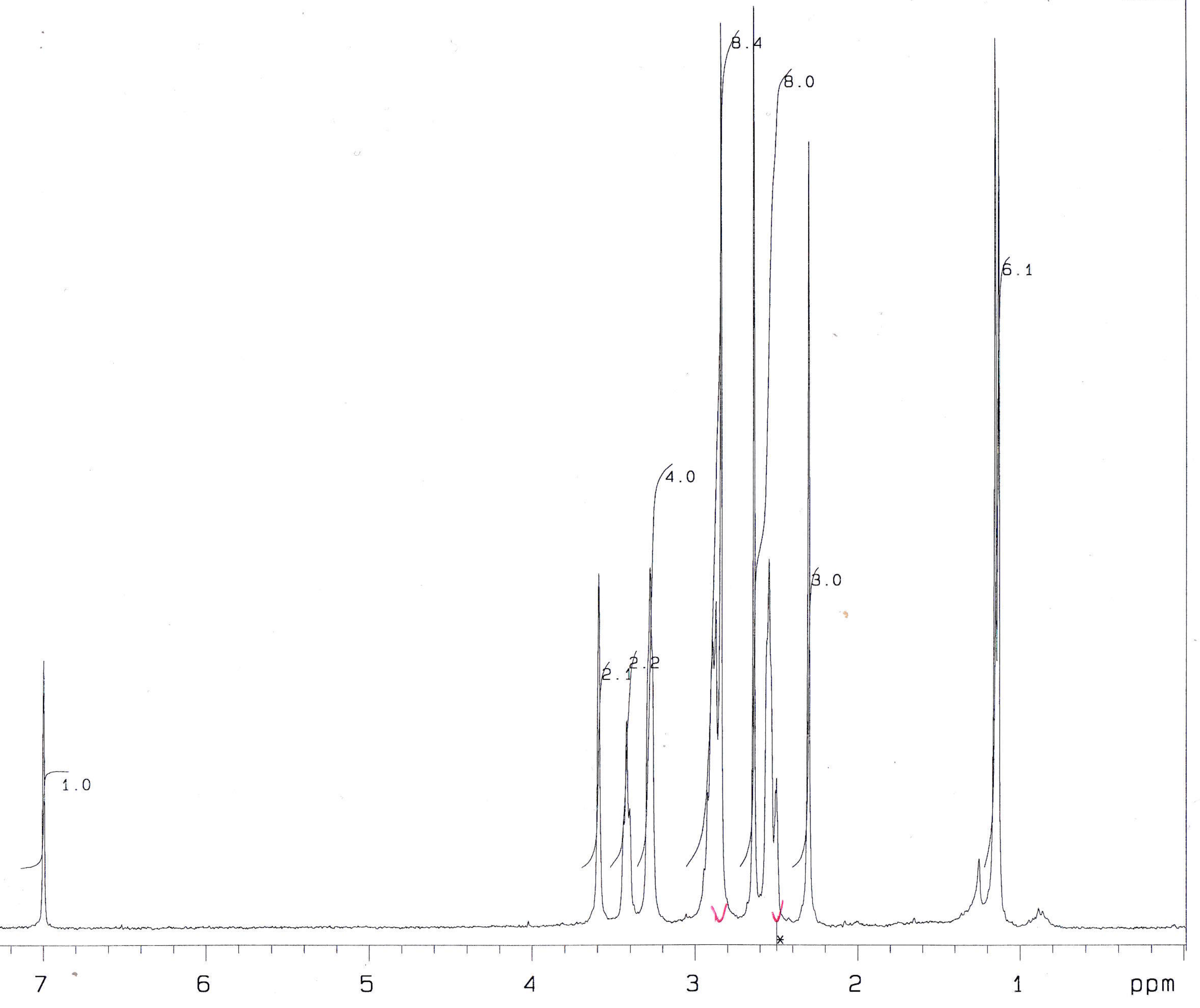

4a

Molecular Structure Research Centre, Yerevan, Armenia, Varian Mercury-300VX

C13 75.465 MHz, nt=640, np=19998, temp=30.0 C, lb=1.0, solvent=DMSO/CCl4 1/3

Apr 12 2022

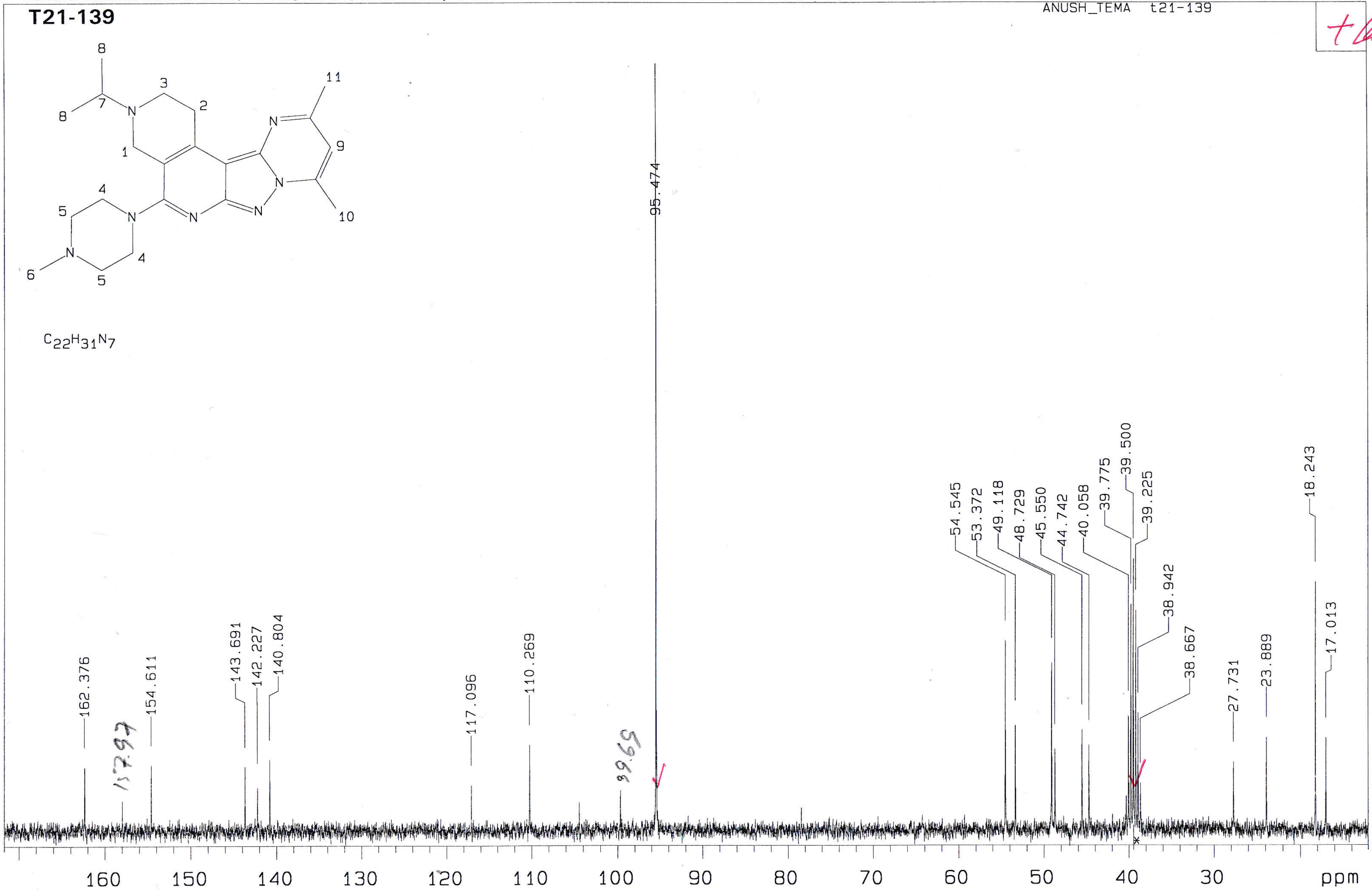

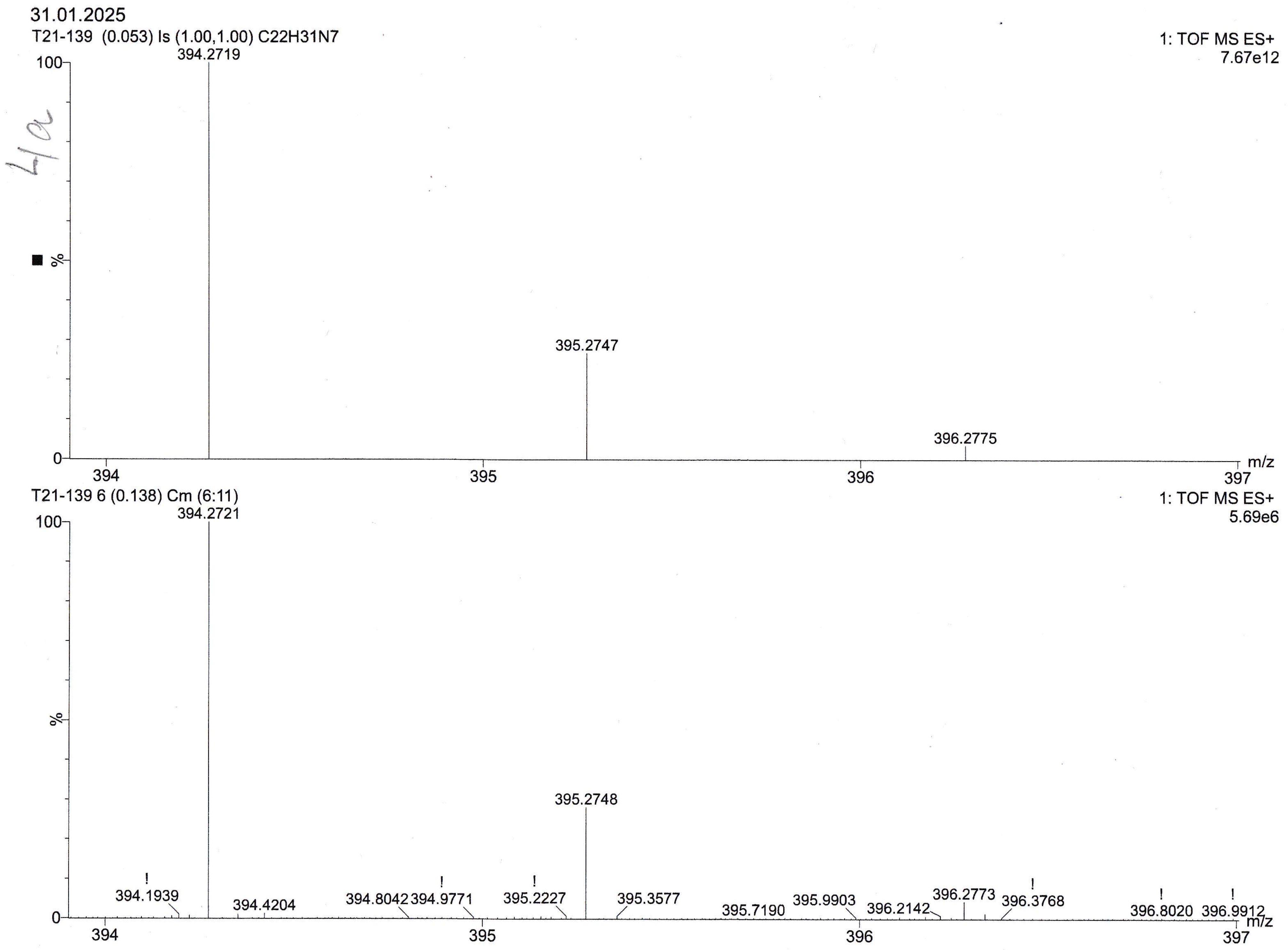

4a
31.01.2025
T21-139 (0.053) Is (1.00,1.00) C22H31N7
1: TOF MS ES+
7.67e12
100
%
0
394.2719
395.2747
396.2775
394
395
396
397
m/z
T21-139 6 (0.138) Cm (6:11)
1: TOF MS ES+
5.69e6
100
%
0
394.2721
394.1939
394.4204
394.8042
394.9771
395.2227
395.2748
395.3577
395.7190
395.9903
396.2142
396.2773
396.3768
396.8020
396.9912
394
395
396
397
m/z

4b

Molecular Structure Research Centre, Yerevan, Armenia, Varian Mercury-300VX

H1 300.088 MHz, nt = 16, np = 32000, temp = 30.0 C, lb = -0.2, solvent = DMSO/CCl4 1/3

Apr 19 2022

ANUSH_TEMA t21-140

T21-140

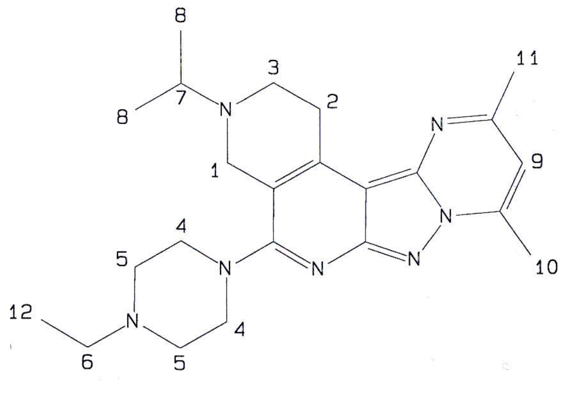

$C_{23}H_{33}N_7$

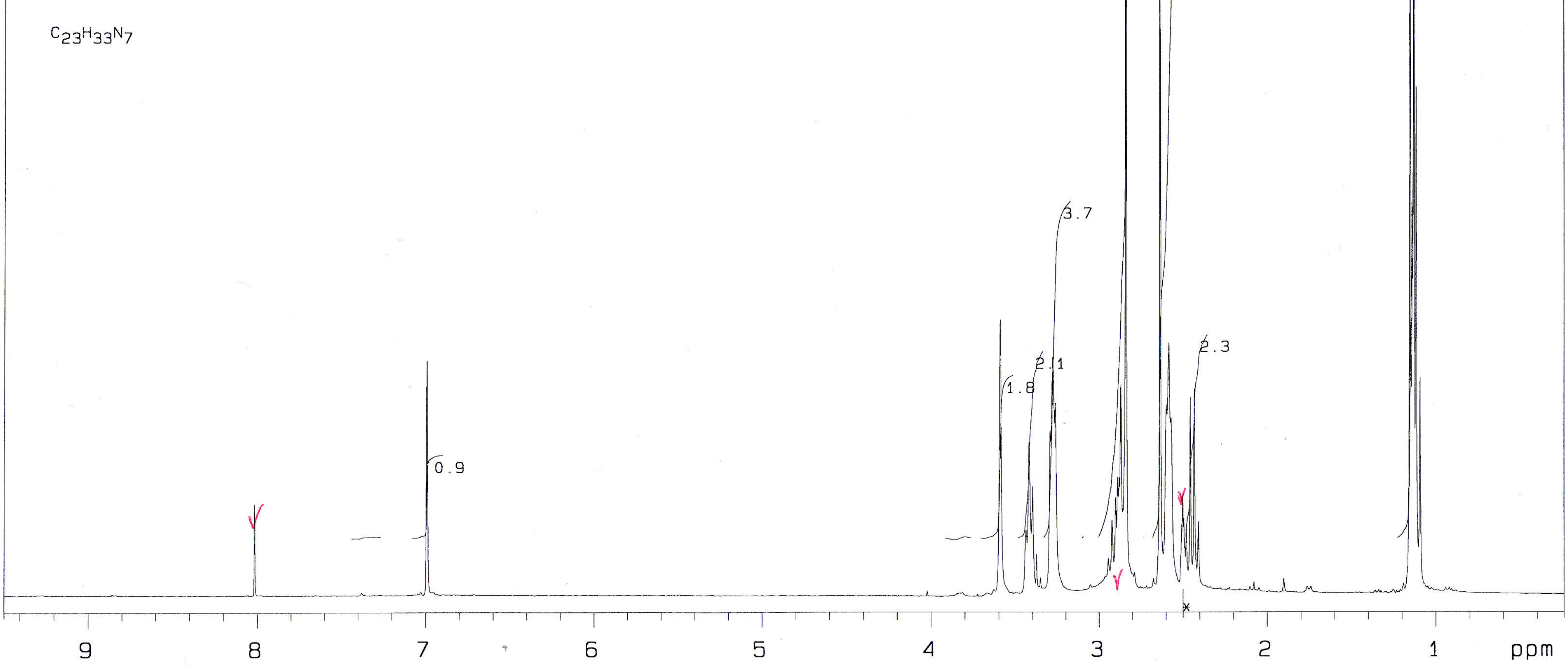

46

Molecular Structure Research Centre, Yerevan, Armenia, Varian Mercury-300VX

C13 75.465 MHz, nt = 624, np = 19998, temp = 30.0 C, lb = 1.0, solvent = DMSO/CCl4 1/3

Apr 19 2022

ANUSH_TEMA t21-140

T21-140

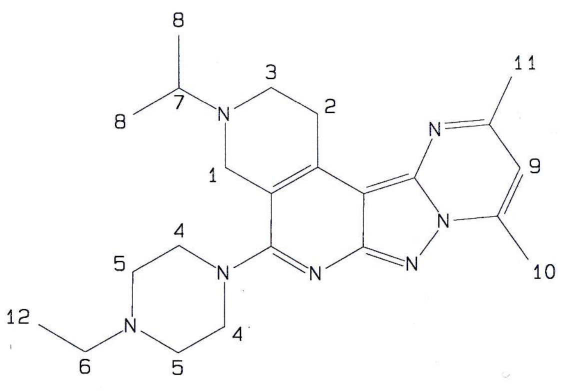

$C_{23}H_{33}N_7$

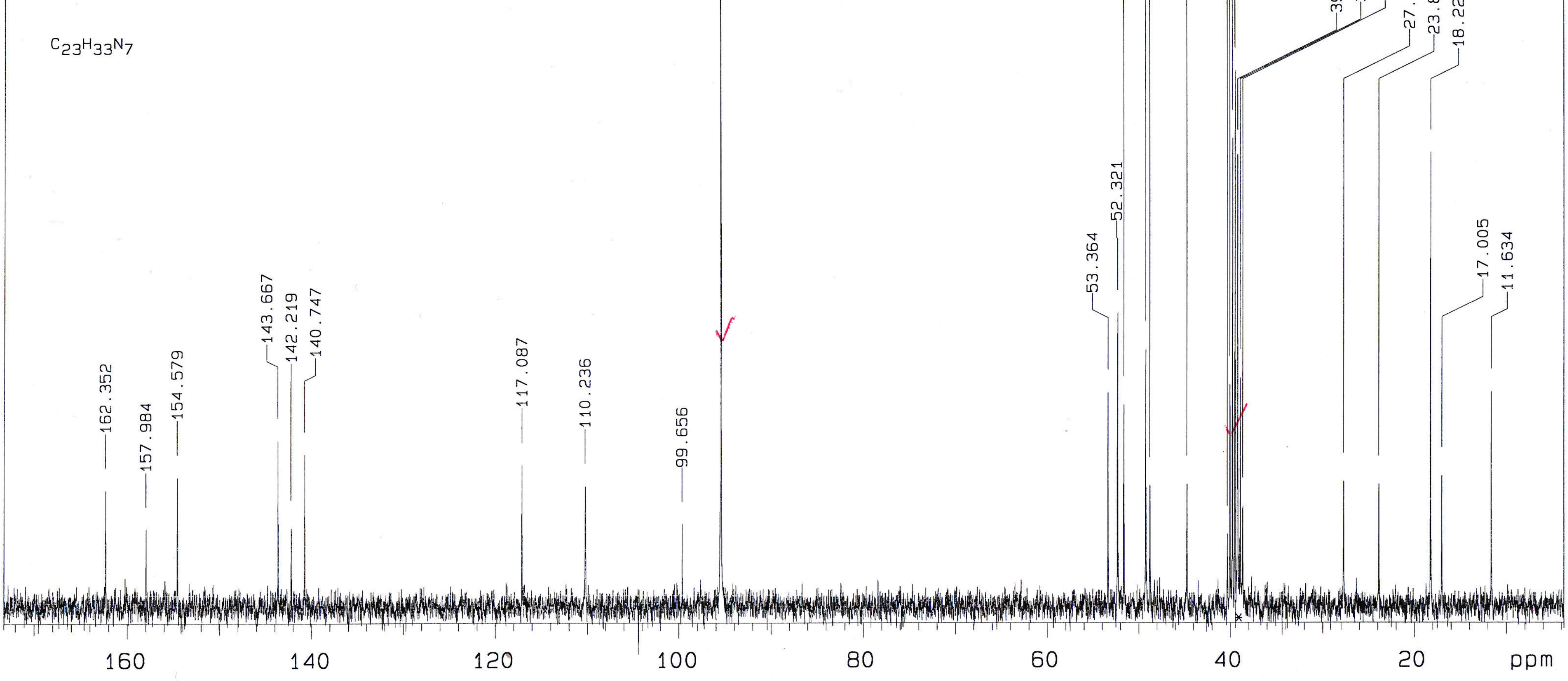

4c

Molecular Structure Research Centre, Yerevan, Armenia, Varian Mercury-300VX

H1 300.088 MHz, nt = 16, np = 32000, temp = 30.0 C, lb = -0.2, solvent = DMSO/CCl4 1/3

Apr 3 2023

ANUSH_TEMA t21-135-4

T21-135-4

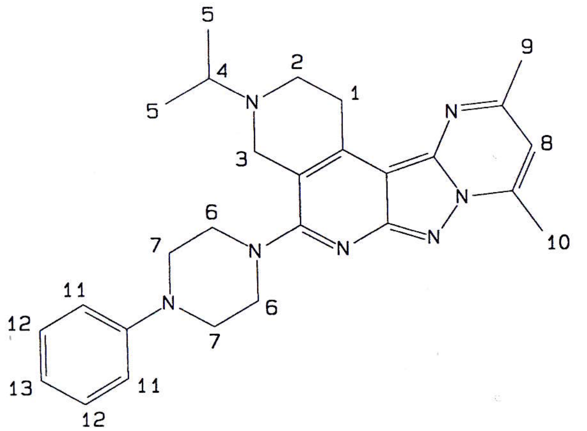

$C_{27}H_{33}N_7$

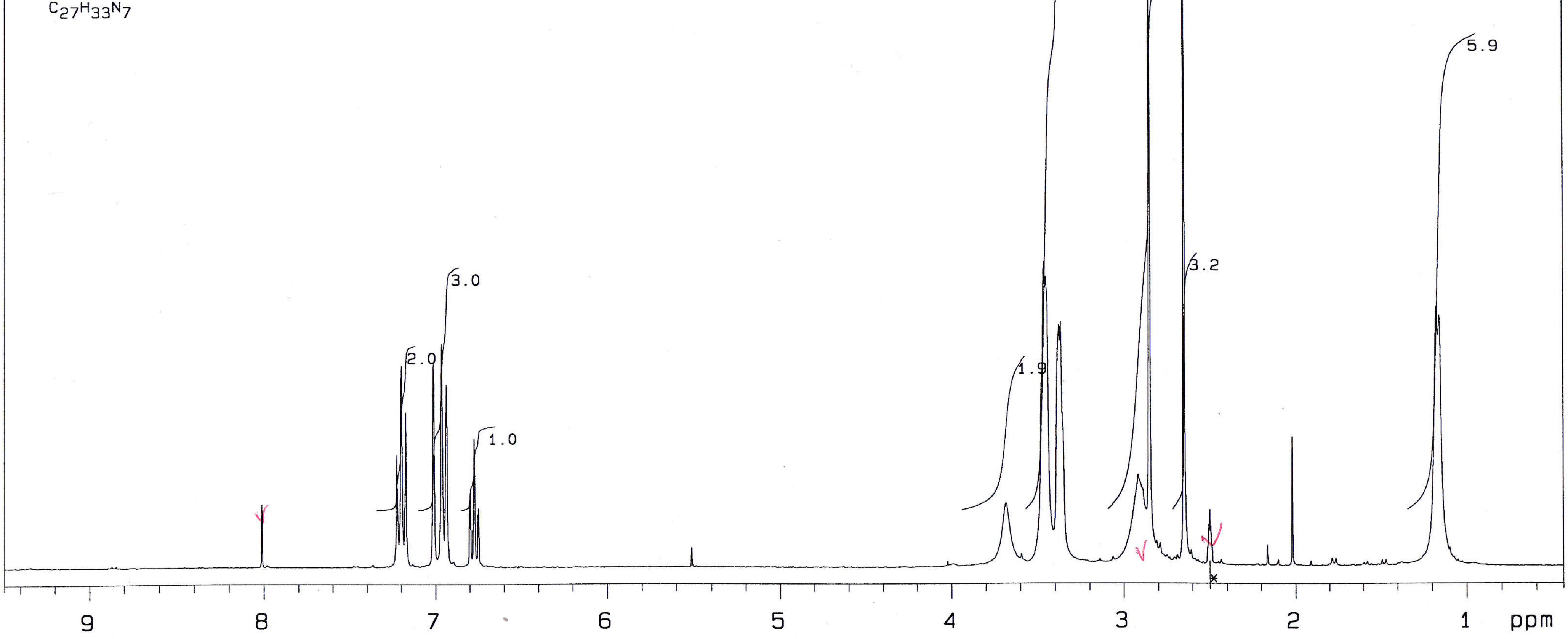

4c

Molecular Structure Research Centre, Yerevan, Armenia, Varian Mercury-300VX

C13 75.465 MHz, nt=1088, np=19998, temp=30.0 C, lb=1.0, solvent=DMSO/CCl4 1/3

Apr 3 2023

ANUSH_TEMA t21-135-4

T21-135-4

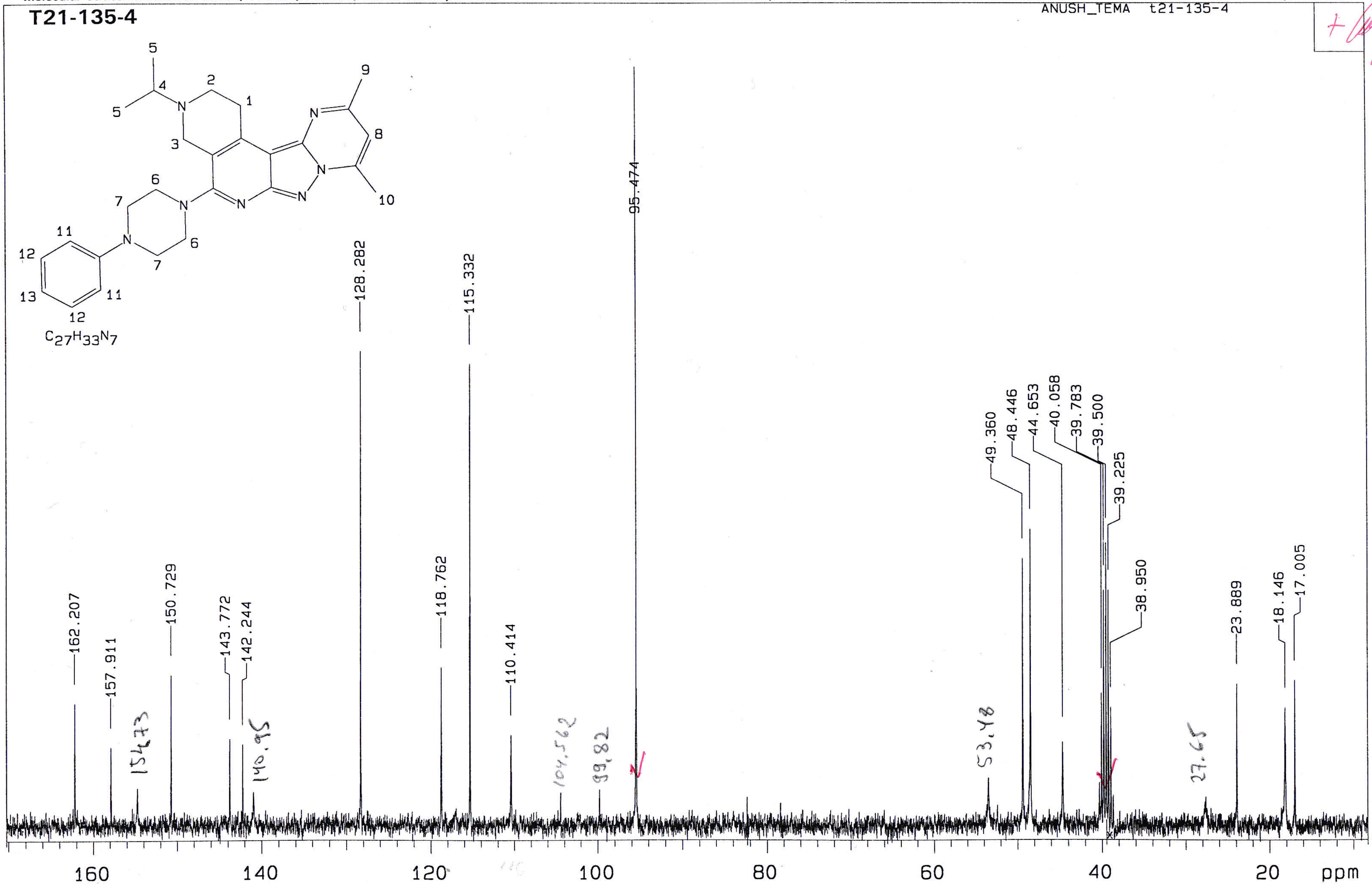

4d

Molecular Structure Research Centre, Yerevan, Armenia, Varian Mercury-300VX H1 300.088 MHz, nt = 16, np = 32000, temp = 30.0 C, lb = -0.2, solvent = DMSO/CCl4 1/3 Apr 5 2022

T21-150

NOCI_22 t21-150

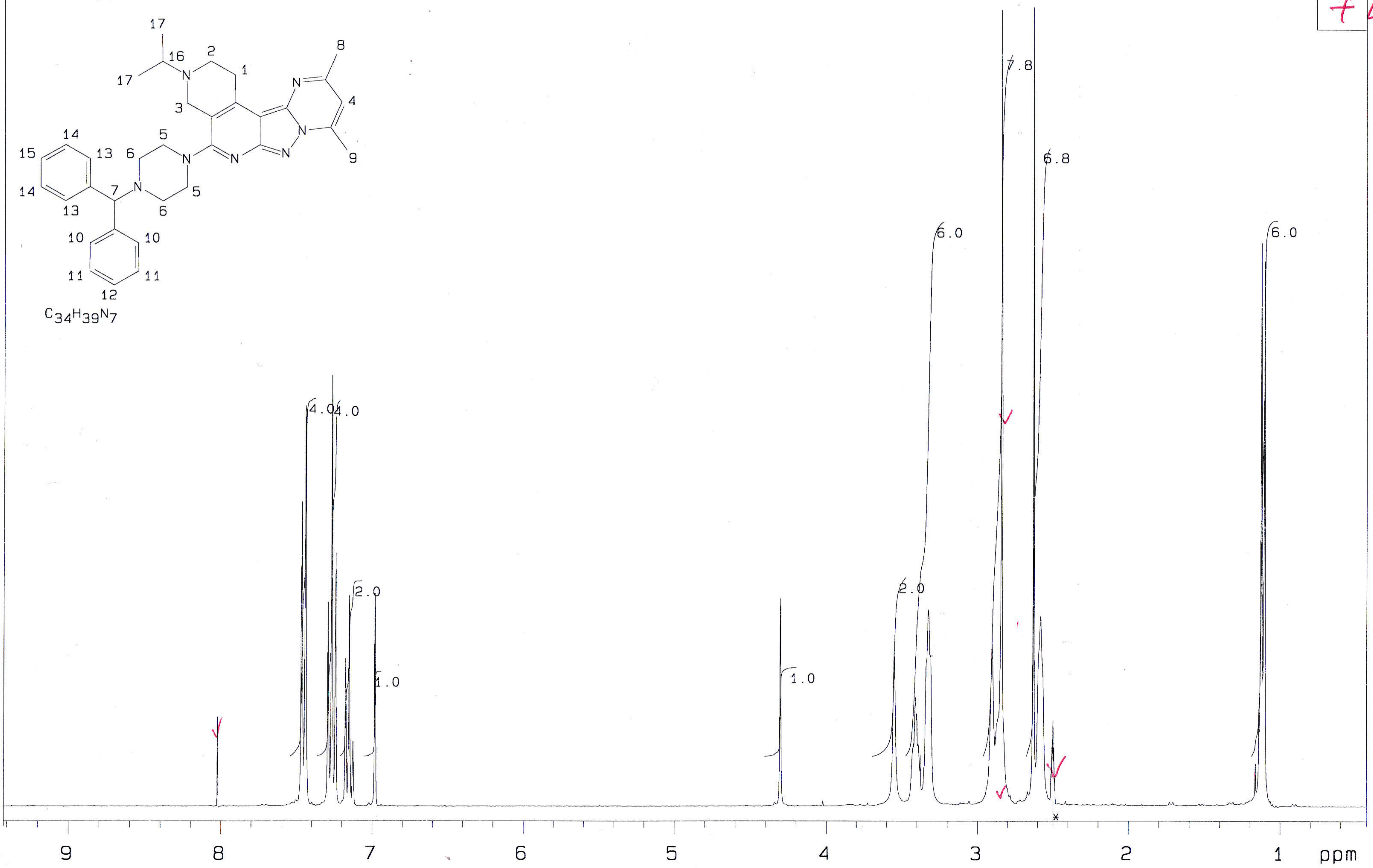

$C_{34}H_{39}N_7$

4d

Molecular Structure Research Centre, Yerevan, Armenia, Varian Mercury-300VX

C13 75.465 MHz, nt = 640, np = 19998, temp = 30.0 C, lb = 1.0, solvent = DMSO

Apr 5 2022

NOCI_22 t21-150

T21-150

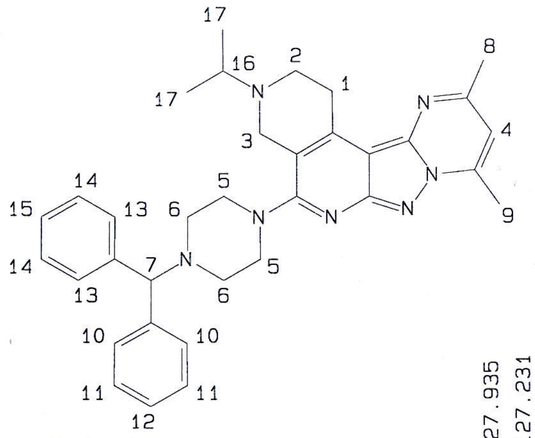

$C_{34}H_{39}N_7$

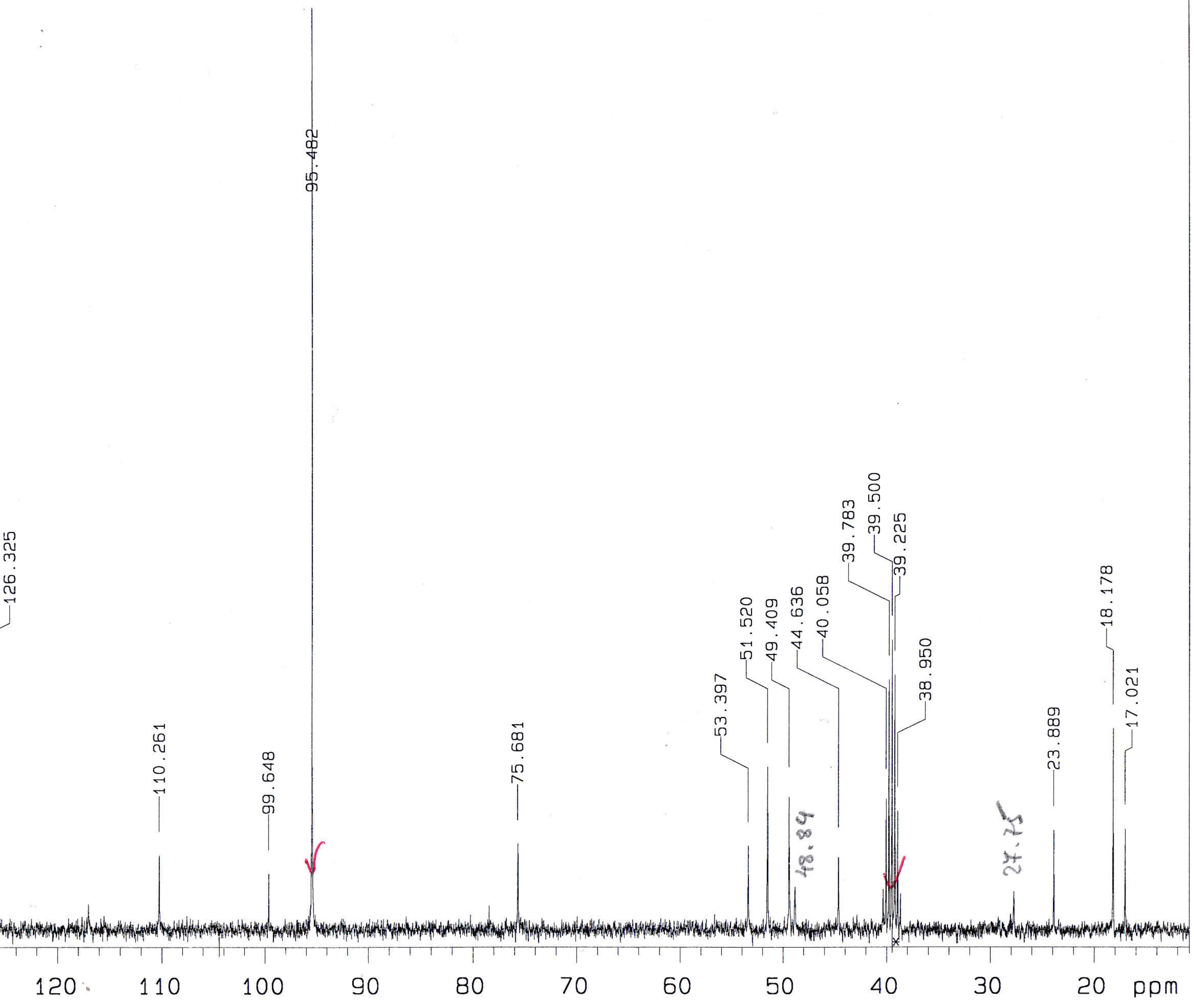

+

4e

Molecular Structure Research Centre, Yerevan, Armenia, Varian Mercury-300VX

H1 300.088 MHz, nt=64, np=32000, temp=30.0 C, lb=-0.2, solvent=DMSO/CCl4 1/3

Feb 24 2023

T21-236

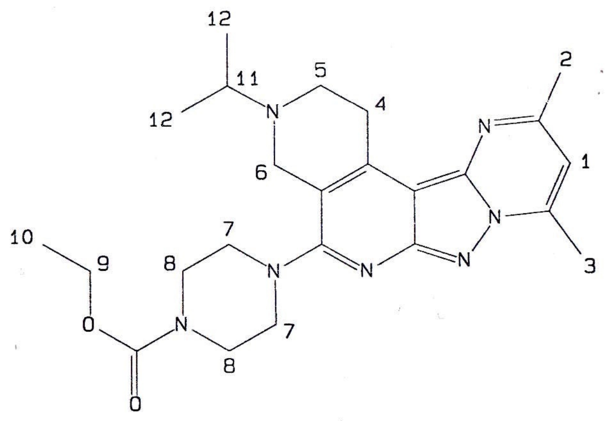

$C_{24}H_{33}N_7O_2$

ANUSH_TEMA t21-236

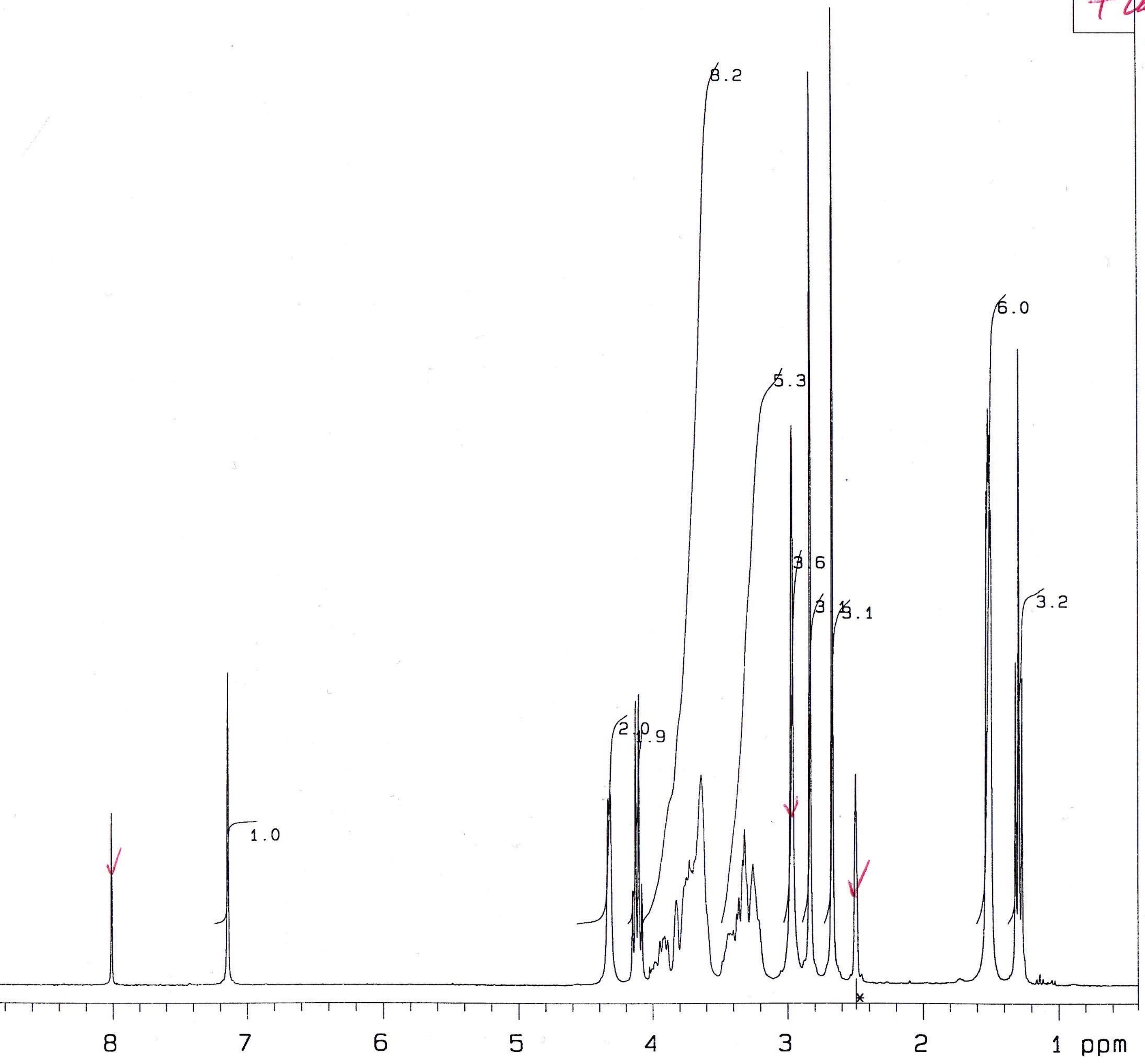

4e

Molecular Structure Research Centre, Yerevan, Armenia, Varian Mercury-300VX   C13 75.465 MHz, nt=512, np=19998, temp=30.0 C, lb=1.0, solvent=DMSO   Feb 24 2023

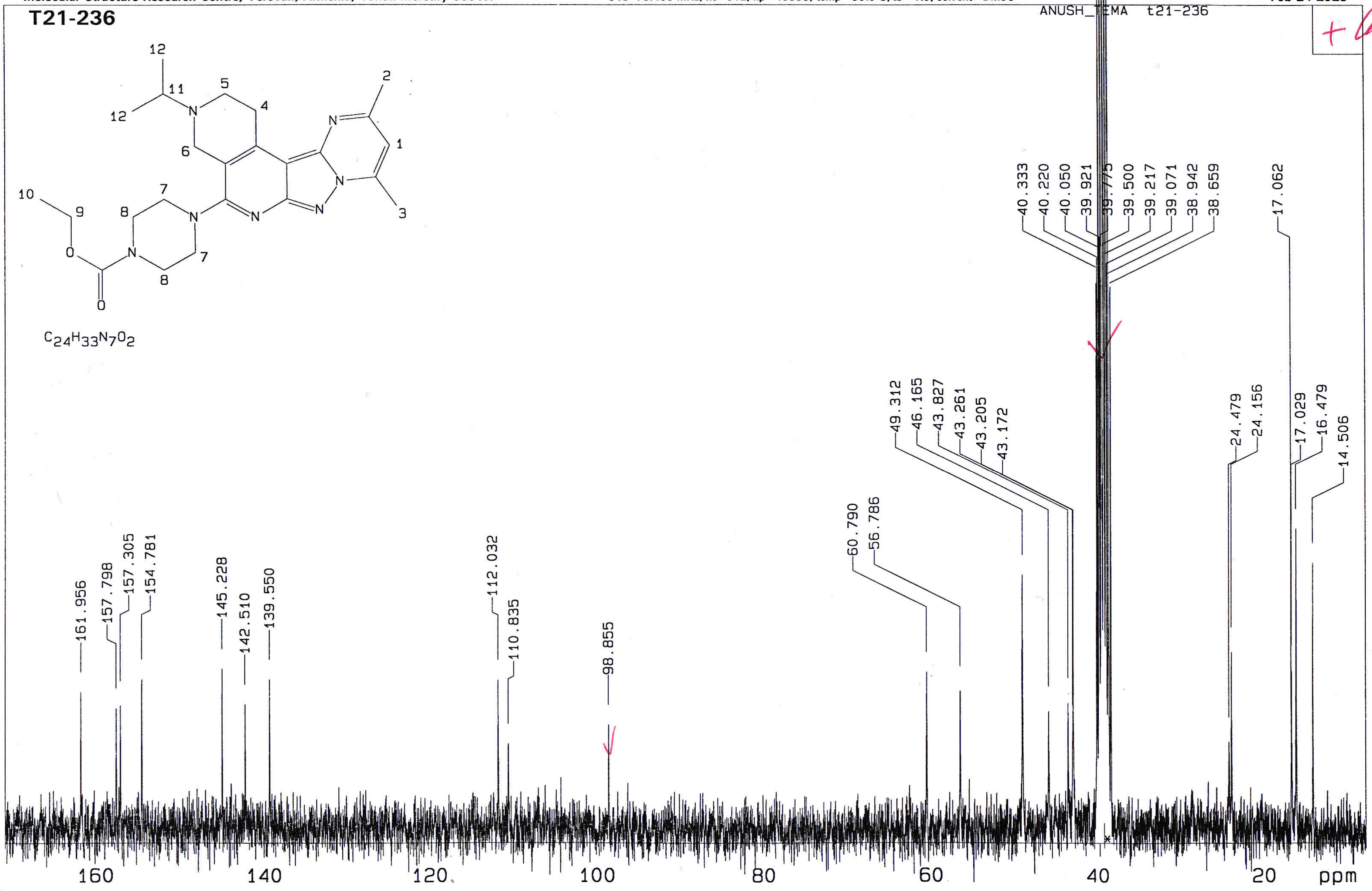

4e

17.01.2025

T21-236 (0.053) Is (1.00,1.00) C24H33N7O2

1: TOF MS ES+
7.46e12

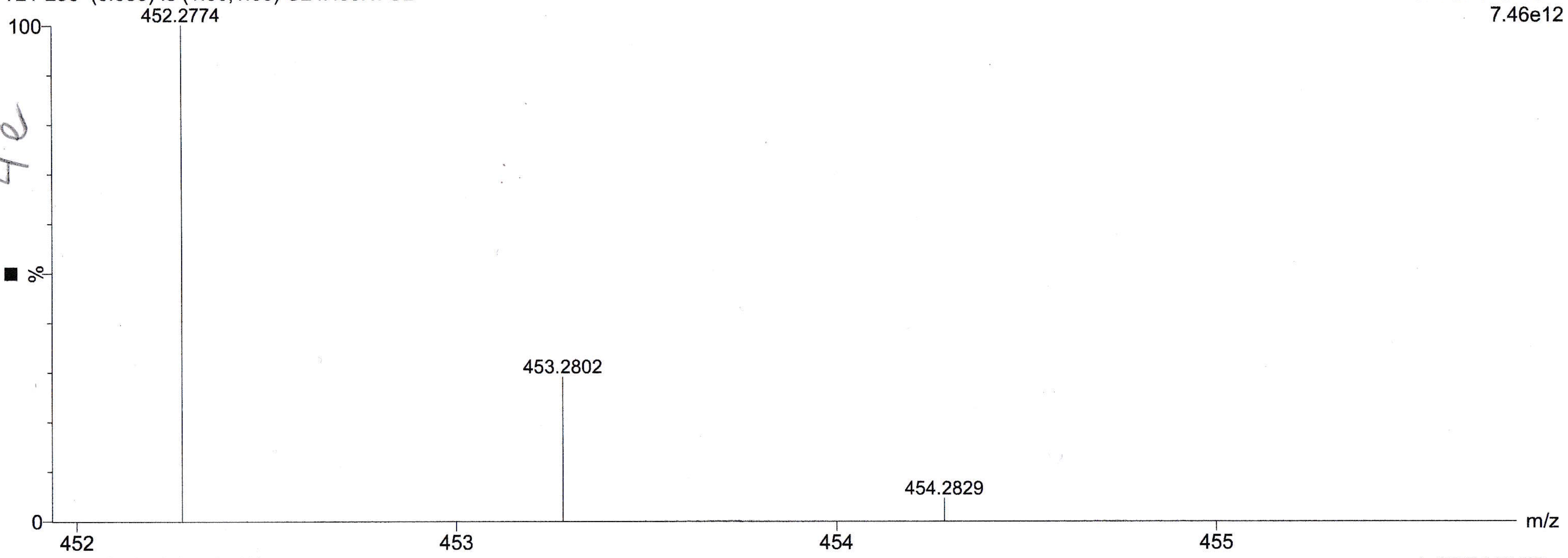

T21-236 7 (0.155) Cm (7:10)

1: TOF MS ES+
2.64e7

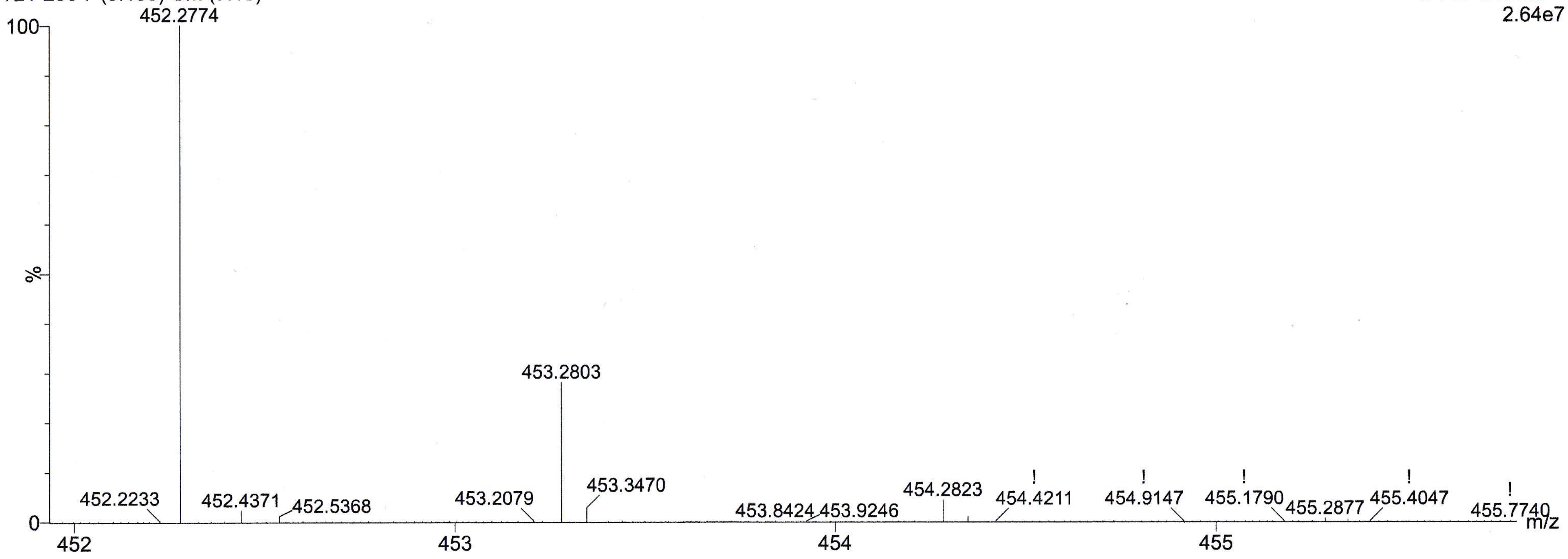

4f

Molecular Structure Research Centre, Yerevan, Armenia, Varian Mercury-300VX

H1 300.088 MHz, nt = 16, np = 32000, temp = 30.0 C, lb = -0.2, solvent = DMSO/CCl4 1/3

Jan 25 2023

ANUSH_TEMA t21-221

T21-221

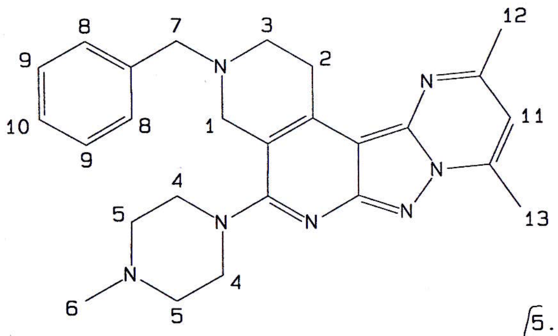

$C_{26}H_{31}N_7$

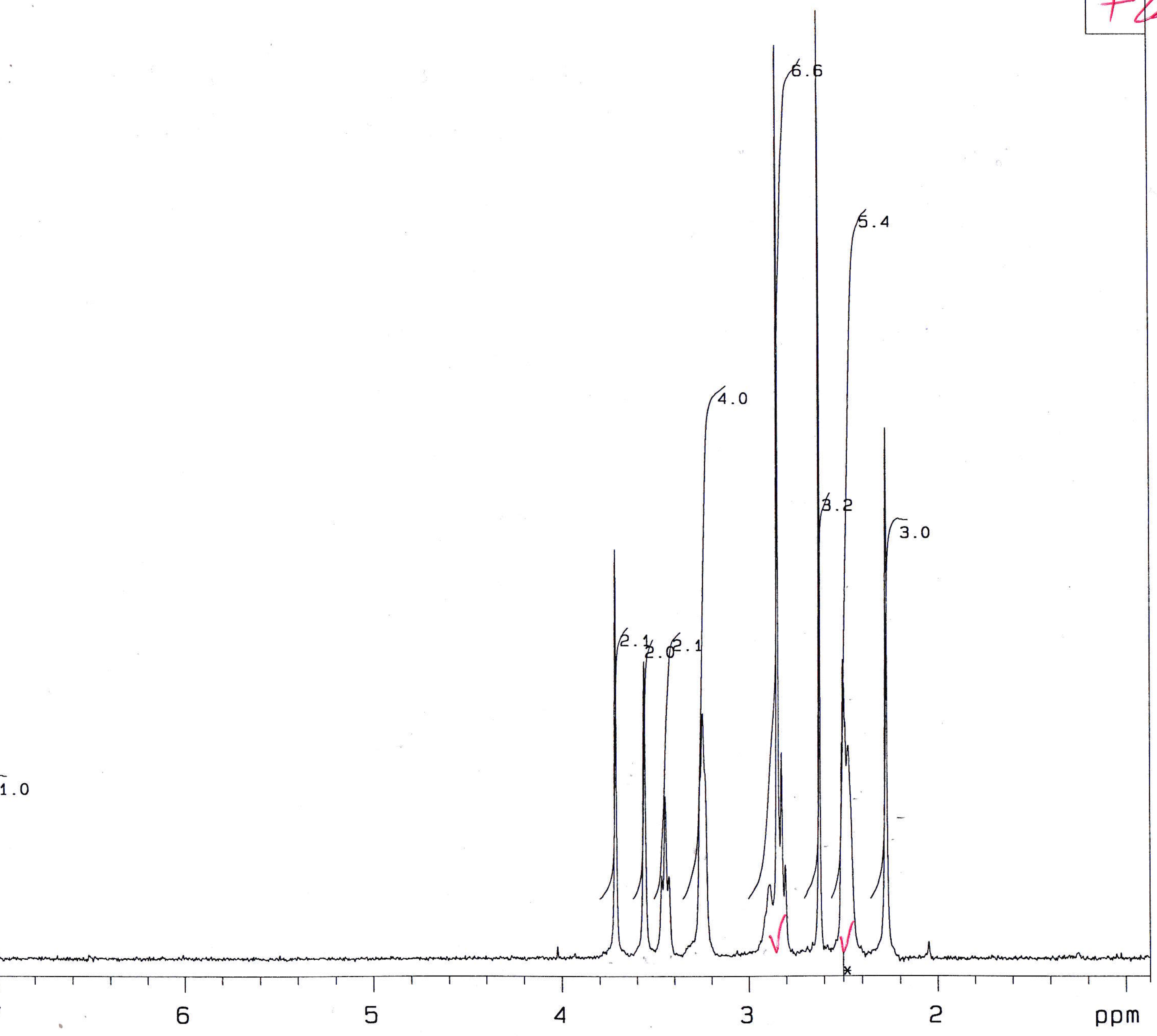

Molecular Structure Research Centre, Yerevan, Armenia, Varian Mercury-300VX

C13 75.465 MHz, nt=384, np=19998, temp=30.0 C, lb=1.0, solvent=DMSO/CCl4 1/3

Jan 25 2023

ANUSH_TEMA t21-221

T21-221

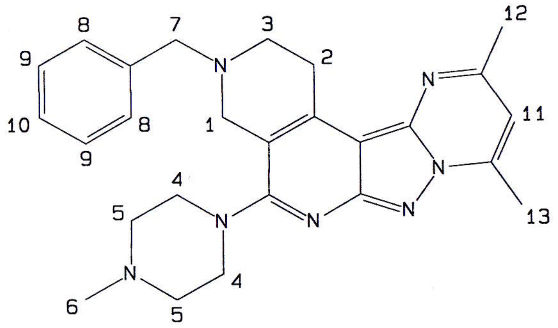

$C_{26}H_{31}N_7$

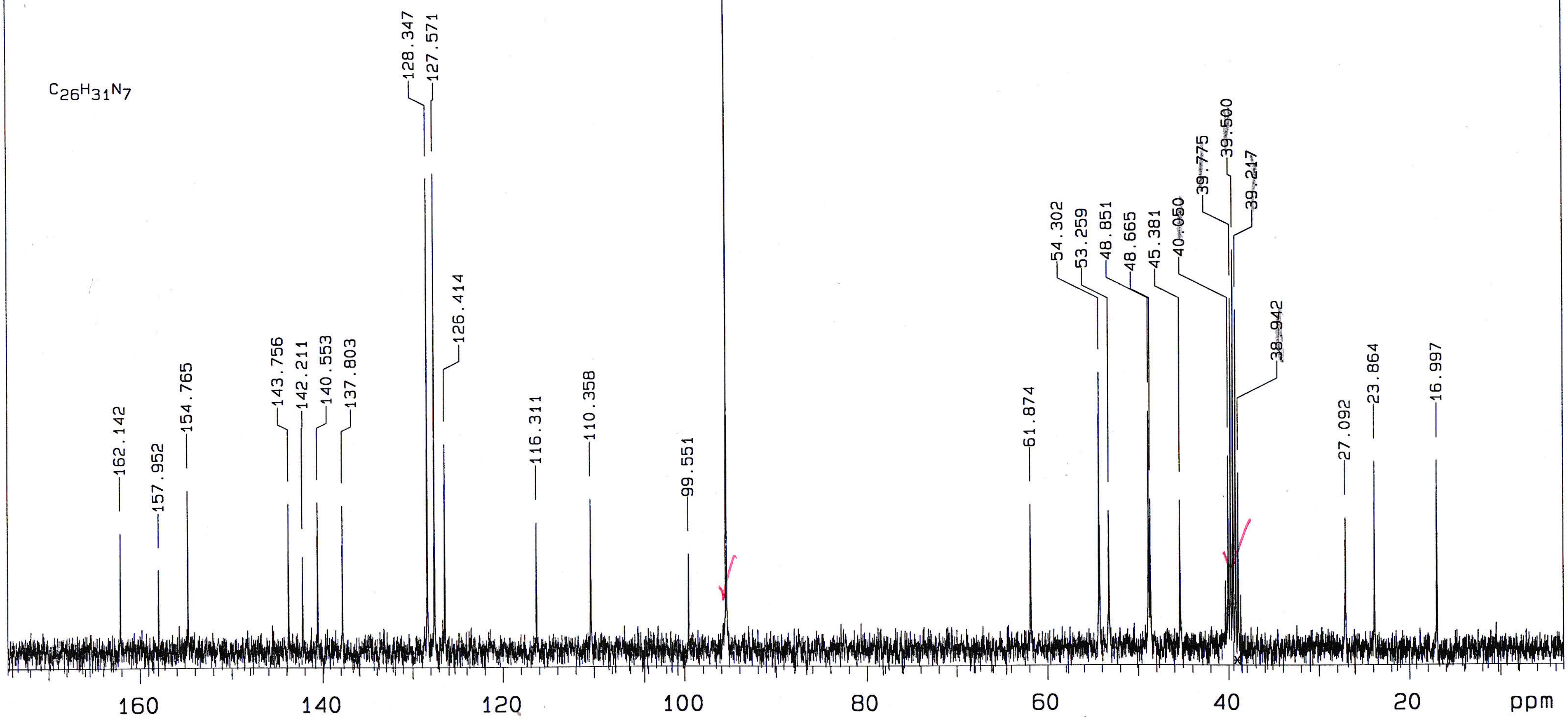

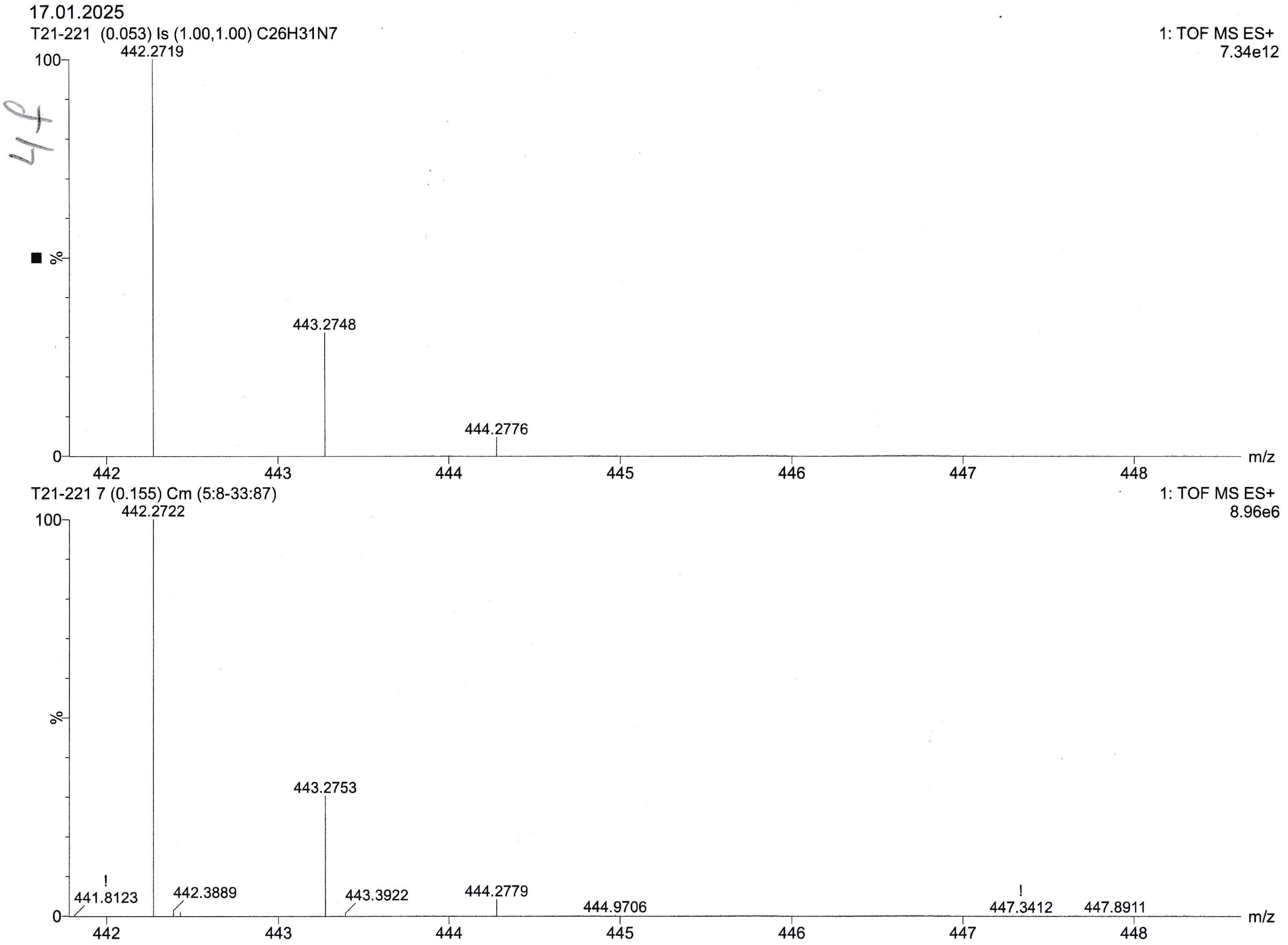
17.01.2025
4f
T21-221 (0.053) Is (1.00,1.00) C26H31N7
1: TOF MS ES+
7.34e12
442.2719
443.2748
444.2776
100
%
0
442
443
444
445
446
447
448
m/z
T21-221 7 (0.155) Cm (5:8-33:87)
1: TOF MS ES+
8.96e6
442.2722
443.2753
441.8123
442.3889
443.3922
444.2779
444.9706
447.3412
447.8911
100
%
0
442
443
444
445
446
447
448
m/z

Molecular Structure Research Center, NAS RA, Yerevan, Armenia

null SF 400.1500038MHz Temp nullK TD null SWH null NS null LB 0.30 GB 0 solvent=null

April 10, 2025 12:24

Avance 400 Neo

TopSpin 4.1.3

hy-232, 1H, CDCl3, temp=30

hy-232 1 1 F:/FID_BRUKER/NOCI_25

4g

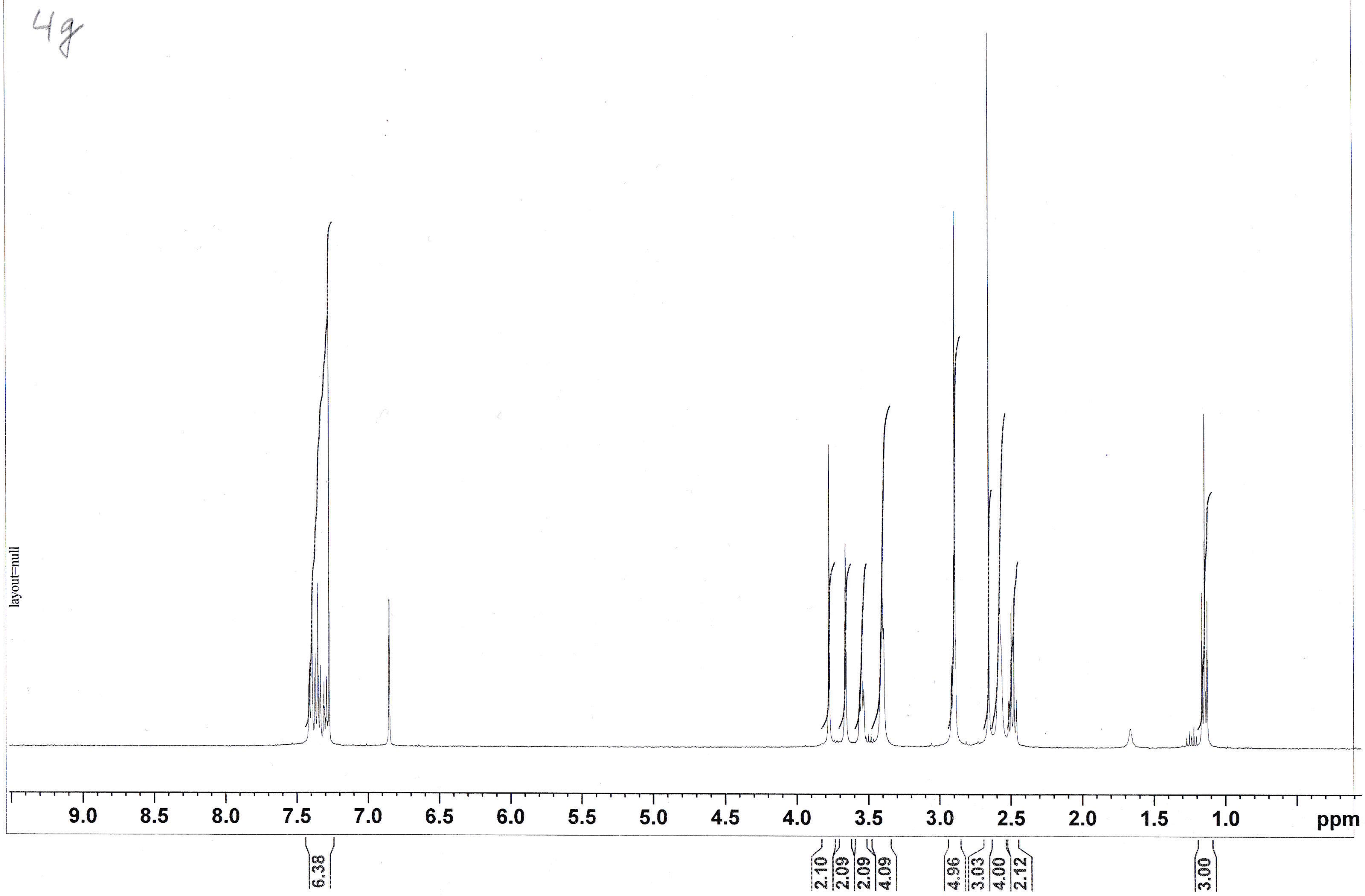

© Molecular Structure Research Center, NAS RA, Yerevan, Armenia
null SF = 100.6177891MHz Temp = nullK TD = null SWH = null NS = null LB = 1.00 GB = 0 solvent: null

April 10, 2025 12:41

Avance 400 Neo
TopSpin 4.1.3

hy-232, 13C, CDCl3

hy-232 2 1 F:/FID_BRUKER/NOCI_25

4g

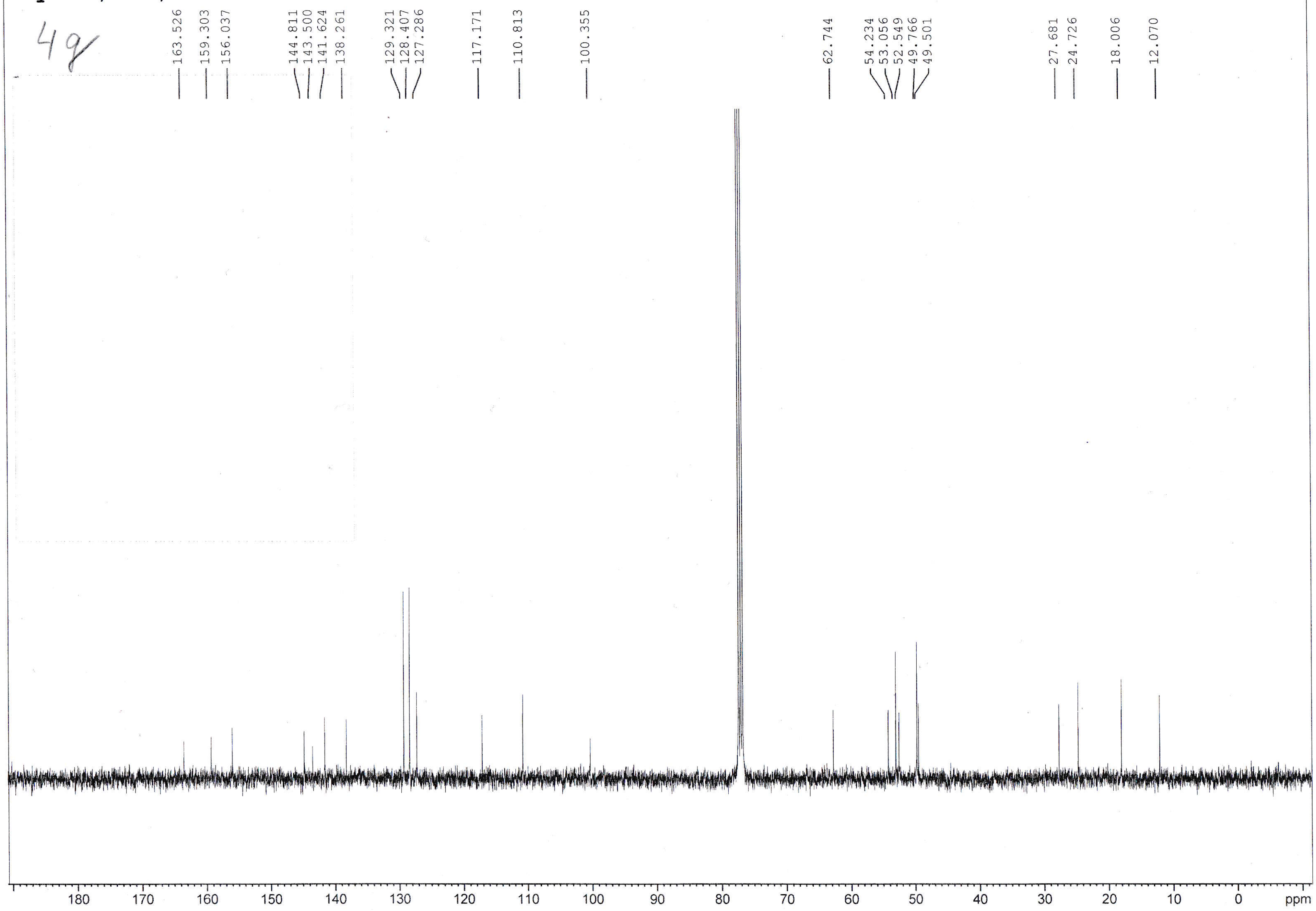

07.02.2024

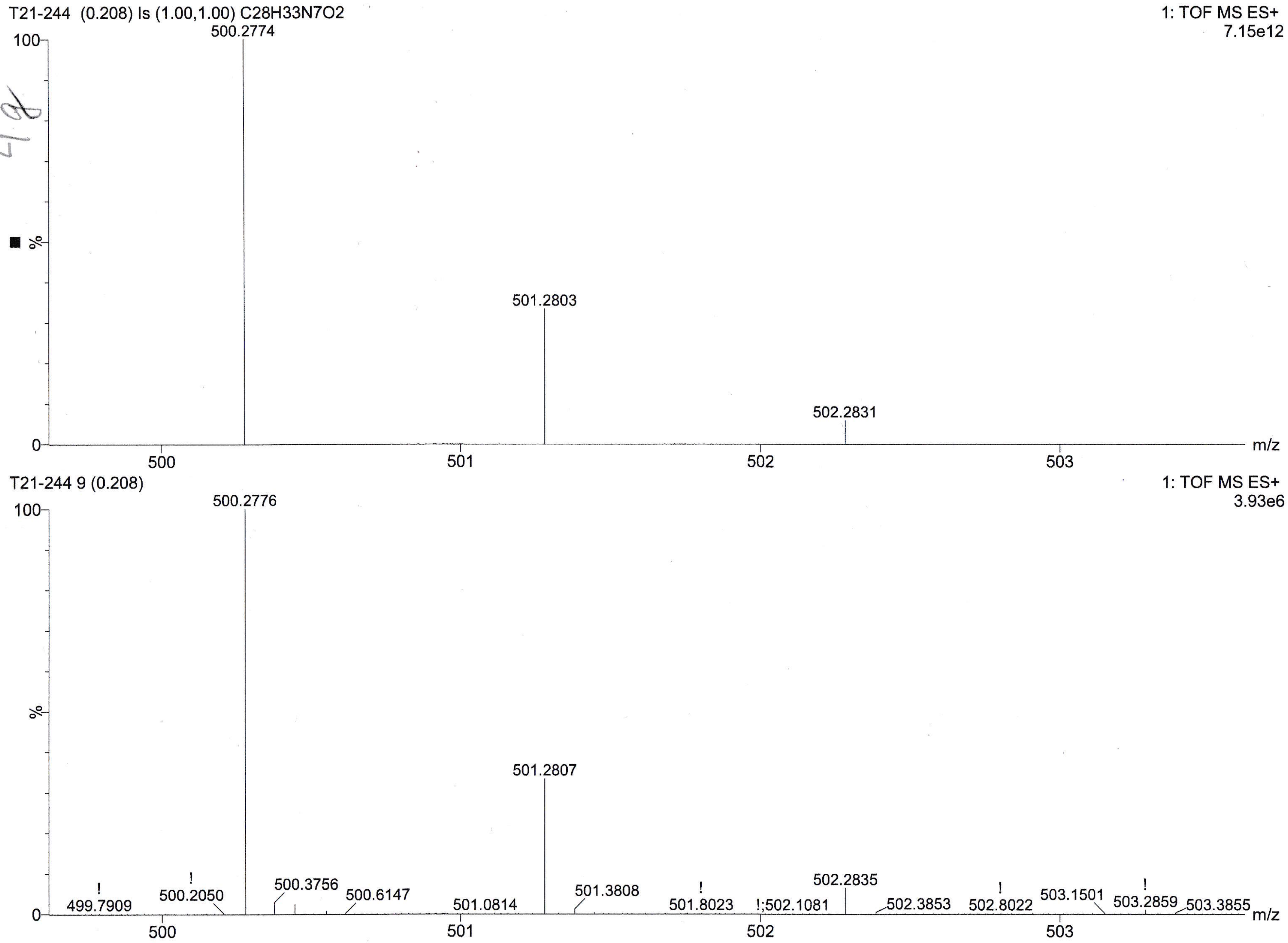

T21-244 (0.208) Is (1.00,1.00) C28H33N7O2
1: TOF MS ES+
7.15e12
500.2774
501.2803
502.2831
T21-244 9 (0.208)
1: TOF MS ES+
3.93e6
499.7909
500.2050
500.2776
500.3756
500.6147
501.0814
501.2807
501.3808
501.8023
502.1081
502.2835
502.3853
502.8022
503.1501
503.2859
503.3855
%
m/z

4h

Molecular Structure Research Centre, Yerevan, Armenia, Varian Mercury-300VX

H1 300.088 MHz, nt=16, np=32000, temp=30.0 C, lb=-0.2, solvent=DMSO/CCl4 1/3

Feb 6 2023

ANUSH_TEMA t21-231

T21-231

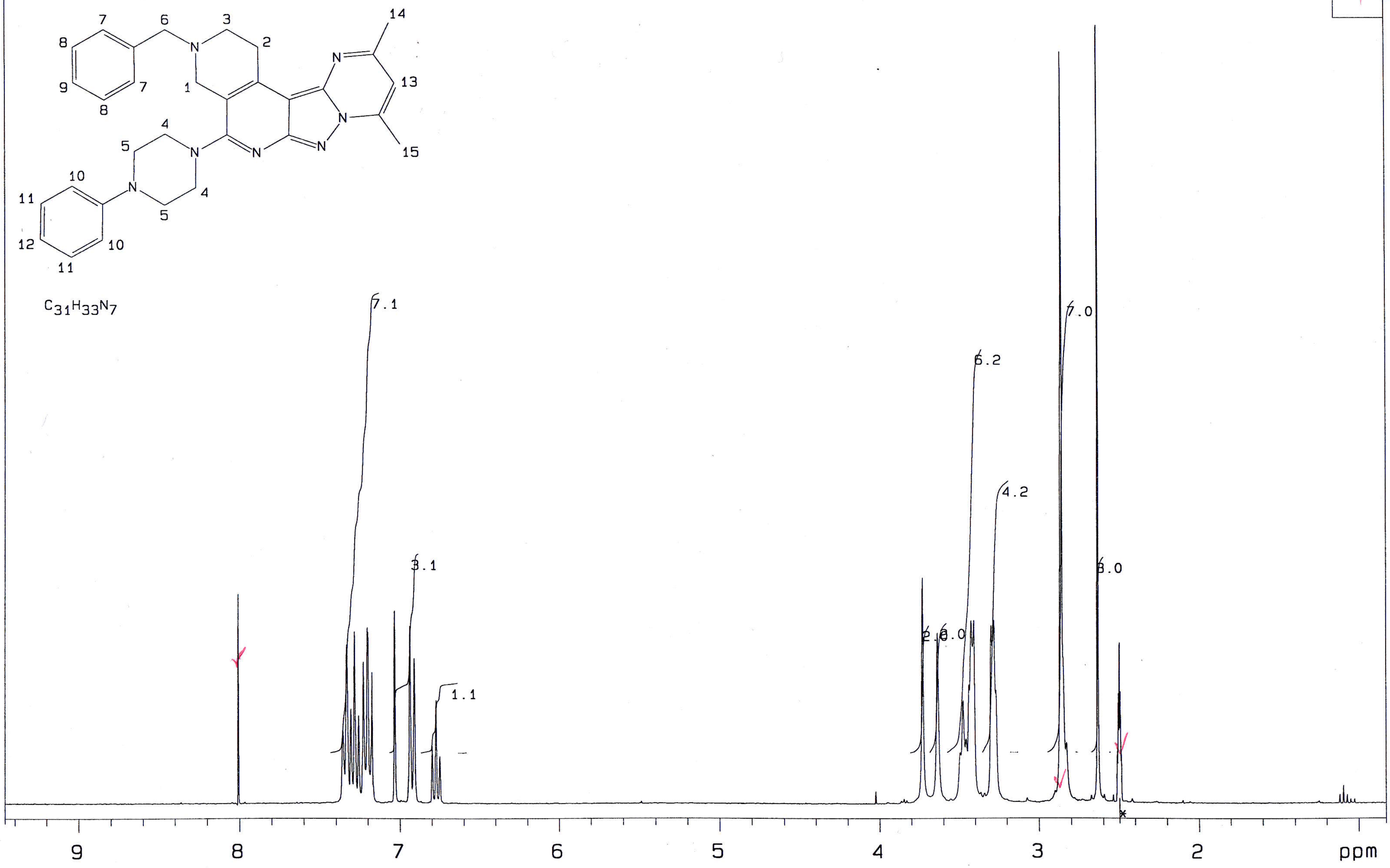

4h

Molecular Structure Research Centre, Yerevan, Armenia, Varian Mercury-300VX

C13 75.465 MHz, nt=576, np=19998, temp=30.0 C, lb=1.0, solvent=DMSO/CCl4 1/3

Feb 6 2023

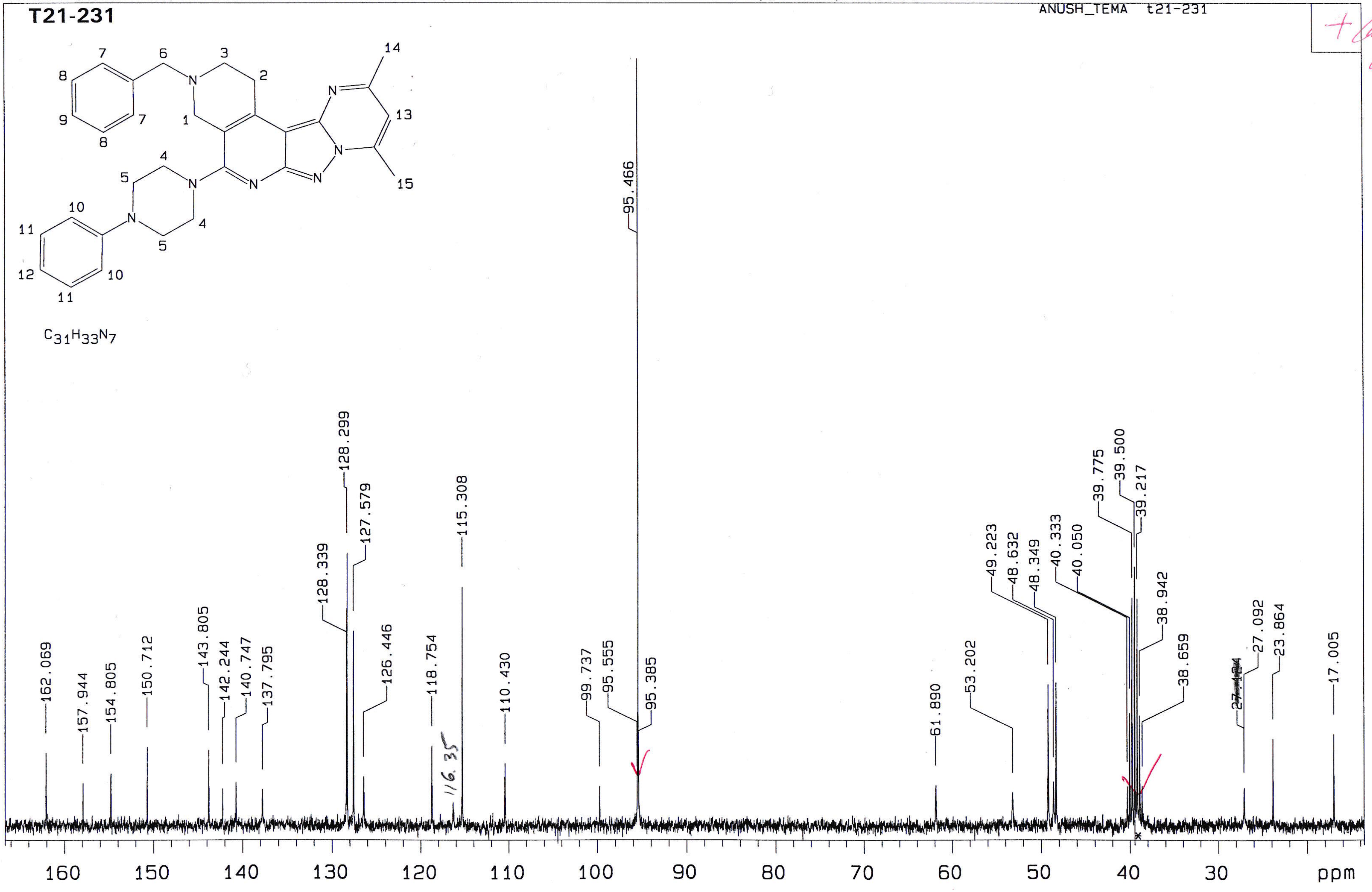

4h

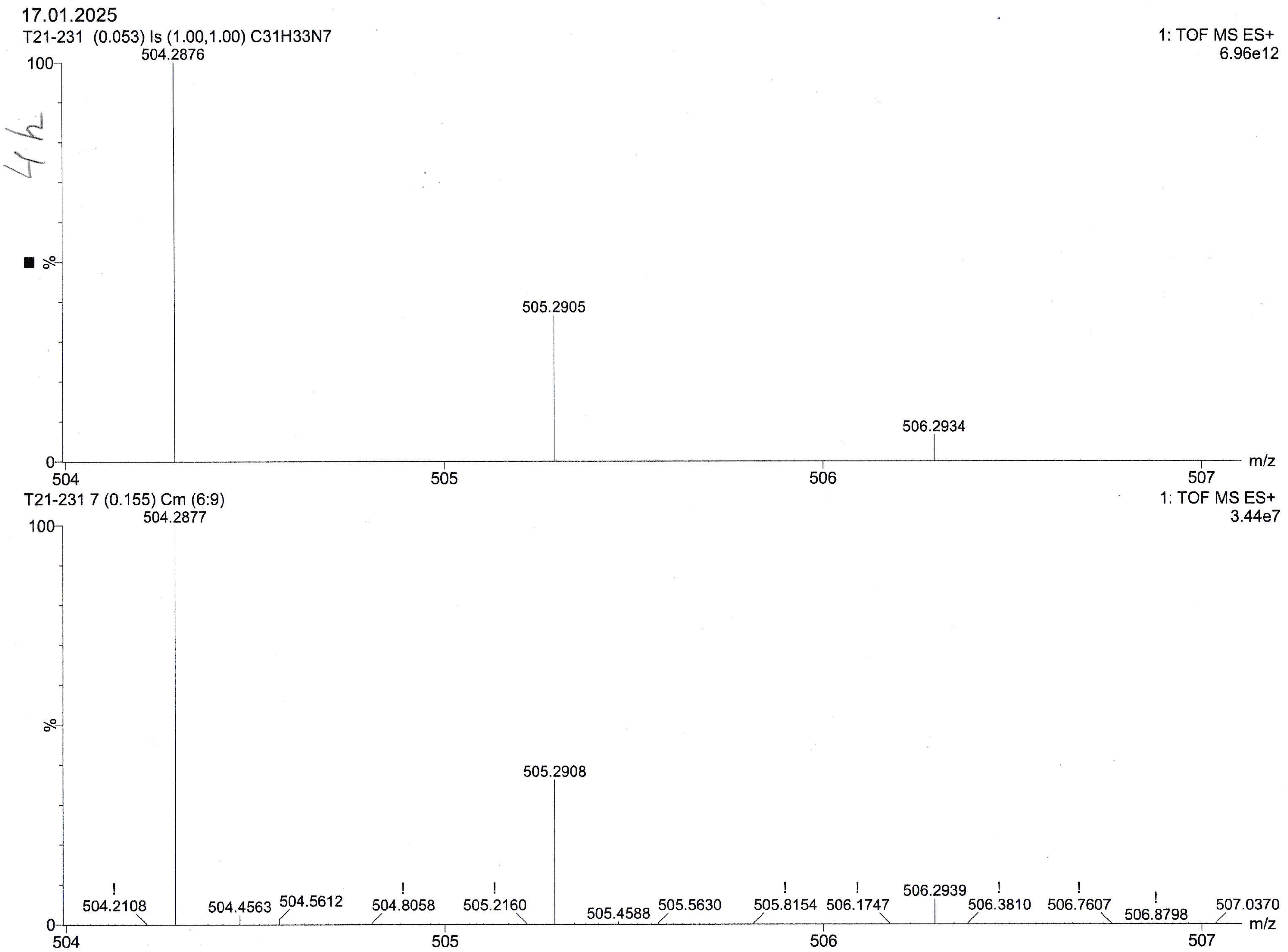
17.01.2025
T21-231 (0.053) Is (1.00,1.00) C31H33N7
1: TOF MS ES+
6.96e12
100
%
0
504.2876
505.2905
506.2934
504
505
506
507
m/z
T21-231 7 (0.155) Cm (6:9)
1: TOF MS ES+
3.44e7
100
%
0
504.2877
505.2908
504.2108
504.4563
504.5612
504.8058
505.2160
505.4588
505.5630
505.8154
506.1747
506.2939
506.3810
506.7607
506.8798
507.0370
504
505
506
507
m/z

4i

Molecular Structure Research Centre, Yerevan, Armenia, Varian Mercury-300VX

H1 300.088 MHz, nt=16, np=32000, temp=30.0 C, lb=-0.2, solvent=DMSO

Mar 9 2023

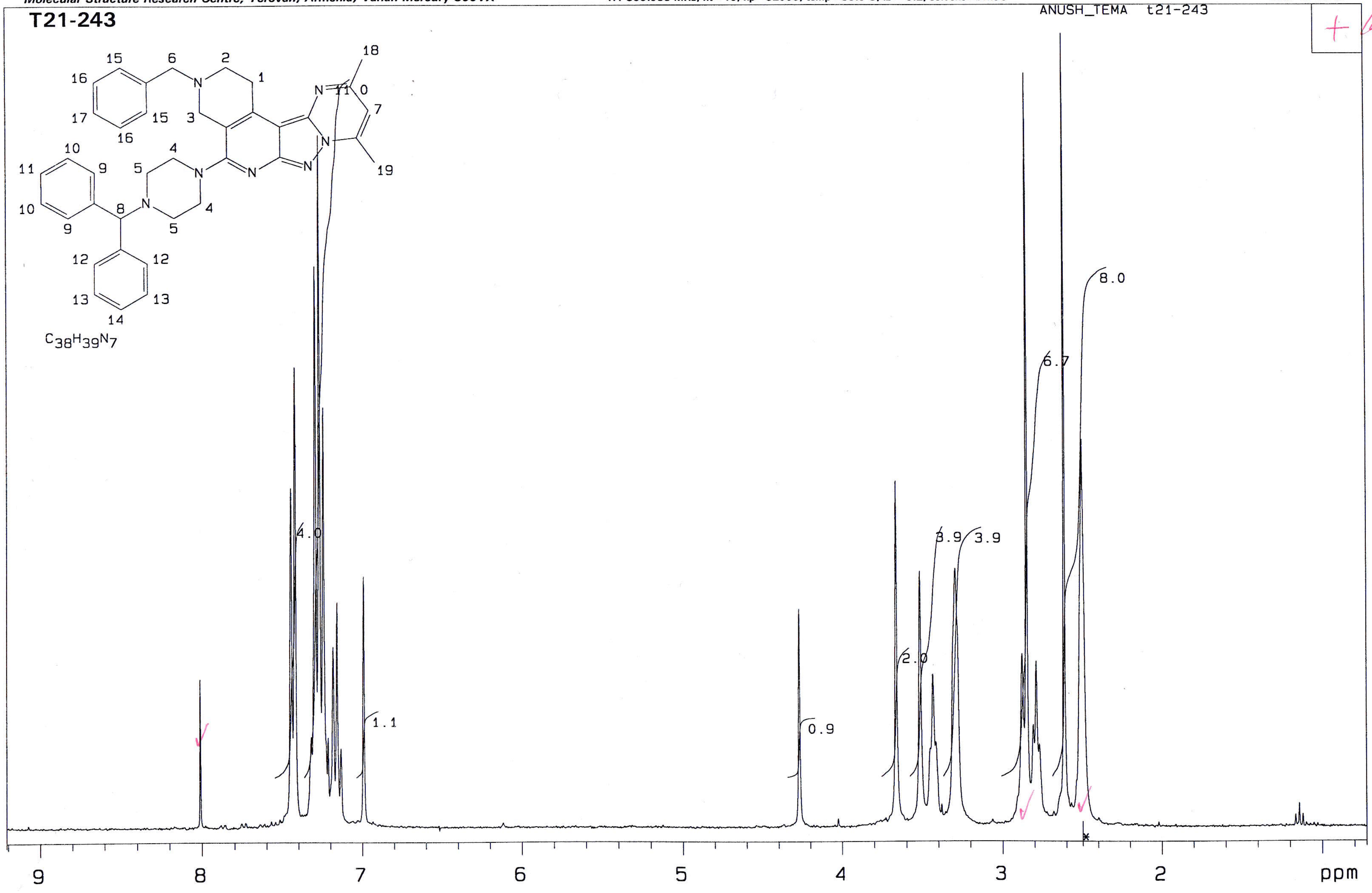

4i

Molecular Structure Research Centre, Yerevan, Armenia, Varian Mercury-300VX

C13 75.465 MHz, nt = 1648, np = 19998, temp = 30.0 C, lb = 1.0, solvent = DMSO/CCl4 1/3

Mar 9 2023

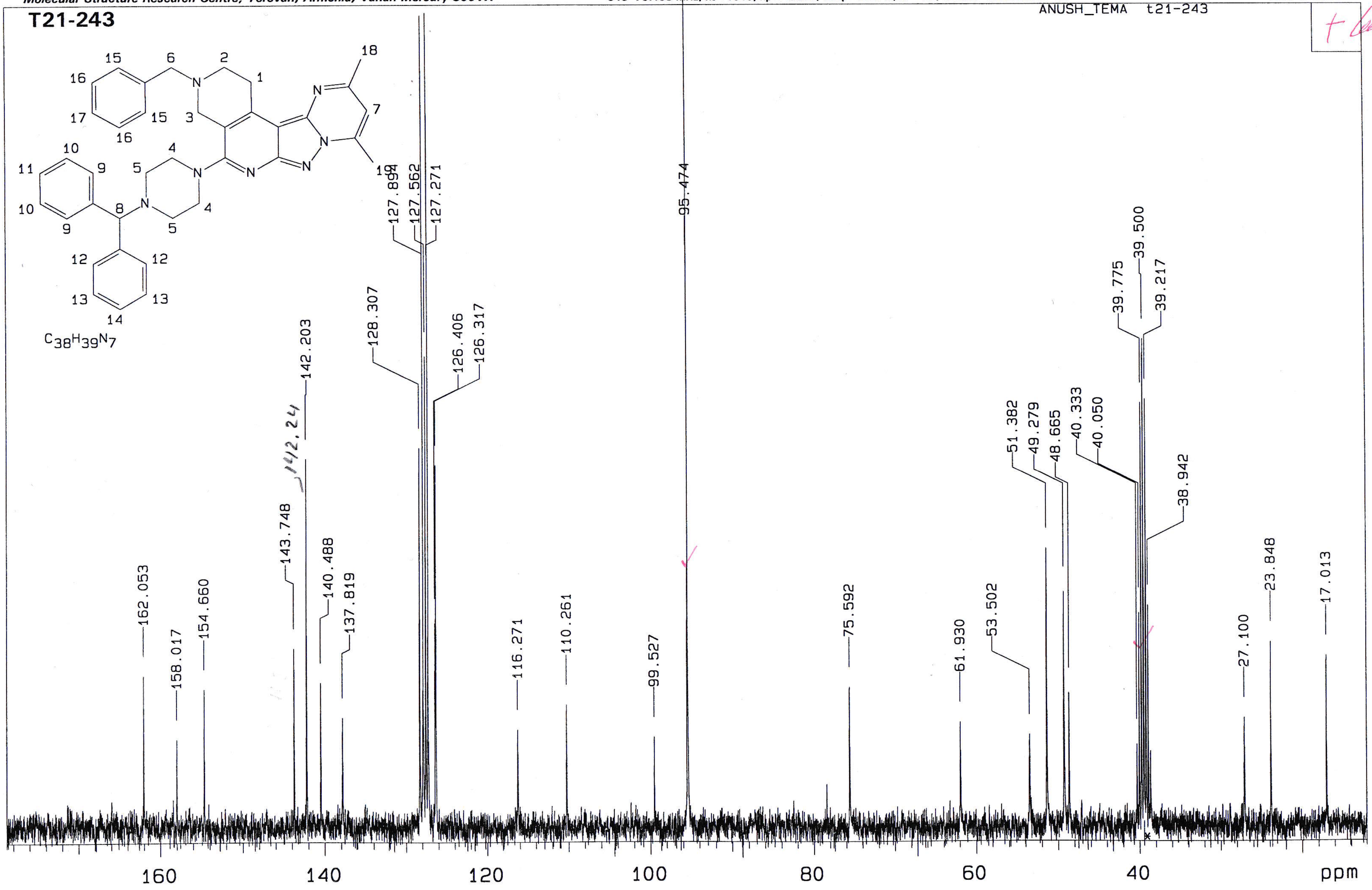

4j

Molecular Structure Research Centre, Yerevan, Armenia, Varian Mercury-300VX

H1 300.088 MHz, nt = 16, np = 32000, temp = 30.0 C, lb = -0.2, solvent = DMSO/CCl4 1/3

Mar 16 2023

ANUSH_TEMA t21-244

T21-244

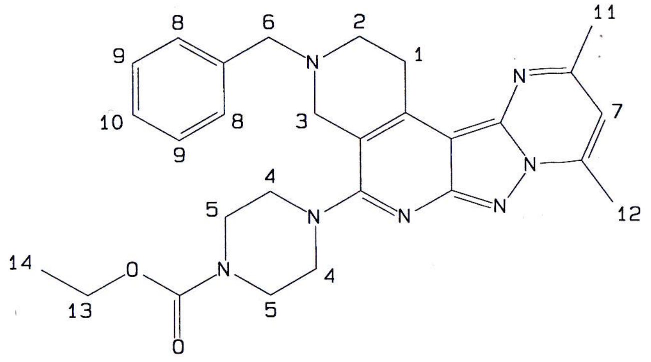

$C_{28}H_{33}N_7O_2$

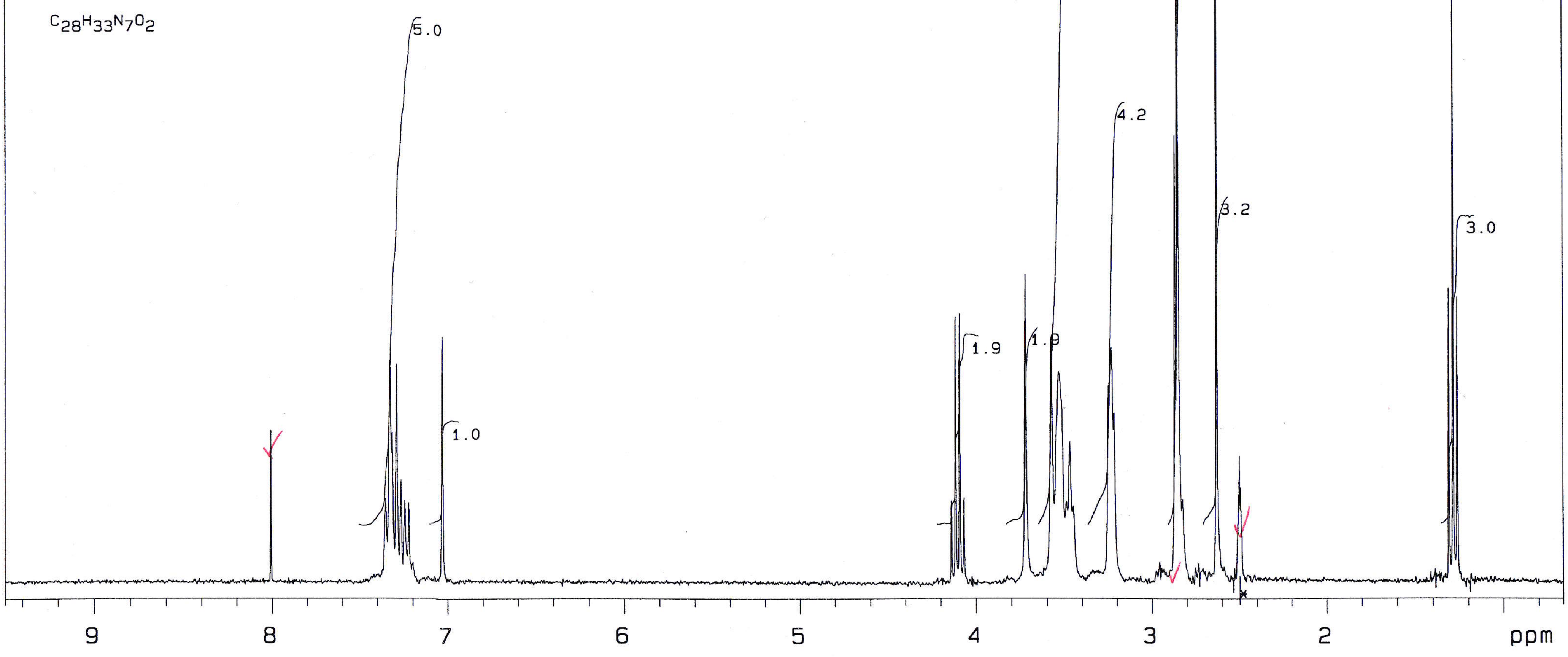

4j

Molecular Structure Research Centre, Yerevan, Armenia, Varian Mercury-300VX

C13 75.465 MHz, nt=992, np=19998, temp=30.0 C, lb=1.0, solvent=DMSO/CCl4 1/3

Mar 16 2023

T21-244

ANUSH_TEMA t21-244

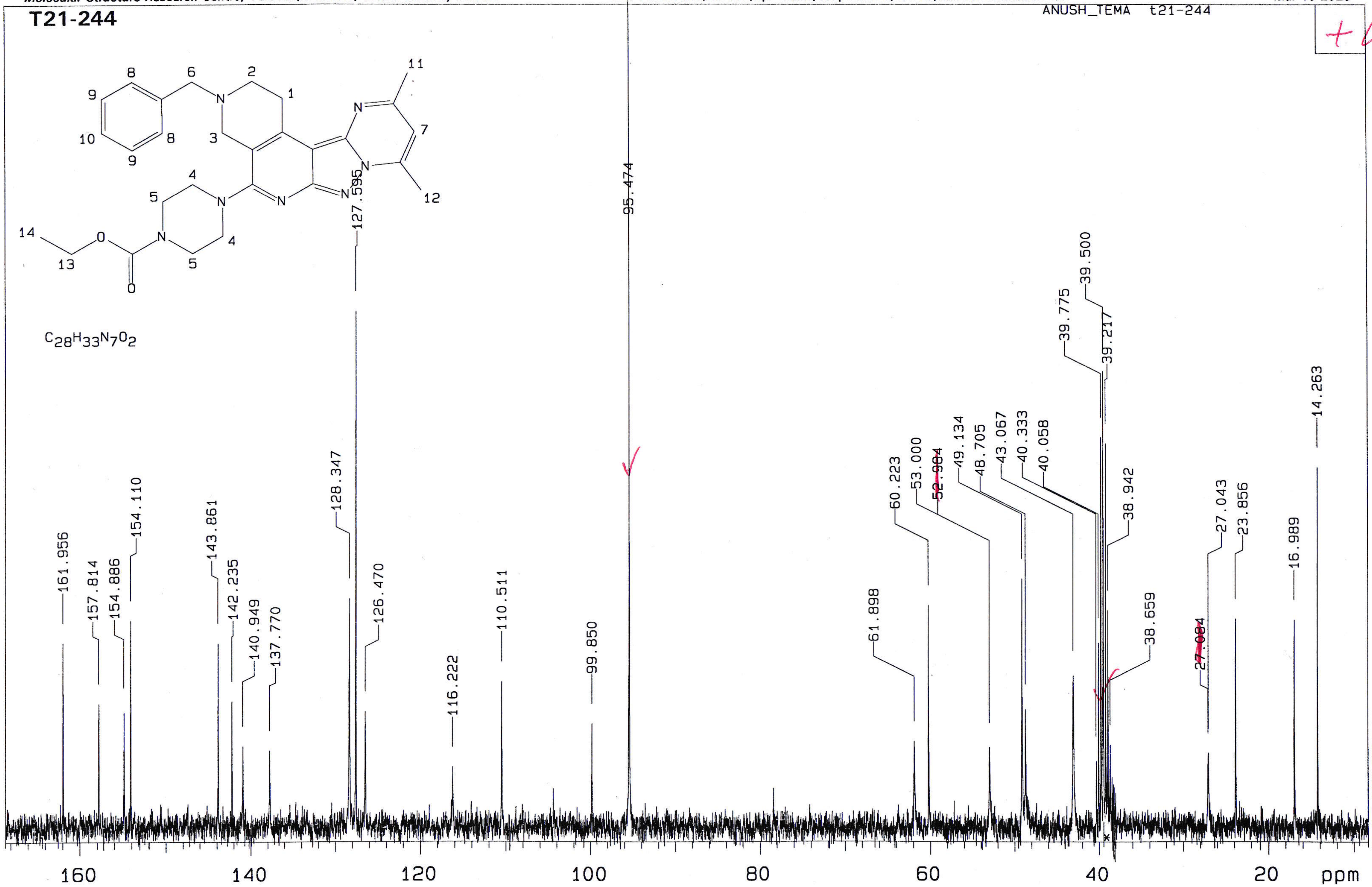

Molecular Structure Research Center, NAS RA, Yerevan, Armenia

null SF 400.1500038MHz Temp nullK TD null SWH null NS null LB 0.30 GB 0 solvent=null

December 5, 2024 3

Avance 400 Neo

TopSpin 4.1.3

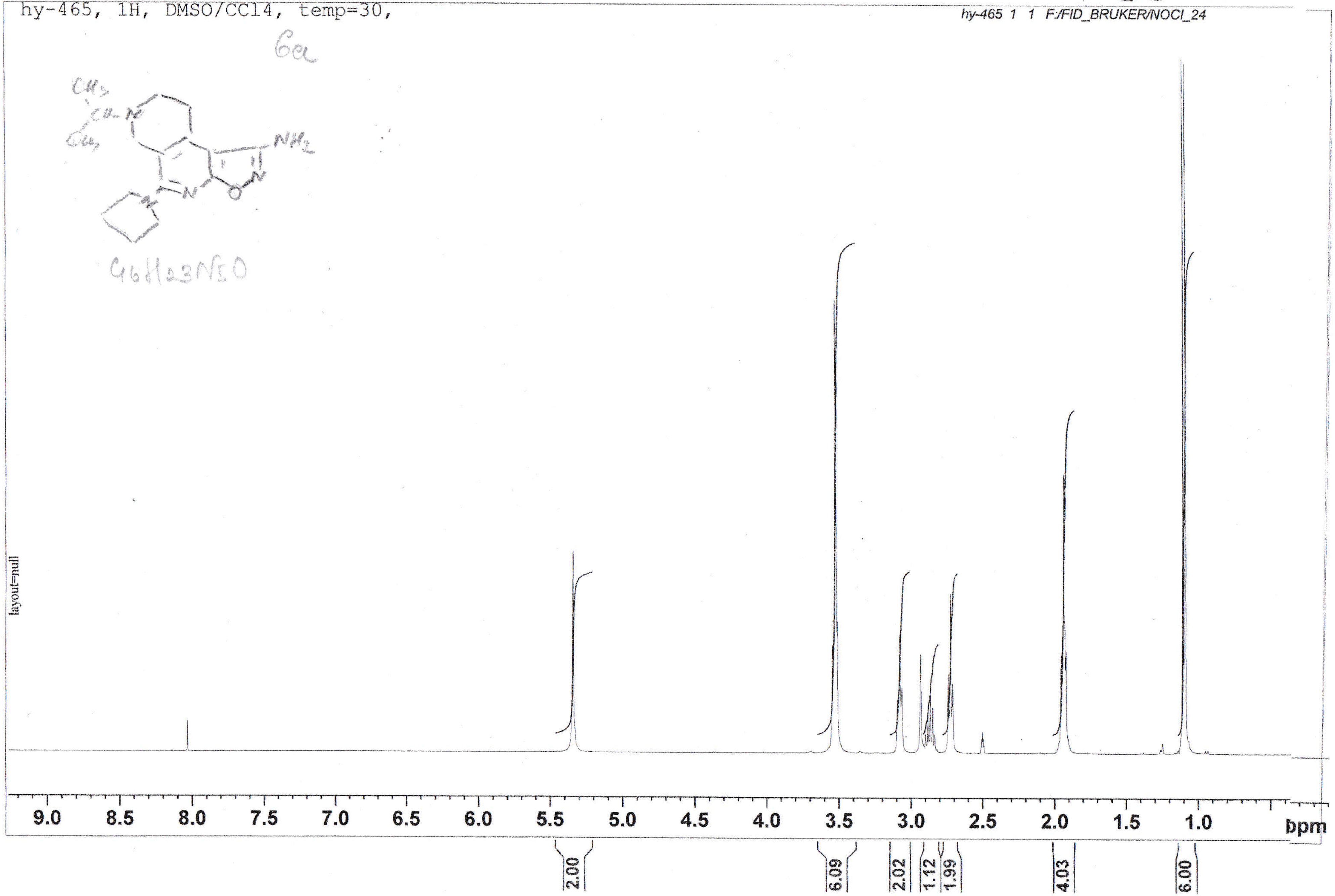

© Molecular Structure Research Center, NAS RA, Yerevan, Armenia

null SF = 100.6178654MHz Temp = nullK TD = null SWH = null NS = null LB = 1.00 GB = 0 solvent: null

December 5, 2024 3

Avance 400 Neo
TopSpin 4.1.3

6a

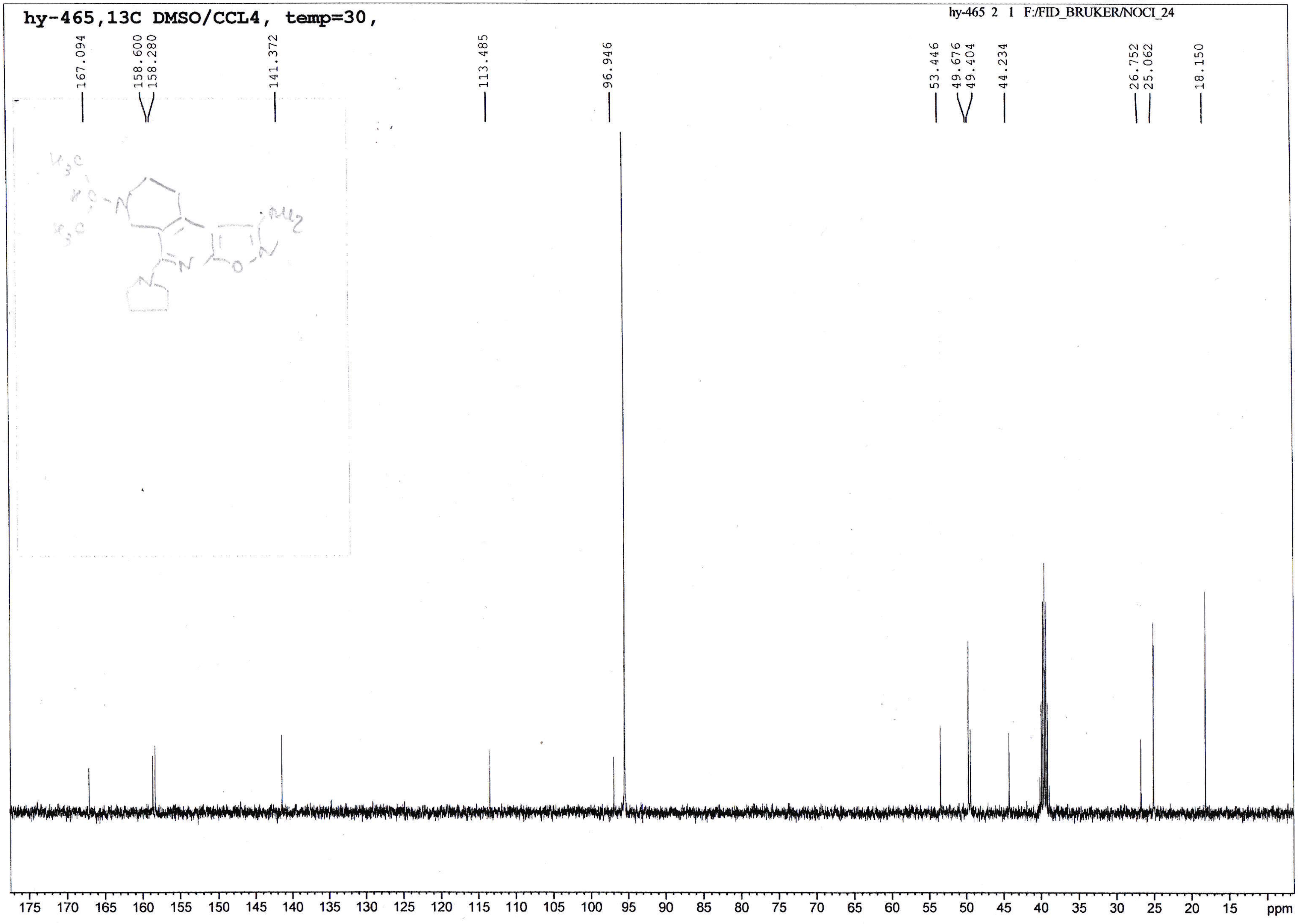

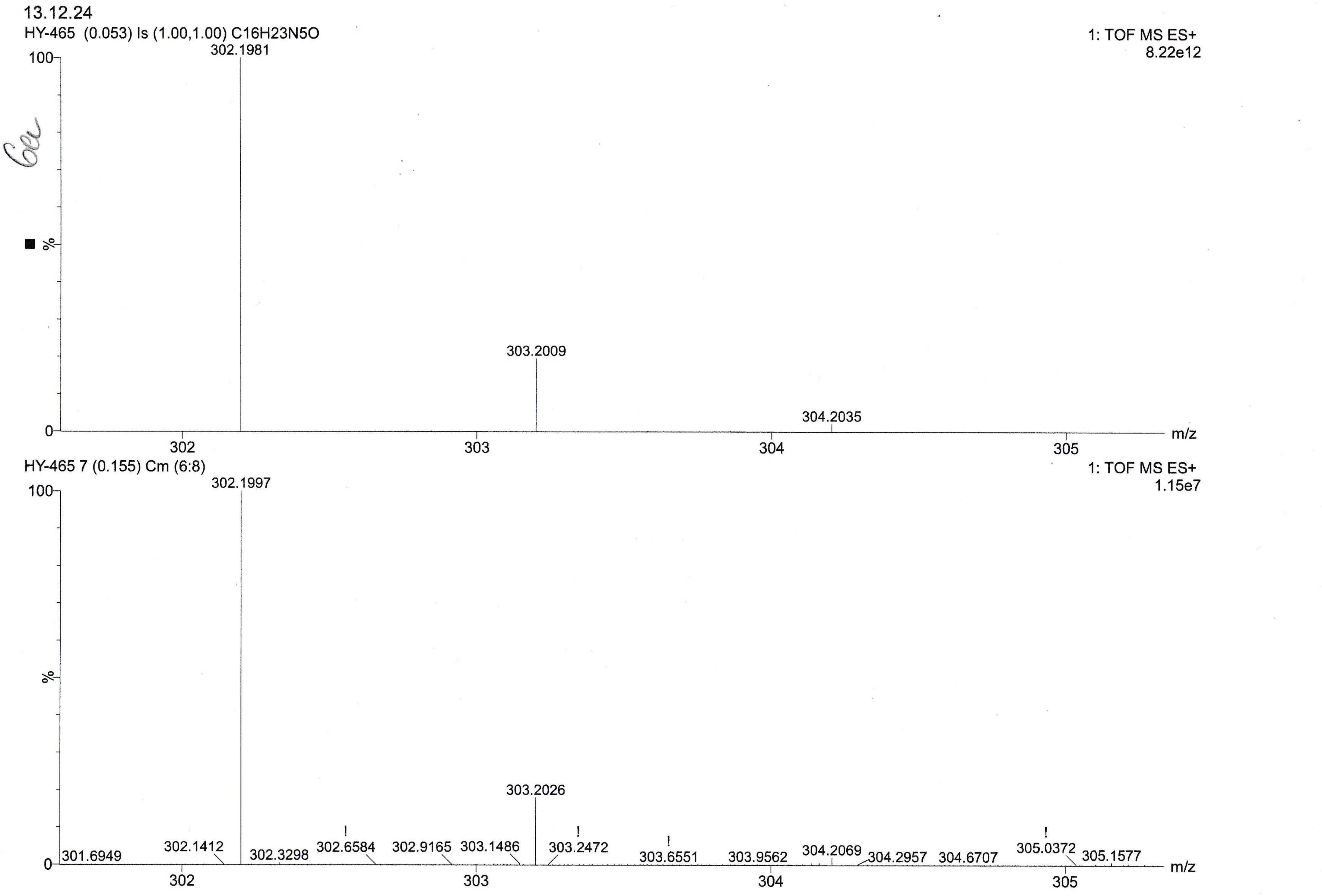
13.12.24
6a
HY-465 (0.053) Is (1.00,1.00) C16H23N5O
1: TOF MS ES+
8.22e12
100
%
0
302.1981
303.2009
304.2035
302
303
304
305
m/z
HY-465 7 (0.155) Cm (6:8)
1: TOF MS ES+
1.15e7
100
%
0
302.1997
303.2026
301.6949
302.1412
302.3298
302.6584
302.9165
303.1486
303.2472
303.6551
303.9562
304.2069
304.2957
304.6707
305.0372
305.1577
302
303
304
305
m/z

Molecular Structure Research Center, NAS RA, Yerevan, Armenia

null SF 400.1500036MHz Temp nullK TD null SWH null NS null LB 0.30 GB 0 solvent=null

February 4, 2025 11:

Avance 400 Neo
TopSpin 4.1.3

hy-481, 1H, DMSO/CCl4, temp=30

hy-481 1 1 F:/FID_BRUKER/NOCl_25

6b

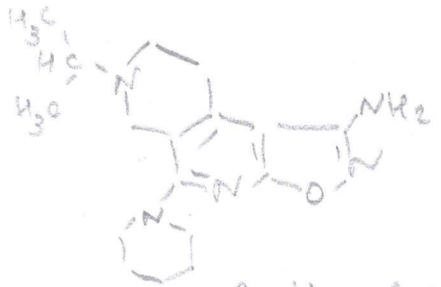

$C_{18}H_{25}N_5O$

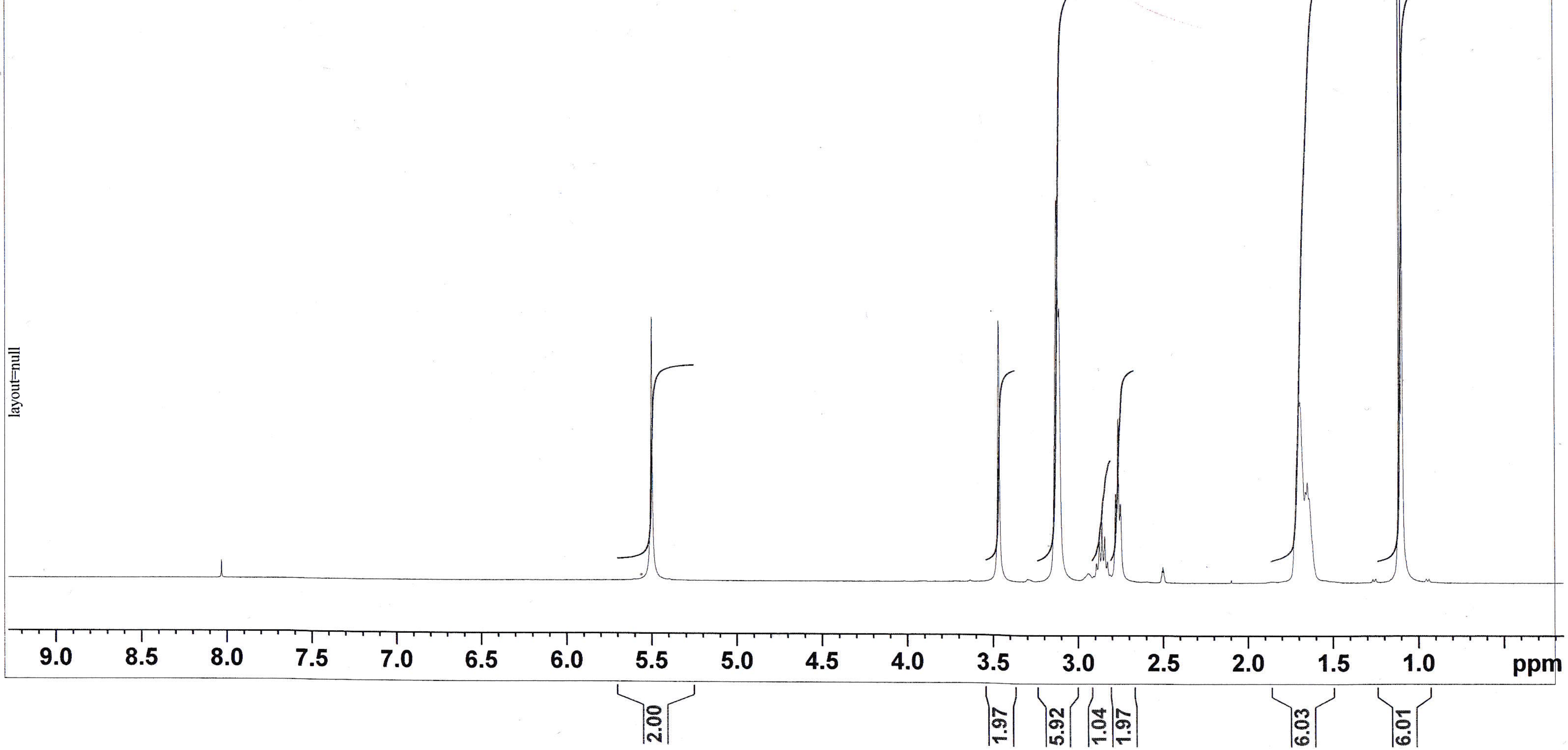

© Molecular Structure Research Center, NAS RA, Yerevan, Armenia

null SF = 100.6178650MHz Temp = nullK TD = null SWH = null NS = null LB = 1.00 GB = 0 solvent: null

February 4, 2025 11:

Avance 400 Neo

TopSpin 4.1.3

6b

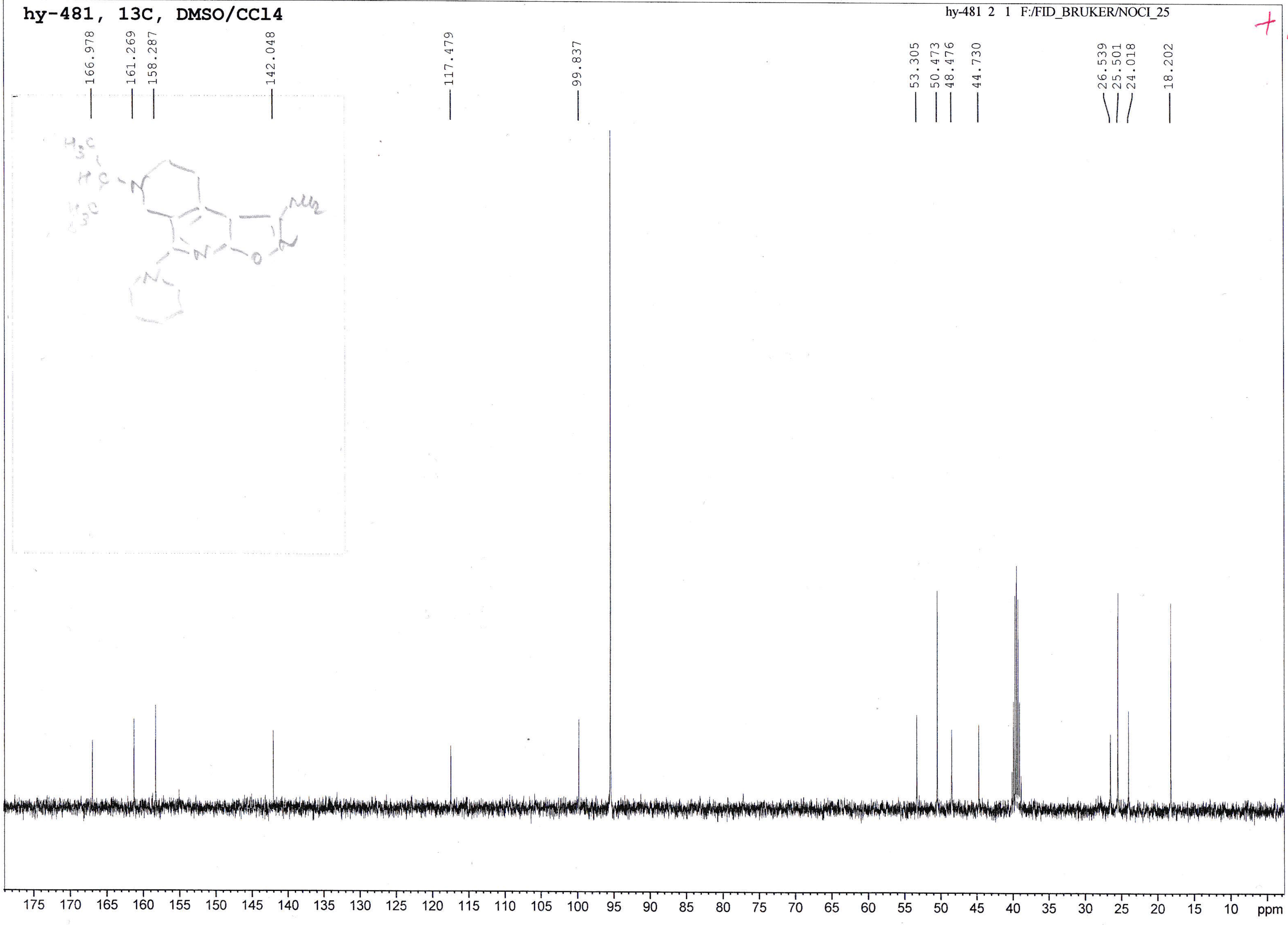

6b

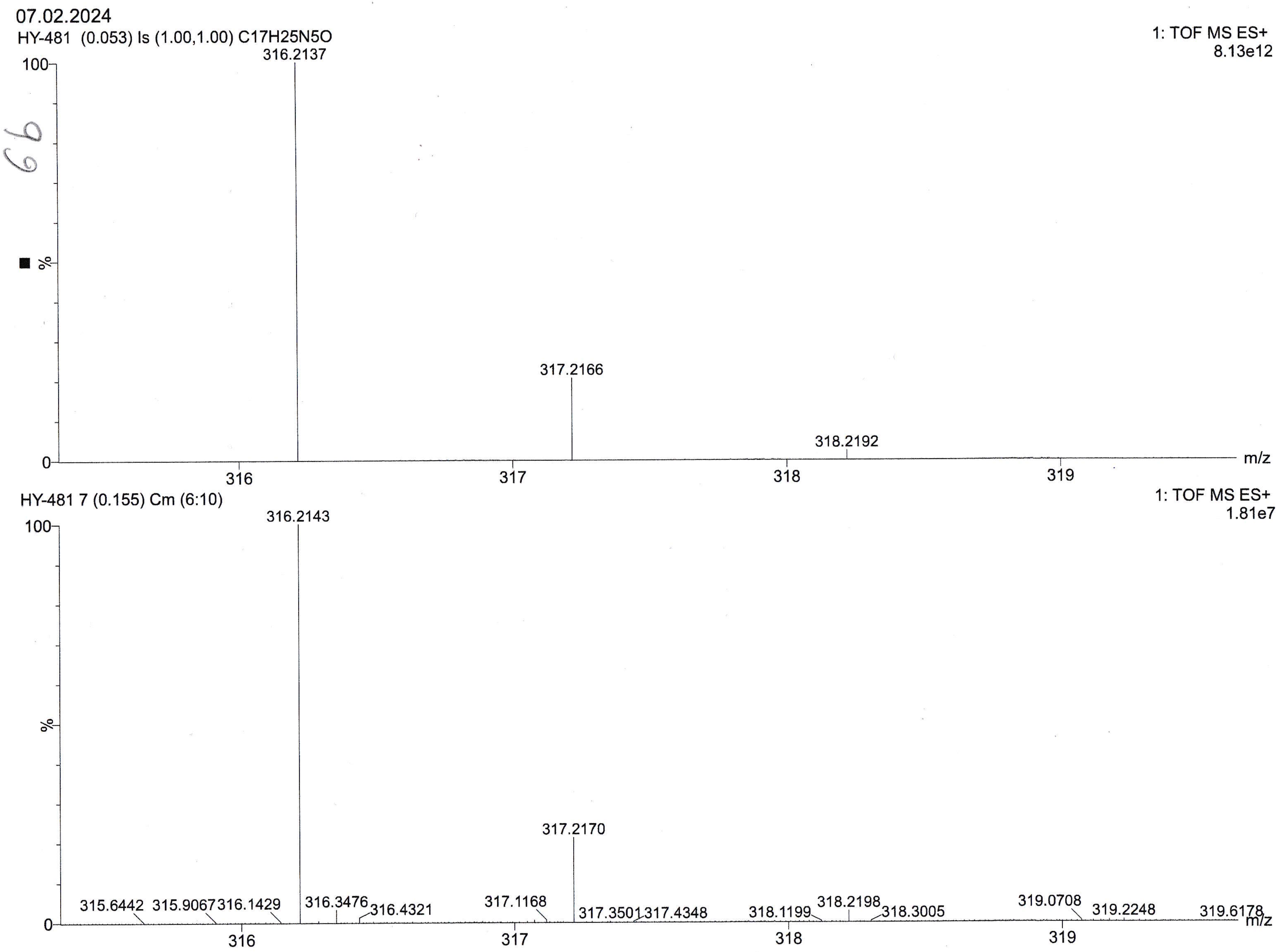
07.02.2024
HY-481 (0.053) Is (1.00,1.00) C17H25N5O
1: TOF MS ES+
8.13e12
316.2137
317.2166
318.2192
100
%
0
316
317
318
319
m/z
HY-481 7 (0.155) Cm (6:10)
1: TOF MS ES+
1.81e7
316.2143
317.2170
315.6442
315.9067
316.1429
316.3476
316.4321
317.1168
317.3501
317.4348
318.1199
318.2198
318.3005
319.0708
319.2248
319.6178
100
%
0
316
317
318
319
m/z

Molecular Structure Research Center, NAS RA, Yerevan, Armenia

null SF 400.1500036MHz Temp nullK TD null SWH null NS null LB 0.30 GB 0 solvent=null

February 11, 2025 4:

Avance 400 Neo

TopSpin 4.1.3

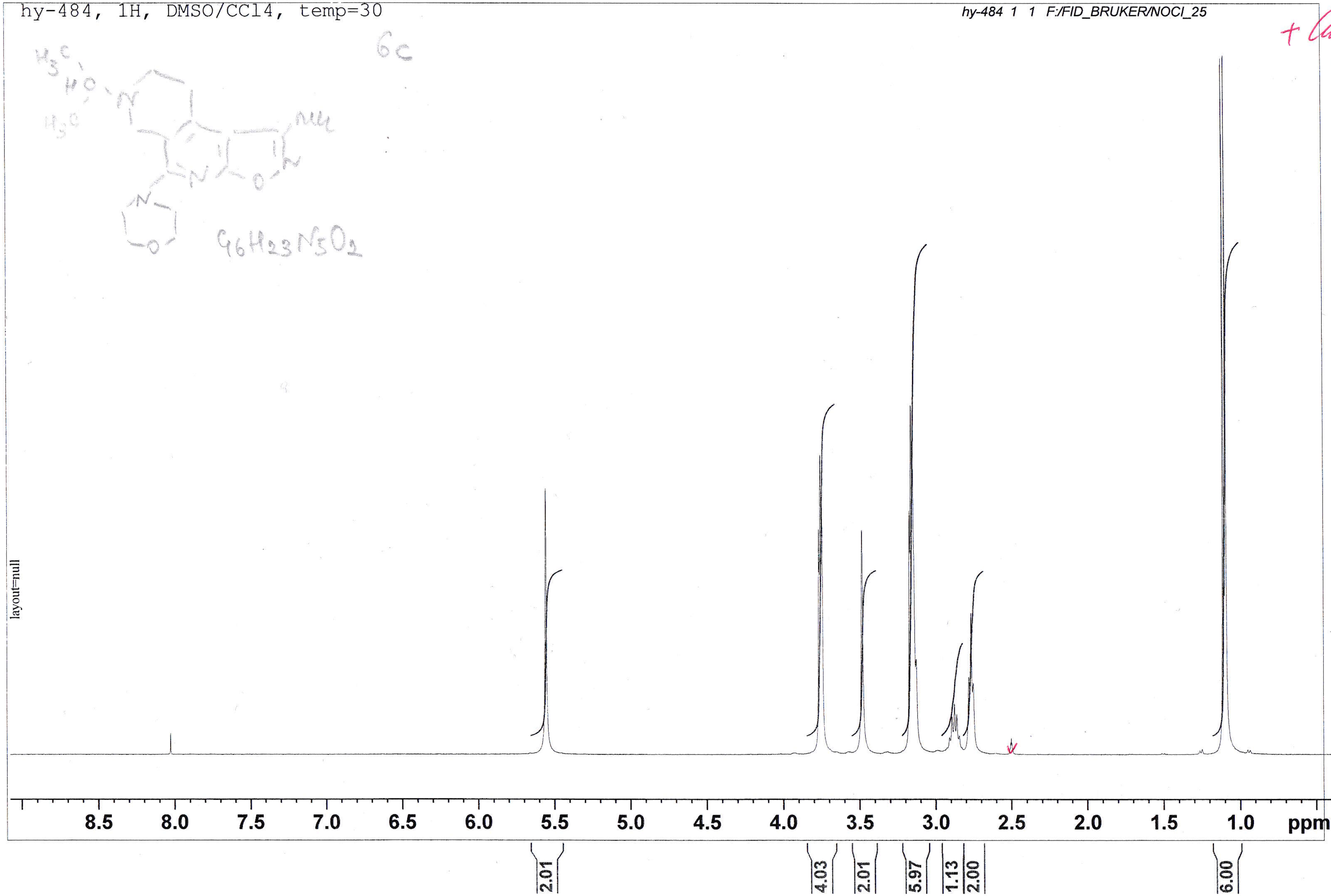

6c

© Molecular Structure Research Center, NAS RA, Yerevan, Armenia

null SF = 100.6178642MHz Temp = nullK TD = null SWH = null NS = null LB = 1.00 GB = 0 solvent: null

February 11, 2025 4:

Avance 400 Neo

TopSpin 4.1.3

hy-484 2 1 F:/FID_BRUKER/NOCI_25

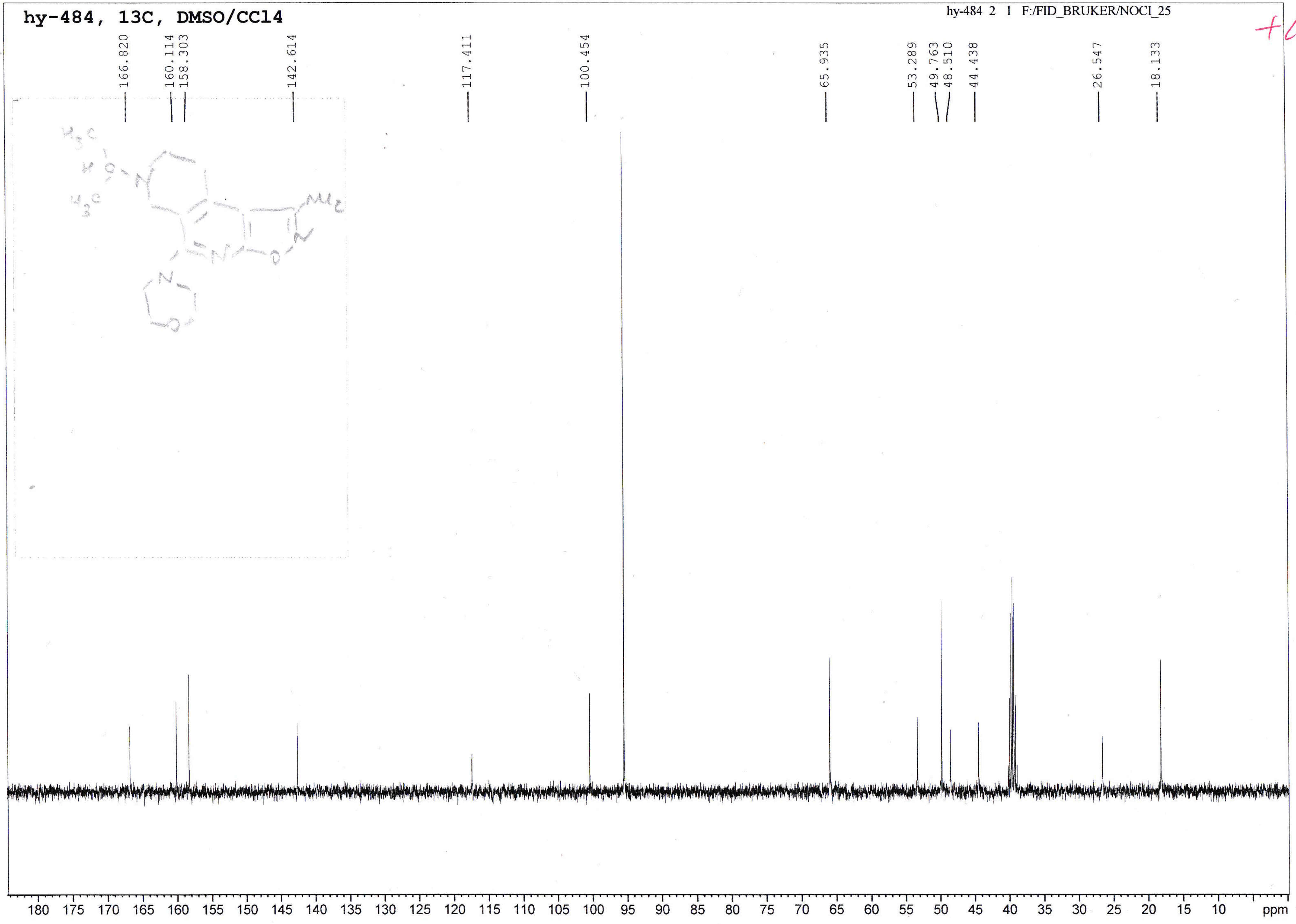

Molecular Structure Research Center, NAS RA, Yerevan, Armenia

null SF 400.1500036MHz Temp nullK TD null SWH null NS null LB 0.30 GB 0 solvent=null

January 17, 2025 12:

Avance 400 Neo

TopSpin 4.1.3

hy-471, 1H, DMSO/CCL4, temp=30

hy-471 1 1 F:/FID_BRUKER/NOCI_25

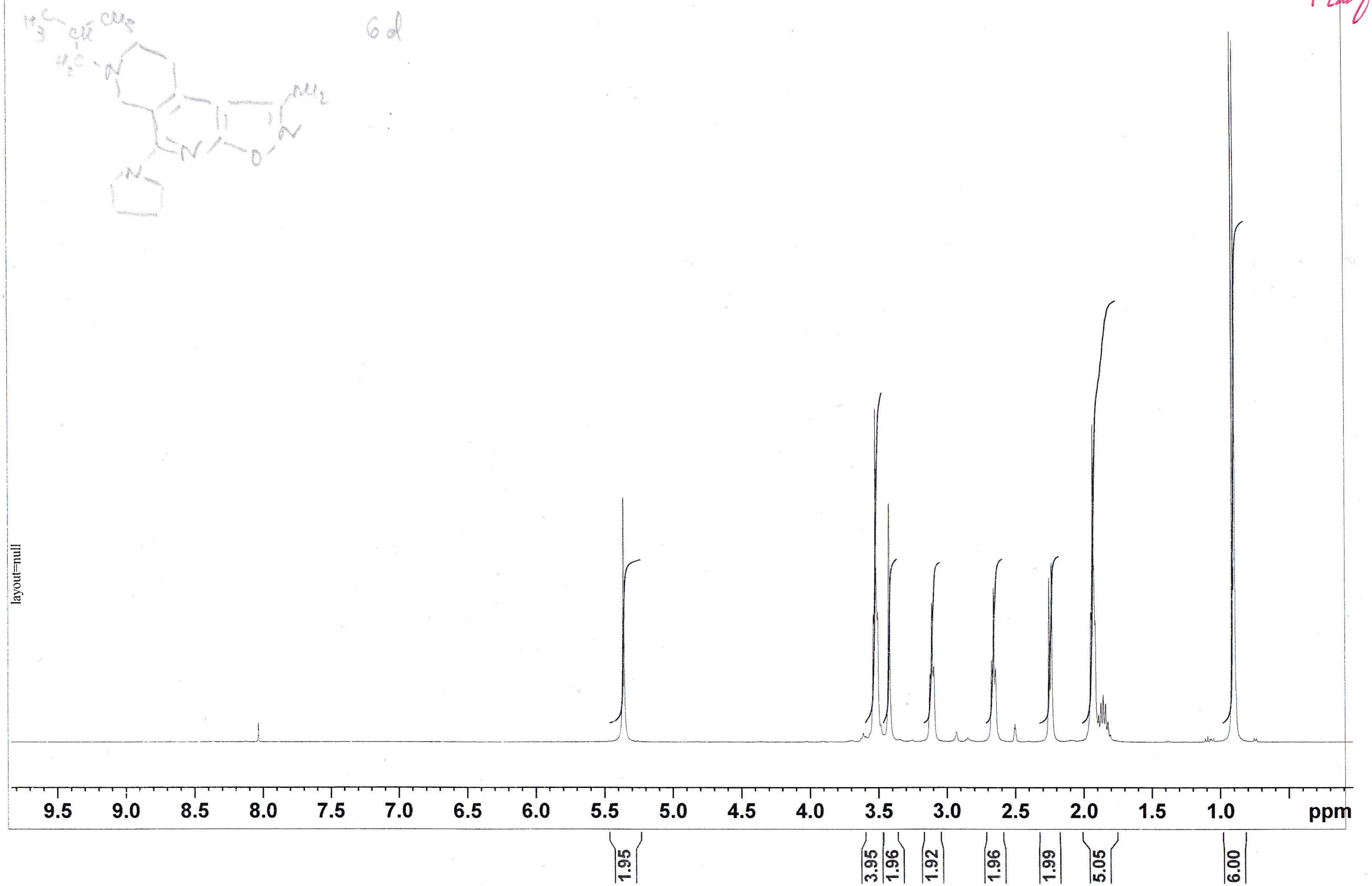

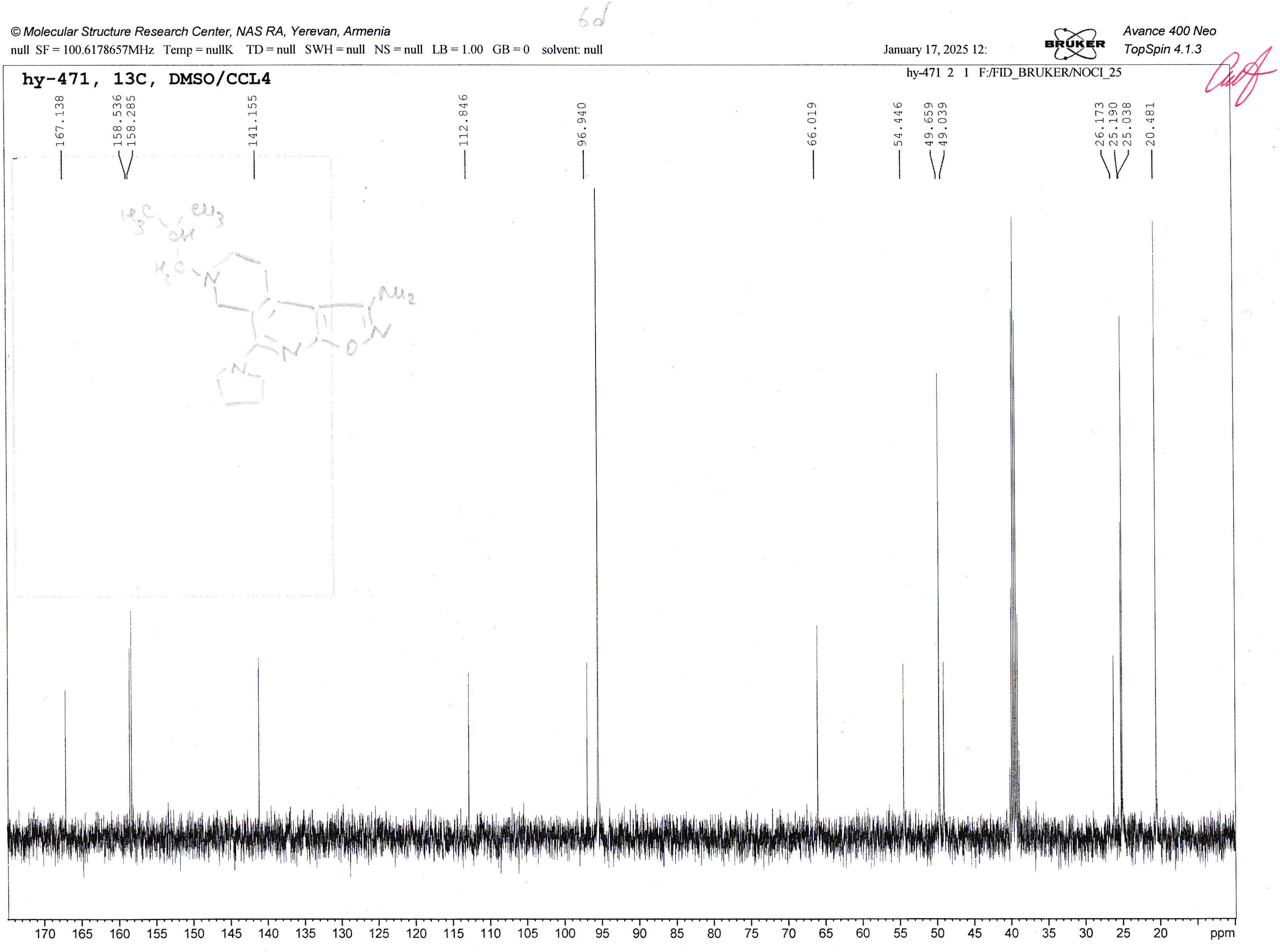
© Molecular Structure Research Center, NAS RA, Yerevan, Armenia
null SF = 100.6178657MHz Temp = nullK TD = null SWH = null NS = null LB = 1.00 GB = 0 solvent: null
January 17, 2025 12:
Avance 400 Neo
TopSpin 4.1.3
hy-471, 13C, DMSO/CCL4
hy-471 2 1 F:/FID_BRUKER/NOCI_25
167.138
158.536
158.285
141.155
112.846
96.940
66.019
54.446
49.659
49.039
26.173
25.190
25.038
20.481
170 165 160 155 150 145 140 135 130 125 120 115 110 105 100 95 90 85 80 75 70 65 60 55 50 45 40 35 30 25 20 ppm

6d

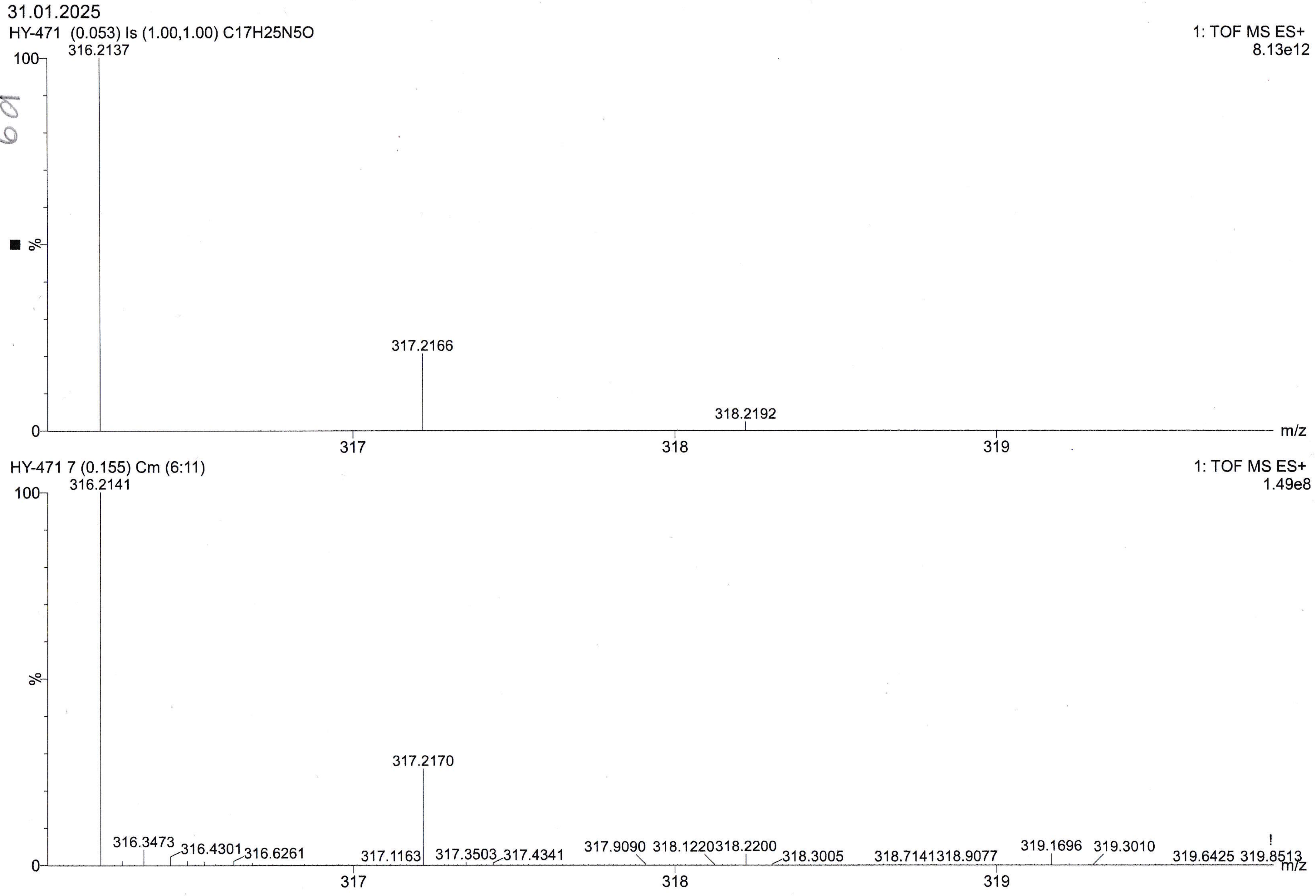

31.01.2025
HY-471 (0.053) Is (1.00,1.00) C17H25N5O
1: TOF MS ES+
8.13e12
100
%
0
316.2137
317.2166
318.2192
317
318
319
m/z
HY-471 7 (0.155) Cm (6:11)
1: TOF MS ES+
1.49e8
316.2141
316.3473
316.4301
316.6261
317.1163
317.2170
317.3503
317.4341
317.9090
318.1220
318.2200
318.3005
318.7141
318.9077
319.1696
319.3010
319.6425
319.8513
